# Enantioselective α-Arylation of Ketones via a Novel Cu(I)–Bis(phosphine) Dioxide Catalytic System

**DOI:** 10.1021/jacs.0c13236

**Published:** 2021-02-26

**Authors:** Margarita Escudero-Casao, Giulia Licini, Manuel Orlandi

**Affiliations:** Department of Chemical Sciences, University of Padova, via Marzolo 1, 35131 Padova, Italy; CIRCC−Consorzio Interuniversitario per le Reattività Chimiche e la Catalisi, University of Padova, via Marzolo 1, 35131 Padova, Italy

## Abstract

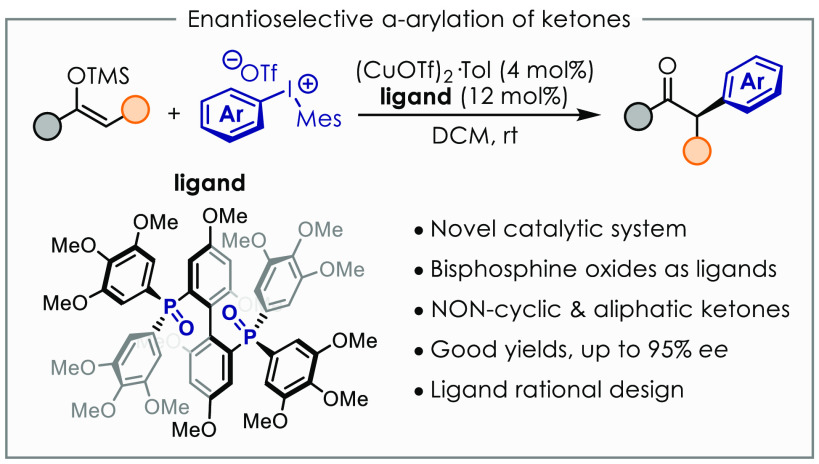

A novel catalytic system based on copper(I) and chiral bis(phosphine) dioxides is described. This allows the arylation of silyl enol ethers to access enolizable α-arylated ketones in good yields and enantiomeric excess up to 95%. Noncyclic ketones are amenable substrates with this method, which complements other approaches based on palladium catalysis. Optimization of the ligand structure is accomplished via rational design driven by correlation analysis. Preliminary mechanistic hypotheses are also evaluated in order to identify the role of chiral bis(phosphine) dioxides.

The Pd-catalyzed α-arylation of carbonyl compounds is a fundamental reaction in transition metal catalysis, which was first reported by the groups of Buchwald and Hartwig in 1997.^1,2^ Since then, this transformation has been successfully employed in academia and industry.^3^ As a result, several groups engaged in the development of enantioselective variants of this transformation.^4^ However, despite several transformations having been developed, the vast majority of these rely on the formation of quaternary stereocenters.^5−14^ Only a few reports have been published that allow for the formation of tertiary stereocenters.^15−21^ This limitation is because of the facile postreaction racemization via product enolization, which is promoted by the strong bases typically required in these reactions.

The first example to set an enolizable stereocenter was reported by Zhou and co-workers in 2011,^18^ who developed the enantioselective Pd-catalyzed α-arylation of esters (Figure 1A). In this work, silyl ketene acetals were employed as substrates to preactivate the carbonyl compound. This modification allowed avoiding the use of strong bases and consequent racemization as previously reported.^22^ Similarly, Gaunt and MacMillan showed that TMS-enol ethers of imides are amenable of enantioselective α-arylation by diaryliodonium salts in the presence of either Cu(I)– or Cu(II)–BOX catalysts (Figure 1B).^16,17^ MacMillan also demonstrated that aldehydes could be α-arylated via enamine catalysis in the presence of diaryliodonium salts and CuBr (Figure 1C).^15^

**Figure 1:**
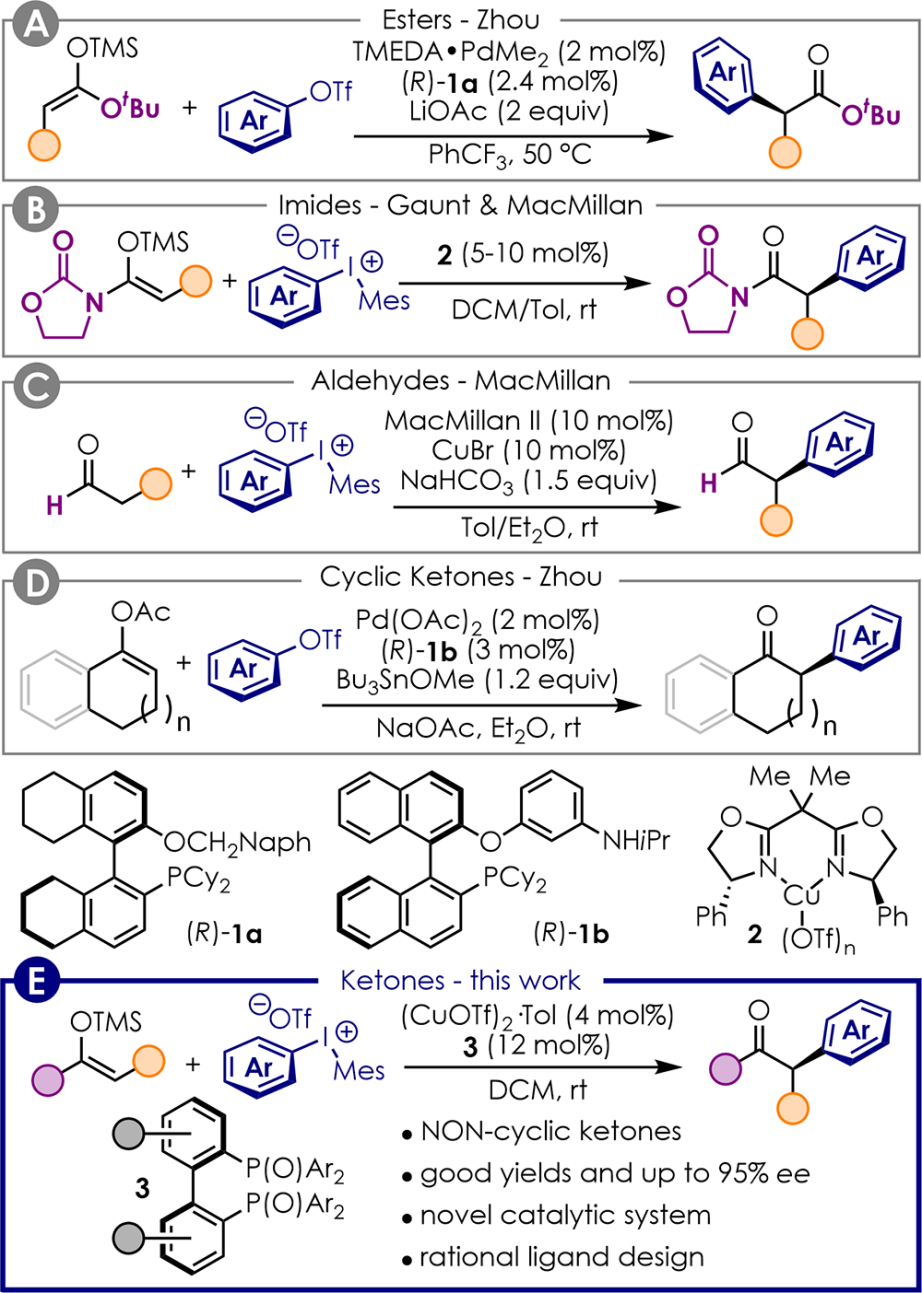
State of the art for the enantioselective α-arylation of carbonyl compounds to set tertiary stereocenters.

Although aldehydes could be activated via enamine catalysis and carboxyl derivatives as TMS-enolates, the α-arylation of ketones proved more challenging. Ketones are less prone to condensation with an amine catalyst than aldehydes, and silyl enol ethers are less nucleophilic than silyl ketene acetals.^23^ However, Zhou and co-workers found that the more nucleophilic Bu_3_Sn-enolates react smoothly under their typical conditions using Pd-catalysis and ligand **1b** (Figure 1D).^21^ Sn-enolates could be generated *in situ* from the corresponding alkenyl acetate in the presence of stoichiometric Bu_3_SnOMe. However, even though efficient for the α-arylation of cyclic ketones, this method proved unsuitable for the arylation of noncyclic substrates. Therefore, a general procedure for the enantioselective α-arylation of ketones is still missing. We herein show that silyl enol ethers of noncyclic ketones can be arylated in enantioselective fashion for the first time by means of a novel Cu(I) catalytic system featuring chiral bis(phosphine) dioxides as ligands (Figure 1E).

Initially, silyl enol ether **4a** was found to react smoothly with diaryliodonium salt **5a** to give the corresponding racemic α-arylated product **6a** in the presence of Cu(OTf)_2_ in DCM. In an effort to render this transformation enantioselective, we found that adding (*R*)-BINAP under aerobic conditions provided the desired product in 72% yield and 74:26 er. Given the propensity of phosphines to be oxidized and of Cu to undergo redox events, several combinations of (*R*)-BINAP, (*R*)-BINAP(O) (monoxide of (*R*)-BINAP), or (*R*)-BINAPO **3a** with Cu(OTf)_2_ or (CuOTf)_2_·Tol were tested under an inert atmosphere (see the Supporting Information). Although *chiral* bis(phosphine) dioxides are well-known Lewis base catalysts,^24,25^ to the best of our knowledge, only three reports exist about their use in combination with transition metal catalysis.^26−28^ Therefore, we were surprised to find that the reaction was indeed promoted by a combination of Cu(I) and chiral bis(phosphine) dioxides **3a**. A number of other classes of ligands were tested that proved unsuitable for this transformation (see the Supporting Information). Complexes **2** previously used for the arylation of silyl ketene imides (Figure 1B)^16,17^ were among those that showed no conversion.

Having identified a suitable class of ligands, a number of bis(phosphine) dioxides **3** were easily obtained from their corresponding commercially available phosphines. The enantioselectivities obtained are summarized in Figure 2A (for reaction yields, see the Supporting Information). In general, the GARPHOSO (**3i**–**3m**) and SEGPHOSO (**3n**–**3p**) scaffolds provided the best performances, with DMM-GARPHOSO **3l** being the best commercially available candidate tested (er 86:14). Electron-poor ligands such as **3m** gave low reactivity, and increasing the steric hindrance of the aryl groups was detrimental for the enantioselectivity (see DTBM-SEGPHOSO **3p**). Even though **3a**–**3u** did not give acceptable selectivity, we reasoned that an optimal ligand structure could be accessed via correlation analysis, assuming that a proper descriptor could be found. This would be preferable to a trial-and-error approach and would reduce the effort toward the synthesis of new candidates.

**Figure 2:**
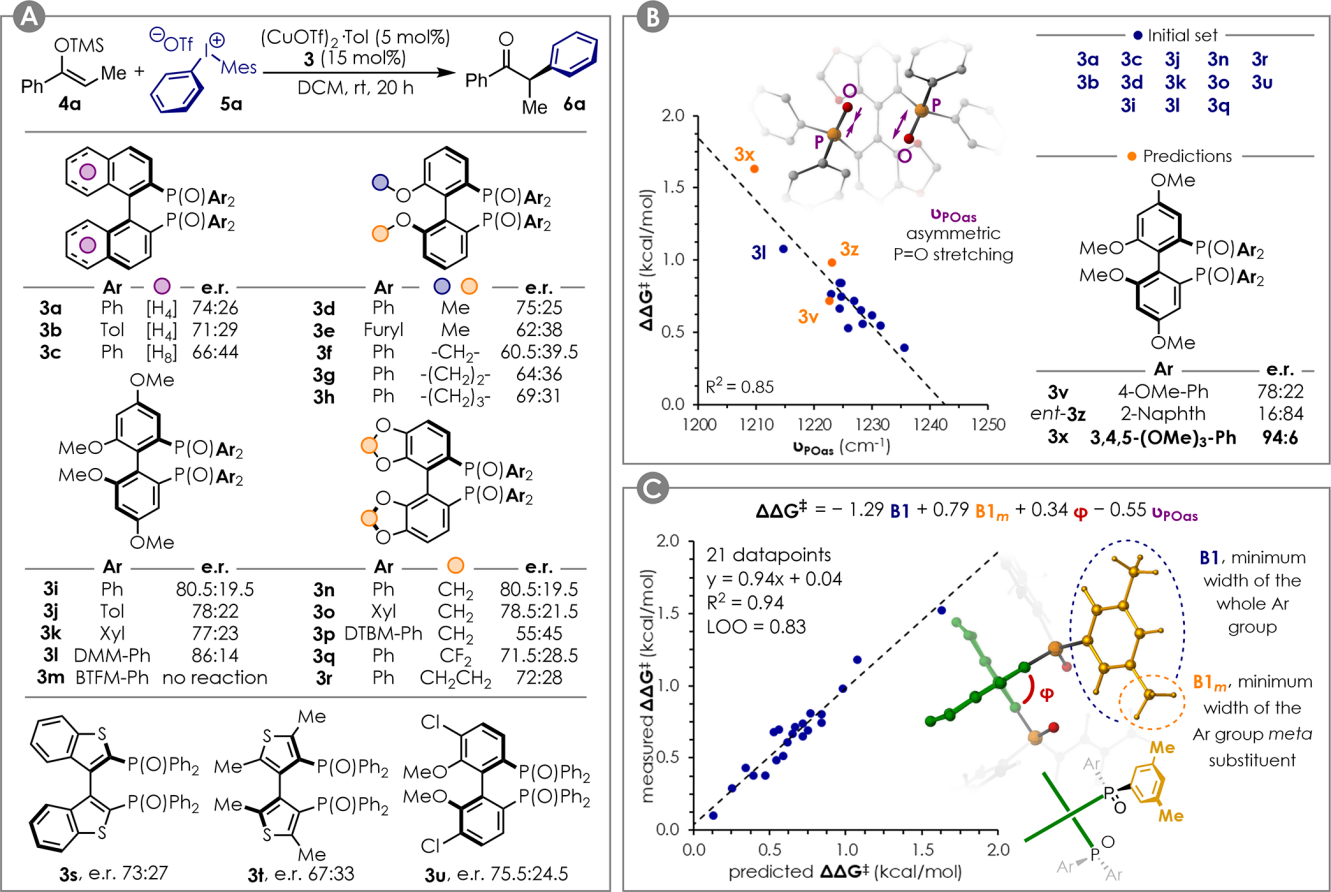
(A) Benchmark reaction and ligand screening. Yields are reported in the Supporting Information. (B) Single parameter correlation between the measured enantioselectivity and the ligand P=O stretching frequency for an unbiased ligands subset. (C) Multidimensional correlation between the ligand structure and the observed enantioselectivity for a ligand set excluding **3t**, **3m**, and **3e**. Tol = 4-Me-Ph, Xyl = 3,5-Me_2_-Ph, DMM = 4-OMe-3,5-Me_2_, BTFM = 3,5-(CF_3_)_2_, DTBM = 4-OMe-3,5-*t*Bu_2_.

The structures of ligands **3a**–**3u** were optimized at the M06-2*X*/6-31G(d) level. The vibrational analysis was performed at the same level of theory to access a number of frequencies that could be relevant descriptors for the electronic properties of the ligand. These included υ_POas_, the frequency of the asymmetric P=O bonds stretching.

Interestingly, when reducing the data set by removing candidates affected by strong steric or structural biases (i.e., **3e**–**3h**, **3p**, **3s**, and **3t**), the correlation in Figure 2B was obtained (blue dots). This relates the reaction enantioselectivity (expressed as ΔΔ*G*^⧧^ in kcal/mol) with υ_POas_, suggesting important effects induced by the electronics of the ligand at the diastereomeric transitions states. Due to additivity of the Hammett σ parameters, a simple comparison of the selectivity for **3i**–**3l** would indicate **3v** as a promising ligand. On the other hand, *virtual* evaluation and predictions given by the correlation in Figure 2B suggested **3v** to be average (predicted er: 80:20) and identified **3x** as the best candidate (predicted er: 90:10). Ligands **3v**, *ent-***3z**, and **3x** were therefore synthesized to test our model. Pleasingly, the correlation was found to be obeyed (orange dots in Figure 2b), with **3x** providing product **6a** in 94:6 er and 58% yield.

Adding **3v**, *ent-***3z**, and **3x** to the whole data set, the selectivity range was extended to ca. 1.5 kcal/mol, allowing for more statistically sound multidimensional linear regression analysis to be performed.^29−32^ This would allow accounting for additional steric and structural effects. Other descriptors acquired for the ligands included: (i) **B1**, **B5**, and **L**, Verloop Sterimol parameters^33^ accounting for the minimum width, maximum width, and length of the whole Ar group; (ii) **B1_*x*_**, **B5**_***x***_, and **L**_***x***_ (where *x* = *m* or *p*), Verloop Sterimol parameters accounting for the minimum width, maximum width, and length of the *meta*- or *para*-substituents of the Ar group; (iii) **%V_*x*_** (where *x* = *m* or *p*), buried volume^34^ of a 3.5 Å sphere placed on the *meta*- or *para*-substituent of the Ar group; ***d*_PO_**, P=O bond length; and **φ**, dihedral angle of the scaffold biaryl moiety. When applying the multivariate linear regression procedure, the model in Figure 2C was obtained. In addition to **υ_POas_**, parameters **B1**, **B1_*m*_**, and **φ** appeared in the model, the former two with opposite coefficient sign. Since also **φ** is likely affected by the size of the Ar groups, this suggests that a fine balance between steric hindrance and geometrical features is required in order to achieve optimal selectivity. The model presents a good quality of fit with *R*^2^ = 0.94 and was proven to be robust by leave-one-out cross validation (LOO *Q*^2^ = 0.83).

With the optimized ligand **3x**, the reaction scope was evaluated (Figure 3). Silyl enol ethers bearing electron-donating substituents in *meta*- or *para*-positions were found to react smoothly to access good yields and er ≥95:5 (**6b**–**6e**, **6h**, **6j**). Despite good reactivity, **6i** gave lower selectivity (er 83:17) likely due to the geometrical bias provided by the *ortho*-OMe group. Decreasing the nucleophilicity of the Si-enolate resulted in diminished chemical activity (**6f**, **6g**). However, we found that changing the ligand to *ent-***3z** for these substrates was beneficial. Despite the lower p*K*_a_ of **6g** due to the CF_3_ group, the er at 6 and 24 h was conserved (92:8 er).

**Figure 3:**
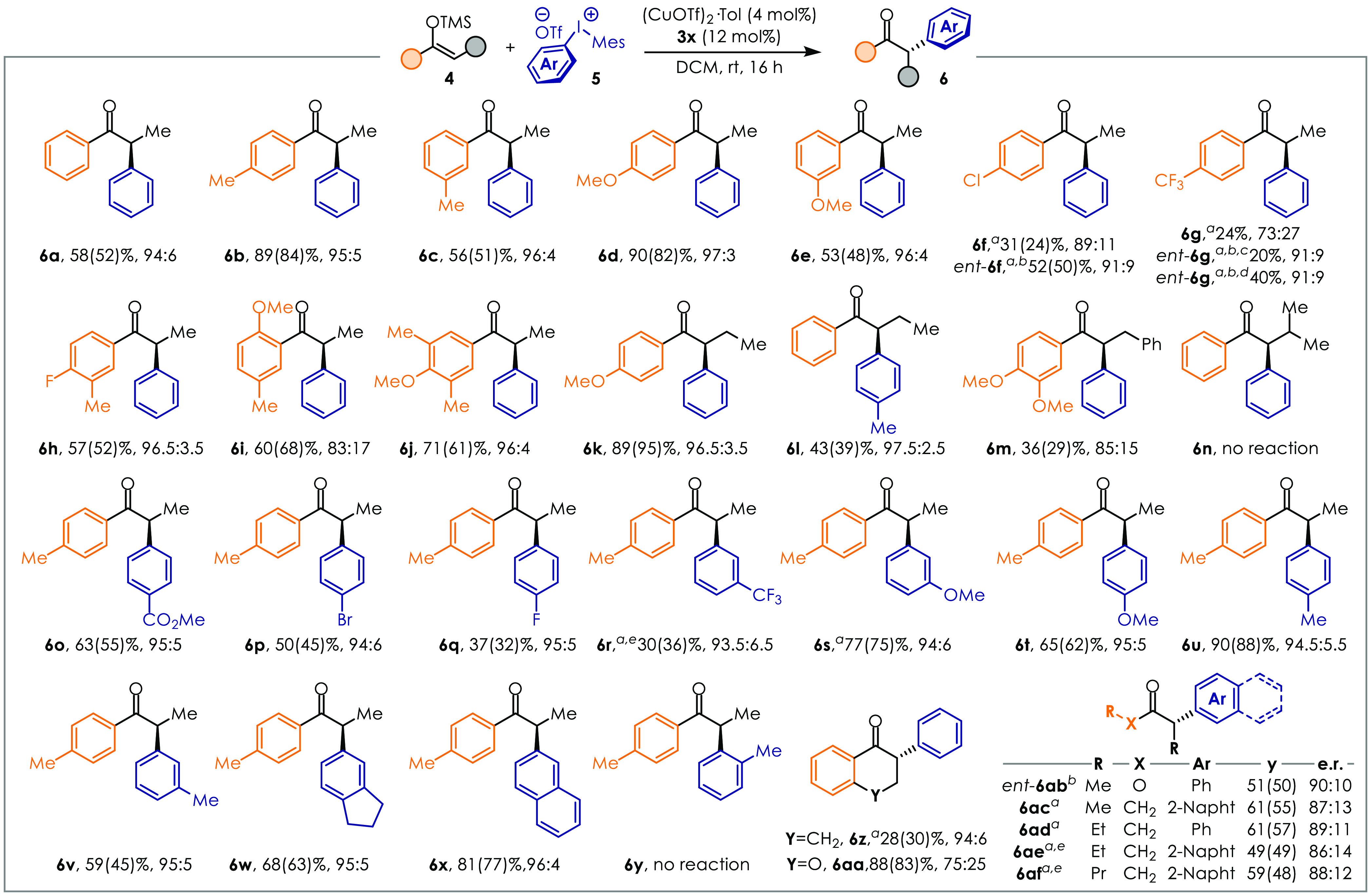
Reaction scope. Yields and selectivity are expressed according with the formula NMR *y*(isol. *y*)%, er. Conditions: diaryliodonium salt **5** (0.1 mmol), silyl enol ether **4** (0.2 mmol), (CuOTf)_2_·Tol (4 mol %), **3x** (12 mol %), DCM (0.5 mL). (a) 3.0 equiv of silyl enol ether **4** was used. (b) *ent-***3z** was used instead of **3x**. (c) Reaction time: 6 h. (d) Reaction time: 24 h. (e) The er was determined on the corresponding alcohol after reduction with LiAlH_4_.

Substrates with a longer α-substituent also resulted in high enantioselectivity, with products **6k** and **6l** being obtained in 96.5:3.5 and 97.5:2.5 er, respectively. *i*Pr or Bn groups onto the nucleophilic α-carbon are detrimental for the reactivity likely due to steric hindrance (**6m**, **6n**). Tetralone **6z** can be obtained in 94:6 er even though with modest yield, while the use of **4aa** resulted in high yields but lower selectivity. Therefore, this method is synthetically complementary to previous work by Zhou.^21^ Notably, linear dialkyl ketones were also suitable reaction partners with only slightly diminished efficiency (**6ac**–**6af**), which is unprecedented in enantioselective α-arylations. Moreover, methyl propionate *ent-***6ab** was obtained in 50% yield and 90:10 er (with ligand *ent-***3z**) showing that also esters are amenable to arylation under our catalytic conditions. Diaryliodonium salts **5** featuring different electronic and steric properties were also tested. Both electron-rich and electron-poor aryl groups reacted in typically good yields and high er (**6o**–**6x**) showing good generality. Steric hindrance next to the reaction site was detrimental, as the *ortho*-tolyl-substituted ketones **6y** and **6n** could not be obtained. Finally, a comparison of the optical rotation with products previously reported allowed the establishment that (*R*)-ligands lead to the formation of (*S*)-products. It should be noted that enantioenriched α-arylated noncyclic ketones of the type reported herein were only accessible via Kumada-type couplings from α-halogenated ketones or by photocatalytic acylation of benzyl radicals.^35−38^ Alternatively, a multistep strategy would need to be followed.^39^

Phosphine oxides are generally believed to be labile ligands in transition metal catalysis.^40−45^ Moreover, they are also known as excellent Lewis bases.^24,25^ As such, this class of compounds is capable of binding hypervalent iodine compounds with binding constant *K* ≃ 50–200 M^–1^ in DCM.^46^ Therefore, the question follows whether ligands **3** would operate as ancillary ligands at the Cu metal center (Figure 4B), or by activation of the arylating agent **5** by formation of a hypervalent Lewis acid–base adduct (Figure 4A).^24^ Preliminary clarification of the role of **3** in the reaction mechanism would help understand this novel catalytic system setting the basis for its future deployment in other transformations. Therefore, this was investigated in our benchmark reaction via initial rate kinetic analysis giving the plot in Figure 4. The plot of the reciprocal of the observed reaction rate constant 1/*k*_obs_ against the concentration of bis(phosphine) dioxides **3a** resulted in a straight line (*R*^2^ = 0.98) with positive slope. The kinetic order −1 suggests that **3a** is involved in a pre-equilibrium that requires it to dissociate from its acidic partner before the reaction can proceed. As phosphine oxides and diaryliodonium salts **5** form 1:1 complexes, the hypothesis that their combination would result in catalytic activity is in contrast with this kinetic data (Figure 4A). On the contrary, this data is consistent with a scenario where an inactive off-cycle complex [Cu(**3a**)_2_X*_n_*] is formed in the presence of a large excess of ligand. This complex would require a ligand molecule to dissociate before the catalytically active species [Cu(**3a**)X_*n*_] can enter into the catalytic cycle (Figure 4B). Clearly, this does not exclude the formation of Lewis complexes in solution. Therefore, even though phosphine oxides act as labile ligands in transition metal catalysis with soft second- and third-row metals,^40−45^ this work adds to precedents showing that these bind to first row transition metals such as Fe^26−28^ or Cu.^47,48^

**Figure 4:**
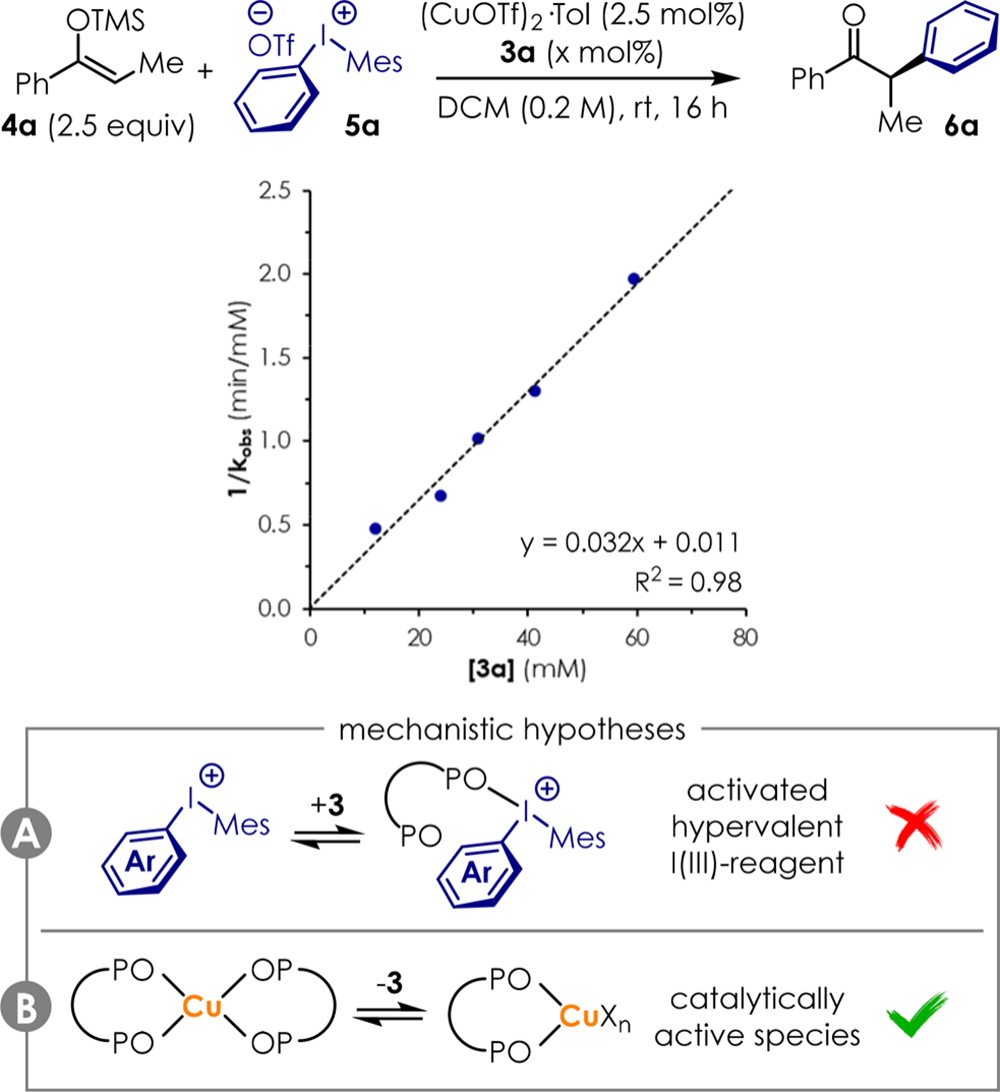
Preliminary mechanistic considerations. Bis(phosphine) dioxides **3** promote the reaction as the ligand to the Cu center rather than as a Lewis base.

In summary, we showed that a novel catalytic system featuring Cu(I) and bis(phosphine) dioxides **3** efficiently promotes the unprecedented enantioselective α-arylation of noncyclic silyl enol ethers. Oxides of commercially available bisphosphines provided selectivity up to 86:14 er. Therefore, ligand **3x** was identified by means of correlation analyses and synthesized in the enantiomerically pure form. This was found to outperform other ligands providing er up to 97.5:2.5. After evaluation of the reaction scope, we turned our attention to the role of **3** in catalysis. We found that, contrarily to common opinions, bis(phosphine) dioxides efficiently bind to the Cu center to promote the reaction as a ligand rather than as a Lewis base. In this instance, this transformation is an example of how new classes of ligands could give access to new reactivity thus underpinning the continuously increasing interest for the use of abundant base metals.^49−51^ Further investigations into the mechanism of this novel catalytic system and extension to other transformations are currently ongoing in our laboratories and will be reported in due course.

## Supporting Information Available

Experimental and computational details and characterization of all compounds (PDF)
