# Supplementary material for: Enantioselective α-Arylation of Ketones via a Novel Cu(I)–Bis(phosphine) Dioxide Catalytic System
Source: J Am Chem Soc. 2021 Feb 26;143(9):3289–94. doi: 10.1021/jacs.0c13236 (PMC8041290; doi:10.1021/jacs.0c13236)
Supplement: Supplementary file 1 — ja0c13236_si_001.pdf [file ja0c13236_si_001.pdf]

Enantioselective α-Arylation of Ketones via a Novel Cu(I)-Bis(phosphine) Dioxide Catalytic System

Margarita Escudero-Casao,[‡,†,+] Giulia Licini,[‡,†] Manuel Orlandi,[,‡,†,+]*

[‡]Department of Chemical Sciences, University of Padova, via Marzolo 1, 35131 Padova.
[†]CIRCC – Consorzio Interuniversitario per le Reattività Chimiche e la Catalisi, Padova Unit., via Marzolo 1, 35131 Padova.

Supporting Information

General Information S2

Reaction optimization S3

Preparation of silyl enol ethers S5

Synthesis of ligands S7

Characterization of arylated ketones S8

Multidimensional correlation analysis S16

Kinetic studies S20

HPLC traces S24

NMR spectra S34

Geometries of ligands S73

References S90

General Information

All reactions were carried out in oven- or flame-dried glassware under an atmosphere of dry Nitrogen unless otherwise noted. Except as otherwise indicated, all reactions were magnetically stirred and monitored by analytical thin layer chromatography (TLC) using Merck pre-coated silica gel plates with F_{254} indicator. Visualization was accomplished by UV light (254 nm), with combination of Cerium Ammonium Molybdate solution as an indicator. Flash column chromatography was performed using silica gel pore size 60 Å, 230-400 mesh particle size, 40-63 µm particle size or Aluminium oxide 90 active neutral. Yields refer to chromatographically and spectrographically pure compounds, unless otherwise noted. Commercial grade reagents and solvents were used without further purification. ^{1}H NMR, ^{13}C NMR, ^{19}F and ^{31}P spectra were recorded on Bruker Avance300 spectrometer. The proton spectra are reported as follows δ (position of proton, multiplicity, coupling constant *J*, number of protons). Multiplicities are indicated by s (singlet), d (doublet), t (triplet), q (quartet), p (quintet), h (septet), m (multiplet) and br (broad). Enantiomeric excess was determined on a Shimadzu HPLC SPD-10A with a variable wavelength detector using chiral stationary phase columns (0.46 cm x 25 cm) from Phenomenex or Daicel. Unless otherwise noted, all reagents were obtained commercially and used without further purification.

All the ketones used in this study were purchased from commercial suppliers (Sigma-Aldrich, TCI, or FluoroChem), except for 1-(4-Fluoro-3-methylphenyl)propan-1-one (**7**)[1], 1-(2-methoxy-5-methylphenyl)propan-1-one (**8**)[2], 1-(4-methoxy-3,5-dimethylphenyl)propan-1-one (**9**)[3], 1-(4-methoxyphenyl)butan-1-one (**10**)[4] and 1-(3,4-dimethoxyphenyl)-3-phenylpropan-1-one (**11**)[5], which were prepared according to known procedures and for which spectral data were in agreement with the literature data.

Diaryliodonium trifluoromethanesulfonates used in this study were prepared according to literature procedures and the spectroscopic characterization matched the literature data:[6] phenylmesityliodonium trifluoromethanesulfonate (**5a**), (4-methylphenyl)(mesityl) iodonium trifluoromethanesulfonate (**5b**), (4-(methoxycarbonyl)phenyl)(mesityl)iodonium trifluoromethanesulfonate (**5c**), (4-bromophenyl)(mesityl) iodonium trifluoromethanesulfonate (**5d**), (4-Fluorophenyl)(mesityl) iodonium trifluoromethanesulfonate (**5e**), (3-trifluoromethoxy)phenyl)(mesityl)iodonium trifluoromethanesulfonate (**5f**), (3-(methoxycarbonyl)phenyl)(mesityl)iodonium trifluoromethanesulfonate (**5g**), (4-methoxyphenyl)(mesityl) iodonium trifluoromethanesulfonate (**5h**), (3-methylphenyl)(mesityl) iodonium trifluoromethanesulfonate (**5i**), (2,3-dihydro-1*H*-inden-5-yl)(2,4,6-trimethylphenyl)iodanium trifluoromethanesulfonate (**5j**) and (2-naphthyl)(mesityl)iodonium trifluoromethanesulfonate (**5k**).

Reaction optimization

We initially found that $Cu(OTf)_2$ could catalyze the arylation of **4a** with **5a**. However, the addition of any ligand to the reaction would result in no conversion of the reagents. However, performing the reaction with a phosphine under aerobic conditions rendered the product in good yield and enantioenriched fashion. A series of tests was run that allowed establishing that $(CuOTf)_2Tol$ in the presence of a phosphine oxide could promote the reaction.

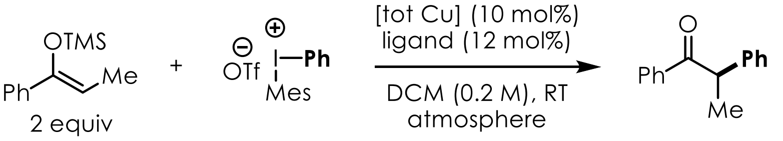

[Cu]	ligand	atmosphere	y (%)	ee (%)
$Cu(OTf)_2$	**7a**	N_2	< 2	n.d.
$Cu(OTf)_2$	**7a**	air	72	45-48
$Cu(OTf)_2$	**7a**	O_2	36	45-48
$Cu(OTf)_2$	**7a**	O_2 then N_2*	92	45-48
$Cu(OTf)_2$	**7b**	N_2	< 2	n.d.
$Cu(OTf)_2$	**3a**	N_2	< 2	n.d.
$(CuOTf)_2$•Tol	**7a**	N_2	< 2	n.d.
$(CuOTf)_2$•Tol	**7b**	N_2	< 2	n.d.
$(CuOTf)_2$•Tol	**3a**	N_2	68	45-48
$(CuOTf)_2$•Tol (2.5 mol%)	**3a** (6 mol%)	N_2	90	45-48

* Stir **7a** and $Cu(OTf)_2$ under oxygen for 30 min then change to nitrogen

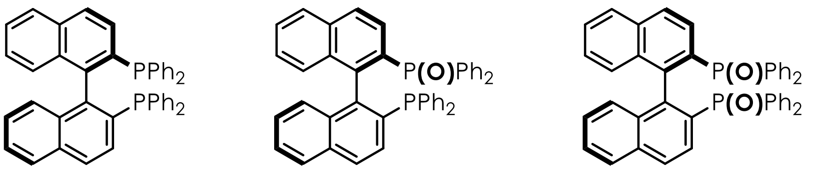

(*R*)-**BINAP, 7a** (*R*)-**BINAP(O), 7b** (*R*)-**BINAPO, 3a**

A number of other ligands were tested in combination with Cu(I), but these gave **no reactivity**:

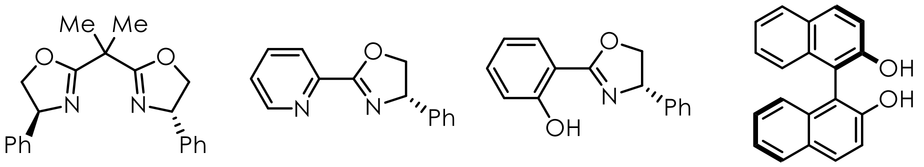

(*S*,*S*)-**Ph-BOX** (*S*)-**Ph-PyOx** (*S*)-**10** (*R*)-**BINOL**

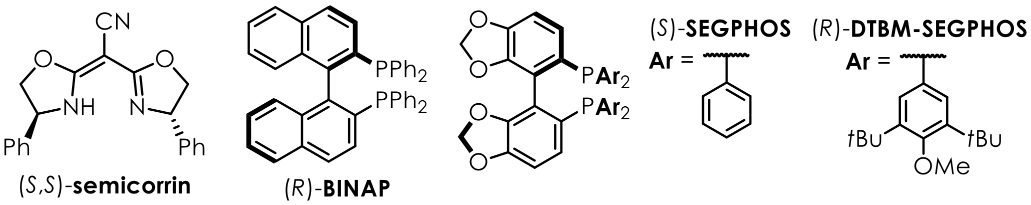

(*S*,*S*)-**semicorrin** (*R*)-**BINAP**

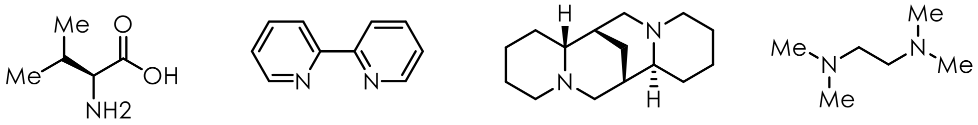

(*S*)-**val-OH** **bipy** **sparteine** **TMEDA**

The reaction was tested in different solvents. No conversion was obtained but for DCM, $CHCl_3$, or DCE.

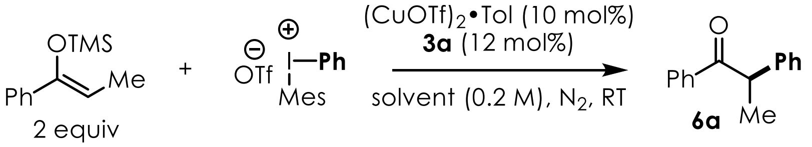

solvent	y (%)	ee (%)
**ACN**	< 2	n.d.
**AcOEt**	< 2	n.d.
**Tol**	< 2	n.d.
**THF**	< 2	n.d.
**Et2O**	< 2	n.d.
**CHCl3**	63	43
**DCE**	48	45

Finally, several ligands were tested in order to optimize the enantioselectivity:

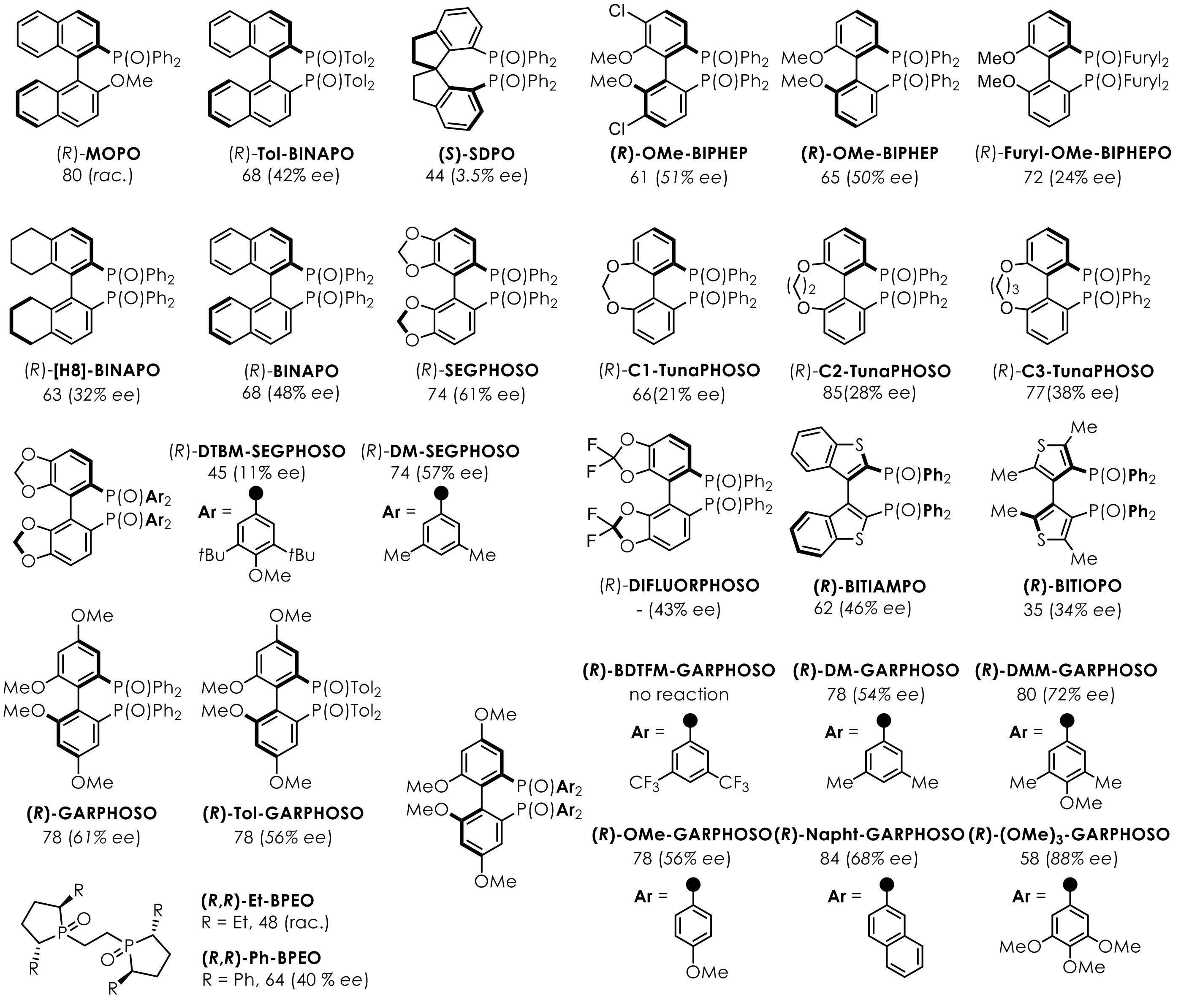

Preparation of silyl enol ethers

Silyl enol ethers **4** used in this study were prepared according to a General procedure A and the spectral data are in agreement with literature data: (*Z*)-(1-phenylprop-1-en-1-yl)oxy)trimethylsilane (**4a**)[7], (*Z*)-trimethyl-(1-p-tolylprop-1-enyloxy)silane (**4b**)[7], (*Z*)-(1-(4-methoxyphenyl)prop-1-enyloxy)trimethylsilane (**4d**)[7], (*Z*)-(1-(4-chlorophenyl)prop-1-enyloxy)-trimethylsilane (**4f**)[8], (*Z*)-(1-phenylbut-1-enyloxy)trimethylsilane (**4k**)[9], ((3,4-dihydronaphthalen-1-yl)oxy)trimethylsilane (**4m**)[10], (*Z*)-(Hept-3-en-4-yloxy)trimethylsilane (**4n**)[10], [(6-Methoxy-3,4-dihydro-1-naphthalenyl)oxy](tri-methyl)silane (**4p**)[11], 4-trimethylsilyloxy-1,2-benzopyran (**4q**)[12], (*Z*)-(1-(4-(trifluoromethyl)phenyl)prop-1-en-1-yl)oxy)trimethylsilane (**4y**)[13], (*Z*)-(pent-2-en-3-yloxy)trimethylsilane (**4z**)[14], (1-Butyl-pent-1-enoyl)-trimethyl-silane (**4x**)[15] and (1*E*)-1-Methoxy-1-(trimethylsiloxy)propene (**4aa**)[16].

General procedure A: preparation of the silyl enol ethers

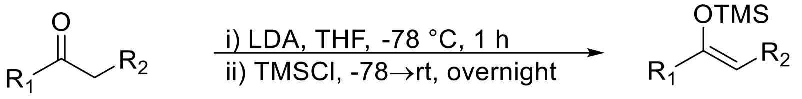

A solution of diisopropylamine (1.95 mL, 13.96 mmol) in THF (40 mL) was cooled to 0 °C and a 2.5 M solution of *n*-butyllithium in n-hexane (5.58 mL, 13.96 mmol) was added slowly by a syringe. This mixture was stirred for 20 min at 0 °C and subsequently cooled to -78 °C. A solution of the corresponding ketone (11.63 mmol) in THF (5 ml) was added by a syringe over a 2 min to the mixture. The solution was stirred for 1 h at -78 °C, then TMSCl (1.77 mL, 13.96 mmol) was added dropwise. The reaction mixture was subsequently allowed to warm up slowly to room temperature and it was stirred overnight. The reaction mixture was quenched with a saturated aqueous solution of $NaHCO_3$ (10 mL) and diluted with *n*-hexane. The layers were separated and the aqueous phase was extracted with hexane (2 x 20 mL). The combined organic layers were washed with H_2O (2 x 15 mL) and brine, dried over anhydrous Na_2SO_4, filtered, and concentrated to obtain the crude product. This was purified by column chromatography (6% H_2O deactivated 90 neutral aluminium oxide and hexane as eluent). The isomer ratio were determined by ^{1}H NMR spectroscopy of the crude products.

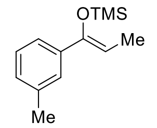

(*Z*)-trimethyl-(1-m-tolylprop-1-enyloxy)silane (**4c**)

Prepared as described above starting from 1-(3-methylphenyl)propan-1-one (1.12 g, 7.56 mmol) to afford the silyl enol ether (1.35 g, 6.12 mmol) as a colourless oil in 81% yield (>25:1 Z:E). **^{1}H NMR (200 MHz, $CDCl_3$)** δ 7.38 – 7.16 (m, 3H), 7.08 (d, *J* = 7.3 Hz, 1H), 5.36 (q, *J* = 6.8, 6.3 Hz, 1H), 2.38 (s, 3H), 1.77 (d, *J* = 6.8 Hz, 3H), 0.18 (s, 9H).

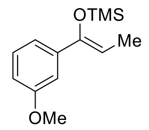

(*Z*)-(1-(3-methoxyphenyl)prop-1-enyloxy)trimethylsilane (**4e**)

Prepared as described above starting from 1-(3-methoxyphenyl)propan-1-one (1.90 g, 11.63 mmol) to afford the silyl enol ether (1.5 g, 6.35 mmol) as a colourless oil in 55% yield (>25:1 Z:E). **^{1}H NMR (300 MHz, $CDCl_3$)** δ 7.19 (t, *J* = 7.9 Hz, 1H), 7.07 – 6.98 (m, 2H), 6.77 (ddd, *J* = 8.2, 2.7, 1.1 Hz, 1H), 5.33 (q, *J* = 6.9 Hz, 1H), 3.80 (s, 3H), 1.73 (d, *J* = 6.9 Hz, 3H), 0.14 (d, *J* = 1.0 Hz, 9H).

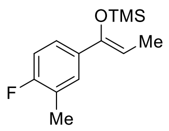

(*Z*)-1-(4-fluoro-3-methylphenyl)prop-1-en-1-yl)oxy)trimethylsilane (**4g**)

Prepared as described above starting from 1-(4-fluoro-3-methylphenyl)propan-1-one **7** (1.22 g, 7.34 mmol) to afford the silyl enol ether (1.12 g, 4.69mmol) as a colourless oil in 64% yield (>25:1 Z:E). **^{1}H NMR (300 MHz, $CDCl_3$)** δ 7.20 (d, *J* = 6.6 Hz, 2H), 6.90 (t, *J* = 8.9 Hz, 1H), 5.22 (q, *J* = 6.8 Hz, 1H), 2.25 (s, 3H), 1.70 (d, *J* = 6.8 Hz, 3H), 0.12 (s, 9H).

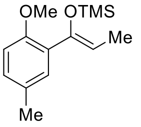

(*Z*)-1-(2-methoxy-5-methylphenyl)prop-1-en-1-yl)oxy)trimethylsilane (**4h**)

Prepared as described as above starting from 1-(2-methoxy-5-methylphenyl)propan-1-one **8** (0.89 g, 5.01 mmol) to afford the silyl enol ether (460 mg, 1.83 mmol) as a colourless oil in 37% yield (>25:1 Z:E). **^{1}H NMR (300 MHz, $CDCl_3$)** δ 7.13 – 6.98 (m, 2H), 6.78 – 6.67 (m, 1H), 5.13 (q, *J* = 6.7 Hz, 1H), 3.79 (s, 3H), 2.26 (s, 3H), 1.72 (d, *J* = 7.2 Hz, 3H), 0.24 (s, 9H).

(*Z*)-1-(4-methoxy-3,5-dimethylphenyl)prop-1-en-1-yl)oxy)trimethylsilane (**4i**)

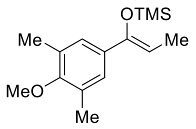

Prepared as described above starting from 1-(4-methoxy-3,5-dimethylphenyl)propan-1-one **9** (1.02 g, 5.33 mmol) to afford the silyl enol ether (1.01 g, 3.83 mmol) as a colourless oil in 72% yield (>25:1 Z:E). **^{1}H NMR (300 MHz, $CDCl_3$)** δ 7.09 (s, 2H), 5.22 (q, *J* = 6.9 Hz, 1H), 3.70 (s, 3H), 2.25 (s, 6H), 1.70 (d, *J* = 6.8 Hz, 3H), 0.13 (s, 9H).

(*Z*)-1-(4-methoxyphenyl)but-1-enyloxy)trimethylsilane (**4j**)

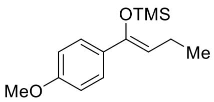

Prepared as described above starting from 1-(4-methoxy-phenyl)butan-1-one **10** (1.5 g, 8.41 mmol) to afford the silyl enol ether (1.36 g, 5.46 mmol) as a colourless oil in 65% yield (>25:1 Z:E). **^{1}H NMR (300 MHz, $CDCl_3$)** δ 7.41 (d, *J* = 8.8 Hz, 2H), 6.84 (d, *J* = 8.8 Hz, 2H), 5.13 (t, *J* = 7.1 Hz, 1H), 3.80 (s, 3H), 2.29 – 2.03 (m, 2H), 1.05 (t, *J* = 7.5 Hz, 3H), 0.15 (s, 9H).

(*Z*)-(1-(3,4-dimethoxyphenyl)-3-phenylprop-1-enyloxy)trimethylsilane (**4l**)

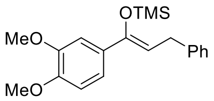

Prepared as described above starting from 1-(3,4-dimethoxyphenyl)butan-1-one **11** (1.5 g, 5.54 mmol) to afford the silyl enol ether (600 mg, 1.75 mmol) as a colourless oil in 32% yield (>25:1 Z:E). **^{1}H NMR (200 MHz, $CDCl_3$)** δ 7.31 (m, 6H), 7.08 (m, 2H), 5.37 (t, *J* = 7.1 Hz, 1H), 3.93 (s, 6H), 3.60 (d, *J* = 7.1 Hz, 2H), 0.22 (s, 9H).

Synthesis of ligands

(*R*)-(4,4,6,6'-tetramethoxybiphenyl-2,2'diyl)bis(bis(3,4,5-trimethoxyphenyl)phosphine)oxide (3x)

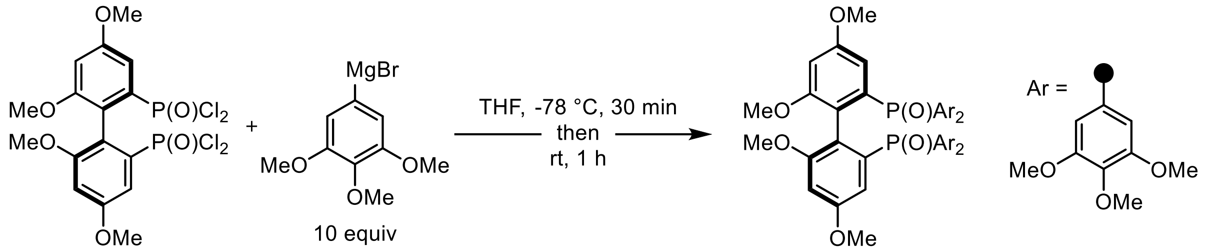

A solution of 3,4,5-Trimethoxyphenylmagnesium bromide (0.5 M in THF, 18 mmol) prepared using standard procedures from Mg turnings, one I_2 crystal, and 3,4,5-Trimethoxybromobenzene was added to a solution of (*R*)-4,4',6,6'-tetramethoxybiphenyl 2,2'-diyldiphosphonic dichloride previously prepared according to a known procedure[17] (1.8 mmol) in THF (20 ml) at -78°C. The mixture was stirred at -78°C for 30 min, then the dry ice/acetone bath was removed and the reaction was let thaw to room temperature and stirred for 1 h at this temperature. The reaction was then quenched by slow addition of aqueous sat. solution of $NHCl_4$ (30 ml). The organic layer was separated and the aqueous layer was extracted with ethyl acetate (2 x 40 ml). The combined organic layer was washed with brine and dried over Na_2SO_4. The solvent was removed and the residues were purified twice via silica gel chromatography (CH_2Cl_2:MeOH 97:3). The solvents were removed to give the product (948 mg, 0.92 mmol, 51% yield) as an off-white solid.
$[\alpha]^{22}_D$ = +44.4 (c = 2.0, $CHCl_3$). **^{1}H NMR (300 MHz, $CDCl_3$)** δ 6.93 (d, $^3J_{H\text{-}P}$ = 13.0 Hz, 4H), 6.90 (d, $^3J_{H\text{-}P}$ = 12.8 Hz, 4H), 6.44 (d, $^3J_{H\text{-}P}$ = 14.5 Hz, 2H), 6.39 (s, 2H), 3.87 (s, 6H), 3.82 (s, 6H), 3.79 (s, 12H), 3.66 (s, 6H), 3.64 (s, 12H), 3.34 (s, 6H). **^{13}C NMR (75 MHz, $CDCl_3$)** δ 160.4 (d, $^3J_{C\text{-}P}$ = 17.1 Hz), 160.3 (d, $^3J_{C\text{-}P}$ = 18.9 Hz), 154.4 (d, $^3J_{C\text{-}P}$ = 17.9 Hz), 154.3 (d, $^3J_{C\text{-}P}$ = 17.4 Hz), 142.1 (d, $^4J_{C\text{-}P}$ = 2.8 Hz), 142.0 (d, $^4J_{C\text{-}P}$ = 2.7 Hz), 133.5 (d, $^1J_{C\text{-}P}$ = 104.8 Hz), 131.2 (d, $^1J_{C\text{-}P}$ = 105.7 Hz), 129.8 (d, $^1J_{C\text{-}P}$ = 103.5 Hz), 124.7 (dd, $^3J_{C\text{-}P}$ = 7.4 Hz, $^2J_{C\text{-}P}$ = 4.3 Hz), 111.4 (d, $^2J_{C\text{-}P}$ = 11.7 Hz), 111.3 ($^2J_{C\text{-}P}$ =14.1 Hz), 111.2 (d, $^2J_{C\text{-}P}$ = 11.3 Hz), 101.9 (d, $^4J_{C\text{-}P}$ = 2.4 Hz), 62.4, 62.3, 57.9, 57.8, 56.7, 56.6. **^{31}P NMR (121 MHz, $CDCl_3$)** δ 28.53 (h, *J* = 13.1 Hz). **HRMS (ESI)** Calcd for $C_{52}H_{61}O_{18}P_2$ ($[M+H]^+$) 1035.3255, found 1035.3375.

(*S*)-(4,4,6,6'-tetramethoxybiphenyl-2,2'diyl)bis(bis(2-naphtyl)phosphine)oxide, (*S*)-2-Napht-GARPHOSO (*ent*-3z)

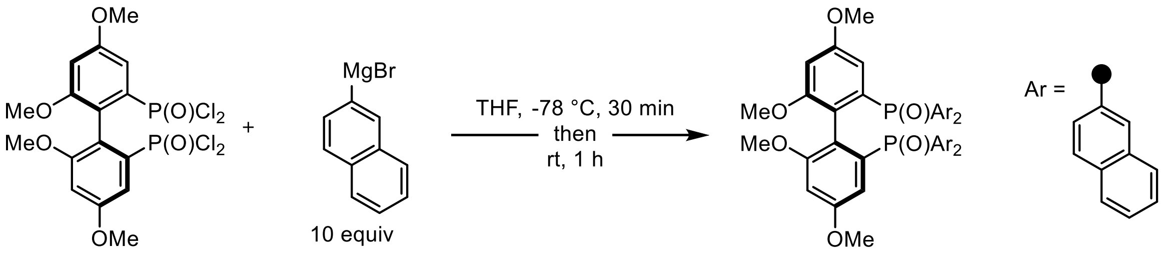

A solution of 2-Naphthylmagnesium bromide (0.5 M in THF, 18 mmol) prepared using standard procedures from Mg turnings, one I_2 crystal, and 2-naphthylbromide was added to a solution of (*S*)-4,4',6,6'-tetramethoxybiphenyl 2,2'-diyldiphosphonic dichloride previously prepared according to a known procedure[18] (1.8 mmol) in THF (20 ml) at -78°C. The mixture was stirred at -78°C for 30 min, then the dry ice/acetone bath was removed and the reaction was let thaw to room temperature and stirred for 1 h at this temperature. The reaction was then quenched by slow addition of aqueous sat. solution of $NHCl_4$ (30 ml). The organic layer was separated and the aqueous layer was extracted with ethyl acetate (2 x 40 ml). The combined organic layer was washed with brine and dried over Na_2SO_4. The solvent was removed and the residues were purified via silica gel chromatography (CH_2Cl_2:MeOH 95:5). The solvents were removed to give the product (1.2 g, 1.45 mmol, 81% yield) as a white solid.
$[\alpha]^{22}_D$ = -72.4 (c = 1.0, $CHCl_3$). **^{1}H NMR (300 MHz, $CDCl_3$)** δ 8.37 (d, $^3J_{H\text{-}P}$ = 14.0 Hz, 2H), 8.09 (d, $^3J_{H\text{-}P}$ = 14.0 Hz, 2H), 7.90 (dd, *J* = 10.1, 7.9 Hz, 7H), 7.78 (d, *J* = 9.8 Hz, 1H), 7.70 (d, *J* = 8.4 Hz, 2H), 7.63 – 7.53 (m, 6H), 7.52 – 7.43 (m, 5H), 7.40 – 7.32 (m, 3H), 6.56 (dd, $^3J_{H\text{-}P}$ = 14.9, 2.3 Hz, 2H), 6.07 (d, *J* = 2.4 Hz, 2H), 3.43 (s, 6H), 3.10 (s, 6H). **^{13}C NMR (75 MHz, $CDCl_3$)** δ 160.8 (d, $^3J_{C\text{-}P}$ = 18.8 Hz), 160.5 (d, $^3J_{C\text{-}P}$ = 17.2 Hz), 136.0 (d, $^4J_{C\text{-}P}$ = 2.5 Hz), 135.8 (d, $^4J_{C\text{-}P}$ = 2.5 Hz), 135.5 (d, *J* = 9.6), 135.4 (d, *J*=4.5 Hz), 133.9 (d, $^2J_{C\text{-}P}$ = 13.5 Hz), 133.7 (d, $^2J_{C\text{-}P}$ = 13.5 Hz), 133.6 (d, $^1J_{C\text{-}P}$ = 104.2 Hz), 130.5 (d, $^3J_{C\text{-}P}$ = 17.5 Hz), 129.4 ($^2J_{C\text{-}P}$ = 13.2 Hz), 129.2 ($^2J_{C\text{-}P}$ = 10.2 Hz), 129.1 ($^2J_{C\text{-}P}$ = 10.2 Hz), 128.9 (d, $^3J_{C\text{-}P}$ = 19.1 Hz), 128.8 (d, $^3J_{C\text{-}P}$ = 19.1 Hz),128.1 (d, $^3J_{C\text{-}P}$ = 18.2), 128.0 (d, $^3J_{C\text{-}P}$ = 18.2), 123.5 (dd, *J* = 8.0, 4.3 Hz), 111.9 (d, $^2J_{C\text{-}P}$ = 13.5 Hz), 101.7 (d, $^4J_{C\text{-}P}$ = 2.3 Hz), 56.5, 56.3. **^{31}P NMR (121 MHz, $CDCl_3$)** δ 31.01.

Characterization of the arylated ketones

General procedure B: α-Arylation of ketones

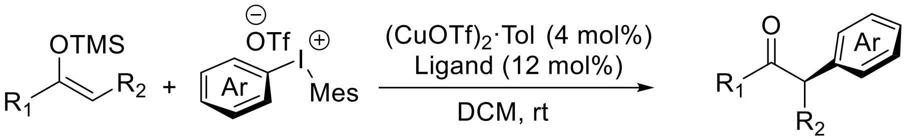

In a N_2-filled glove box, an oven dried vial was charged with $(CuOTf)_2$.Tol (4 mol%). The vial was sealed with a ptfe septum screw cap and was removed from the glove box. We observed that adding other solid reagents into the vial out of the glovebox exposing the Cu salt to the air, provided identical (sometimes slightly superior) results. Therefore, the phosphine oxide ligand **3x** (12 mol%), the appropriate mesitylaryliodonium triflate **5** (1.0 equiv.), and a magnetic stirrer were quickly added to the vial out of the glovebox for simplicity. The vial was then sealed and filled with nitrogen and then dichloromethane (0.2 M). After 1 min the appropriate silyl enol ether **4** (2 equiv.) was added via syringe and the reaction mixture stirred for 16 h at room temperature. The reaction was quenched by addition of diethyl ether (2 ml), filtered through a short pad of silica gel, and washed with diethyl ether (ca. 8 ml). A stock solution of the internal standard in DCM was added, and the mixture was concentrated under vacuum. NMR yields were determined on the crude product (internal standard: dimethyl terephthalate, 6H, 3.96 ppm and 4H, 8.1 ppm). Purification by column chromatography on silica gel using the solvent mixture reported below afforded the desired α-arylated ketone (see yields below). The enantiomeric purity was determined by chiral HPLC analysis on the purified product.

Synthesis of the racemic compounds for HPLC peaks attribution was prepared according to the same procedure using *rac*-SEGPHOSO **3n** instead of (*R*)-**3x** as ligand.

(2*S*)-1,2-diphenylpropan-1-one (6a)

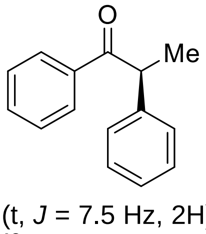

Prepared according to General Procedure B using $(CuOTf)_2$.Tol (4 mol%), MesPhIOTf **5a** (26.7 mg, 0.056 mmol, 1 equiv.), PO Ligand **3x** (12 mol%) and **4a** (23 mg, 0.112 mmol, 2 equiv.) in dichloromethane (0.3 mL) at room temperature for 16 hours; flash chromatography on silica gel (Hexane: dichloromethane 6:4) provided the title compound (7.3 mg, 0.034 mmol, 62% yield (68 % NMR yield), 94:6 er) as white solid (HPLC analysis of the product: Phenomenex cellulose-3, Hexane: 2-propanol 98:2, flow: 0.8 mL/min, T_R = 16.7 min (major) and T_R = 19.3 min (minor). $[\alpha]^{22}_D$ = +175 (c = 1.0, $CHCl_3$). **^{1}H NMR (300 MHz, $CDCl_3$)** δ 7.94 (d, *J* = 7.7 Hz, 2H), 7.47 (t, *J* = 7.3 Hz, 1H), 7.37 (t, *J* = 7.5 Hz, 2H), 7.27 (t, *J* = 5.6 Hz, 4H), 7.19 (p, *J* = 4.2 Hz, 1H), 4.68 (q, *J* = 6.9 Hz, 1H), 1.53 (d, *J* = 6.7 Hz, 3H). **^{13}C NMR (75 MHz, $CDCl_3$)** δ 200.5, 141.6, 136.7, 132.9, 129.1, 128.9, 128.6, 127.9, 127.0, 48.1, 19.6. **HRMS (ESI)** Calcd for $C_{15}H_{14}ONa$ ($[M+Na]^+$) 233.0936, found 233.0940. The spectral data are in agreement with literature data.[19]

(2*S*)-1-(4-methylphenyl)-2-phenylpropan-1-one (6b)

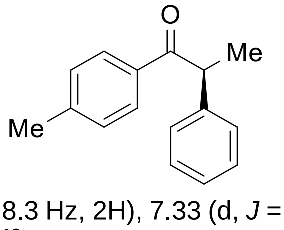

Prepared according to General Procedure B using $(CuOTf)_2$.Tol (4 mol%), MesPhIOTf **5a** (65 mg, 0.137 mmol, 1 equiv.), PO Ligand **3x** (12 mol%) and **4b** (60 mg, 0.274 mmol, 2 equiv.) in dichloromethane (0.7 mL) at room temperature for 16 hours; flash chromatography on silica gel (Hexane: dichloromethane 6:4) provided the title compound (25.8 mg, 0.115 mmol, 84% yield (89 % NMR yield), 95:5 er) as colourless oil (HPLC analysis of the product: Phenomenex cellulose-3, Hexane: 2-propanol 98:2, flow: 0.8 mL/min, 254 nm, T_R = 16.9 min (major) and T_R = 22.6 min (minor). $[\alpha]^{22}_D$ = +98.8 (c = 1.7, $CHCl_3$). **^{1}H NMR (200 MHz, $CDCl_3$)** δ 7.90 (d, *J* = 8.3 Hz, 2H), 7.33 (d, *J* = 4.4 Hz, 4H), 7.28 – 7.14 (m, 3H), 4.71 (q, *J* = 6.9 Hz, 1H), 2.39 (s, 3H), 1.57 (d, *J* = 6.9 Hz, 3H). **^{13}C NMR (50 MHz, $CDCl_3$)** δ 200.1, 143.7, 141.9, 134.1, 129.3, 129.0, 127.9, 126.9, 47.9, 21.7, 19.6. **HRMS (ESI)** Calcd for $C_{16}H_{16}ONa$ ($[M+Na]^+$) 247.1099, found 247.1105. The spectral data are in agreement with literature data.[20]

(2*S*)-1-(3-methylphenyl)-2-phenylpropan-1-one (6c)

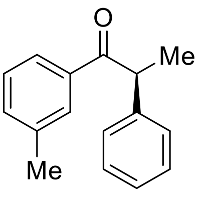

Prepared according to General Procedure B using $(CuOTf)_2$.Tol (4 mol%), MesPhIOTf **5a** (66 mg, 0.139 mmol, 1 equiv.), PO Ligand **3x** (12 mol%) and **4c** (92 mg, 0.417 mmol, 3 equiv.) in dichloromethane (0.7 mL) at room temperature for 16 hours; flash chromatography on silica gel (Hexane: dichloromethane 6:4) provided the title compound (15.9 mg, 0.07 mmol, 51% yield (56 % NMR yield), 96:4 er) as colourless oil (HPLC analysis of the product: Phenomenex cellulose-3, Hexane: 2-propanol 98:2, flow: 0.8 mL/min, 254 nm, T_R = 14.3 min (major) and T_R = 18.3 min (minor). $[\alpha]^{22}_D$ = +104.63 (c = 0.8, $CHCl_3$). **^{1}H NMR (200 MHz, $CDCl_3$)** δ 7.75 (d, *J* = 10.0 Hz, 2H), 7.26 (m, 7H), 4.69 (q, *J* = 6.9 Hz, 1H), 2.35 (s, 3H), 1.53 (d, *J* = 6.9 Hz, 3H). **^{13}C NMR (50 MHz, $CDCl_3$)** δ 200.7, 141.7, 138.4,

136.7, 133.7, 129.4, 129.0, 128.4, 127.9, 127.0, 126.1, 48.0, 21.5, 19.6. **HRMS (ESI)** Calcd for $C_{16}H_{16}ONa$ ($[M+H]^+$) 225.1279, found 225.1263.

(2*S*)-1-(4-methoxyphenyl)-2-phenylpropan-1-one (6d)

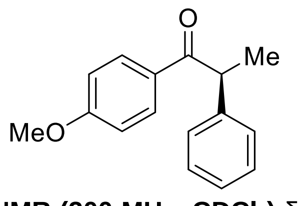

Prepared according to General Procedure B using $(CuOTf)_2$.Tol (4 mol%), MesPhIOTf **5a** (54 mg, 0.114 mmol, 1 equiv.), PO Ligand **3x** (12 mol%) and **4d** (54 mg, 0.228 mmol, 2 equiv.) in dichloromethane (0.57 mL) at room temperature for 16 hours; flash chromatography on silica gel (Hexane: dichloromethane 4:6) provided the title compound (22.4 mg, 0.093 mmol, 82% yield (90 % NMR yield), 97:3 er) as white solid (HPLC analysis of the product: Phenomenex cellulose-3, Hexane: 2-propanol 98:2, flow: 0.8 mL/min, 254 nm, T_R = 32.3 min (major) and T_R = 37.0 min (minor). $[\alpha]^{22}_{D}$ = +67.16 (c = 2.2, $CHCl_3$). **^{1}H NMR (200 MHz, $CDCl_3$)** δ 7.99 (d, *J* = 8.9 Hz, 2H), 7.34 – 7.19 (m, 5H), 6.90 (d, *J* = 8.9 Hz, 2H), 4.69 (q, *J* = 6.8 Hz, 1H), 3.85 (s, 3H), 1.56 (d, *J* = 6.9 Hz, 3H). **^{13}C NMR (50 MHz, $CDCl_3$)** δ 199.1, 163.4, 142.2, 131.3, 129.7, 129.1, 127.9, 127.0, 113.9, 55.6, 47.7, 19.8. **HRMS (ESI)** Calcd for $C_{16}H_{16}O_2$ ($[M+H]^+$) 241.1229, found 241.1227. The spectral data are in agreement with literature data.[21]

(2*S*)-1-(3-methoxyphenyl)-2-phenylpropan-1-one (6e)

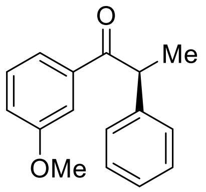

Prepared according to General Procedure B using $(CuOTf)_2$.Tol (4 mol%), MesPhIOTf **5a** (45 mg, 0.095 mmol, 1 equiv.), PO Ligand **3x** (12 mol%) and **4e** (46 mg, 0.195 mmol, 2 equiv.) in dichloromethane (0.47 mL) at room temperature for 16 hours; flash chromatography on silica gel (Hexane: dichloromethane 4:6) provided the title compound (11 mg, 0.045 mmol, 48% yield (53 % NMR yield), 96:4 er) as colourless oil (HPLC analysis of the product: Phenomenex cellulose-3, Hexane: 2-propanol 98:2, flow: 0.8 mL/min, 254 nm, T_R = 23.0 min (major) and T_R = 32.0 min (minor). $[\alpha]^{22}_{D}$ = +109.8 (c = 1.0, $CHCl_3$). **^{1}H NMR (300 MHz, $CDCl_3$)** δ 7.51 (d, *J* = 7.7 Hz, 1H), 7.46 (t, *J* = 2.1 Hz, 1H), 7.33 – 7.25 (m, 4H), 7.23 (d, *J* = 2.7 Hz, 1H), 7.17 (dt, *J* = 8.6, 4.0 Hz, 1H), 7.00 (ddd, *J* = 8.2, 2.7, 1.0 Hz, 1H), 4.64 (q, *J* = 6.9 Hz, 1H), 3.77 (s, 3H), 1.51 (d, *J* = 6.9 Hz, 3H). **^{13}C NMR (75 MHz, $CDCl_3$)** δ 200.3, 159.9, 141.6, 138.0, 129.6, 129.1, 127.9, 127.0, 121.5, 119.4, 113.3, 55.5, 48.2, 19.6. **HRMS (ESI)** Calcd for $C_{16}H_{16}O_2$ ($[M+H]^+$) 241.1229, found 241.1227.

(2*R*)-1-(4-chlorophenyl)-2-phenylpropan-1-one (*ent*-6f)

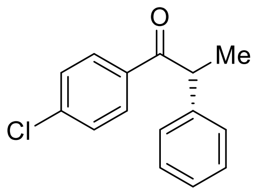

Prepared according to General Procedure B using $(CuOTf)_2$.Tol (4 mol%), MesPhIOTf **5a** (68 mg, 0.144 mmol, 1 equiv.), PO Ligand *ent*-**3z** (12 mol%) and **4f** (104 mg, 0.432 mmol, 3 equiv.) in dichloromethane (0.72 mL) at room temperature; flash chromatography on silica gel (Hexane: dichloromethane 6:4) provided the title compound (18 mg, 0.072 mmol, 50% yield, 91:9 er) as colourless oil (HPLC analysis of the product: Phenomenex cellulose-3, Hexane: 2-propanol 98:2, flow: 0.8 mL/min, 240 nm, T_R = 10.6 min (minor) and T_R = 13.3 min (major). $[\alpha]^{22}_{D}$ = +28.0 (c = 0.7, $CHCl_3$). **^{1}H NMR (200 MHz, $CDCl_3$)** δ 7.92 (d, *J* = 8.6 Hz, 2H), 7.38 (m, 3H), 7.33 – 7.09 (m, 4H), 4.65 (q, *J* = 6.8 Hz, 1H), 1.57 (d, *J* = 6.8 Hz, 3H). **^{13}C NMR (50 MHz, $CDCl_3$)** δ 199.3, 141.4, 139.4, 135.0, 130.4, 129.3, 129.0, 127.9, 127.3, 48.3, 19.6. **HRMS (ESI)** Calcd for $C_{15}H_{14}ClO$ ($[M+H]^+$) 245.0733, found 245.0744. The spectral data are in agreement with literature data.[22]

(2*R*)-2-phenyl-1-[4-(trifluoromethyl)phenyl]propan-1-one (*ent*-6g)

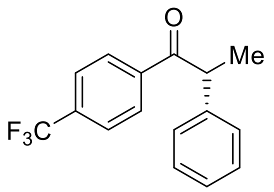

Prepared according to General Procedure B using $(CuOTf)_2$.Tol (4 mol%), MesPhIOTf **5a** (80.2 mg, 0.17 mmol, 1 equiv.), PO Ligand *ent*-**3z** (12 mol%) and **4g** (140 mg, 0.51 mmol, 3 equiv.) in dichloromethane (0.85 mL) at room temperature for 24 hours; flash chromatography on silica gel (hexane: dichloromethane 8:2) provided the title compound (19 mg, 0.068 mmol, 40 % yield (40% NMR yield), 92:8 *er*) as colourless oil (HPLC analysis of the product: Phenomenex cellulose-3, Hexane: 2-propanol 98:2, flow: 0.8 mL/min, 240 nm, T_R = 8.1 min (minor) and T_R = 9.5 min (major). **^{1}H NMR (300 MHz, $CDCl_3$)** δ 8.02 (d, *J* = 8.1 Hz, 2H), 7.63 (d, *J* = 8.2 Hz, 2H), 7.34 – 7.27 (m, 1H), 7.26 – 7.15 (m, 2H), 4.65 (q, *J* = 6.8 Hz, 1H), 1.55 (d, *J* = 6.9 Hz, 3H). **^{13}C NMR (75 MHz, $CDCl_3$)** δ 199.4, 140.9, 139.4, 134.2 (q, $^2J_{C-F}$ = 32.4 Hz), 129.4, 129.2, 127.9, 127.4, 125.7 (q, $^3J_{C-F}$ = 3.8 Hz), 123.8 (q, *J* = 272.7 Hz), 48.7, 19.5. **^{19}F NMR (188 MHz, $CDCl_3$)** δ –63.6. The spectral data are in agreement with literature data.[23]

(2*S*)-1-(4-fluoro-3-methylphenyl)-2-phenylpropan-1-one (6h)

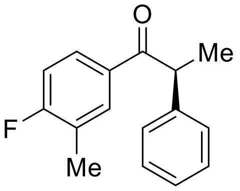

Prepared according to General Procedure B using $(CuOTf)_2$.Tol (4 mol%), MesPhIOTf **5a** (47 mg, 0.1 mmol, 1 equiv.), PO Ligand **3x** (12 mol%) and **4g** (48 mg, 0.2 mmol, 2 equiv.) in dichloromethane (0.5 mL) at room temperature for 16 hours; flash chromatography on silica gel (hexane: dichloromethane 6:4) provided the title compound (12.8 mg, 0.052 mmol, 52% yield (57% NMR yield), 96.5:3.5 *er*) as colourless oil (HPLC analysis of the product: Phenomenex cellulose-3, Hexane: 2-propanol 98:2, flow: 0.8 mL/min, 240 nm, T_R = 11.9 min (major) and T_R = 17.3 min (minor). $[\alpha]^{22}_D$ = 101.2 (c = 0.98, $CHCl_3$). **^{1}H NMR (200 MHz, $CDCl_3$)** δ 8.02 – 7.66 (m, 2H), 7.27 (d, *J* = 12.2 Hz, 5H), 7.01 (t, *J* = 8.8 Hz, 1H), 4.68 (q, *J* = 6.8 Hz, 1H), 2.30 (s, 3H), 1.57 (d, *J* = 6.8 Hz, 3H). **^{13}C NMR (50 MHz, $CDCl_3$)** δ 199.3, 164.3 (d, $^1J_{C-F}$ = 253.5 Hz), 141.7, 132.8 (d, $^3J_{C-F}$ = 6.5 Hz), 129.2, 128.9 (d, $^3J_{C-F}$ = 9.4 Hz), 127.9, 127.2, 125.5 (d, $^2J_{C-F}$ = 17.9 Hz), 115.3 (d, $^2J_{C-F}$ = 23.0 Hz), 48.0, 19.7, 14.8. **^{19}F NMR (188 MHz, $CDCl_3$)** δ -110.1. **HRMS (ESI)** Calcd for $C_{16}H_{16}FO$ ($[M+H]^+$) 243.1185, found 243.1164.

(2*S*)-1-(2-methoxy-5-methylphenyl)-2-phenylpropan-1-one (6i)

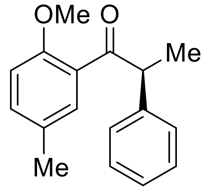

Prepared according to General Procedure B using $(CuOTf)_2$.Tol (4 mol%), MesPhIOTf **5a** (60 mg, 0.127 mmol, 1 equiv.), PO Ligand **3x** (12 mol%) and **4h** (64 mg, 0.254 mmol, 2 equiv.) in dichloromethane (0.64 mL) at room temperature for 16 hours; flash chromatography on silica gel (hexane: dichloromethane 6:4) provided the title compound (22 mg, 0.086 mmol, 68% yield (60% NMR yield), 83:17 *er*) as colourless oil (HPLC analysis of the product: Phenomenex cellulose-3, Hexane: 2-propanol 98:2, flow: 0.8 mL/min, 240 nm, T_R = 17.9 min (minor) and T_R = 24.5 min (major). $[\alpha]^{22}_D$ = 6.7 (c = 2.2, $CHCl_3$).**^{1}H NMR (300 MHz, $CDCl_3$)** δ 7.33 – 7.23 (m, 5H), 7.19 (td, *J* = 8.5, 7.5, 2.3 Hz, 2H), 6.79 (d, *J* = 8.4 Hz, 1H), 4.77 (q, *J* = 7.0 Hz, 1H), 3.84 (s, 3H), 2.26 (s, 3H), 1.53 (d, *J* = 7.0 Hz, 3H). **^{13}C NMR (75 MHz, $CDCl_3$)** δ 204.4, 155.8, 141.6, 133.4, 130.9, 130.0, 128.8, 128.5, 128.3, 126.7, 111.5, 55.6, 51.8, 20.4, 19.0. **HRMS (ESI)** Calcd for $C_{17}H_{19}O_2$ ($[M+H]^+$) 255.1385, found 255.1727.

(2*S*)-1-(4-methoxy-3,5-dimethylphenyl)-2-phenylpropan-1-one (6j)

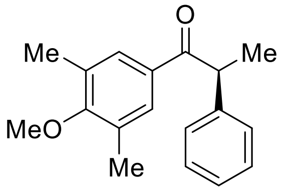

Prepared according to General Procedure B using $(CuOTf)_2$.Tol (4 mol%), MesPhIOTf **5a** (87 mg, 0.184 mmol, 1 equiv.), PO Ligand **3x** (12 mol%) and **4i** (97 mg, 0.368 mmol, 2 equiv.) in dichloromethane (0.9 mL) at room temperature; flash chromatography on silica gel (hexane: dichloromethane 4:6) provided the title compound (30 mg, 0.111 mmol, 61% yield (71% NMR yield), 96:4 *er*) as colourless oil (HPLC analysis of the product: Phenomenex cellulose-3, Hexane: 2-propanol 98:2, flow: 0.8 mL/min, 240 nm, T_R = 14.6 min (major) and T_R = 18.4 min (minor). $[\alpha]^{22}_D$ = 52.0 (c = 2.7, $CHCl_3$). **^{1}H NMR (200 MHz, $CDCl_3$)** δ 7.64 (s, 2H), 7.35 – 7.24 (m, 4H), 7.22 – 7.10 (m, 1H), 4.66 (q, *J* = 6.9 Hz, 1H), 3.71 (s, 3H), 2.26 (s, 6H), 1.51 (d, *J* = 6.8 Hz, 3H). **^{13}C NMR (50 MHz, $CDCl_3$)** δ 199.9, 161.1, 141.9, 132.3, 131.2, 130.0, 129.0, 127.9, 126.9, 59.7, 47.6, 19.7, 16.4. **HRMS (ESI)** Calcd for $C_{18}H_{21}O_2$ ($[M+H]^+$) 269.1542, found 269.1540.

(2*S*)-1-(4-methoxyphenyl)-2-phenylbutan-1-one (6k)

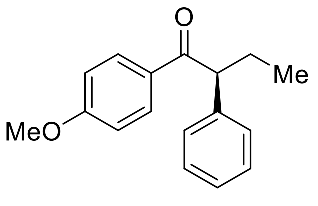

Prepared according to General Procedure B using $(CuOTf)_2$.Tol (4 mol%), MesPhIOTf **5a** (50 mg, 0.106 mmol, 1 equiv.), PO Ligand **3x** (12 mol%) and **4j** (53 mg, 0.212 mmol, 2 equiv.) in dichloromethane (0.53 mL) at room temperature for 16 hours; flash chromatography on silica gel (hexane: dichloromethane 6:4) provided the title compound (25.5 mg, 0.100 mmol, 95% yield (89% NMR yield), 95.5:4.5 *er*) as white solid (HPLC analysis of the product: Daicel AS-H, Hexane: 2-propanol 99:1, flow: 1.0 mL/min, 245 nm, T_R = 9.3 min (major) and T_R = 10.5 min (minor). $[\alpha]^{22}_D$ = 45.94 (c = 2.5, $CHCl_3$). **^{1}H NMR (200 MHz, $CDCl_3$)** δ 8.01 (d, *J* = 8.9 Hz, 2H), 7.46 – 7.09 (m, 5H), 6.90 (d, *J* = 8.9 Hz, 2H), 4.44 (t, *J* = 7.3 Hz, 1H), 3.85 (s, 3H), 2.22 (dq, *J* = 14.5, 7.3 Hz, 1H), 1.89 (dp, *J* = 14.6, 7.4 Hz, 1H), 0.94 (t, *J* = 7.4 Hz, 3H). **^{13}C NMR (50 MHz, $CDCl_3$)** δ 198.7, 163.4, 140.2, 131.1, 130.2, 128.9, 128.8, 128.8, 128.5, 128.5, 128.4, 128.3, 128.2, 127.0, 113.8, 113.7, 55.5, 55.2, 27.3, 12.5, 12.4. **HRMS (ESI)** Calcd for $C_{17}H_{19}O_2$ ($[M+H]^+$) 255.1385, found 255.1400.

(2S)-2-(4-methylphenyl)-1-phenylbutan-1-one (6l).

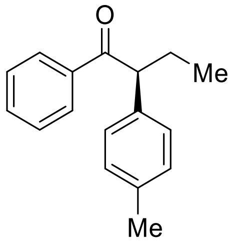

Prepared according to General Procedure B using $(CuOTf)_2$.Tol (4 mol%), MesTolIOTf **5b** (31 mg, 0.064 mmol, 1 equiv.), PO Ligand **3x** (12 mol%) and **4k** (26 mg, 0.127 mmol, 2 equiv.) in dichloromethane (0.32 mL) at room temperature for 16 hours; flash chromatography on silica gel (hexane: dichloromethane 6:4) provided the title compound (6 mg, 0.025 mmol, 39% yield (43% NMR yield), 97.5:2.5 *er*) as colourless oil (HPLC analysis of the product: Phenomenex cellulose-3, Hexane: 2-propanol 99.5:0.5, flow: 0.8 mL/min, 254 nm, T_R = 13.3 min (minor) and T_R = 18.4 min (major). $[\alpha]^{22}_D$ = 104.62 (c = 0.6, $CHCl_3$). **^{1}H NMR (200 MHz, $CDCl_3$)** δ 8.00 (dt, *J* = 6.8, 1.6 Hz, 2H), 7.44 (m, 3H), 7.23 (d, *J* = 8.2 Hz, 2H), 7.13 (d, *J* = 7.8 Hz, 2H), 4.45 (t, *J* = 7.3 Hz, 1H), 2.32 (s, 3H), 2.19 (dt, *J* = 14.6, 6.9 Hz, 1H), 1.87 (dp, *J* = 14.6, 7.4 Hz, 1H), 0.94 (t, *J* = 7.4 Hz, 3H). **^{13}C NMR (50 MHz, $CDCl_3$)** δ 200.4, 137.3, 136.8, 136.7, 132.8, 129.7, 128.8, 128.7, 128.6, 128.3, 55.2, 27.2, 21.2, 12.4. **HRMS (ESI)** Calcd for $C_{17}H_{18}ONa$ ($[M+Na]^+$) 261.1265, found 261.1265.

(2S)-1-(3,4-dimethoxyphenyl)-2,3-diphenylpropan-1-one (6m)

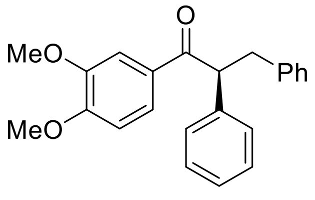

Prepared according to General Procedure B using $(CuOTf)_2$.Tol (4 mol%), MesPhIOTf **5a** (63 mg, 0.132 mmol, 1 equiv.), PO Ligand **3x** (12 mol%) and **4l** (90 mg, 0.264 mmol, 2 equiv.) in dichloromethane (0.6 mL) at room temperature for 16 hours; flash chromatography on silica gel (hexane: dichloromethane 4:6) provided the title compound (13 mg, 0.037 mmol, 29% yield (36% NMR yield), 85:15 *er*) as colourless oil (HPLC analysis of the product: Phenomenex cellulose-1, Hexane: 2-propanol 98:2, flow: 0.8 mL/min, 254 nm, T_R = 32.8 min (minor) and T_R = 37.3 min (major). $[\alpha]^{22}_D$ = 62.2 (c = 1.1, $CHCl_3$). **^{1}H NMR (200 MHz, $CDCl_3$)** δ 7.71 – 7.47 (m, 2H), 7.35 – 7.02 (m, 10H), 6.80 (d, *J* = 8.3 Hz, 1H), 4.83 (t, *J* = 7.2 Hz, 1H), 3.90 (d, *J* = 1.7 Hz, 6H), 3.61 (dd, *J* = 13.7, 7.6 Hz, 1H), 3.11 (dd, *J* = 13.7, 6.9 Hz, 1H). **^{13}C NMR (75 MHz, $CDCl_3$)** δ 197.9, 153.2, 149.1, 140.1, 139.8, 130.0, 129.2, 129.0, 128.3, 128.3, 127.2, 126.2, 123.5, 111.1, 110.1, 56.1, 56.0, 55.6, 40.3. **HRMS (ESI)** Calcd for $C_{23}H_{23}O_3$ ($[M+H]^+$) 347.1647, found 347.1634.

Methyl 4-[(2S)-1-(4-methylphenyl)-1-oxopropan-2-yl]benzoate (6o)

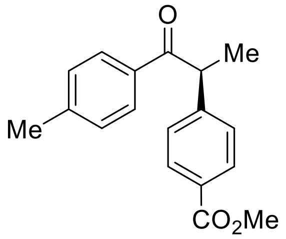

Prepared according to General Procedure B using $(CuOTf)_2$.Tol (4 mol%), diaryliodonium salt **5c** (40 mg, 0.07 mmol, 1 equiv.), PO Ligand 3x (12 mol%) and **4b** (33.4 mg, 0.151 mmol, 2 equiv.) in dichloromethane (0.4 mL) at room temperature for 16 hours; flash chromatography on silica gel (hexane: dichloromethane 6:4) provided the title compound (11.7 mg, 0.041 mmol, 55% yield (63% NMR yield), 95:5 *er*) as white solid (HPLC analysis of the product: Phenomenex cellulose-3, Hexane: 2-propanol 98:2, flow: 1 mL/min, 254 nm, T_R = 23.8 min (minor) and T_R = 26.8 min (major). $[\alpha]^{22}_D$ = 24.2 (c = 1.0, $CHCl_3$). **^{1}H NMR (300 MHz, $CDCl_3$)** δ 7.96 (d, *J* = 8.0 Hz, 2H), 7.83 (d, *J* = 7.9 Hz, 2H), 7.35 (d, *J* = 7.9 Hz, 2H), 7.18 (d, *J* = 7.9 Hz, 2H), 4.72 (q, *J* = 6.9 Hz, 1H), 3.88 (s, 3H), 2.35 (s, 3H), 1.54 (d, *J* = 6.9 Hz, 3H). **^{13}C NMR (75 MHz, $CDCl_3$)** δ 199.4, 167.0, 147.0, 144.0, 133.9, 130.4, 129.4, 129.0, 128.9, 128.0, 52.2, 47.8, 21.7, 19.4. **HRMS (ESI)** Calcd for $C_{18}H_{19}O_3$ ($[M+H]^+$) 283.1334, found 283.1333.

(2S)-2-(4-bromophenyl)-1-(4-methylphenyl)propan-1-one (6p)

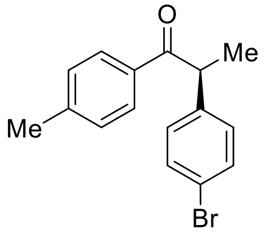

Prepared according to General Procedure B using $(CuOTf)_2$.Tol (4 mol%), diaryliodonium salt **5d** (21 mg, 0.047 mmol, 1 equiv.), PO Ligand **3x** (12 mol%) and **4b** (20.7 mg, 0.097mmol, 2 equiv.) in dichloromethane (0.2 mL) at room temperature for 16 hours; flash chromatography on silica gel (hexane: dichloromethane 6:4) provided the title compound (6.4 mg, 0.021 mmol, 45% yield (50% NMR yield), 94:6 *er*) as white solid (HPLC analysis of the product: Phenomenex cellulose-3, Hexane: 2-propanol 98:2, flow: 0.8 mL/min, 254 nm, T_R = 12.4 min (minor) and T_R = 16.5 min (major). $[\alpha]^{22}_D$ = 45.1 (c = 0.5, $CHCl_3$). **^{1}H NMR (300 MHz, $CDCl_3$)** δ 7.83 (d, *J* = 7.9 Hz, 2H), 7.41 (d, *J* = 8.1 Hz, 2H), 7.17 (t, *J* = 8.8 Hz, 4H), 4.63 (q, *J* = 6.9 Hz, 1H), 2.36 (s, 3H), 1.50 (d, *J* = 6.9 Hz, 3H). **^{13}C NMR (75 MHz, $CDCl_3$)** δ 199.6, 144.0, 140.8, 133.8, 132.2, 129.6, 129.4, 129.0, 120.9, 47.2, 21.7, 19.5. **HRMS (ESI)** Calcd for $C_{16}H_{16}BrO$ ($[M+H]^+$) 303.0385, found 303.0376.

(2S)-2-(4-fluorophenyl)-1-(4-methylphenyl)propan-1-one (6q)

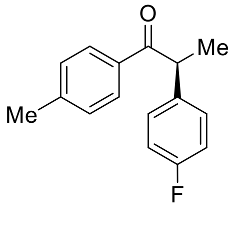

Prepared according to General Procedure B using $(CuOTf)_2$.Tol (4 mol%), diaryliodonium salt **5e** (52 mg, 0.106 mmol, 1 equiv.), PO Ligand **3x** (12 mol%) and **4b** (47 mg, 0.213 mmol, 2 equiv.) in dichloromethane (0.5 mL) at room temperature for 16 hours; flash chromatography on silica gel (hexane: dichloromethane 6:4) provided the title compound (8 mg, mmol, 32% yield (37% NMR yield), 95.5:4.5 *er*) as colourless oil (HPLC analysis of the product: Daicel AS-H, Hexane: 2-propanol 99:1, flow: 1 mL/min, 254 nm, T_R = 8.1 min (major) and T_R = 12.8 min (minor). $[\alpha]^{22}_D$ =

57.9 (c = 0.8, $CHCl_3$). **^{1}H NMR (300 MHz, $CDCl_3$)** δ 7.88 (d, J = 8.0 Hz, 2H), 7.29 (dd, J = 5.7, 2.8 Hz, 2H), 7.23 (d, J = 8.0 Hz, 2H), 7.01 (t, J = 8.6 Hz, 2H), 4.70 (q, J = 6.9 Hz, 1H), 2.40 (s, 3H), 1.54 (d, J = 7.1 Hz, 3H). **^{13}C NMR (50 MHz, $CDCl_3$)** δ 200.0, 164.3, 159.4, 143.9, 137.5, 133.9, 129.5, 129.4, 129.3, 129.0, 116.1, 116.0, 115.7, 115.6, 46.9, 21.7, 19.7. **^{19}F NMR** (188 MHz, $CDCl_3$) δ -116.4. **HRMS (ESI)** Calcd for $C_{16}H_{16}FO$ ($[M+H]^+$) 243.1185, found 243.1169.

(2S)-1-(4-methylphenyl)-2-[3-(trifluoromethyl)phenyl]propan-1-one (6r)

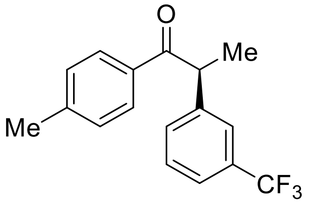

Prepared according to General Procedure B using $(CuOTf)_2$.Tol (4 mol%), diaryliodonium salt **5f** (50 mg, 0.093 mmol, 1 equiv.), PO Ligand **3x** (12 mol%) and **4b** (62 mg, 0.28 mmol, 3 equiv.) in dichloromethane (0.46 mL) at room temperature for 16 hours; flash chromatography on silica gel (hexane: dichloromethane 6:4) provided the title compound (10 mg, 0.034 mmol, 36% yield (30 % NMR yield), as colourless oil.**^{1}H NMR (200 MHz, $CDCl_3$)** δ 7.86 (d, J = 8.2 Hz, 2H), 7.62 – 7.33 (m, 4H), 7.21 (d, J = 8.0 Hz, 2H), 4.76 (q, J = 6.9 Hz, 1H), 2.37 (s, 3H), 1.55 (d, J = 6.9 Hz, 3H). **^{13}C NMR (75 MHz, $CDCl_3$)** δ 199.5, 144.2, 142.6, 133.7, 131.3, 129.5, 129.0, 126.9, 124.77 (q, $^3J_{C-F}$ = 3.3 Hz), 123.94 (q, $^3J_{C-F}$ = 3.5 Hz), 47.3, 21.7, 19.7. **^{19}F NMR** (188 MHz, $CDCl_3$) δ -62.9. **HRMS (ESI)** Calcd for $C_{17}H_{16}F_3O$ ($[M+H]^+$) 293.1153, found 293.1147.

(1*S*,2*S*)-1-(4-methylphenyl)-2-[3-(trifluoromethyl)phenyl]propan-1-ol (12).

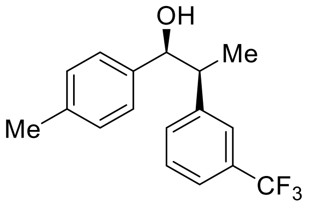

In accordance to a reported procedure,[21] a solution of lithium aluminium hydride (1.0 M in hexanes; 40 µL, 0.040 mmol) was slowly added to a −78 °C solution of (*S*)-**6r** (10 mg, 0.034 mmol) in THF (0.2 mL) in a 10-mL RBF under nitrogen. The reaction mixture was allowed to warm to 0 °C and then it was stirred at 0 °C for 2 h. Next, water (0.5 mL) was added dropwise to the reaction mixture at 0 °C to quench the reaction. Aqueous HCl (1.0 N; 0.5 mL) was added, and the mixture was extracted with EtOAc (3x5 mL). The combined organic extracts were dried over $NaSO_4$, filtered, and concentrated. The residue was purified by flash chromatography on silica gel, which afforded the alcohol as colourless oil (9.8 mg, 97% yield; >20:1 dr, 93.5:6.5 *er* (HPLC analysis of the product: Phenomenex cellulose-3, Hexane: 2-propanol 98:2, flow: 0.8 mL/min, 254 nm, T_R = 20.0 min (major) and T_R = 25.5 min (minor). $[\alpha]^{22}_D$ = –18.7 (c = 0.9, $CHCl_3$). **^{1}H NMR (200 MHz, $CDCl_3$)** δ 7.53 (d, J = 10.1 Hz, 3H), 7.33 – 7.03 (m, 4H), 4.71 (d, J = 8.4 Hz, 1H), 3.14 (p, J = 7.4 Hz, 1H), 2.40 (s, 3H), 1.77 (br, 1H), 1.15 (d, J = 7.1 Hz, 3H). **^{13}C NMR (50 MHz, $CDCl_3$)** δ 144.9, 139.6, 137.9, 131.7, 129.2, 128.9, 126.8, 124.95 (q, J = 3.9 Hz), 123.67 (q, J = 3.9 Hz), 79.3, 47.8, 21.3, 18.5. **^{19}F NMR** (188 MHz, $CDCl_3$) δ -62.9. **HRMS (ESI)** Calcd for $C_{17}H_{18}F_3O$ ($[M+H]^+$) 295.1310, found. The relative stereochemistry was determined by comparison of the ^{1}H NMR data of similar compound with reported values.[24]

(2S)-2-(3-methoxyphenyl)-1-(4-methylphenyl)propan-1-one (6s).

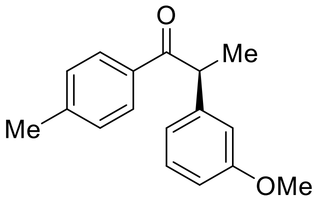

Prepared according to General Procedure B using $(CuOTf)_2$.Tol (4 mol%), diaryliodonium salt **5g** (45 mg, 0.089 mmol, 1 equiv.), PO Ligand **3x** (12 mol%) and **4b** (60 mg, 0.269 mmol, 3 equiv.) in dichloromethane (0.44 mL) at room temperature for 16 hours; flash chromatography on silica gel (hexane: dichloromethane 6:4) provided the title compound (17 mg, 0.066 mmol, 75% yield (77 % NMR), 94:6 *er*) as colourless oil (HPLC analysis of the product: Phenomenex cellulose-3, Hexane: 2-propanol 99:1, flow: 0.5 mL/min, 254 nm, T_R = 45.1 min (major) and T_R = 49.0 min (minor). $[\alpha]^{22}_D$ = 67.4 (c = 1.6, $CHCl_3$).**^{1}H NMR (200 MHz, $CDCl_3$)** δ 7.90 (d, J = 8.3 Hz, 2H), 7.32-7.12 (m, 3H), 6.95 – 6.82 (m, 2H), 6.77 (ddd, J = 8.2, 2.6, 1.0 Hz, 1H), 4.67 (q, J = 6.8 Hz, 1H), 3.80 (s, 3H), 2.39 (s, 3H), 1.56 (d, J = 6.8 Hz, 3H). **^{13}C NMR (50 MHz, $CDCl_3$)** δ 199.9, 160.1, 143.7, 143.4, 134.1, 130.0, 129.3, 129.0, 120.3, 113.6, 112.2, 112.2, 55.3, 47.9, 21.7, 19.5. **HRMS (ESI)** Calcd for $C_{17}H_{19}O_2$ ($[M+H]^+$) 255.1385, found 255.1400.

(2S)-2-(4-methoxyphenyl)-1-(4-methylphenyl)propan-1-one (6t)

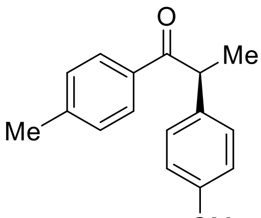

Prepared according to General Procedure B using $(CuOTf)_2$.Tol (4 mol%), diaryliodonium salt **5h** (96 mg, 0.191 mmol, 1 equiv.), PO Ligand **3x** (12 mol%) and **4b** (84 mg, 0.382 mmol, 2 equiv.) in dichloromethane (0.95 mL) at room temperature for 16 hours; flash chromatography on silica gel (hexane: dichloromethane 4:6) provided the title compound (30 mg, 0.118 mmol, 62% yield (65% NMR yield), 95:5 *er*) as colourless oil (HPLC analysis of the product: Phenomenex cellulose-3, Hexane: 2-propanol 98:2, flow: 1 mL/min, 254 nm, T_R = 21.4 min (major) and T_R = 27.0 min (minor). $[\alpha]^{22}_D$ = 68.3 (c = 2.8, $CHCl_3$). **^{1}H NMR (300 MHz, $CDCl_3$)** δ 7.85 (d, J = 8.0 Hz, 2H), 7.18 (dd, J = 8.2, 5.6 Hz, 4H), 6.82 (d, J = 8.4 Hz, 2H), 4.62 (q, J = 6.9 Hz, 1H), 3.75 (s, 3H), 2.35 (s, 3H), 1.49 (d, J = 6.8 Hz, 3H). **^{13}C NMR (75 MHz, $CDCl_3$)** δ 200.3,

158.6, 143.6, 134.2, 133.9, 129.3, 129.0, 128.9, 114.5, 55.4, 47.0, 21.7, 19.6. **HRMS (ESI)** Calcd for $C_{17}H_{18}O_2Na$ ([M+Na]$^+$) 277.1204, found 277.1193.

(2S)-1,2-bis(4-methylphenyl)propan-1-one (6u)

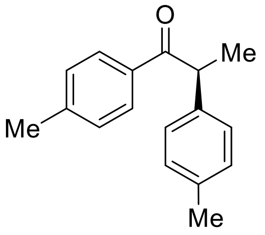

Prepared according to General Procedure B using $(CuOTf)_2$.Tol (4 mol%), diaryliodonium salt **5b** (36 mg, 0.07 mmol, 1 equiv.), PO Ligand **3x** (12 mol%) and **4b** (33 mg, 0.151 mmol, 2 equiv.) in dichloromethane (0.4 mL) at room temperature for 16 hours; flash chromatography on silica gel (hexane: dichloromethane 6:4) provided the title compound (15.8 mg, 0.066 mmol, 88% yield (90% NMR yield), 94.5:5.5 *er*) as colourless oil (HPLC analysis of the product: Phenomenex cellulose-3, Hexane: 2-propanol 99:1, flow: 0.8 mL/min, 254 nm, T_R = 17.2 min (minor) and T_R = 21.0 min (major). $[\alpha]^{22}_D$ = 56.9 (c = 1.6, $CHCl_3$). **^{1}H NMR (300 MHz, $CDCl_3$)** δ 7.85 (d, *J* = 8.0 Hz, 2H), 7.17 (d, *J* = 8.0 Hz, 4H), 7.09 (d, *J* = 7.9 Hz, 2H), 4.63 (q, *J* = 6.8 Hz, 1H), 2.35 (s, 3H), 2.28 (s, 3H), 1.50 (d, *J* = 6.8 Hz, 3H). **^{13}C NMR (75 MHz, $CDCl_3$)** δ 200.2, 143.6, 138.9, 136.5, 134.2, 129.8, 129.3, 129.0, 127.7, 47.5, 21.7, 21.1, 19.6. **HRMS (ESI)** Calcd for $C_{17}H_{19}O$ ([M+H]$^+$) 239.1436, found 239.1431.

(2S)-2-(3-methylphenyl)-1-(4-methylphenyl)propan-1-one (6v)

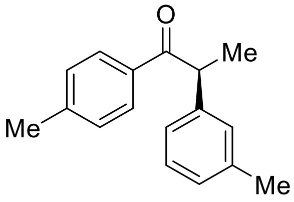

Prepared according to General Procedure B using $(CuOTf)_2$.Tol (4 mol%), diaryliodonium salt **5i** (45 mg, 0.093 mmol, 1 equiv.), PO Ligand **3x** (12 mol%) and **4b** (62 mg, 0.279 mmol, 3 equiv.) in dichloromethane (0.46 mL) at room temperature for 16 hours; flash chromatography on silica gel (hexane: dichloromethane 6:4) provided the title compound (10 mg, 0.041 mmol, 45% yield (59% NMR yield), 95.5:4.5 *er*) as colourless oil (HPLC analysis of the product: Phenomenex cellulose-3, Hexane: 2-propanol 98:2, flow: 0.5 mL/min, 254 nm, T_R = 19.9 min (major) and T_R = 22.2 min (minor). $[\alpha]^{22}_D$ = 84.7 (c = 0.9, $CHCl_3$). **^{1}H NMR (300 MHz, $CDCl_3$)** δ 7.87 (d, *J* = 8.3 Hz, 2H), 7.17 (dd, *J* = 7.9, 4.2 Hz, 3H), 7.09 (d, *J* = 2.3 Hz, 2H), 7.00 (d, *J* = 7.5 Hz, 1H), 4.63 (q, *J* = 6.8 Hz, 1H), 2.35 (s, 3H), 2.30 (s, 3H), 1.51 (d, *J* = 6.9 Hz, 3H). **^{13}C NMR (75 MHz, $CDCl_3$)** δ 200.2, 143.6, 141.8, 138.7, 134.2, 129.3, 129.1, 128.9, 128.5, 127.7, 125.0, 47.8, 21.7, 21.6, 19.7. **HRMS (ESI)** Calcd for $C_{17}H_{19}O$ ([M+H]$^+$) 239.1436, found 239.1431.

(2S)-2-(2,3-dihydro-1H-inden-5-yl)-1-(4-methylphenyl)propan-1-one (6w)

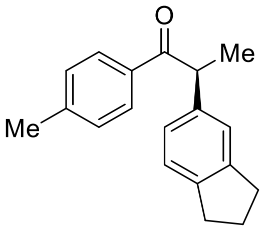

Prepared according to General Procedure B using $(CuOTf)_2$.Tol (4 mol%), diaryliodonium salt **5j** (137 mg, 0.267 mmol, 1 equiv.), PO Ligand **3x** (12 mol%) and **4b** (177 mg, 0.803 mmol, 3 equiv.) in dichloromethane (1.3 mL) at room temperature for 16 hours; flash chromatography on silica gel (hexane: dichloromethane 6:4) provided the title compound (45 mg, 0.168 mmol, 63% yield (68% NMR yield), 95:5 *er*) as colourless oil (HPLC analysis of the product: Phenomenex cellulose-3, Hexane: 2-propanol 99:1, flow: 0.8 mL/min, 254 nm, T_R = 16.0 min (minor) and T_R = 28.5 min (major). $[\alpha]^{22}_D$ = 65.1 (c = 3.0, $CHCl_3$). **^{1}H NMR (300 MHz, $CDCl_3$)** δ 7.89 (d, *J* = 8.0 Hz, 2H), 7.16 (dd, *J* = 12.4, 7.5 Hz, 4H), 7.06 (d, *J* = 7.7 Hz, 1H), 4.65 (q, *J* = 6.8 Hz, 1H), 2.85 (q, *J* = 6.9 Hz, 4H), 2.35 (s, 3H), 2.03 (p, *J* = 7.4 Hz, 2H), 1.52 (d, *J* = 6.9 Hz, 3H). **^{13}C NMR (75 MHz, $CDCl_3$)** δ 200.3, 145.2, 143.5, 143.0, 139.7, 134.2, 129.3, 129.1, 125.8, 124.9, 123.7, 47.7, 32.9, 32.6, 25.5, 21.7, 19.8. **HRMS (ESI)** Calcd for $C_{19}H_{20}ONa$ ([M+Na]$^+$) 287.1412, found 287.1404.

(2S)-1-(4-methylphenyl)-2-(naphthalen-2-yl)propan-1-one (6x)

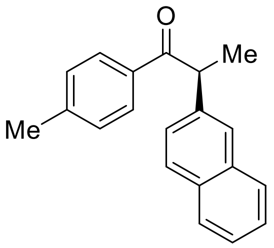

Prepared according to General Procedure B using $(CuOTf)_2$.Tol (4 mol%), diaryliodonium salt **5k** (88 mg, 0.167 mmol, 1 equiv.), PO Ligand **3x** (12 mol%) and **4b** (111 mg, 0.503 mmol, 3 equiv.) in dichloromethane (0.8 mL) at room temperature for 16 hours; flash chromatography on silica gel (hexane: dichloromethane 6:4) provided the title compound (35 mg, 0.128 mmol, 77% yield (81% NMR yield), 96:4 *er*) as white solid (HPLC analysis of the product: Phenomenex cellulose-3, Hexane: 2-propanol 98:2, flow: 0.8 mL/min, 254 nm, T_R = 30.3 min (minor) and T_R = 33.6 min (major). $[\alpha]^{22}_D$ = 81.9 (c = 1.5, $CHCl_3$). **^{1}H NMR (200 MHz, $CDCl_3$)** δ 7.91 (d, *J* = 8.2 Hz, 2H), 7.77 (td, *J* = 7.3, 6.3, 1.9 Hz, 4H), 7.50 – 7.35 (m, 3H), 7.16 (d, *J* = 7.9 Hz, 2H), 4.84 (q, *J* = 6.8 Hz, 1H), 2.33 (s, 3H), 1.62 (d, *J* = 6.8 Hz, 3H). **^{13}C NMR (50 MHz, $CDCl_3$)** δ 200.0, 143.7, 139.4, 134.1, 133.8, 132.5, 129.3, 129.1, 128.9, 127.8, 127.7, 126.5, 126.2, 126.1, 125.8, 48.0, 21.7, 19.6. **HRMS (ESI)** Calcd for $C_{20}H_{19}O$ ([M+H]$^+$) 275.1436, found 275.1425.

(2S)-2-phenyl-3,4-dihydronaphthalen-1(2H)-one (6z)

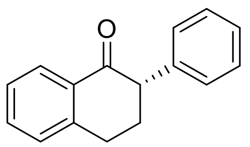

Prepared according to General Procedure B using $(CuOTf)_2$.Tol (4 mol%), MesPhIOTf **5a** (47 mg, 0.1 mmol, 1 equiv.), PO Ligand **3x** (12 mol%) and **4m** (66 mg, 0.3 mmol, 3 equiv.) in dichloromethane (0.5 mL) at room temperature for 16 hours; flash chromatography on silica gel (hexane: dichloromethane 6:4) provided the title compound (6.6 mg, 0.03 mmol, 30% yield (28% NMR yield), 94:6 *er*) as white solid (HPLC analysis of the product: Daicel AS-H, Hexane: 2-propanol 98:2, flow: 1 mL/min, 254 nm, T_R = 13.1 min (minor) and T_R = 19.3 min (major). $[\alpha]^{22}_D$ = 1.7 (c = 0.6, $CHCl_3$).**^{1}H NMR (300 MHz, $CDCl_3$)** δ 8.10 (d, *J* = 7.7 Hz, 1H), 7.50 (t, *J* = 7.4 Hz, 1H), 7.38 – 7.23 (m, 5H), 7.19 (d, *J* = 6.7 Hz, 2H), 3.80 (t, *J* = 7.8 Hz, 1H), 3.22 – 2.89 (m, 2H), 2.44 (td, *J* = 7.4, 6.7, 3.5 Hz, 2H). **^{13}C NMR (75 MHz, $CDCl_3$)** δ 199.7, 145.5, 141.2, 134.9, 134.4, 130.3, 130.0, 129.9, 129.3, 128.4, 128.3, 55.9, 32.7, 30.2. **HRMS (ESI)** Calcd for $C_{16}H_{15}O$ ($[M+H]^+$) 223.1123, found 223.1111. The spectral data are in agreement with literature data.[25]

(3*R*)-3-phenyl-2,3-dihydro-4*H*-1-benzopyran-4-one (**6aa**).

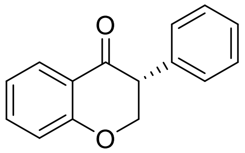

Prepared according to General Procedure B using $(CuOTf)_2$.Tol (4 mol%), MesPhIOTf **5a** (52.8 mg, 0.102 mmol, 1 equiv.), PO Ligand **3x** (12 mol%) and **4aa** (74 mg, 0.336 mmol, 3 equiv.) in dichloromethane (0.56 mL) at room temperature for 16 hours; flash chromatography on silica gel (hexane: dichloromethane 6:4) provided the title compound (20 mg, 0.089 mmol, 88% yield (NMR yield was not determined due to peaks overlaying), 76:24 *er*) as a yellow solid (HPLC analysis of the product: Daicel AS-H, Hexane: 2-propanol 98:2, flow: 0.8 mL/min, 254 nm, T_R = 17.3 min (minor) and T_R = 24.3 min (major). $[\alpha]^{15}_D$ = +4.23 (c = 1.0, $CHCl_3$). **^{1}H NMR (300 MHz, $CDCl_3$)** δ 7.96 (d, *J* = 7.8 Hz, 1H), 7.50 (t, *J* = 7.7 Hz, 1H), 7.37 – 7.24 (m, 5H), 7.09 – 6.97 (m, 2H), 4.67 (d, *J* = 7.1 Hz, 2H), 3.99 (t, *J* = 7.1 Hz, 1H). **^{13}C NMR (75 MHz, $CDCl_3$)** δ 193.6, 163.1, 137.5, 136.5, 130.4, 130.1, 129.3, 123.1, 122.6, 119.4, 73.0, 53.8. **HRMS (ESI)** Calcd for $C_{15}H_{13}O_2$ ($[M+H]^+$) : 225.0916, found: 225.0904.

(*R*)-Methyl 2-phenylpropanoate (*ent*-6ab)

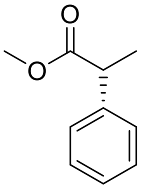

Prepared according to General Procedure B using $(CuOTf)_2$.Tol (4 mol%), MesPhIOTf **5a** (43 mg, 1 equiv.), PO Ligand *ent*-**3z** (12 mol%) and **4ab** (3 equiv.) in dichloromethane (0.45 mL) at room temperature for 16 hours; flash chromatography on silica gel (hexane: dichloromethane 6:4) provided the title compound (7.5 mg, 0.045 mmol, 50% yield (51% NMR yield), 90:10 *er* (HPLC: Phenomenex cellulose-3, Hexane: 2-propanol 98:2, flow: 0.8 mL/min, 220 nm, T_R =11.3 min (minor) and T_R =15.4 min (major)) as as colourless oil. **^{1}H NMR (300 MHz, $CDCl_3$)** δ 7.35 – 7.17 (m, 5H), 3.72 (q, *J* = 7.6, 7.0 Hz, 1H), 3.66 (s, 3H), 1.50 (d, *J* = 7.0 Hz, 3H). **^{13}C NMR (75 MHz, CDCl3)** δ 176.5, 142.1, 130.1, 129.0, 128.6, 53.5, 46.9, 20.1. The spectral data are in agreement with literature data.[26]

(2*S*)-2-(Naphthalen-2-yl)pentan-3-one (6ac)

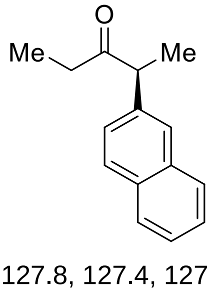

Prepared according to General Procedure B using $(CuOTf)_2$.Tol (4 mol%), diaryliodonium salt **5k** (48.5 mg, 0.092 mmol, 1 equiv.), PO Ligand **3x** (12 mol%) and **4ac** (44 mg, 0.278 mmol, 3 equiv.) in dichloromethane (0.46 mL) at room temperature for 16 hours; flash chromatography on silica gel (hexane: dichloromethane 6:4) provided the title compound (12 mg, 0.056 mmol, 55% yield (61% NMR yield), 87:13 *er*) as as colourless oil (HPLC analysis of the product: : Daicel AS-H, Hexane 100 %, flow: 0.8 mL/min, 225 nm, T_R = 28.9 min (major) and T_R = 34.8 min (minor). $[\alpha]^{15}_D$ = +164.3 (c = 0.6, $CHCl_3$). **^{1}H NMR (300 MHz, $CDCl_3$)** δ 7.81 (d, *J* = 8.0 Hz, 3H), 7.68 (s, 1H), 7.46 (m, 2H), 7.32 (dd, *J* = 8.5, 1.8 Hz, 1H), 3.93 (q, *J* = 7.0 Hz, 1H), 2.41 (m, 2H), 1.48 (d, *J* = 7.0 Hz, 3H), 0.97 (t, *J* = 7.3 Hz, 3H). **^{13}C NMR (75 MHz, $CDCl_3$)** δ 213.0, 139.9, 135.1, 134.0, 130.1, 129.2, 128.1, 127.8, 127.4, 127.3, 54.3, 35.8, 19.0, 9.5. **HRMS (ESI)** Calcd for $C_{15}H_{17}O$ ($[M+H]^+$) : 213.1279, found: 213.1291.

(3S)-3-phenylheptan-4-one (6ad)

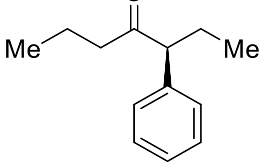

Prepared according to General Procedure B using $(CuOTf)_2$.Tol (4 mol%), MesPhIOTf **5a** (52 mg, 0.110 mmol, 1 equiv.), PO Ligand **3x** (12 mol%) and **4n** (62 mg, 0.333 mmol, 3 equiv.) in dichloromethane (0.55 mL) at room temperature for 16 hours; flash chromatography on silica gel (hexane: dichloromethane 6:4) provided the title compound (12 mg, 0.063 mmol, 57% yield (61% NMR yield) 89:11 *er*) as colourless oil (HPLC analysis of the product: Daicel AS-H, Hexane 100, flow: 1 mL/min, 215 nm, T_R = 8.9 min (major) and T_R = 9.9 min (minor). $[\alpha]^{22}_D$ = 108.34 (c = 1.2, $CHCl_3$). **^{1}H NMR (200 MHz, $CDCl_3$)** δ 7.45 – 7.13 (m, 5H), 3.56 (t, *J* = 7.4 Hz, 1H), 2.37 (t, *J* = 7.3 Hz, 2H), 2.10 (dp, *J* = 14.5, 7.3 Hz, 1H), 1.74 (dt, *J* = 13.6, 7.4 Hz, 1H), 1.55 (tdd, *J* = 14.0, 6.0, 3.4 Hz, 2H), 0.84 (q, *J* = 7.2 Hz, 6H). **^{13}C NMR (50 MHz, $CDCl_3$)** δ 210.8, 139.2, 128.9, 128.5, 128.4, 127.2, 61.0, 44.0, 25.4, 17.3, 13.7, 12.3. **HRMS (ESI)** Calcd for $C_{13}H_{18}ONa$ ($[M+Na]^+$) 213.1255, found 213.1595. The spectral data are in agreement with literature data.[27]

(3*S*)-3-(naphthalen-2-yl)heptan-4-one (6ae)

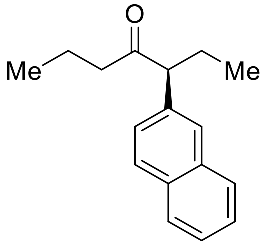

Prepared according to General Procedure B using $(CuOTf)_2$.Tol (4 mol%), diaryliodonium salt **5k** (96 mg, 0.186 mmol, 1 equiv.), PO Ligand **3x** (12 mol%) and **4n** (103 mg, 0.55 mmol, 3 equiv.) in dichloromethane (0.92 mL) at room temperature for 16 hours; flash chromatography on silica gel (hexane: dichloromethane 6:4) provided the title compound (22 mg, 0.091 mmol, 49% yield (49% NMR yield), 85:15 *er*) as as colourless oil. **^{1}H NMR (300 MHz, $CDCl_3$)** δ 7.80 (d, *J* = 7.6 Hz, 3H), 7.67 (s, 1H), 7.46 (dd, *J* = 6.6, 3.1 Hz, 2H), 7.32 (d, *J* = 8.4 Hz, 1H), 3.68 (t, *J* = 7.6 Hz, 1H), 2.36 (t, *J* = 6.7 Hz, 2H), 2.14 (dp, *J* = 13.8, 7.8, 6.9 Hz, 1H), 1.81 (dp, *J* = 14.0, 7.3 Hz, 1H), 1.52 (dhept, *J* = 13.5, 7.2, 6.5 Hz, 2H), 0.85 (t, *J* = 6.8 Hz, 3H), 0.77 (t, *J* = 6.8 Hz, 3H). **^{13}C NMR (75 MHz, $CDCl_3$)** δ 212.1, 138.1, 135.1, 134.1, 130.0, 129.2, 129.2, 128.8, 127.7, 127.3, 62.4, 45.5, 26.7, 18.7, 15.1, 13.6. **HRMS (ESI)** Calcd for $C_{17}H_{21}O$ ($[M+H]^+$) : 241.1592, found: 241.1595.

(3*S*,4*R*)-3-(naphthalen-2-yl)heptan-4-ol (13)

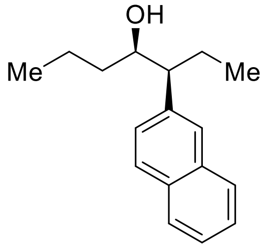

In accordance to a reported procedure, a solution of lithium aluminium hydride (1.0 M in hexanes; 99 µL, 0.099 mmol) was slowly added to a −78 °C solution of (*S*)-**6ae** (20 mg, 0.083 mmol) in THF (1 mL) in a 10-mL RBF under argon. The reaction mixture was allowed to warm to 0 °C and then it was stirred at 0 °C for 2 h. Next, water (0.5 mL) was added dropwise to the reaction mixture at 0 °C to quench the reaction. Aqueous HCl (1.0 N; 0.5 mL) was added, and the mixture was extracted with EtOAc (3x5 mL). The combined organic extracts were dried over $NaSO_4$, filtered, and concentrated. The residue was purified by flash chromatography on silica gel, which afforded the alcohol as colourless oil (20 mg, 99% yield; >20 :1 dr, 85:15 *er* (HPLC analysis of the product: Phenomenex cellulose-3, Hexane: 2-propanol 99:1, flow: 0.5 mL/min, 215 nm, T_R = 24.6 min (minor) and T_R = 30.4 min (major). $[\alpha]^{15}_D$ = +20.16 (c = 1.0, $CHCl_3$). **^{1}H NMR (300 MHz, $CDCl_3$)** δ 7.81 (d, *J* = 8.7 Hz, 3H), 7.66 (d, *J* = 1.7 Hz, 1H), 7.53 – 7.34 (m, 3H), 3.85 (ddd, *J* = 8.6, 5.9, 2.9 Hz, 1H), 2.65 (dt, *J* = 10.6, 5.5 Hz, 1H), 1.85 (m, 2H), 1.53 (m, 2H), 1.45 – 1.22 (m, 2H), 0.92 (t, *J* = 6.9 Hz, 3H), 0.80 (t, *J* = 7.4 Hz, 3H). **^{13}C NMR (75 MHz, $CDCl_3$)** δ 140.5, 135.0, 134.0, 129.5, 129.4, 129.1, 129.1, 128.5, 127.5, 126.9, 76.0, 55.7, 38.9, 26.5, 20.5, 15.6, 13.8.

(4*S*)-4-(naphthalen-2-yl)nonan-5-one (6af)

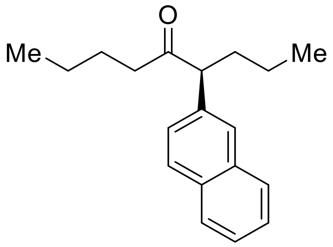

Prepared according to General Procedure B using $(CuOTf)_2$.Tol (4 mol%), diaryliodonium salt **5k** (77 mg, 0.147 mmol, 1 equiv.), PO Ligand **3x** (12 mol%) and **4x** (95 mg, 0.441 mmol, 3 equiv.) in dichloromethane (0.73 mL) at room temperature for 16 hours; flash chromatography on silica gel (hexane: dichloromethane 6:4) provided the title compound (15 mg, 0.07 mmol, 48% yield (59% NMR yield), 88:12 *er*) as as colourless oil.**^{1}H NMR (300 MHz, $CDCl_2$)** δ 7.80 (d, *J* = 7.6 Hz, 3H), 7.67 (s, 1H), 7.46 (dt, *J* = 6.0, 2.1 Hz, 2H), 7.33 (d, *J* = 8.5 Hz, 1H), 3.79 (t, *J* = 7.5 Hz, 1H), 2.38 (t, *J* = 7.4 Hz, 2H), 2.08 (ddt, *J* = 13.4, 9.3, 6.7 Hz, 1H), 1.79 (ddd, *J* = 14.9, 10.2, 6.3 Hz, 1H), 1.48 (tt, *J* = 14.9, 6.7 Hz, 2H), 1.20 (dh, *J* = 21.9, 7.1 Hz, 4H), 0.89 (t, *J* = 7.3 Hz, 3H), 0.79 (t, *J* = 7.3 Hz, 3H). **^{13}C NMR (75 MHz, $CDCl_3$)** δ 213.0, 138.3, 135.1, 134.1, 130.0, 129.2, 129.1, 128.7, 127.7, 127.3, 60.4, 43.2, 35.8, 27.4, 23.7, 22.2, 15.5, 15.3. **HRMS (ESI)** Calcd for $C_{19}H_{25}O$ ($[M+H]^+$) : 269.1905, found: 269.1913.

(4*S*,5*R*)-4-(naphthalen-2-yl)nonan-5-ol (14)

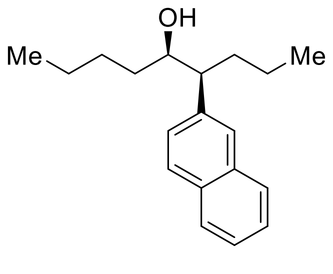

In accordance to a reported procedure, a solution of lithium aluminium hydride (1.0 M in hexanes; 58 µL, 0.058 mmol) was slowly added to a −78 °C solution of (*S*)-**6af** (13 mg, 0.048 mmol) in THF (0.3 mL) in a 10-mL RBF under argon. The reaction mixture was allowed to warm to 0 °C and then it was stirred at 0 °C for 2 h. Next, water (0.5 mL) was added dropwise to the reaction mixture at 0 °C to quench the reaction. Aqueous HCl (1.0 N; 0.5 mL) was added, and the mixture was extracted with EtOAc (3x5 mL). The combined organic extracts were dried over $NaSO_4$, filtered, and concentrated. The residue was purified by flash chromatography on silica gel, which afforded the alcohol as colourless oil (9 mg, 70% yield; >20 :1 dr, 88:12 *er* HPLC: Phenomenex cellulose-3, Hexane: 2-propanol 99.5:0.5, flow: 0.5 mL/min, 215 nm, T_R = 24.8 min (minor) and T_R = 30.4 min (major). $[\alpha]^{15}_D$ = +22.84 (c = 0.25, $CHCl_3$). **^{1}H NMR (300 MHz, $CHCl_3$)** δ 7.99 – 7.76 (m, 3H), 7.68 (dd, *J* = 18.5, 1.7 Hz, 1H), 7.57 – 7.34 (m, 3H), 3.81 (dq, *J* = 9.1, 3.6, 3.1 Hz, 1H), 2.76 (dt, *J* = 9.2, 6.0 Hz, 1H), 1.78 (m, 2H), 1.52-1.29 (m, 6H), 1.25-1.10 (m, 2H), 0.89 (t, *J* = 7.0 Hz, 3H), 0.86 (t, *J* = 7.3 Hz, 3H). **^{13}C NMR (75 MHz, $CDCl_3$)** δ 140.7, 134.9, 134.0, 129.6, 129.3, 129.1, 129.1, 128.5, 127.5, 126.9, 76.5, 53.5, 36.4, 35.8, 29.5, 24.3, 22.3, 15.6 (2C). **HRMS (ESI)** Calcd for $C_{19}H_{26}ONa$ ($[M+Na]^+$) : 293.1881, found: 293.1872.

Multidimensional correlation analysis

Geometry optimizations and frequency calculations were carried out using Gaussian 16.[21] Vibrational frequencies and intensities were calculated at the M06-2X/6-31G(d) level of theory. Sterimol values **B1**, **B5**, and **L** were calculated for the M06/2X optimized geometries using Molecular Modeling Pro®.[22] Buried volumes V% were evaluated using SambVca 2.0.[23] Multidimensional regression analyses were performed using Matlab®.[24]

Parameters for the ligands were calculated from the whole structure as truncated versions provided biased structural features. The graphical description of the parameters acquired is reported in Figure S1. The values of the parameters collected for ligands **3a-3x** are reported in Table S1 together with the selectivity they have shown in our benchmark reaction (see *Reaction optimization* section). Here, the selectivity is expressed as ΔΔG‡ in kcal/mol. ΔΔG‡ values were calculated using the formula ΔΔG‡ = -RTln(*er*) where R is the gas constant, T is temperature, and *er* is the enantiomeric ratio.

Figure S1.

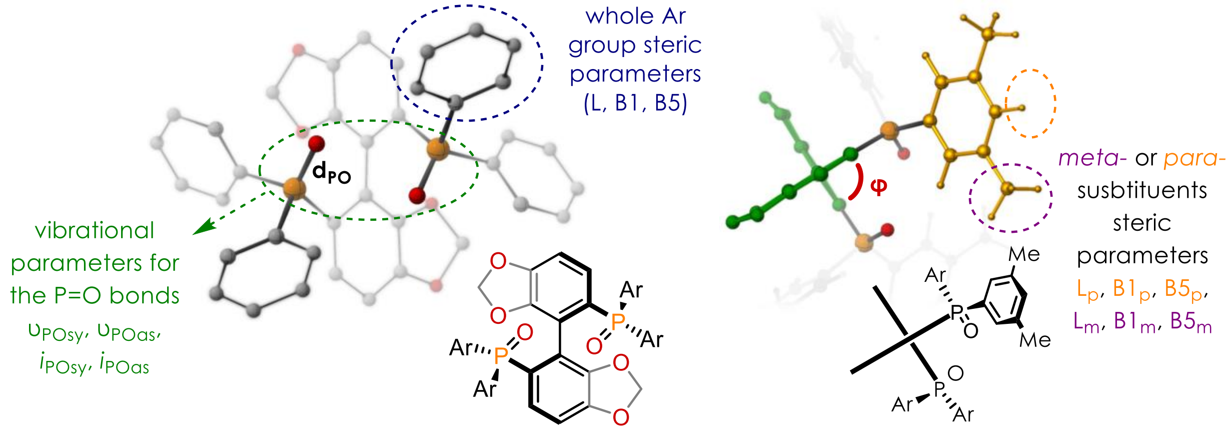

υ_{POsy} = symmetric stretching frequency of the P=O bonds
υ_{POas} = asymmetric stretching frequency of the P=O bonds
i_{POsy} = symmetric stretching intensity of the P=O bonds
i_{POas} = asymmetric stretching intensity of the P=O bonds
L = length of the aryl group
B1 = minimum width (or thickness) of the aryl group
B5 = maximum width of the aryl group
L_p = length of the aryl para- substituent
$B1_p$ = minimum width of the aryl para- substituent
$B5_p$ = maximum width of the aryl para- substituent
L_m = length of the aryl meta-substituent
$B1_m$ = minimum width of the aryl meta-substituent
$B5_m$ = maximum width of the aryl meta-substituent
φ = dihedral angle of the biaryl scaffold
d_{PO} = P-O bond length

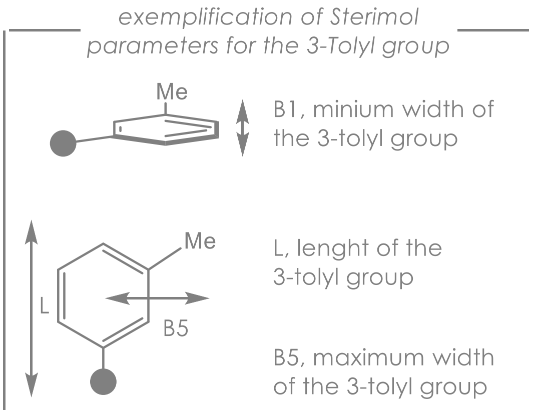

Table S1.

	Ligand	er	ΔΔG‡ (kcal/mol)	L	B1	B5	L_m	$B1_m$	$B5_m$	L_p	$B1_p$	$B5_p$	φ (deg)	d_{PO}	υ_{POsy}	i_{POsy}	υ_{POas}	i_{POas}
**3a**	BINAPO	74 : 26	0.62	6.34	1.77	3.23	2.08	1.00	1.00	2.08	1.00	1.00	86.6	1.49887	1233.27	15.19	1229.93	204.25
**3b**	Tol-BINAPO	71 : 29	0.53	7.36	1.77	3.40	2.08	1.00	1.00	3.07	1.70	2.19	83.5	1.49939	1231.67	37.38	1225.90	173.37
**3c**	H_8-BINAPO	66 : 34	0.39	6.34	1.77	3.23	2.08	1.00	1.00	2.08	1.00	1.00	81.1	1.49935	1237.13	21.87	1235.56	79.81
**3d**	OMe-BIPHEPO	75 : 25	0.65	6.34	1.77	3.23	2.08	1.00	1.00	2.08	1.00	1.00	87.3	1.4989	1229.95	1.53	1228.04	147.71
**3f**	C1-TUNEPHOSO	60.5 : 39.5	0.25	6.34	1.77	3.23	2.08	1.00	1.00	2.08	1.00	1.00	57.9	1.49958	1239.26	51.04	1228.39	293.41
**3g**	C2-TUNEPHOSO	64 : 36	0.34	6.34	1.77	3.23	2.08	1.00	1.00	2.08	1.00	1.00	65.3	1.4995	1240.56	44.22	1226.71	275.59
**3h**	C3-TUNEPHOSO	69 : 31	0.47	6.34	1.77	3.23	2.08	1.00	1.00	2.08	1.00	1.00	71.7	1.49938	1242.55	57.04	1231.34	261.57
**3i**	GARPHOSO	80.5 : 19.5	0.84	6.34	1.77	3.23	2.08	1.00	1.00	2.08	1.00	1.00	86.2	1.49926	1223.06	1.48	1224.70	271.72
**3j**	Tol-GARPHOSO	78 : 22	0.75	7.38	1.81	3.40	2.08	1.00	1.00	3.07	1.70	2.19	85.6	1.49941	1223.77	0.00	1224.65	233.95
**3k**	DM-GARPHOSO	77 : 23	0.72	6.64	2.05	4.49	3.07	1.70	2.19	2.08	1.00	1.00	85.3	1.49918	1230.85	42.95	1226.92	107.33
**3l**	DMM-GARPHOSO	86 : 14	1.07	8.46	1.95	4.58	3.07	1.70	2.19	4.19	1.52	3.23	85.0	1.49947	1214.19	4.83	1214.70	311.69
**3n**	SEGPHOSO	80.5 : 19.5	0.84	6.34	1.77	3.23	2.08	1.00	1.00	2.08	1.00	1.00	90.3	1.49884	1221.16	11.27	1224.42	304.16
**3o**	DM-SEGPHOSO	78.5 : 21.5	0.77	6.64	2.05	4.49	3.07	1.70	2.19	2.08	1.00	1.00	81.9	1.49895	1214.26	1.95	1222.87	248.32
**3p**	DTBM-SEGPHOSO	55.5 : 44.5	0.13	8.18	3.45	5.92	4.38	2.91	3.35	4.19	1.52	3.23	79.8	1.50043	1216.67	50.29	1215.49	269.36
**3q**	DIFLUORPHOSO	71.5 : 28.5	0.54	6.34	1.77	3.23	2.08	1.00	1.00	2.08	1.00	1.00	80.4	1.49881	1232.81	1.93	1231.52	344.74
**3r**	SYNPHOSO	72 : 28	0.56	6.34	1.77	3.23	2.08	1.00	1.00	2.08	1.00	1.00	90.2	1.49897	1231.05	3.99	1228.34	285.43
**3s**	BITIAMPO	73 : 27	0.59	6.34	1.77	3.23	2.08	1.00	1.00	2.08	1.00	1.00	89.3	1.49745	1236.53	21.22	1234.46	276.56
**3u**	OMe-Cl-BIPHEPO	75.5 : 24.5	0.67	6.34	1.77	3.23	2.08	1.00	1.00	2.08	1.00	1.00	83.0	1.49881	1235.64	20.26	1224.36	235.37
**3v**	4-OMe-GARPHOSO	77 : 23	0.72	8.60	1.90	3.22	2.08	1.00	1.00	4.19	1.52	3.23	85.5	1.50006	1221.59	0.52	1222.58	334.34
**3x**	345-$(OMe)_3$-GARPHOSO	94 : 6	1.63	4.19	1.52	3.23	4.19	1.52	3.23	4.19	1.52	3.23	71.2	1.50124	1211.16	12.18	1209.72	463.52
**3z**	Naphth-GARPHOSO	84 : 16	0.98	8.50	1.77	4.27	4.51	1.70	4.55	4.51	1.70	4.55	71.9	1.49868	1222.30	11.25	1223.13	217.03

Preliminary single parameter correlations showed that ν_{POas} was the only parameter providing a trend with the observed enantioselectivity. However, a good correlation (R^2=0.85) could be obtained only by removing structurally biased ligands (Figure S2). This suggests that the selectivity is due to both electronic and geometrical/steric features of the ligand. Therefore, we reasoned that a comprehensive model could be obtained by multidimensional correlation analysis.

Figure S2.

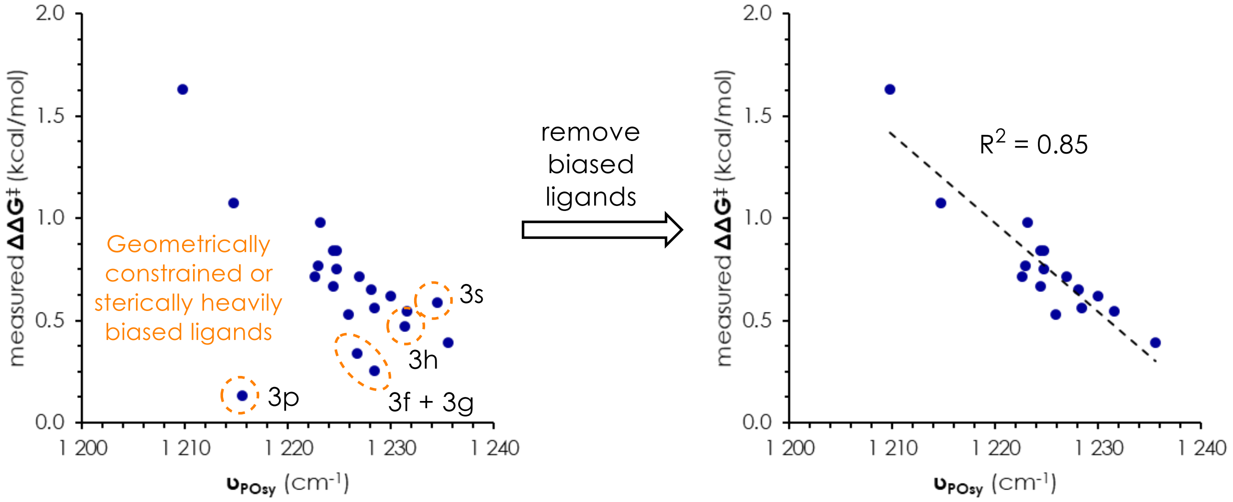

Model development

The linear regression model was developed using the functions *LinearModel.stepwise* and *LinearModel.fit* implemented in MATLAB® R2020a software in order to obtain the predicted ΔΔG‡. A good linear correlation (R^2 close to 1.0 and intercept close to 0.0) between the **Predicted ΔΔG‡** and the **Measured ΔΔG‡** indicates that the obtained model adequately approximates the system under study. The Leave-One-Out (LOO) value was also generated using MATLAB and is reported in the plot. Relevance of the descriptors rendered by the regression procedure is also established by evaluation of the corresponding p-values, which in this case were always < 10^{-4}.

The descriptors present in the model equation support the evidence obtained by single parameter correlation, with **B1**, **B1$_m$** and **φ** descriptors being important contributors to the observed selectivity together with the electronic parameter ν_{POas}.

Figure S3.

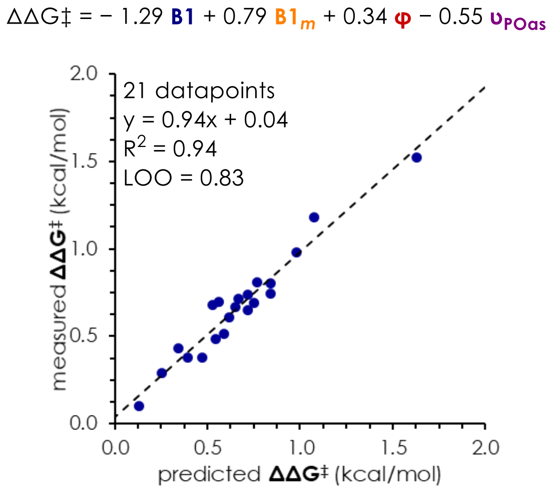

ligand	measured ΔΔG‡ kcal/mol)	predicted ΔΔG‡ kcal/mol)
**3a**	0.62	0.61
**3b**	0.53	0.68
**3c**	0.39	0.38
**3d**	0.65	0.67
**3f**	0.25	0.29
**3g**	0.34	0.43
**3h**	0.47	0.38
**3i**	0.84	0.75
**3j**	0.75	0.69
**3k**	0.72	0.74
**3l**	1.07	1.18
**3n**	0.84	0.81
**3o**	0.77	0.81
**3p**	0.13	0.10
**3q**	0.54	0.48
**3r**	0.56	0.69
**3s**	0.59	0.51
**3u**	0.67	0.72
**3v**	0.72	0.65
**3x**	1.63	1.52
**3z**	0.98	0.98

Kinetic studies

Evaluation of the initial rates for our benchmark reaction in the presence of different amounts of ligand **3a** was accomplished by reaction NMR profiling as follow:

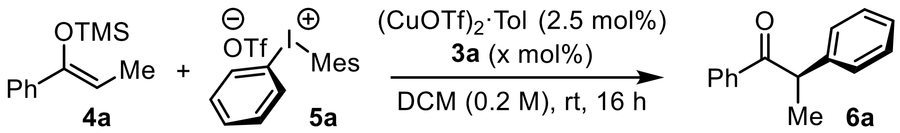

In a N_2-filled glove box, an oven dried vial was charged with $(CuOTf)_2$.Tol (2.5 mol%) and a magnetic stir bar. The vial was sealed with a ptfe septum screw cap and was removed from the glove box. Ligand **3a** (x mol%, see table below), salt **5a** (1.0 equiv.), and a known amount of internal standard (dimethyl terephthalate) were charged in a second sealed vial, placed under N_2, and dissolved in the proper amount of CD_2Cl_2. This solution was added to the vial with $(CuOTf)_2$.Tol and stirred for 60 s, after which 2.5 equiv of **4a** were added by syringe. The mixture was stirred vigorously for 60 s to ensure dissolution of all of the Cu salt, and then was transferred by syringe in NMR tube placed under N_2 inside a Schlenk tube with a rubber septum. The NMR tube was capped under high positive pressure of N_2, sealed with parafilm and placed into the NMR instrument. NMR spectra were recorded every 5 min with the first spectrum being recorded after exactly 10 min from the addition of **4a**.

Concentration of the product **6a** against time are reported below for a set of 5 experiments with [**3a**] ranging from 6 to 30 mol% (Table S2). In Figure S3 the reaction profiles are plotted. Using the first 4 experimental points (from 10 to 25 min) for each profile, an initial rate k_{obs} was determined by linear fitting with Excel. The plot of the reciprocal $1/k_{obs}$ against [**3a**] shows the kinetic order of **3a** to be -1.

Figure S3.

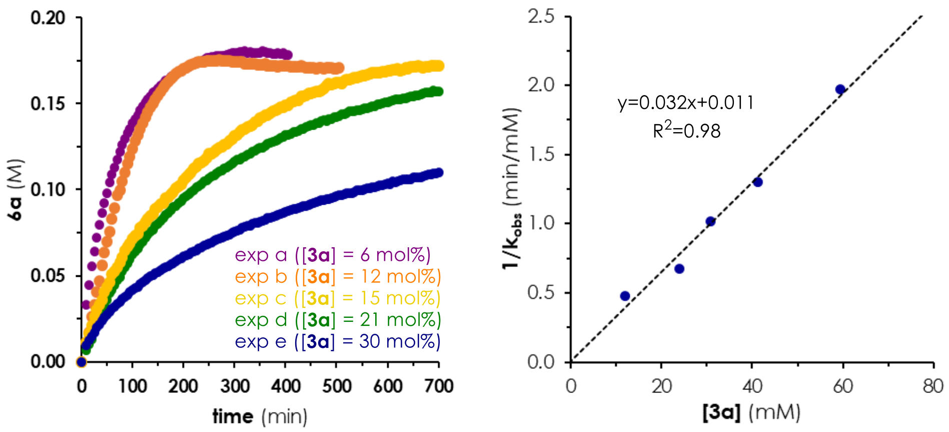

exp	[**3a**] (mM)	k_{obs} (mM/min)	$1/k_{obs}$
a	12	2.085	0.479
b	24	1.479	0.675
c	31	0.984	1.015
d	41	0.774	1.291
e	59	0.507	1.970

Table S2.

	exp a	exp b	exp c	exp d	exp e
**3a** mol%	6	12	15	21	30
[**3a**] (mM)	12	24	31	41	59
t (min)	**6a** (M)	**6a** (M)	**6a** (M)	**6a** (M)	**6a** (M)
0	0.000	0.000	0.000	0.000	0.000
10	0.033	0.012	0.011	0.007	0.010
15	0.045	0.017	0.017	0.010	0.012
20	0.055	0.026	0.021	0.013	0.015

25	0.064	0.033	0.026	0.019	0.017
30	0.072	0.041	0.030	0.022	0.020
35	0.079	0.047	0.033	0.027	0.022
40	0.086	0.056	0.036	0.030	0.024
45	0.092	0.063	0.040	0.033	0.025
50	0.098	0.070	0.043	0.036	0.027
55	0.104	0.076	0.046	0.040	0.029
60	0.109	0.083	0.051	0.042	0.031
65	0.113	0.089	0.053	0.045	0.032
70	0.118	0.097	0.055	0.047	0.034
75	0.122	0.100	0.058	0.049	0.035
80	0.126	0.105	0.060	0.053	0.037
85	0.129	0.110	0.065	0.055	0.038
90	0.133	0.115	0.067	0.057	0.039
95	0.135	0.119	0.069	0.060	0.041
100	0.138	0.124	0.071	0.062	0.042
105	0.141	0.128	0.074	0.064	0.043
110	0.143	0.132	0.075	0.066	0.044
115	0.146	0.135	0.078	0.068	0.045
120	0.149	0.139	0.079	0.070	0.046
125	0.151	0.142	0.081	0.072	0.047
130	0.153	0.145	0.083	0.074	0.048
135	0.154	0.148	0.084	0.076	0.049
140	0.156	0.150	0.086	0.077	0.050
145	0.157	0.152	0.089	0.079	0.051
150	0.159	0.155	0.090	0.080	0.052
155	0.160	0.157	0.092	0.082	0.053
160	0.162	0.159	0.092	0.084	0.054
165	0.163	0.161	0.095	0.085	0.055
170	0.164	0.163	0.096	0.087	0.056
175	0.166	0.164	0.098	0.088	0.056
180	0.166	0.166	0.099	0.090	0.057
185	0.167	0.167	0.101	0.091	0.058
190	0.169	0.168	0.102	0.092	0.059
195	0.169	0.169	0.103	0.093	0.060
200	0.170	0.170	0.105	0.095	0.061
205	0.171	0.171	0.107	0.096	0.062
210	0.172	0.172	0.109	0.098	0.062
215	0.173	0.172	0.110	0.098	0.063
220	0.173	0.173	0.112	0.100	0.064
225	0.174	0.174	0.114	0.101	0.065
230	0.174	0.174	0.115	0.102	0.066
235	0.174	0.174	0.116	0.103	0.067
240	0.175	0.174	0.117	0.105	0.067
245	0.177	0.175	0.119	0.106	0.068
250	0.176	0.175	0.121	0.106	0.069
255	0.177	0.175	0.121	0.107	0.069
260	0.177	0.175	0.122	0.108	0.070
265	0.178	0.175	0.123	0.109	0.071
270	0.178	0.175	0.124	0.111	0.072
275	0.178	0.175	0.125	0.111	0.072
280	0.178	0.175	0.126	0.113	0.073
285	0.178	0.175	0.127	0.113	0.073
290	0.179	0.175	0.128	0.114	0.074
295	0.179	0.174	0.129	0.115	0.075
300	0.179	0.174	0.131	0.116	0.076

305	0.179	0.174	0.132	0.117	0.076
310	0.180	0.174	0.134	0.118	0.077
315	0.180	0.174	0.134	0.118	0.078
320	0.180	0.173	0.135	0.119	0.078
325	0.180	0.173	0.136	0.120	0.079
330	0.180	0.173	0.137	0.121	0.079
335	0.180	0.173	0.138	0.122	0.080
340	0.179	0.173	0.139	0.123	0.081
345	0.179	0.173	0.140	0.123	0.081
350	0.179	0.173	0.142	0.124	0.081
355	0.180	0.172	0.142	0.125	0.082
360	0.180	0.173	0.143	0.125	0.083
365	0.180	0.172	0.143	0.126	0.083
370	0.180	0.172	0.144	0.127	0.084
375	0.179	0.172	0.145	0.128	0.084
380	0.179	0.172	0.146	0.129	0.085
385	0.179	0.171	0.147	0.129	0.085
390	0.179	0.172	0.148	0.130	0.086
395	0.180	0.172	0.148	0.131	0.086
400	0.178	0.172	0.149	0.132	0.087
405	0.179	0.172	0.149	0.132	0.087
410		0.172	0.151	0.133	0.088
415		0.171	0.151	0.133	0.088
420		0.171	0.152	0.134	0.089
425		0.171	0.152	0.134	0.089
430		0.172	0.154	0.135	0.090
435		0.171	0.154	0.135	0.090
440		0.171	0.155	0.136	0.091
445		0.171	0.156	0.137	0.091
450		0.171	0.156	0.137	0.091
455		0.171	0.157	0.138	0.092
460		0.170	0.157	0.139	0.093
465		0.171	0.157	0.139	0.093
470		0.171	0.158	0.140	0.094
475		0.170	0.159	0.140	0.094
480		0.171	0.160	0.140	0.095
485		0.171	0.161	0.141	0.095
490		0.171	0.161	0.141	0.095
495		0.170	0.162	0.142	0.096
500		0.170	0.162	0.142	0.096
505		0.171	0.162	0.143	0.097
510			0.162	0.144	0.097
515			0.163	0.144	0.097
520			0.164	0.144	0.098
525			0.164	0.145	0.098
530			0.165	0.145	0.098
535			0.165	0.146	0.099
540			0.165	0.146	0.099
545			0.165	0.146	0.100
550			0.166	0.147	0.100
555			0.166	0.148	0.100
560			0.166	0.147	0.101
565			0.167	0.148	0.101
570			0.168	0.148	0.101
575			0.168	0.149	0.101
580			0.168	0.149	0.102

585	0.168	0.150	0.103
590	0.169	0.150	0.103
595	0.169	0.151	0.103
600	0.169	0.151	0.103
605	0.169	0.151	0.103
610	0.170	0.152	0.105
615	0.170	0.152	0.104
620	0.170	0.153	0.105
625	0.169	0.153	0.105
630	0.170	0.153	0.106
635	0.171	0.153	0.106
640	0.170	0.154	0.106
645	0.171	0.154	0.106
650	0.171	0.154	0.107
655	0.171	0.155	0.107
660	0.172	0.155	0.107
665	0.171	0.156	0.107
670	0.171	0.156	0.108
675	0.171	0.156	0.108
680	0.172	0.156	0.109
685	0.172	0.156	0.109
690	0.172	0.157	0.110
695	0.172	0.157	0.109
700	0.172	0.157	0.110
705	0.174	0.158	0.110
710	0.172	0.158	0.110
715	0.172	0.158	0.111
720	0.172	0.158	0.111
725	0.172	0.158	0.111
730	0.173	0.159	0.111
735	0.173	0.159	0.112
740	0.173	0.160	0.112
745	0.172		0.112
750			0.113
755			0.112

HPLC traces

STD-product, cellulose-3, Hex:IPA 98:2, 0.8 mL/min, 254 nm

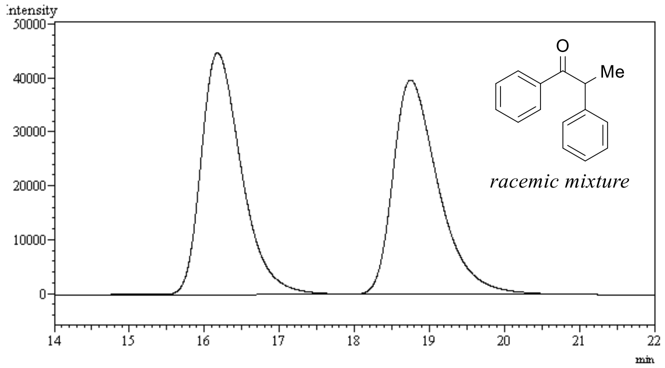

Peak#	Ret.Time	Area	Height	Conc.	Unit	Mark	ID#	Cmpd Name
1	16.177	1671530	44824	50.141				
2	18.751	1662098	39691	49.859				
Total		3333628	84515					

MANU438, cellulose-3, Hex:IPA 98:2, 0.8 mL/min, 254 nm

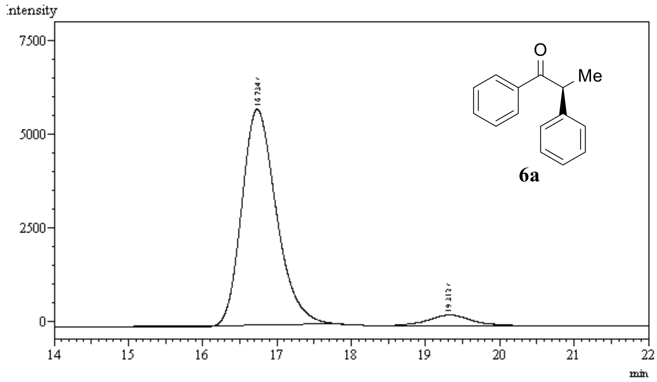

Peak#	Ret.Time	Area	Height	Conc.	Unit	Mark	ID#	Cmpd Name
1	16.734	185978	5775	94.266				
2	19.312	11312	284	5.734				
Total		197290	6059					

FC23, cellulosa-3, Hex/IPA 98:2, 0.8 mL/min

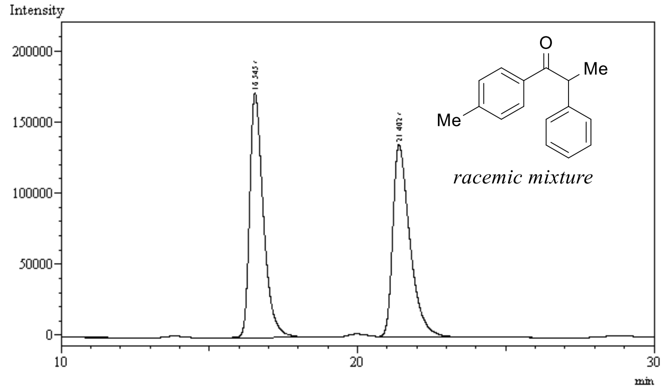

Peak#	Ret.Time	Area	Height	Conc.	Unit	Mark	ID#	Cmpd Name
1	16.545	5522451	171985	50.461				
2	21.402	5421636	135206	49.539				
Total		10944087	307191					

MANU445 f-7 cellulose-3, Hex/IPA 99:1, 0.8 mL/min, 254 nm

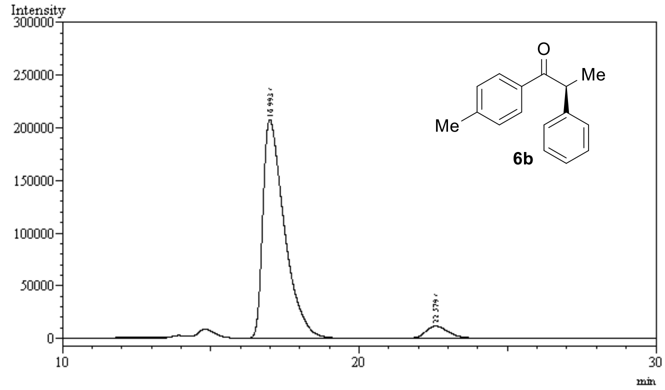

Peak#	Ret.Time	Area	Height	Conc.	Unit	Mark	ID#	Cmpd Name
1	16.993	10488222	207982	94.889				
2	22.579	564929	11083	5.111				
Total		11053151	219065					

MEC-284, cellulose-3, Hexane 98:2, 0.8 mL/min, 254 nm

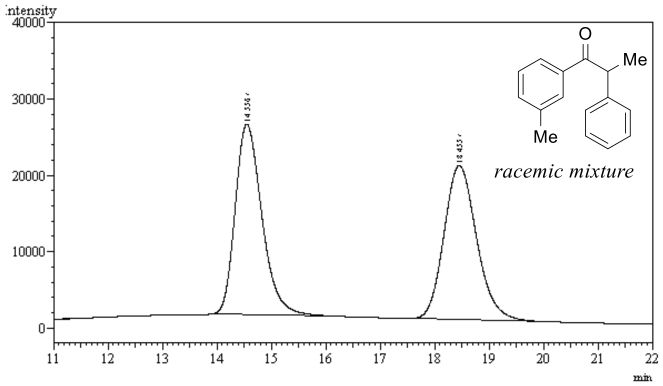

Peak#	Ret.Time	Area	Height	Conc.	Unit	Mark	ID#	Cmpd Name
1	14.556	855177	24971	50.386				
2	18.455	842071	20201	49.614				
Total		1697248	45172					

MEC-283, cellulose-3, Hexane 98:2, 0.8 mL/min, 254 nm

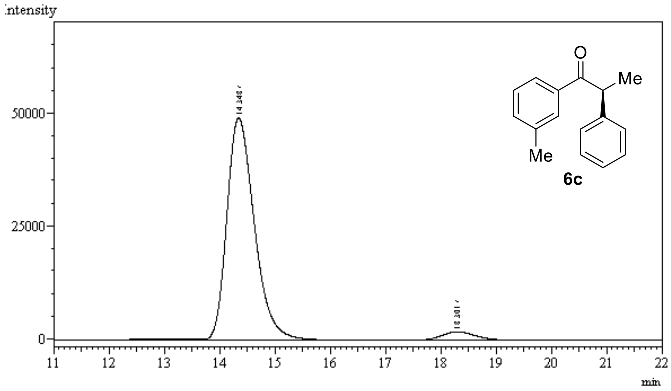

Peak#	Ret.Time	Area	Height	Conc.	Unit	Mark	ID#	Cmpd Name
1	14.348	1697253	49217	96.186				
2	18.301	67294	1728	3.814				
Total		1764547	50945					

MEC065, Cellulose-3, Hex/IPA 98:2, 0.8 mL/min

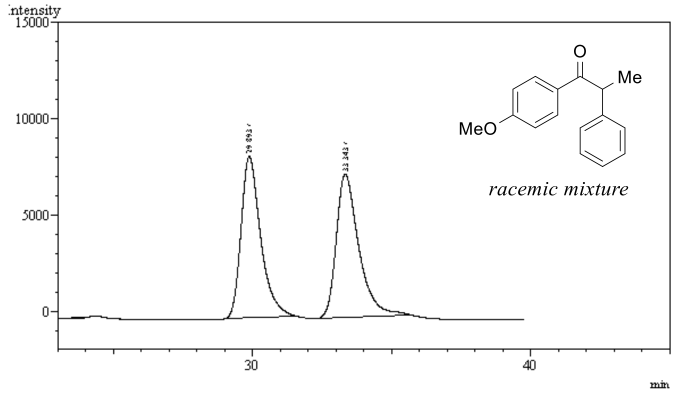

Peak#	Ret.Time	Area	Height	Conc.	Unit	Mark	ID#	Cmpd Name
1	29.893	411100	8376	49.575				
2	33.343	418150	7456	50.425				
Total		829250	15832					

MANU446, cellulose-3, Hex/IPA 98:2, 0.8 mL/min, 254 nm

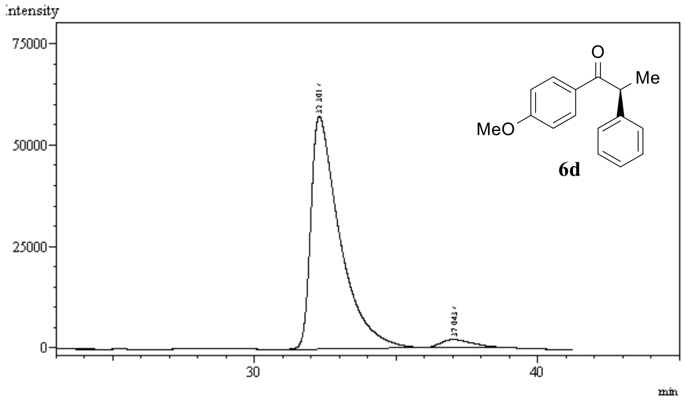

Peak#	Ret.Time	Area	Height	Conc.	Unit	Mark	ID#	Cmpd Name
1	32.301	4073804	57319	96.895				
2	37.043	130539	1832	3.105				
Total		4204343	59151					

MEC-240, cellulose-3, Hex/IPA 98:2, 0.8 mL/min, 254 nm

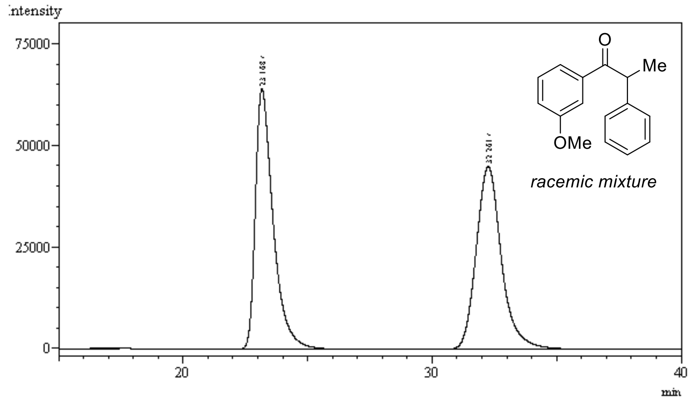

Peak#	Ret.Time	Area	Height	Conc.	Unit	Mark	ID#	Cmpd Name
1	23.168	3165353	64004	50.190				
2	32.261	3141437	44855	49.810				
Total		6306790	108859					

MANU467, cellulose-3, Hex/IPA 98:2, 0.8 mL/min, 245 nm

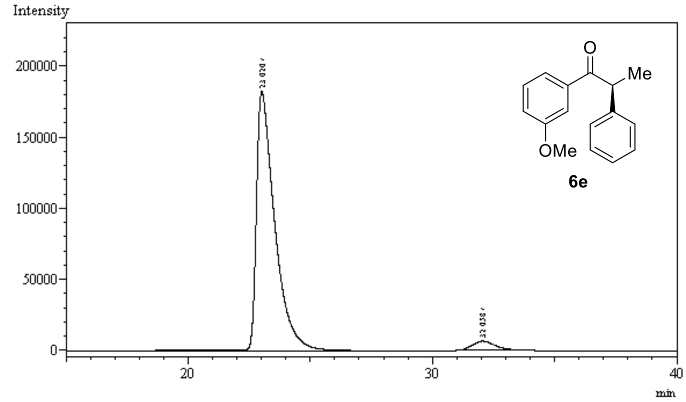

Peak#	Ret.Time	Area	Height	Conc.	Unit	Mark	ID#	Cmpd Name
1	23.020	9431117	182271	96.138				
2	32.058	378833	6149	3.862				
Total		9809950	188420					

MEC-278, racemic, cellulose-3, Hexane 98:2, 0.8 mL/min, 254 nm

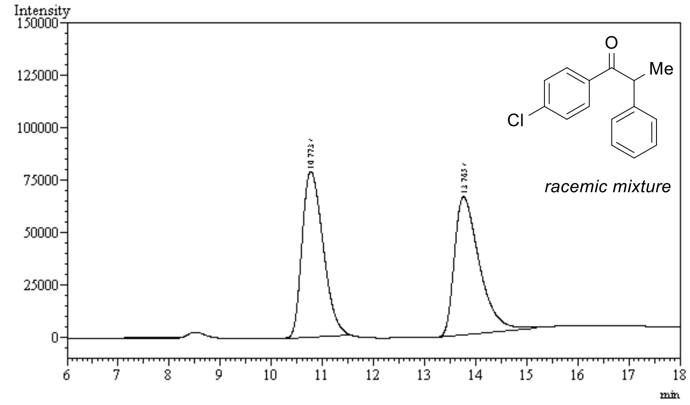

Peak#	Ret.Time	Area	Height	Conc.	Unit	Mark	ID#	Cmpd Name
1	10.773	2274151	79076	50.350				
2	13.765	2242509	65866	49.650				
Total		4516660	144942					

MANU526, Cellulose-3, Hex:IPA 98:2, 0.8 mL/min, 254 nm

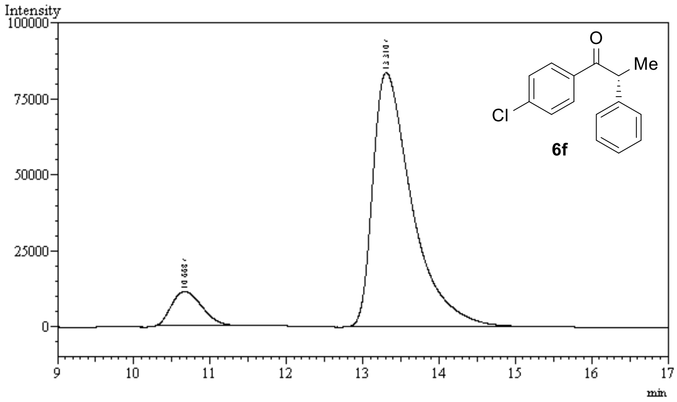

Peak#	Ret.Time	Area	Height	Conc.	Unit	Mark	ID#	Cmpd Name
1	10.668	302455	11121	9.151				
2	13.310	3002584	83835	90.849				
Total		3305039	94956					

MEC-241, cellulose-3, Hex/IPA 98:2, 0.8 mL/min, 254 nm

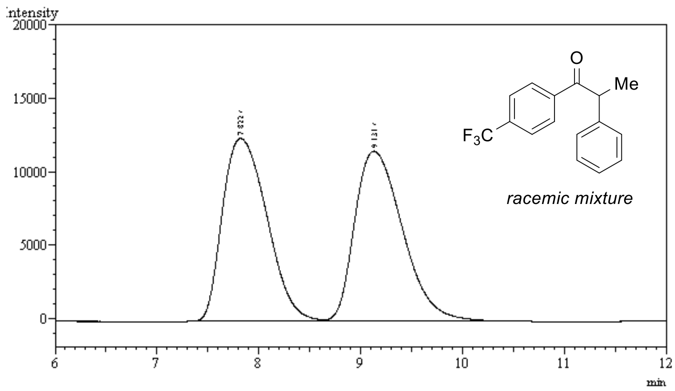

Peak#	Ret.Time	Area	Height	Conc.	Unit	Mark	ID#	Cmpd Name
1	7.822	378368	12467	50.095				
2	9.131	376938	11585	49.905		V		
Total		755306	24052					

MANU525-24h, cellulose-3, Hex:IPA 98:2, 0.8 mL/min, 254 nm

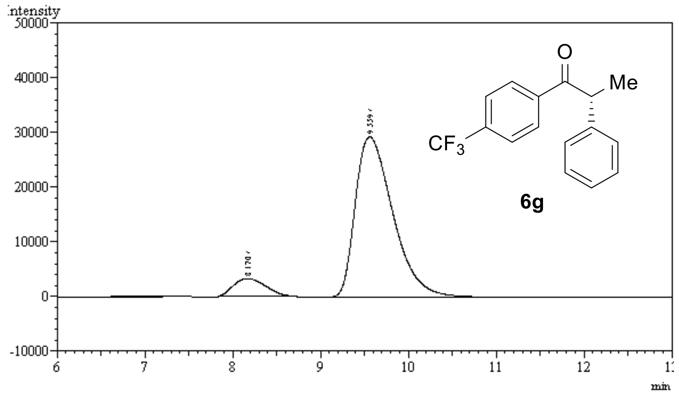

Peak#	Ret.Time	Area	Height	Conc.	Unit	Mark	ID#	Cmpd Name
1	8.170	83334	3266	8.700				
2	9.559	874499	29352	91.300				
Total		957833	32618					

MEC-242, cellulose-3, Hex/IPA 98:2, 0.8 mL/min, 254 nm

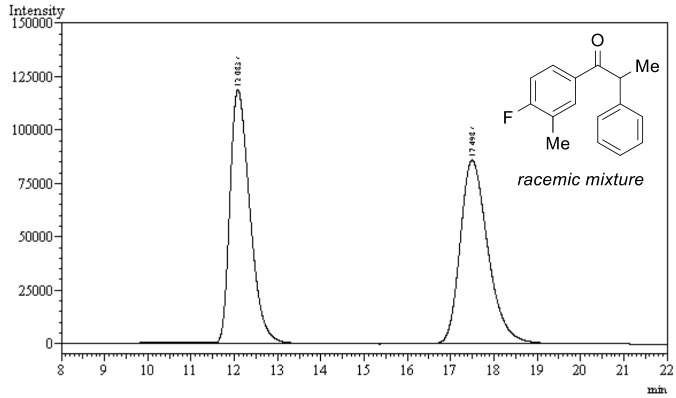

Peak#	Ret.Time	Area	Height	Conc.	Unit	Mark	ID#	Cmpd Name
1	12.083	3821589	118933	50.033				
2	17.498	3816474	85946	49.967				
Total		7638063	204879					

MANU468, cellulose-3, Hex/IPA 98:2, 0.8 mL/min, 245 nm

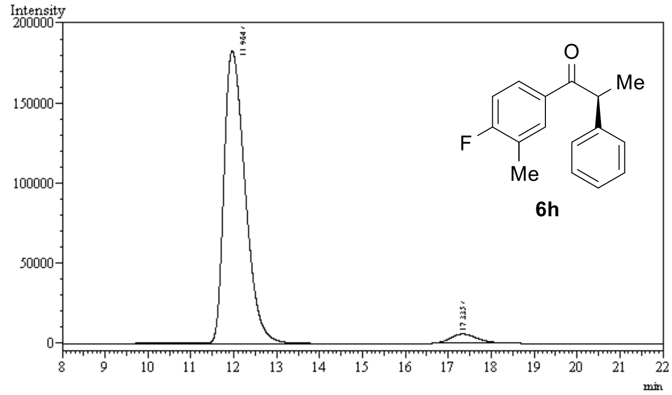

Peak#	Ret.Time	Area	Height	Conc.	Unit	Mark	ID#	Cmpd Name
1	11.964	6236031	182821	96.331				
2	17.335	237490	5634	3.669				
Total		6473521	188455					

MEC-243, cellulose-3, Hex/IPA 98:2, 0.8 mL/min, 210 nm

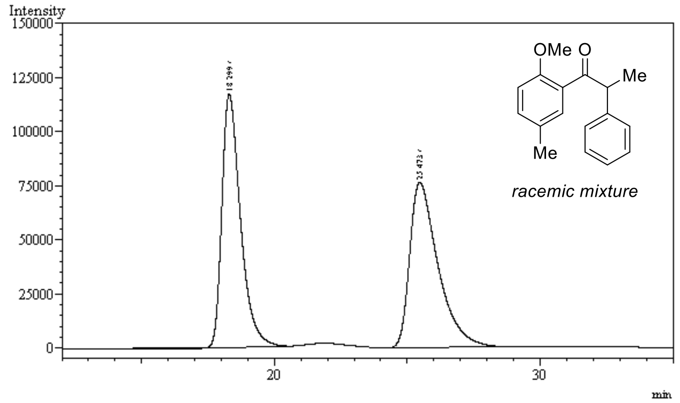

Peak#	Ret.Time	Area	Height	Conc.	Unit	Mark	ID#	Cmpd Name
1	18.299	5720770	117279	50.184				
2	25.473	5678774	76483	49.816				
Total		11399544	193762					

MANU470, cellulose-3, Hex/IPA 98:2, 0.8 mL/min, 245 nm

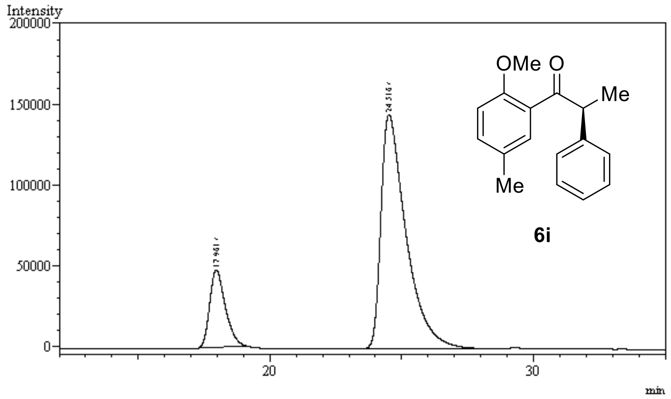

Peak#	Ret.Time	Area	Height	Conc.	Unit	Mark	ID#	Cmpd Name
1	17.961	1981809	47794	17.098				
2	24.516	9608978	144839	82.902				
Total		11590787	192633					

MEC-273, racemic, cellulose-3, Hexane 98:2, 0.8 mL/min, 254 nm

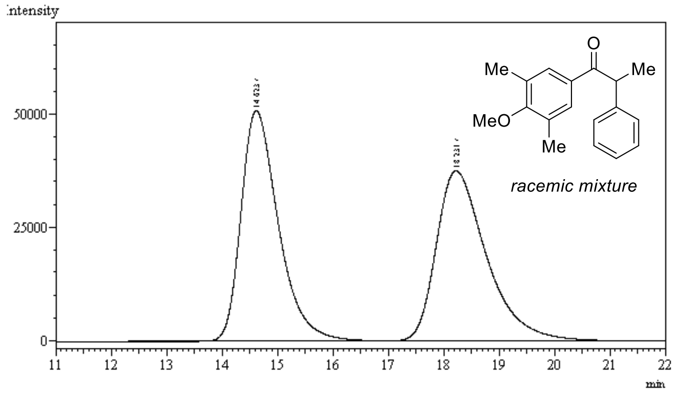

Peak#	Ret.Time	Area	Height	Conc.	Unit	Mark	ID#	Cmpd Name
1	14.623	2377361	50786	50.217				
2	18.231	2356835	37517	49.783				
Total		4734196	88303					

MEC-272, cellulose-3, Hexane 98:2, 0.8 mL/min, 254 nm

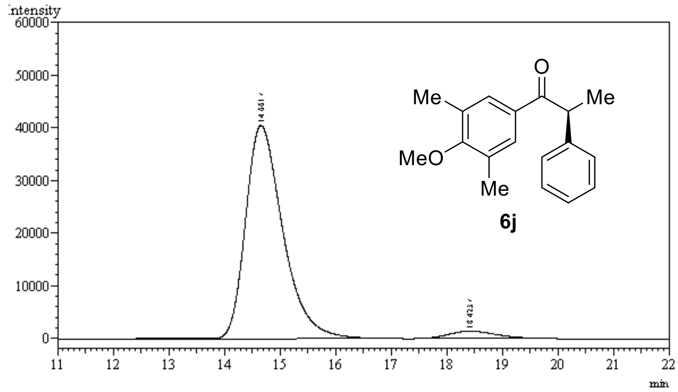

Peak#	Ret.Time	Area	Height	Conc.	Unit	Mark	ID#	Cmpd Name
1	14.661	1889060	40528	95.758				
2	18.423	83679	1401	4.242				
Total		1972739	41929					

MEC-250, AS-H, Hex/IPA 99:1, 1 mL/min, 245 nm

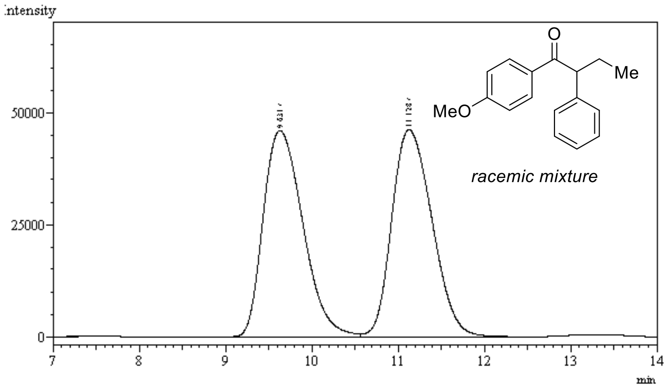

Peak#	Ret.Time	Area	Height	Conc.	Unit	Mark	ID#	Cmpd Name
1	9.631	1468158	45938	49.565				
2	11.128	1493899	46189	50.435		V		
Total		2962057	92127					

MANU472, AS-H, Hex/IPA 99:1, 1 mL/min, 245 nm

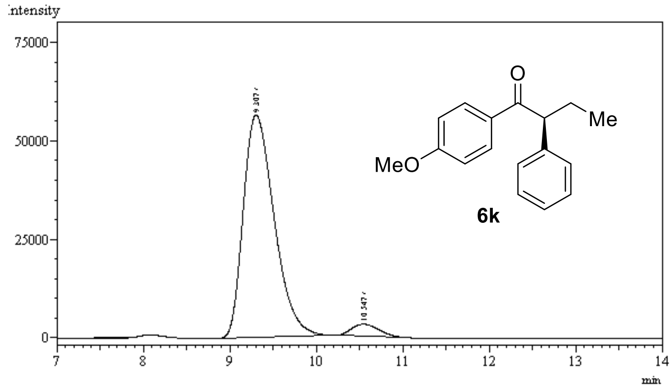

Peak#	Ret.Time	Area	Height	Conc.	Unit	Mark	ID#	Cmpd Name
1	9.307	1406997	56478	95.646				
2	10.547	64051	2982	4.354				
Total		1471048	59460					

MEC-229, cellulose-3, Hex/IPA 99.5:0.5, 0.8 mL/min, 254 nm

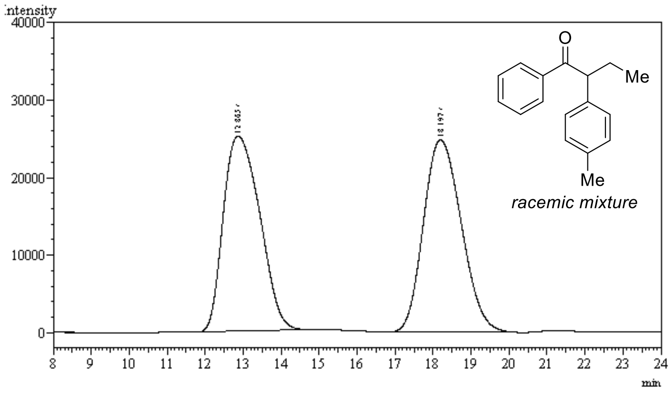

Peak#	Ret.Time	Area	Height	Conc.	Unit	Mark	ID#	Cmpd Name
1	12.865	1683675	25213	49.483				
2	18.197	1718853	24832	50.517				
Total		3402528	50045					

MANU456, cellulose-3, Hex/IPA 99.5:0.5, 0.8 mL/min, 254 nm

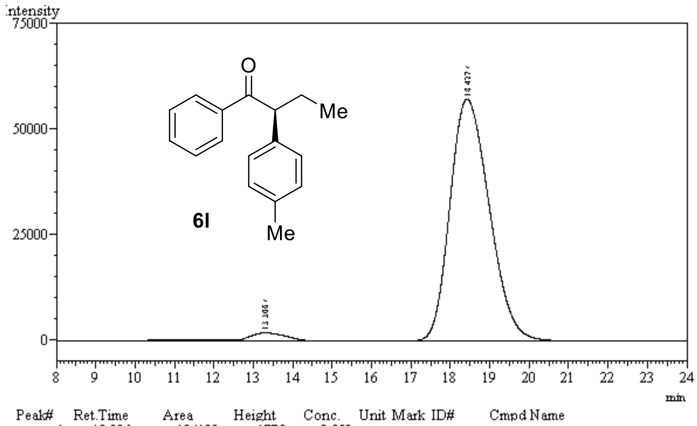

Peak#	Ret.Time	Area	Height	Conc.	Unit	Mark	ID#	Cmpd Name
1	13.306	104182	1775	2.558				
2	18.427	3968592	57217	97.442				
Total		4072774	58992					

MEC-254, Cellulose-1, Hex/IPA 98:2, 0.8 mL/min, 254 nm

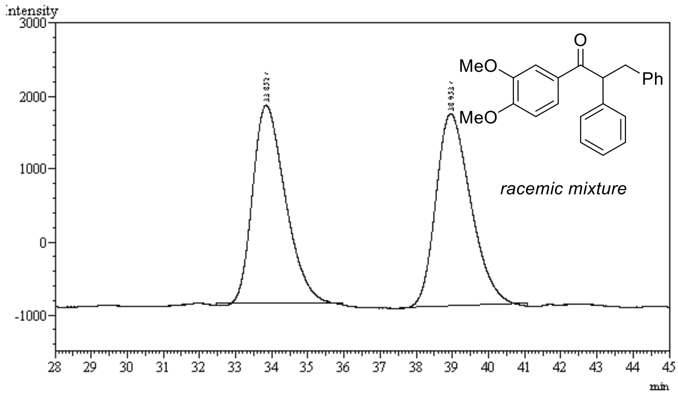

Peak#	Ret.Time	Area	Height	Conc.	Unit	Mark	ID#	Cmpd Name
1	33.852	169453	2712	49.025				
2	38.953	176192	2632	50.975				
Total		345645	5344					

MEC-257, Cellulose-1, Hex:IPA 98:2, 0.8 mL/min, 254 nm

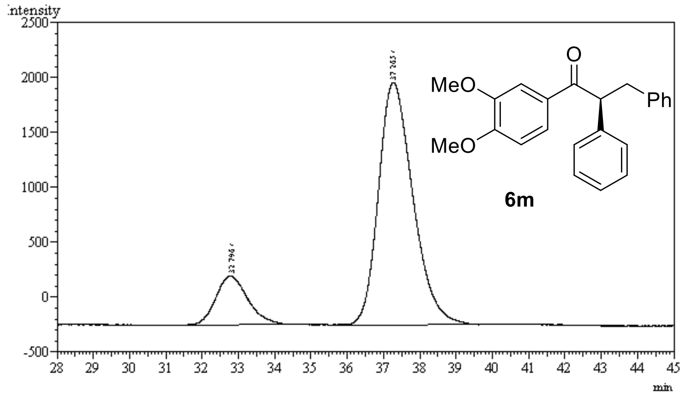

Peak#	Ret.Time	Area	Height	Conc.	Unit	Mark	ID#	Cmpd Name
1	32.796	26672	440	15.443				
2	37.265	146044	2207	84.557				
Total		172716	2647					

MEC-195, cellulose-3, Hex/IPA 98:2, 1 mL/min, 254 nm

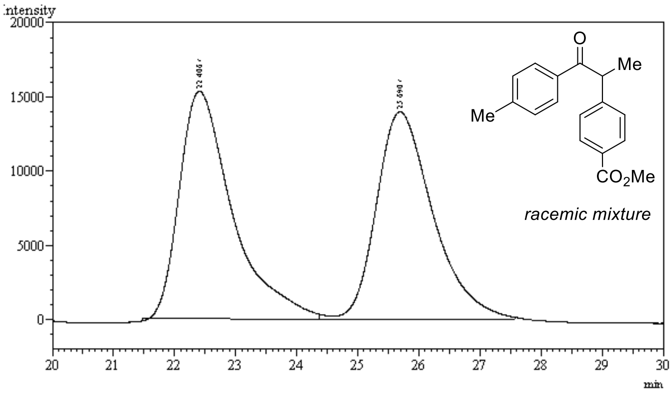

Peak#	Ret.Time	Area	Height	Conc.	Unit	Mark	ID#	Cmpd Name
1	22.406	923511	15295	50.469				
2	25.690	906347	13997	49.531				
Total		1829858	29292					

MANU447 cellulose-3, Hex/IPA 98:2, 1 mL/min, 254 nm

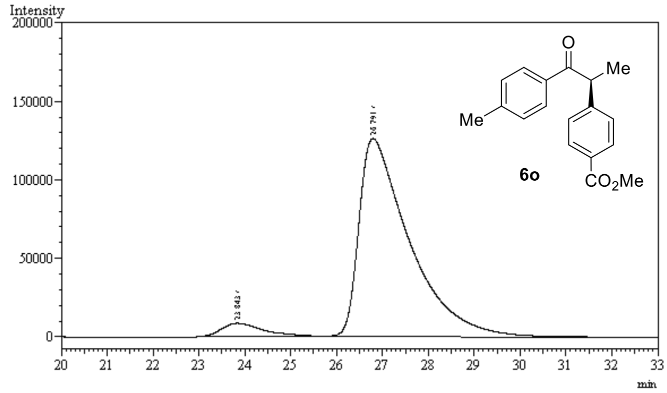

Peak#	Ret.Time	Area	Height	Conc.	Unit	Mark	ID#	Cmpd Name
1	23.843	482537	8105	4.782				
2	26.791	9608028	125991	95.218				
Total		10090565	134096					

MEC-190, racemic, cellulose-3, Hex/IPA 98:2, 0.8 mL/min, 254 nm

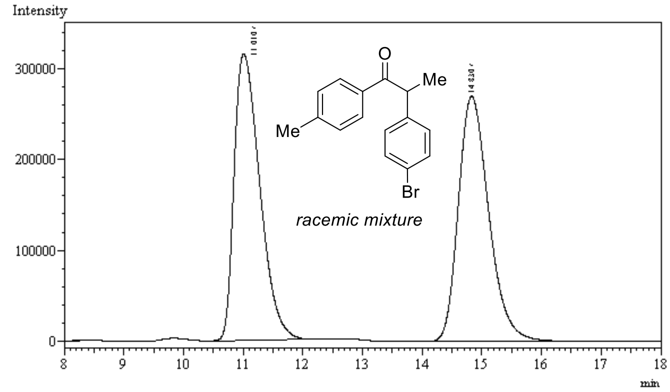

Peak#	Ret.Time	Area	Height	Conc.	Unit	Mark	ID#	Cmpd Name
1	11.010	9430411	315134	49.700				
2	14.830	9544342	269368	50.300				
Total		18974753	584502					

MANU454 pure, cellulose-3, Hex/IPA 98:2, 0.8 mL/min, 254 nm

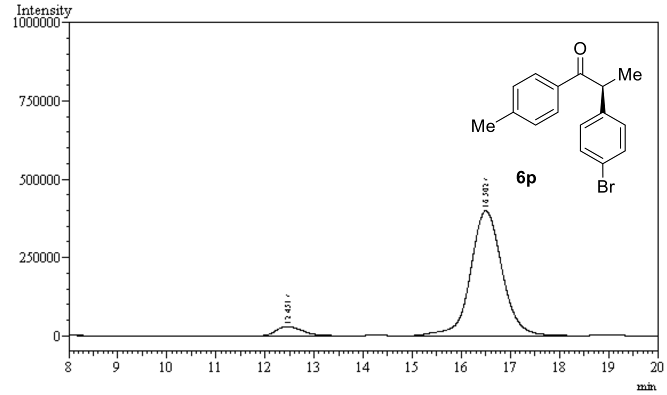

Peak#	Ret.Time	Area	Height	Conc.	Unit	Mark	ID#	Cmpd Name
1	12.451	1053154	29245	5.595				
2	16.502	17769786	398970	94.405				
Total		18822940	428215					

MEC-191, racemic, AS-H, Hex/IPA 99:1, 1 mL/min, 254 nm

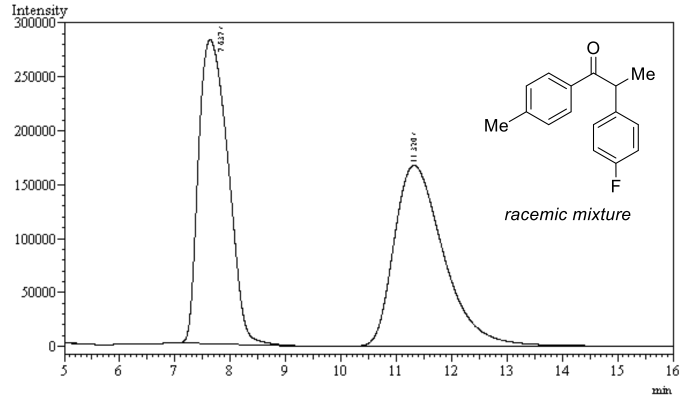

Peak#	Ret.Time	Area	Height	Conc.	Unit	Mark	ID#	Cmpd Name
1	7.637	10420751	281751	49.993				
2	11.320	10423688	167637	50.007				
Total		20844439	449388					

MEC-293, AS-H, Hexane 99:1, 1 mL/min, 254 nm

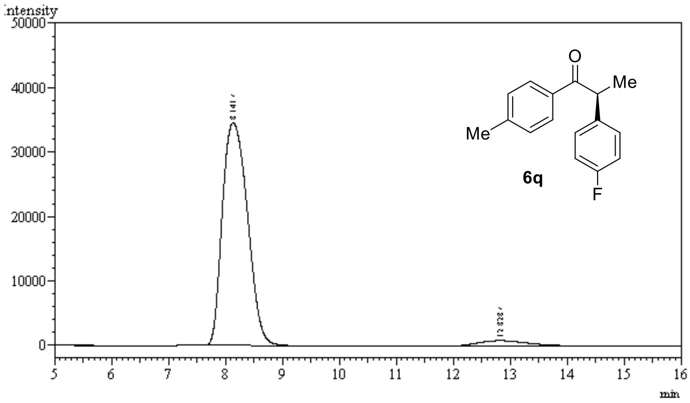

Peak#	Ret.Time	Area	Height	Conc.	Unit	Mark	ID#	Cmpd Name
1	8.141	1060873	34546	95.523				
2	12.828	49717	830	4.477				
Total		1110590	35376					

MEC-311, cellulose-3, Hex:IPA 98:2, 0.8 mL/min, 254 nm

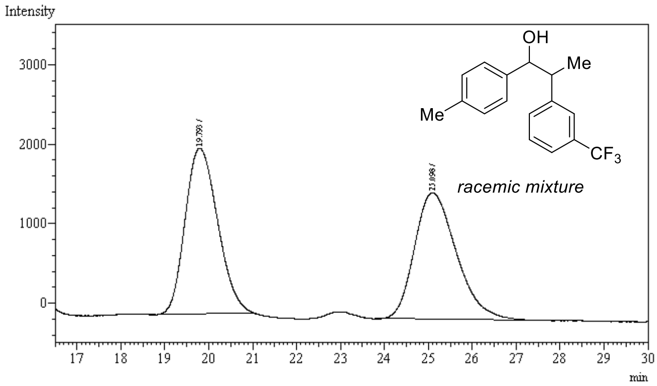

Peak#	Ret.Time	Area	Height	Conc.	Area%
1	19.793	106895	2080	50.336	50.3364
2	25.098	105466	1593	49.664	49.6636
Total		212361	3673		100.0000

MEC-312, cellulose-3, Hex:IPA 98:2, 0.8 mL/min, 254 nm

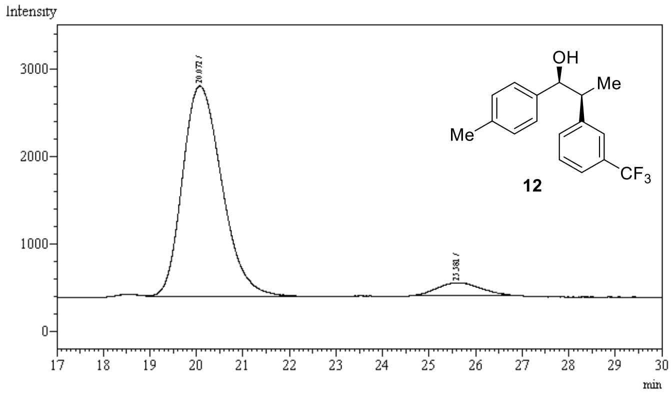

Peak#	Ret.Time	Area	Height	Conc.	Area%
1	20.072	141553	2404	93.547	93.5469
2	25.581	9765	148	6.453	6.4531
Total		151318	2552		100.0000

MEC-300, cellulose-3, Hex:IPA 99:1, 0.5 mL/min, 254 nm

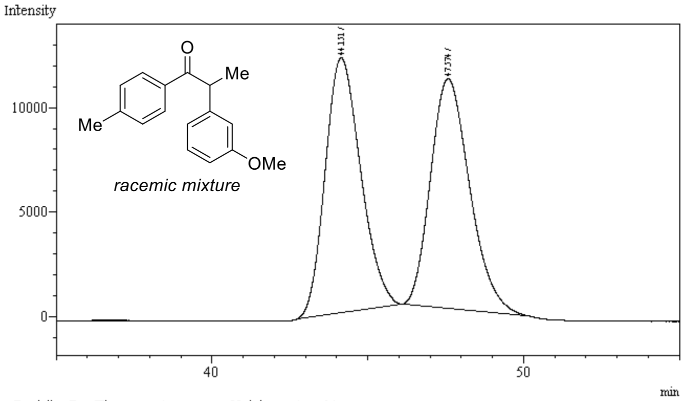

Peak#	Ret.Time	Area	Height	Area%
1	44.151	965383	12187	50.2373
2	47.574	956262	10962	49.7627
Total		1921645	23149	100.0000

MEC-301, cellulose-3, Hex:IPA 99:1, 0.5 mL/min, 254 nm

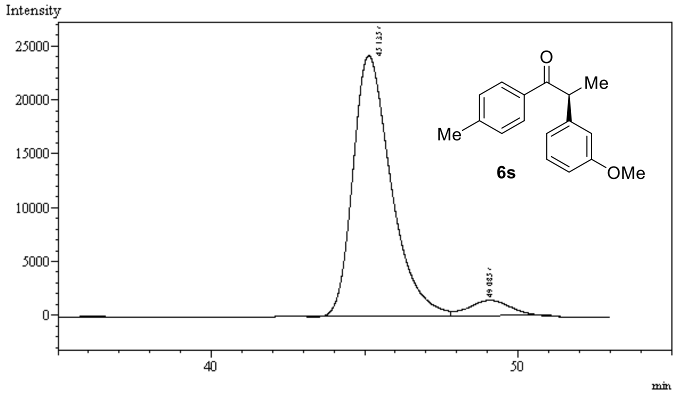

Peak#	Ret.Time	Area	Height	Conc.	Area%
1	45.135	2077099	24138	94.103	94.1028
2	49.085	130166	1410	5.897	5.8972
Total		2207265	25548		100.0000

MEC-194, racemic, cellulose-3, Hexane 98:2, 1 mL/min, 254 nm

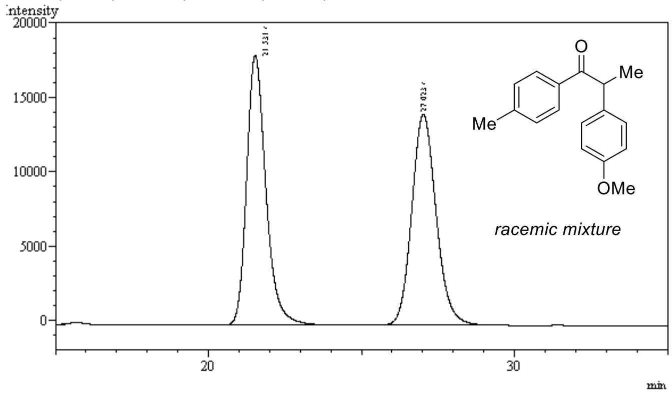

Peak#	Ret.Time	Area	Height	Conc.	Unit	Mark	ID#	Cmpd Name
1	21.531	787020	18113	50.293				
2	27.023	777850	14134	49.707				
Total		1564870	32247					

MEC-262, cellulose-3, Hexane 98:2, 1 mL/min, 254 nm

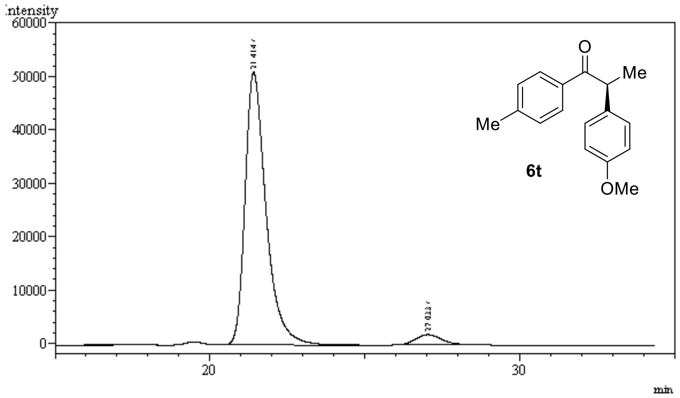

Peak#	Ret.Time	Area	Height	Conc.	Unit	Mark	ID#	Cmpd Name
1	21.414	2406042	51021	95.370				
2	27.033	116813	1943	4.630				
Total		2522855	52964					

MEC-192, racemic, cellulose-3, Hex/IPA 99:1, 0.8 mL/min, 254 nm

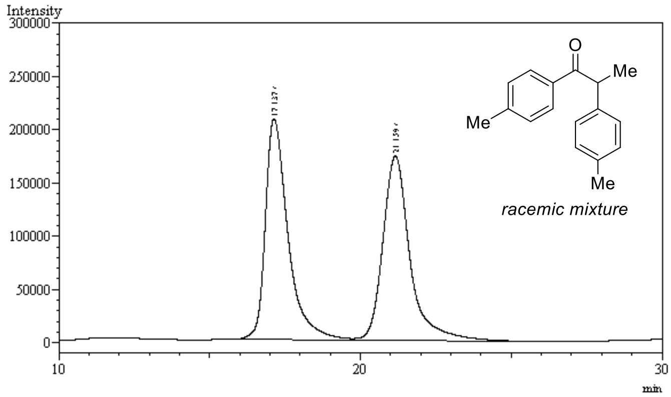

Peak#	Ret.Time	Area	Height	Conc.	Unit	Mark	ID#	Cmpd Name
1	17.137	10857904	207598	49.909				
2	21.159	10897282	173318	50.091		V		
Total		21755186	380916					

MANU451, cellulose-3, Hex/IPA 99:1, 0.8 mL/min, 254 nm

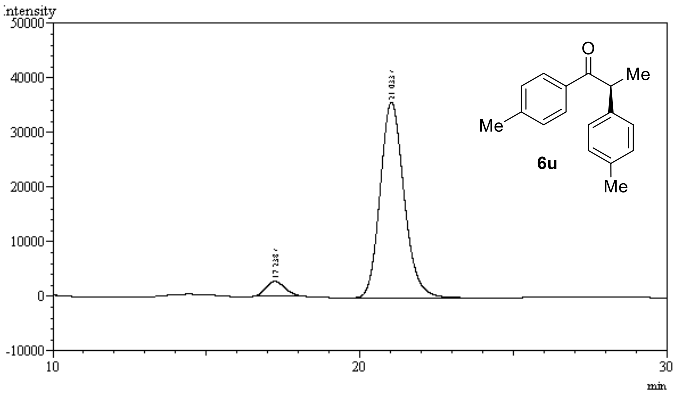

Peak#	Ret.Time	Area	Height	Conc.	Unit	Mark	ID#	Cmpd Name
1	17.238	113853	2664	5.540				
2	21.033	1941082	35849	94.460				
Total		2054935	38513					

MEC-193, racemic, cellulose-3, Hexane 98:2, 0.5 mL/min, 254 nm

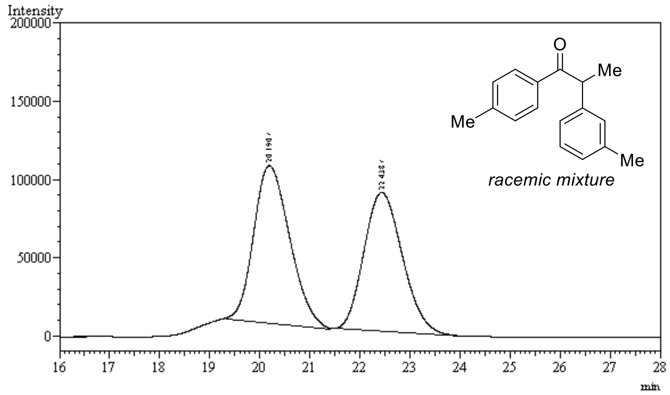

Peak#	Ret.Time	Area	Height	Conc.	Unit	Mark	ID#	Cmpd Name
1	20.190	5061677	100842	51.543				
2	22.438	4758578	88935	48.457				
Total		9820255	189777					

MEC-266, cellulose-3, Hexane 98:2, 0.5 mL/min, 254 nm

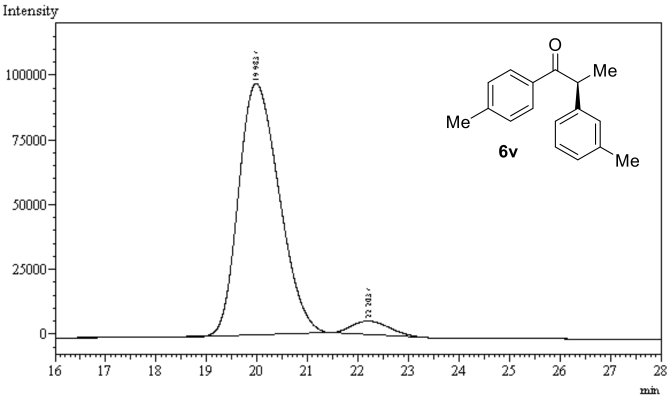

Peak#	Ret.Time	Area	Height	Conc.	Unit	Mark	ID#	Cmpd Name
1	19.983	5398726	96860	95.439				
2	22.203	257974	5119	4.561				
Total		5656700	101979					

MEC-197, racemic, cellulose-3, Hexane 99:1, 0.8 mL/min, 254 nm

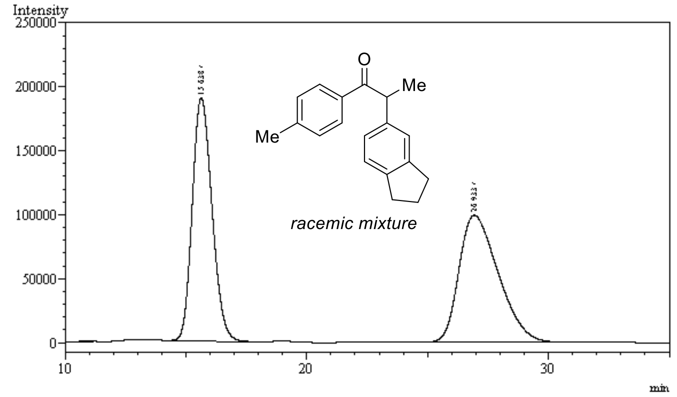

Peak#	Ret.Time	Area	Height	Conc.	Unit	Mark	ID#	Cmpd Name
1	15.638	10820556	190496	49.194				
2	26.933	11174977	99213	50.806				
Total		21995533	289709					

MEC-271, cellulose-3, Hexane 99:1, 0.8 mL/min, 254 nm

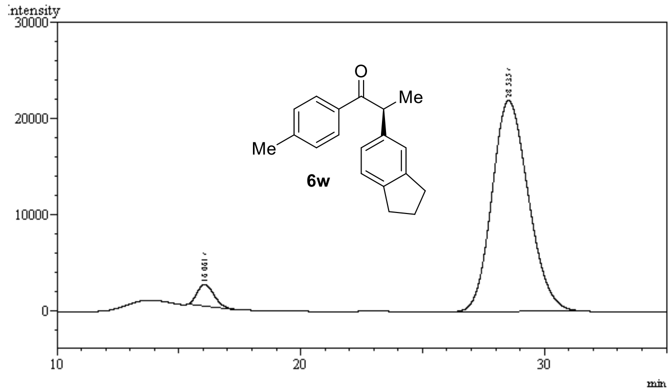

Peak#	Ret.Time	Area	Height	Conc.	Unit	Mark	ID#	Cmpd Name
1	16.061	110205	2270	4.592				
2	28.535	2289822	22006	95.408				
Total		2400027	24276					

MEC-287, cellulose-3, Hexane 98:2, 0.8 mL/min, 254 nm

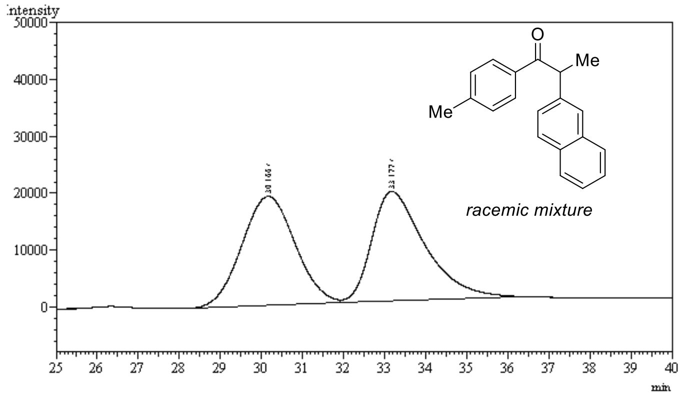

Peak#	Ret.Time	Area	Height	Conc.	Unit	Mark	ID#	Cmpd Name
1	30.166	1729641	19183	49.963				
2	33.177	1732235	19285	50.037		V		
Total		3461876	38468					

MEC-290, cellulose-3, Hexane 98:2, 0.8 mL/min, 254 nm

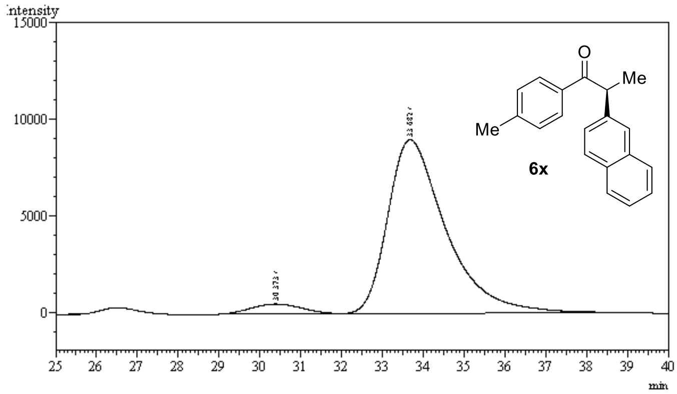

Peak#	Ret.Time	Area	Height	Conc.	Unit	Mark	ID#	Cmpd Name
1	30.373	39021	482	4.187				
2	33.682	892928	9023	95.813				
Total		931949	9505					

MEC-230, AS-H, Hex/IPA 98:2, 1 mL/min, 254 nm

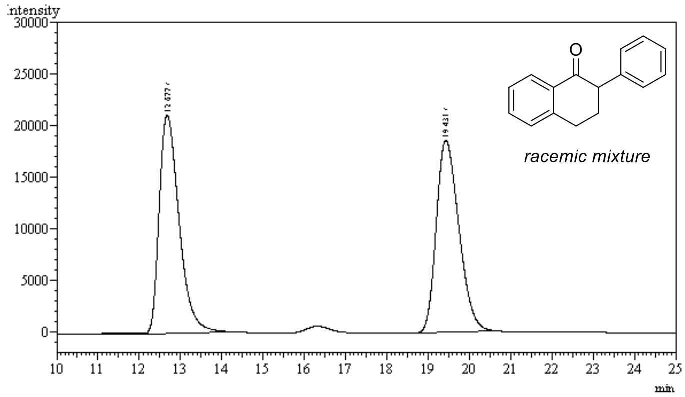

Peak#	Ret.Time	Area	Height	Conc.	Unit	Mark	ID#	Cmpd Name
1	12.677	707096	21160	49.855				
2	19.431	711207	18603	50.145				
Total		1418303	39763					

MANU476, AS-H, Hex:IPA 98:2, 1 mL/min, 254 nm

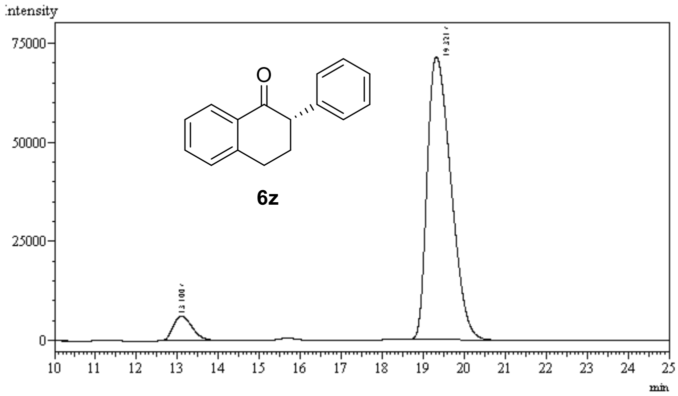

Peak#	Ret.Time	Area	Height	Conc.	Unit	Mark	ID#	Cmpd Name
1	13.100	189845	6016	6.195				
2	19.321	2874847	71429	93.805				
Total		3064692	77445					

MEC-387, AS-H, Hex: IPA 98:2, 0.8 mL/min, 254 nm

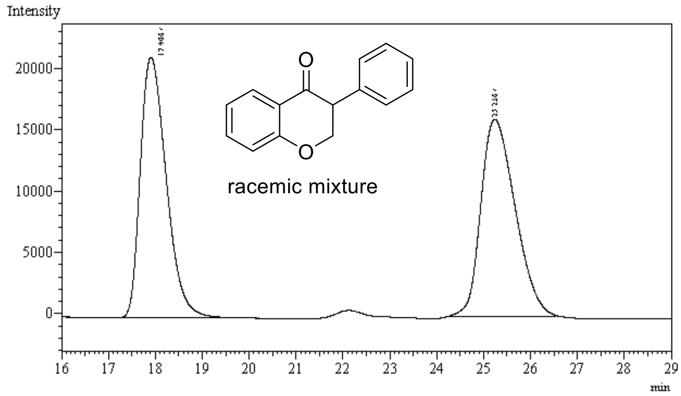

Peak#	Ret.Time	Area	Height	Conc.
1	17.906	812676	21242	49.906
2	25.236	815739	16065	50.094
Total		1628415	37307	

MANU515, AS-H, Hex: IPA 98:2, 0.8 mL/min, 254 nm

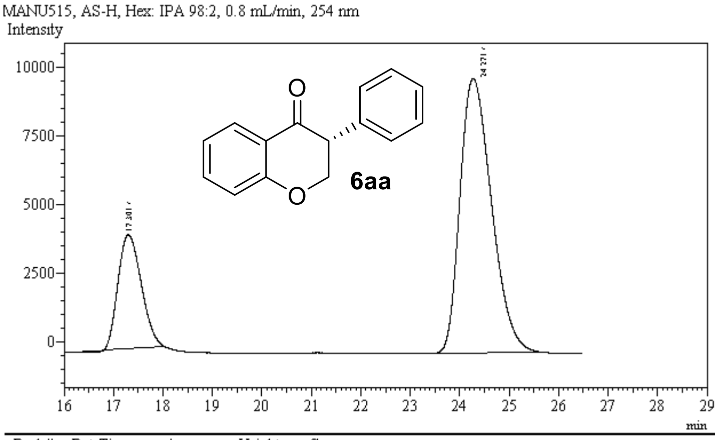

Peak#	Ret.Time	Area	Height	Conc.
1	17.301	136572	4159	23.563
2	24.271	443034	10021	76.437
Total		579606	14180	

MEC110F5, Cellulose-3, Hex:IPA 98:2, 0.8 mL/min, 220 nm

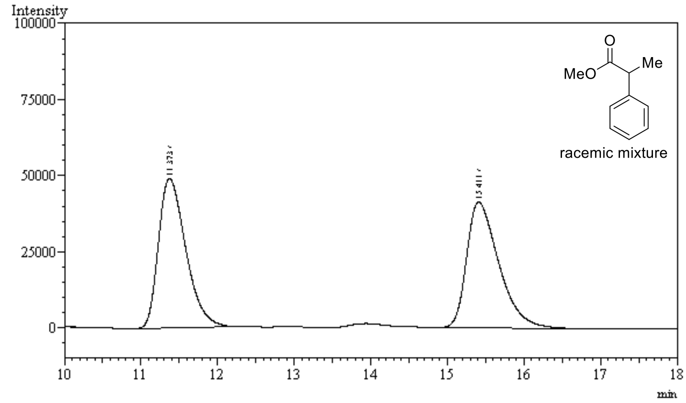

Peak#	Ret.Time	Area	Height	Conc.	Unit	Mark	ID#	Cmpd Name
1	11.373	1219361	49048	50.126				
2	15.411	1213241	41361	49.874				
Total		2432602	90409					

MANU527, Cellulose-3, Hex:IPA 98:2, 0.8 mL/min, 240 nm

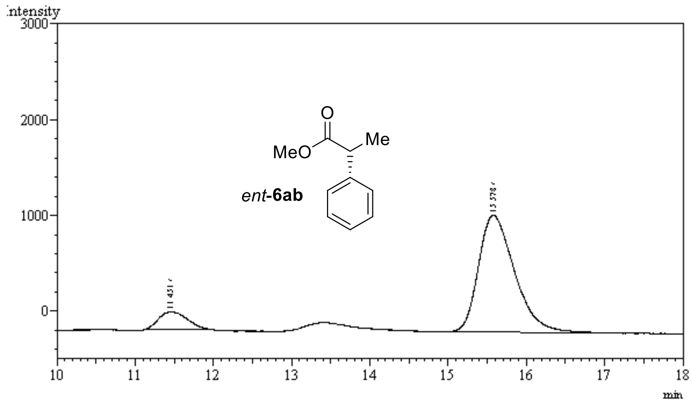

Peak#	Ret.Time	Area	Height	Conc.	Unit	Mark	ID#	Cmpd Name
1	11.451	4580	181	10.524				
2	15.578	38936	1219	89.476				
Total		43516	1400					

MANU512, AS-H, Hex 100%, 0.8 mL/min, 254 nm

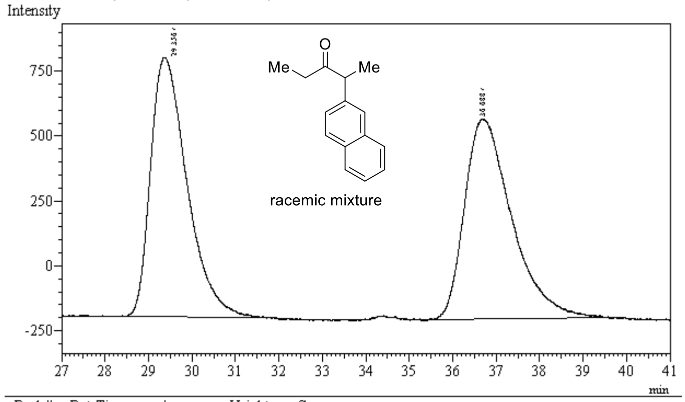

Peak#	Ret.Time	Area	Height	Conc.
1	29.356	58350	1000	49.873
2	36.688	58648	774	50.127
Total		116998	1774	

MANU520, AS-H, Hex 100%, 0.8 mL/min, 225 nm

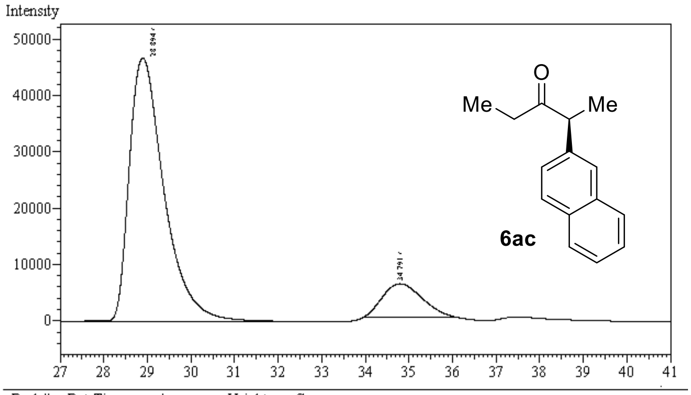

Peak#	Ret.Time	Area	Height	Conc.
1	28.894	2610071	46865	87.215
2	34.791	382610	5983	12.785
Total		2992681	52848	

MEC-256, AS-H, Hexane 100 %, 1 mL/min, 215 nm

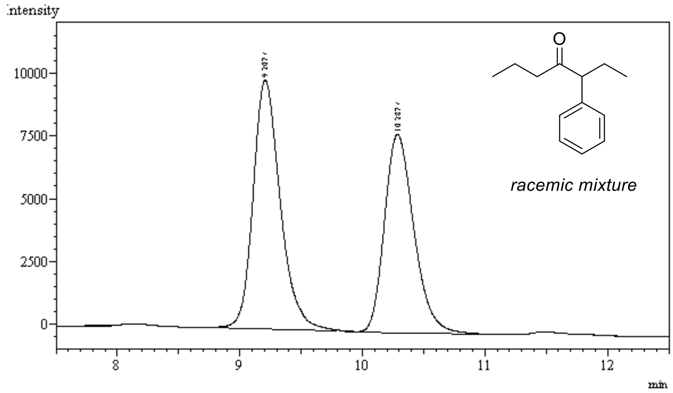

Peak#	Ret.Time	Area	Height	Conc.	Unit	Mark	ID#	Cmpd Name
1	9.207	151985	9877	53.628				
2	10.287	131421	7876	46.372				
Total		283406	17753					

MANU477, AS-H, Hexane 100, 1 mL/min, 215 nm

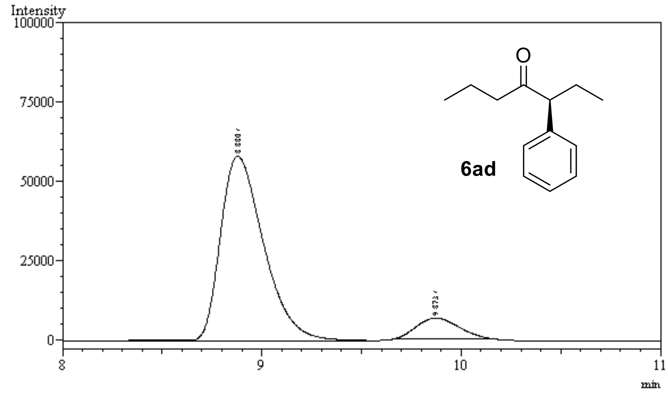

Peak#	Ret.Time	Area	Height	Conc.	Unit	Mark	ID#	Cmpd Name
1	8.880	878657	58340	89.797				
2	9.873	99831	6655	10.203				
Total		978488	64995					

MEC-397, Cellulose-3, Hex:IPA 99.5:0.5, 0.5 mL/min, 215 nm

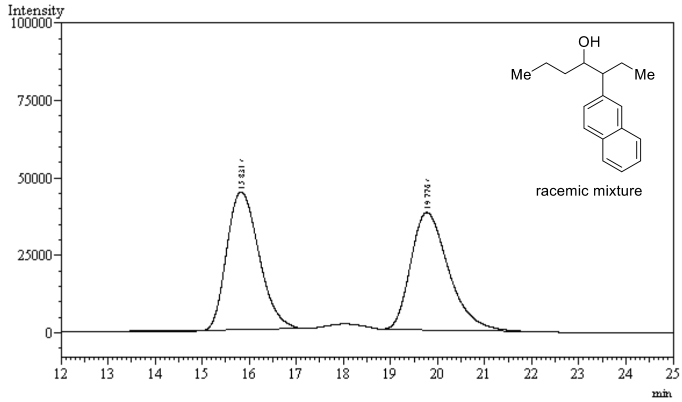

Peak#	Ret.Time	Area	Height	Conc.	Unit	Mark	ID#	Cmpd Name
1	15.831	2135898	44654	50.104				
2	19.776	2127054	37954	49.896				
Total		4262952	82608					

MEC-397, Cellulose-3, Hex:IPA 99.5:0.5, 0.5 mL/min, 215 nm

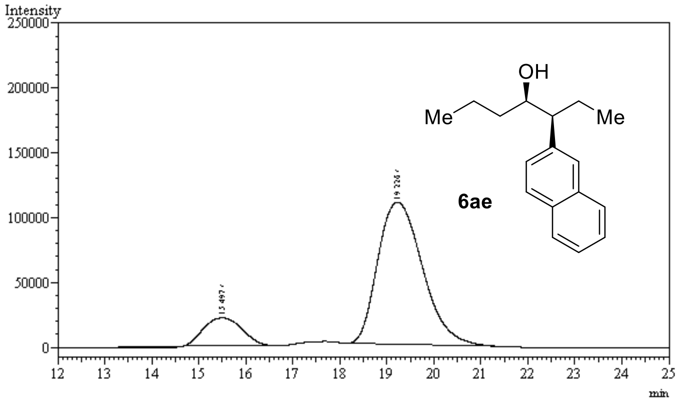

Peak#	Ret.Time	Area	Height	Conc.	Unit	Mark	ID#	Cmpd Name
1	15.497	1203929	21359	14.160				
2	19.226	7298427	109642	85.840				
Total		8502356	131001					

MEC-396, Cellulose-3, Hex:IPA 99.5:0.5, 0.5 mL/min, 215 nm

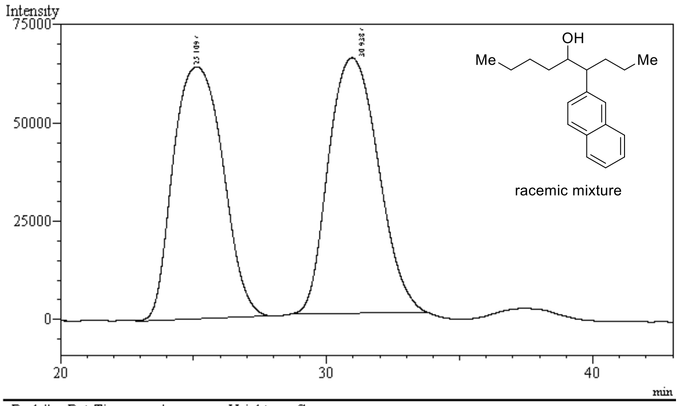

Peak#	Ret.Time	Area	Height	Conc.
1	25.109	8380454	64219	49.700
2	30.938	8481751	64954	50.300
Total		16862205	129173	

MEC-395, Cellulose-3, Hex:IPA 99.5:0.5, 0.5 mL/min, 215 nm

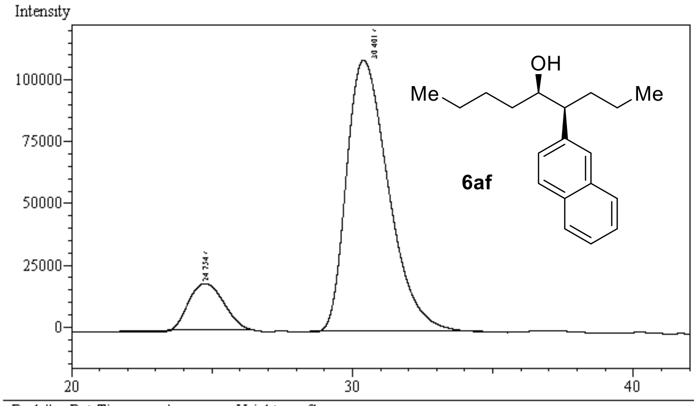

Peak#	Ret.Time	Area	Height	Conc.
1	24.754	1609249	18683	12.359
2	30.401	11411408	109798	87.641
Total		13020657	128481	

NMR spectra

^{1}H NMR (300 MHz, $CDCl_3$)

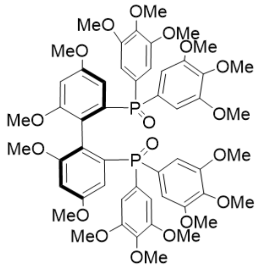

3x

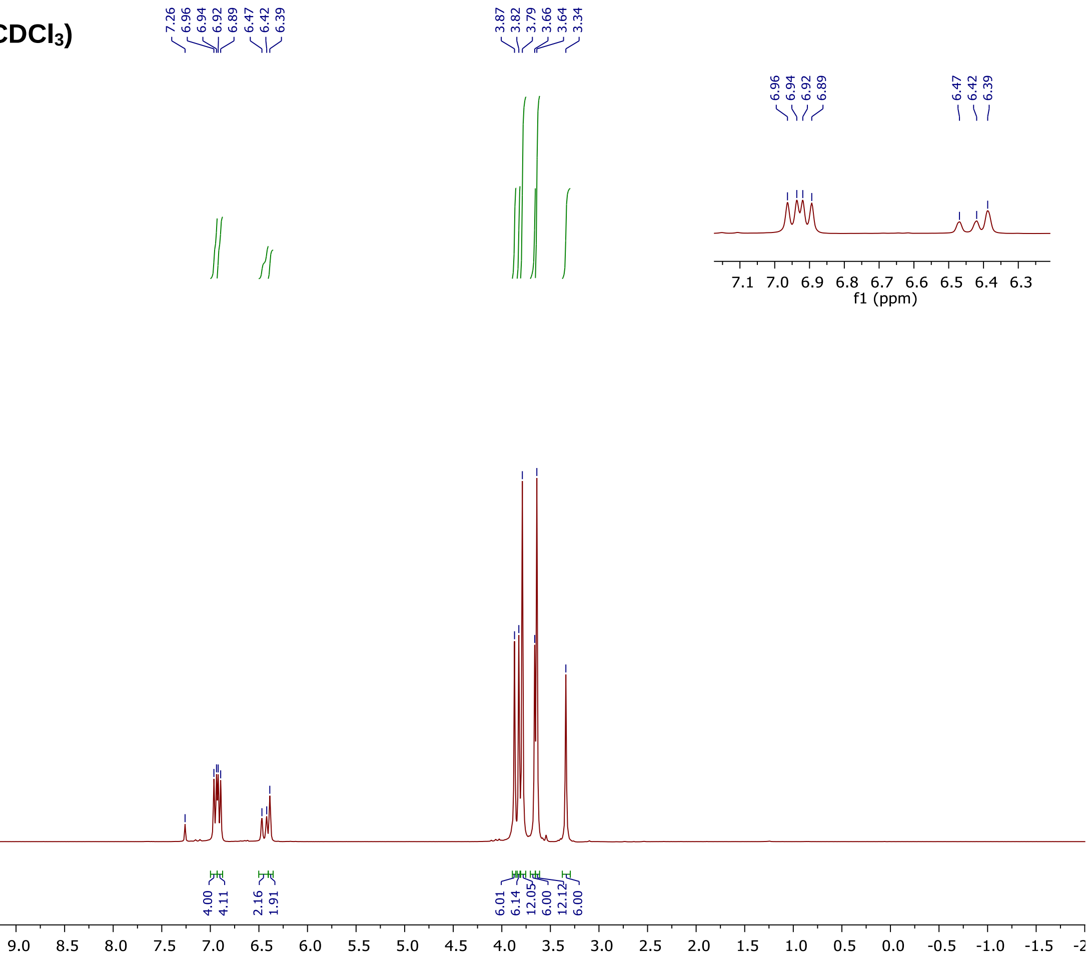

^{13}C NMR (75 MHz, $CDCl_3$)

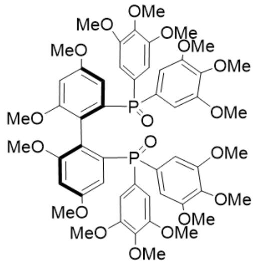

3x

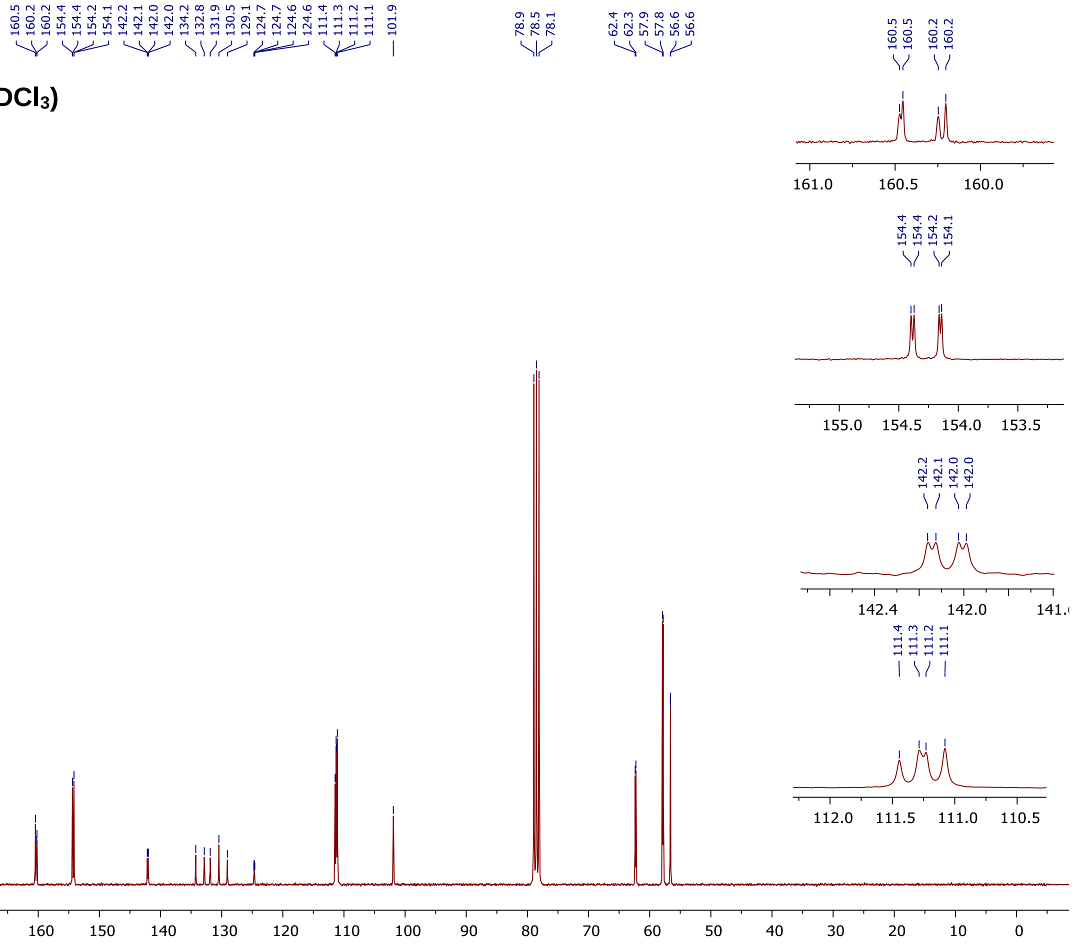

^{31}P NMR (121 MHz, $CDCl_3$)

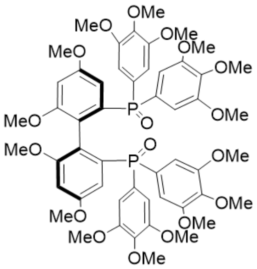

3x

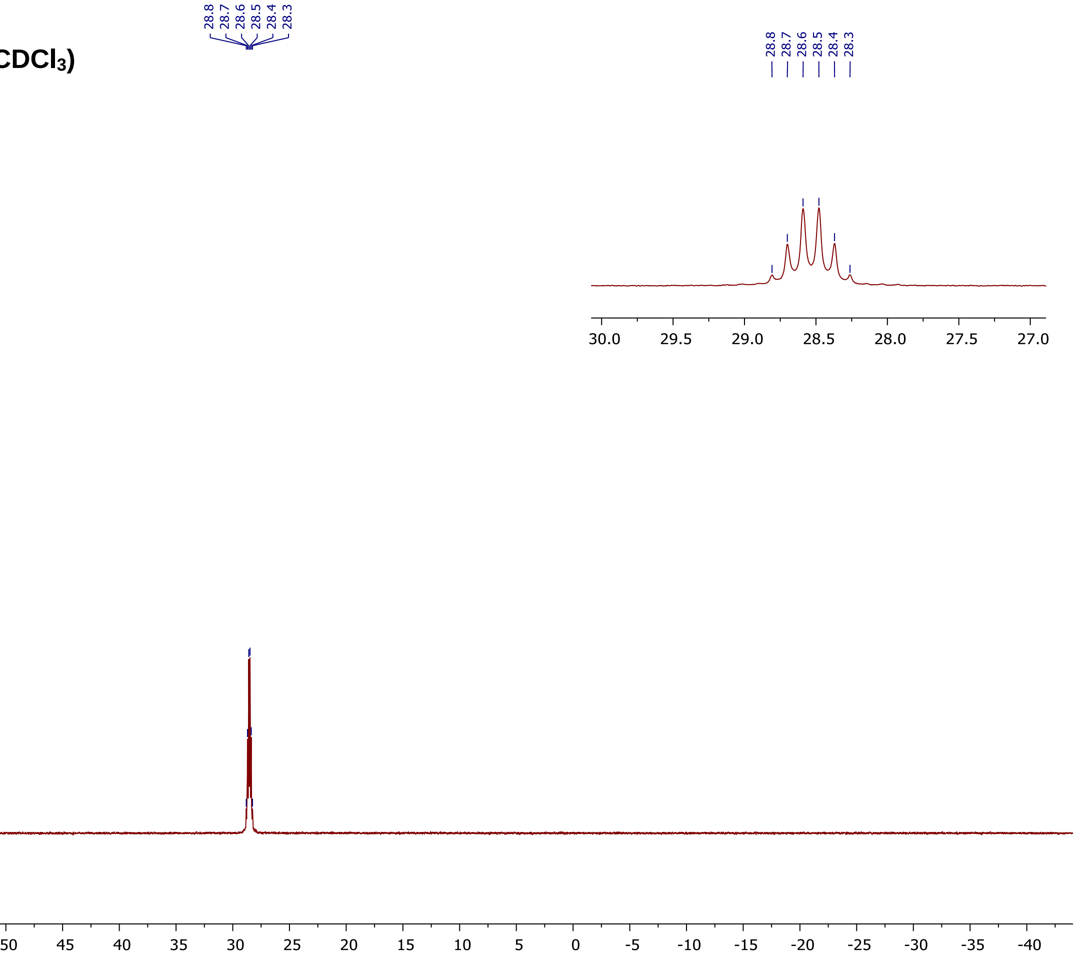

^{1}H NMR (300 MHz, $CDCl_3$)

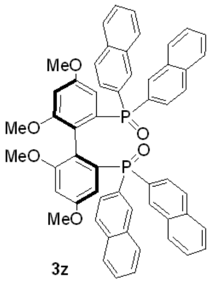

3z

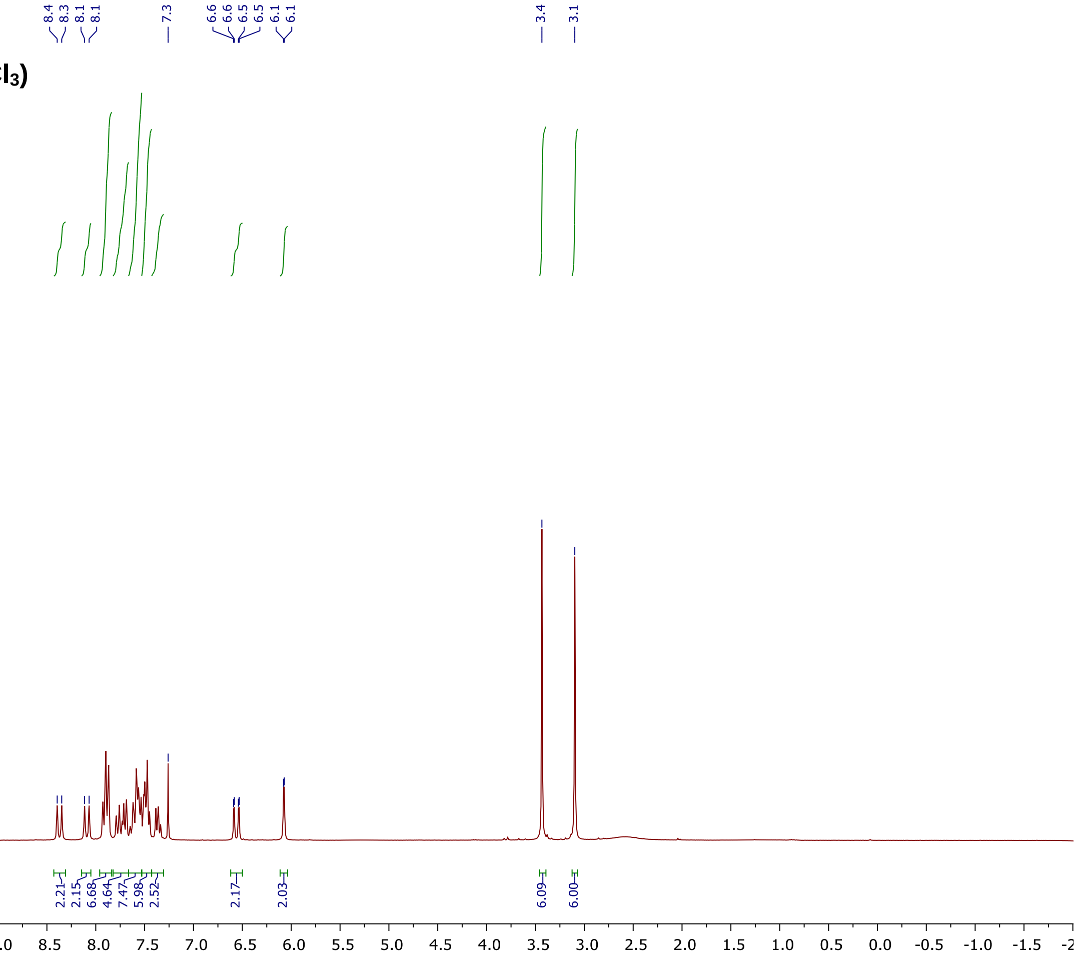

^{13}C NMR (75 MHz, $CDCl_3$)

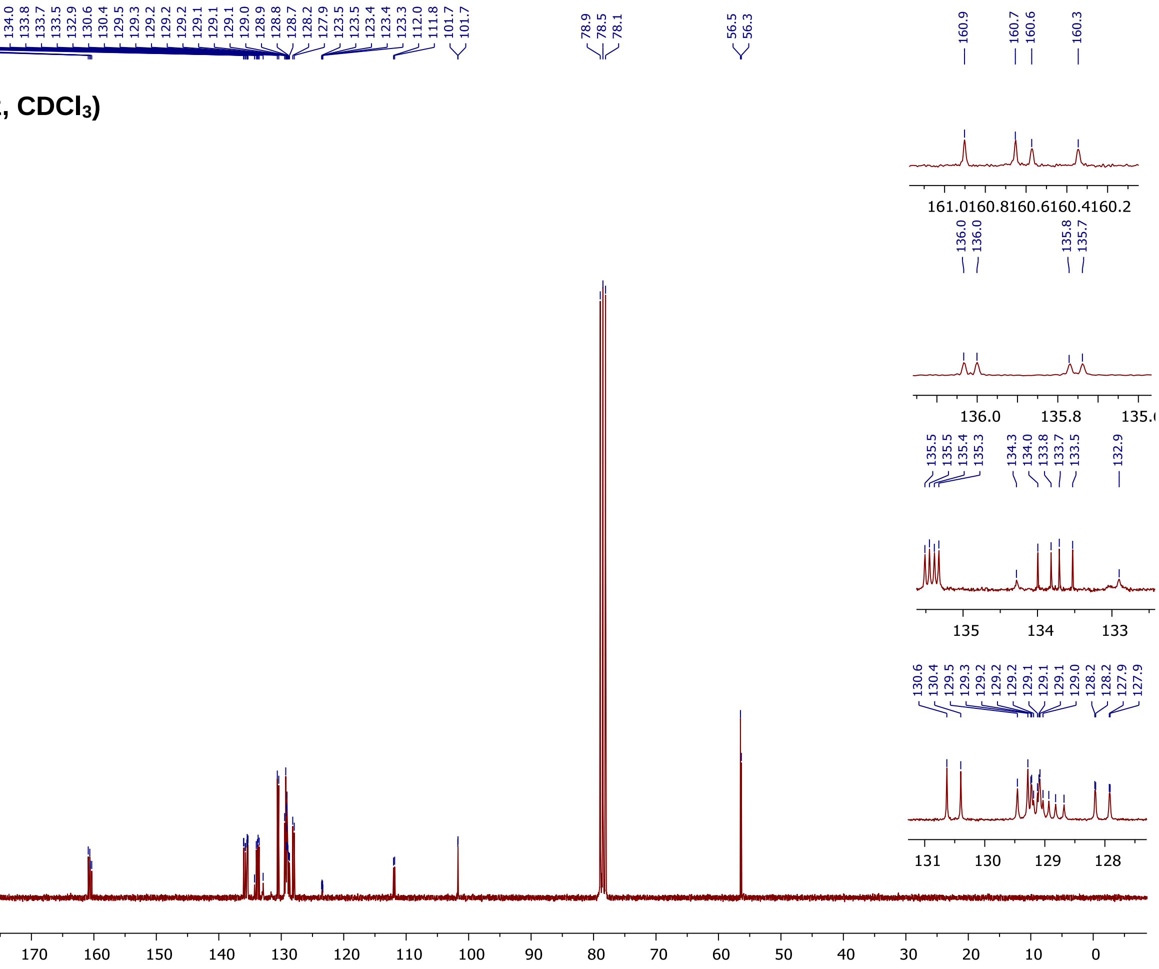

3z

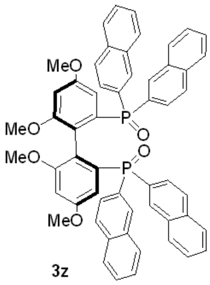

^{31}P NMR (121 MHz, $CDCl_3$)

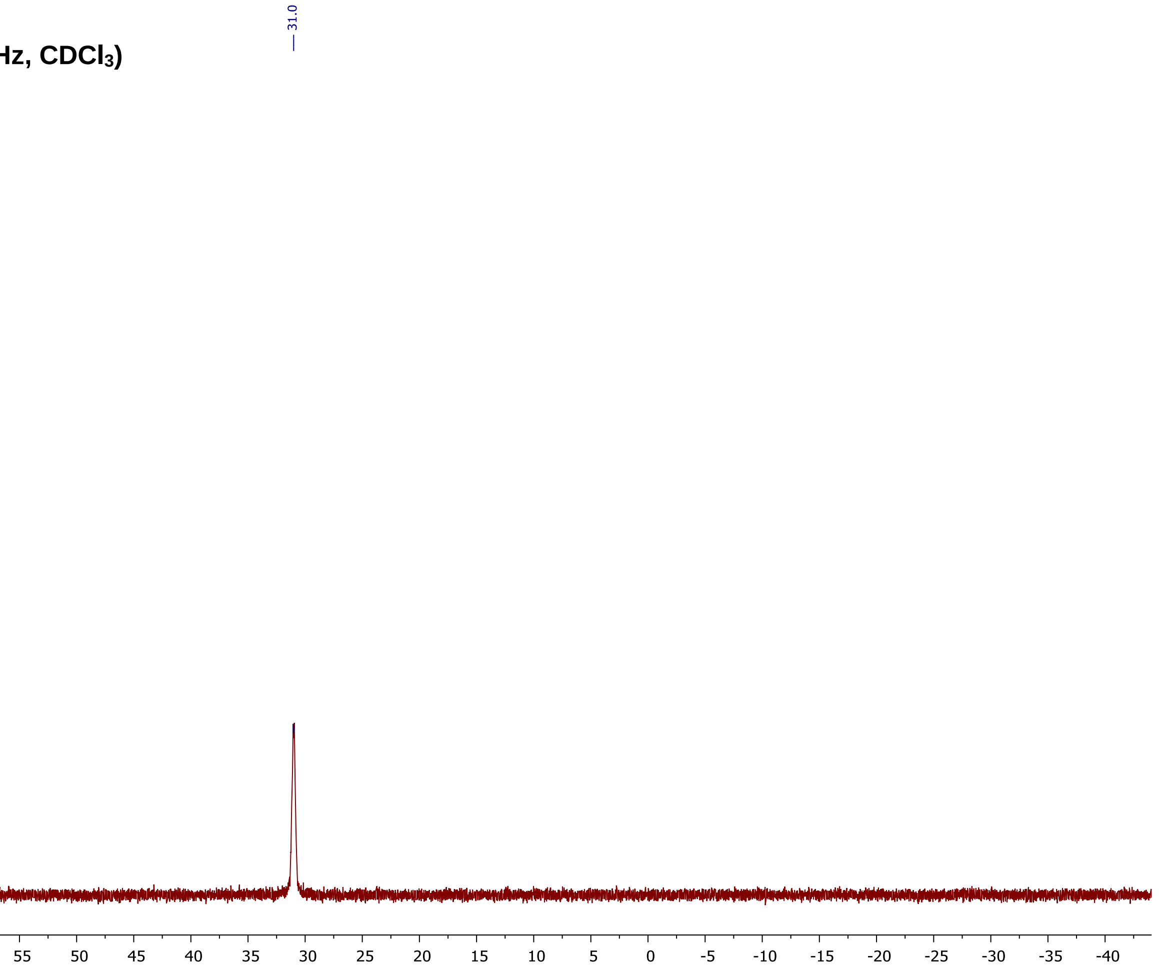

3z

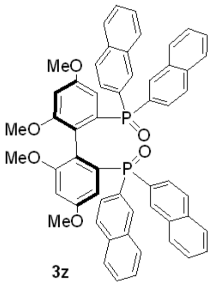

^{1}H NMR (300 MHz, $CDCl_3$)

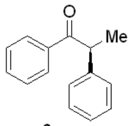

6a

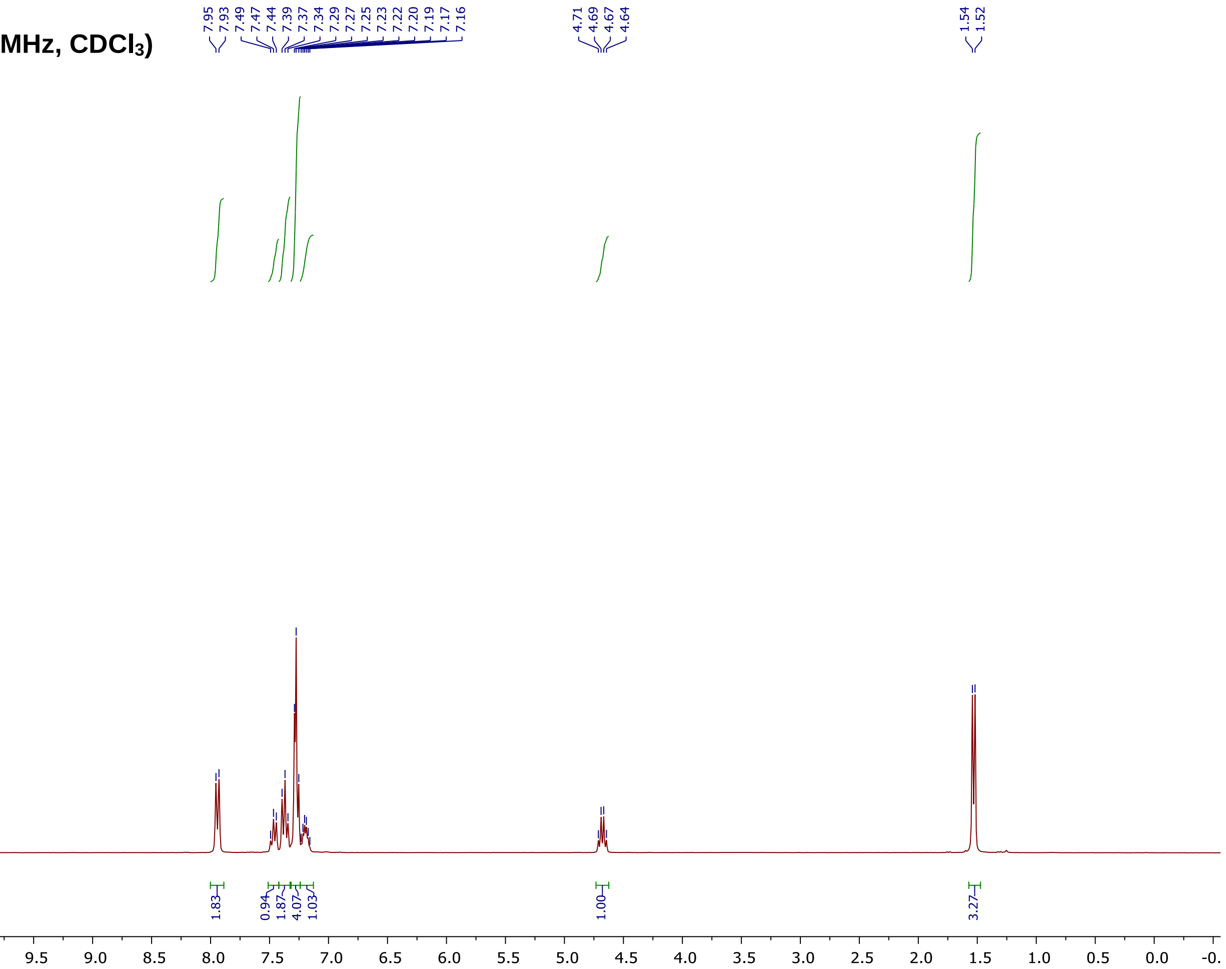

^{13}C NMR (75 MHz, $CDCl_3$)

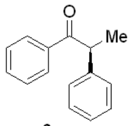

6a

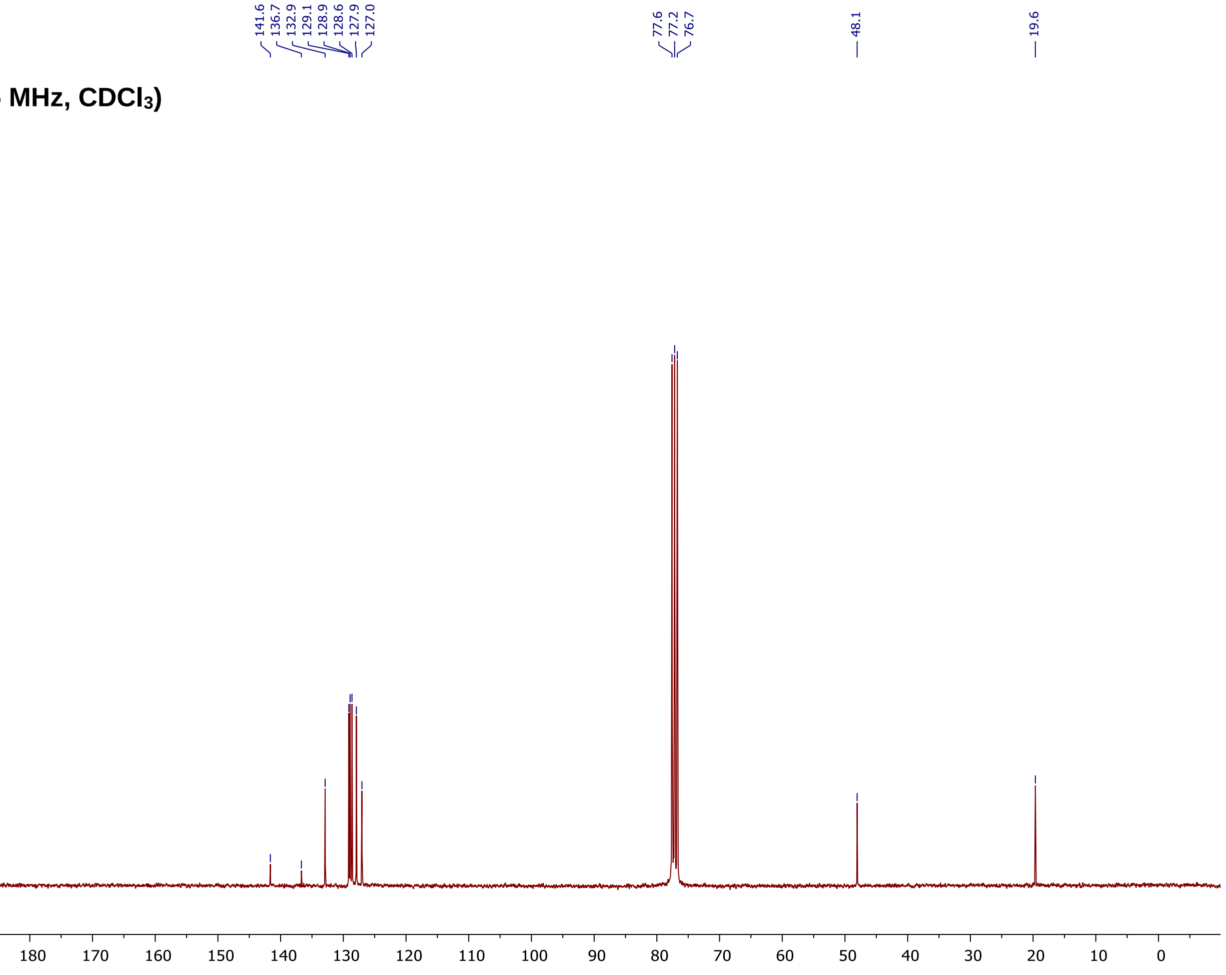

^{1}H NMR (200 MHz, $CDCl_3$)

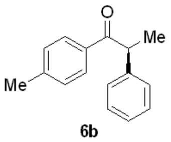

6b

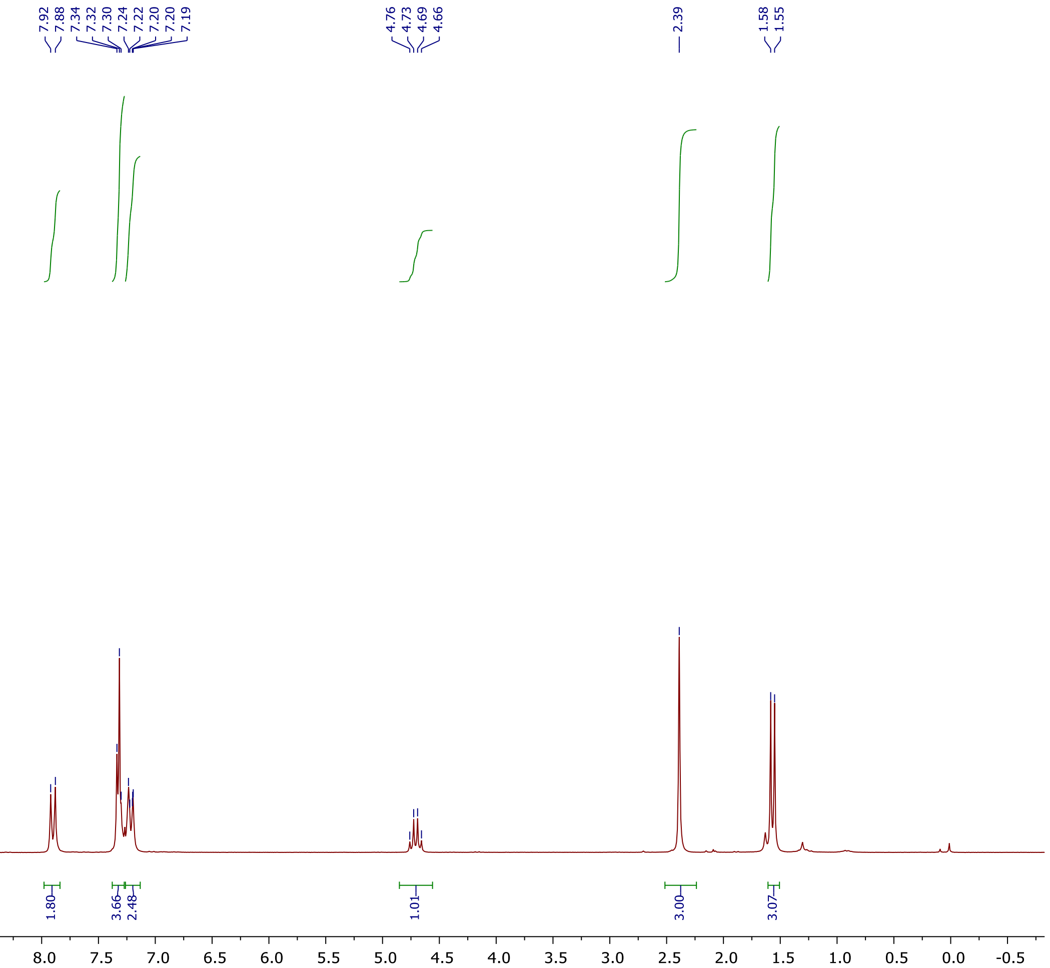

^{13}C NMR (50 MHz, $CDCl_3$)

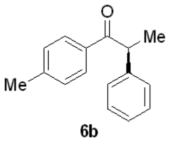

6b

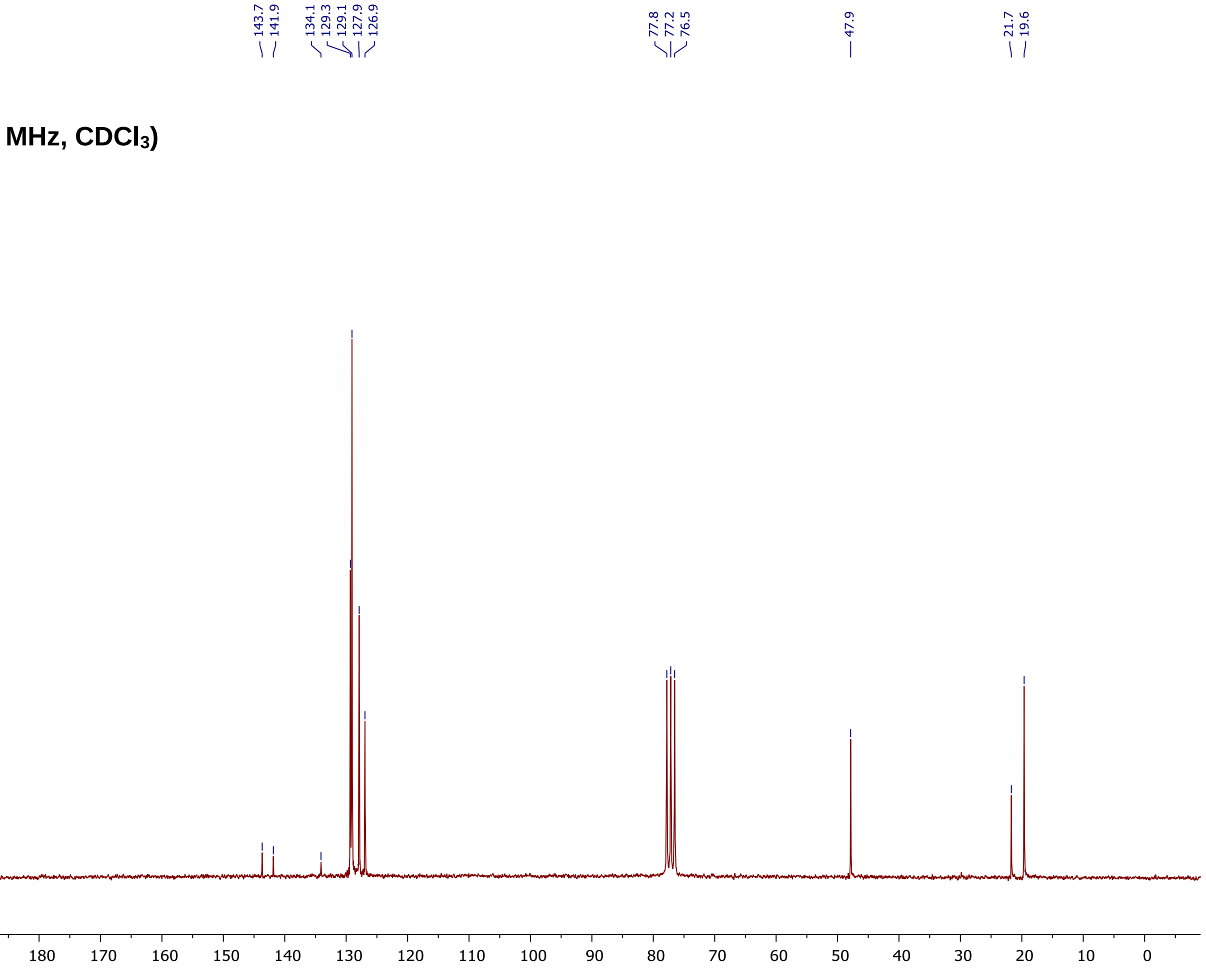

^{1}H NMR (200 MHz, $CDCl_3$)

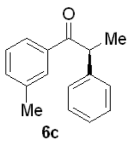

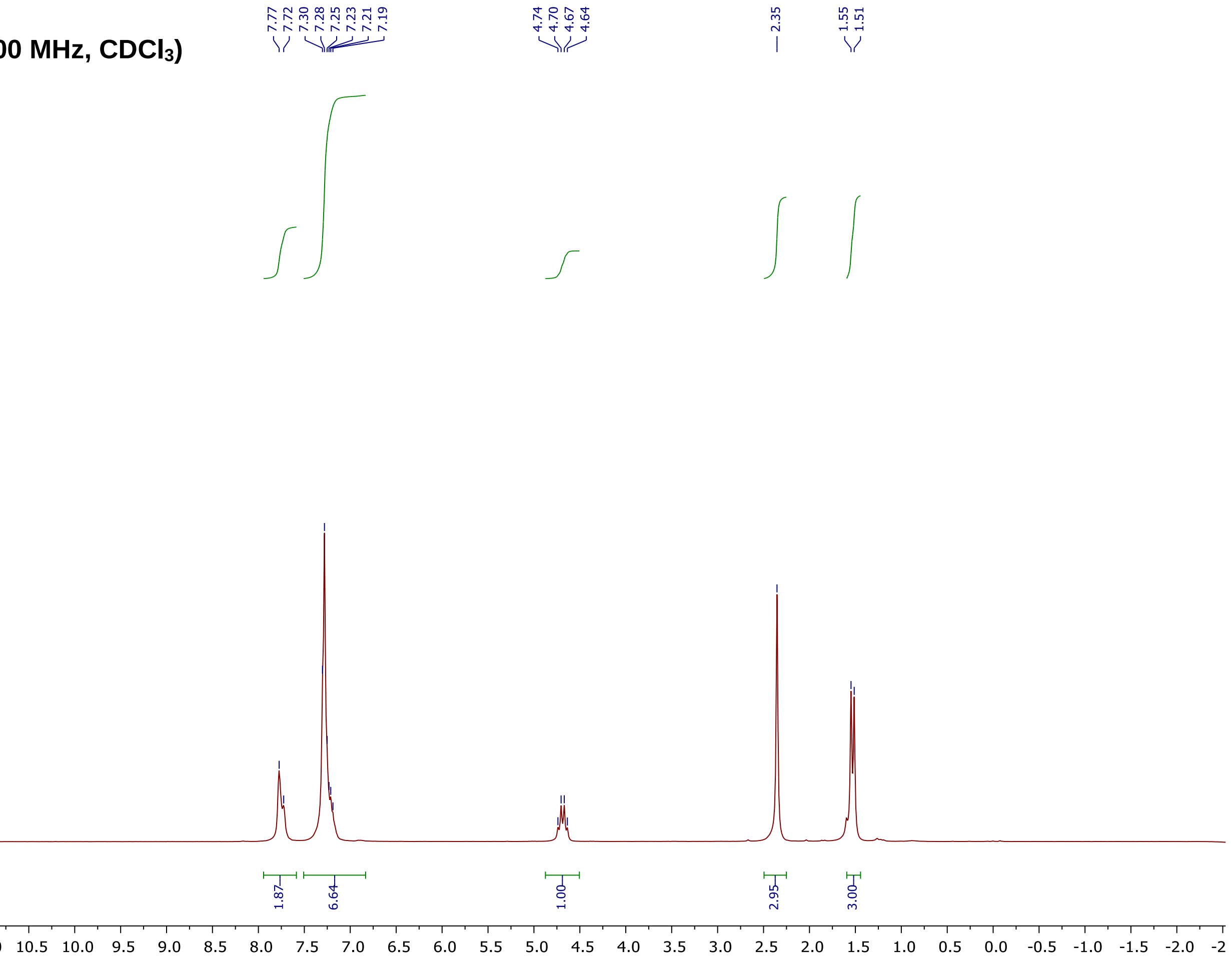

^{13}C NMR (50 MHz, $CDCl_3$)

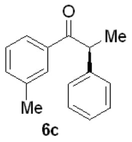

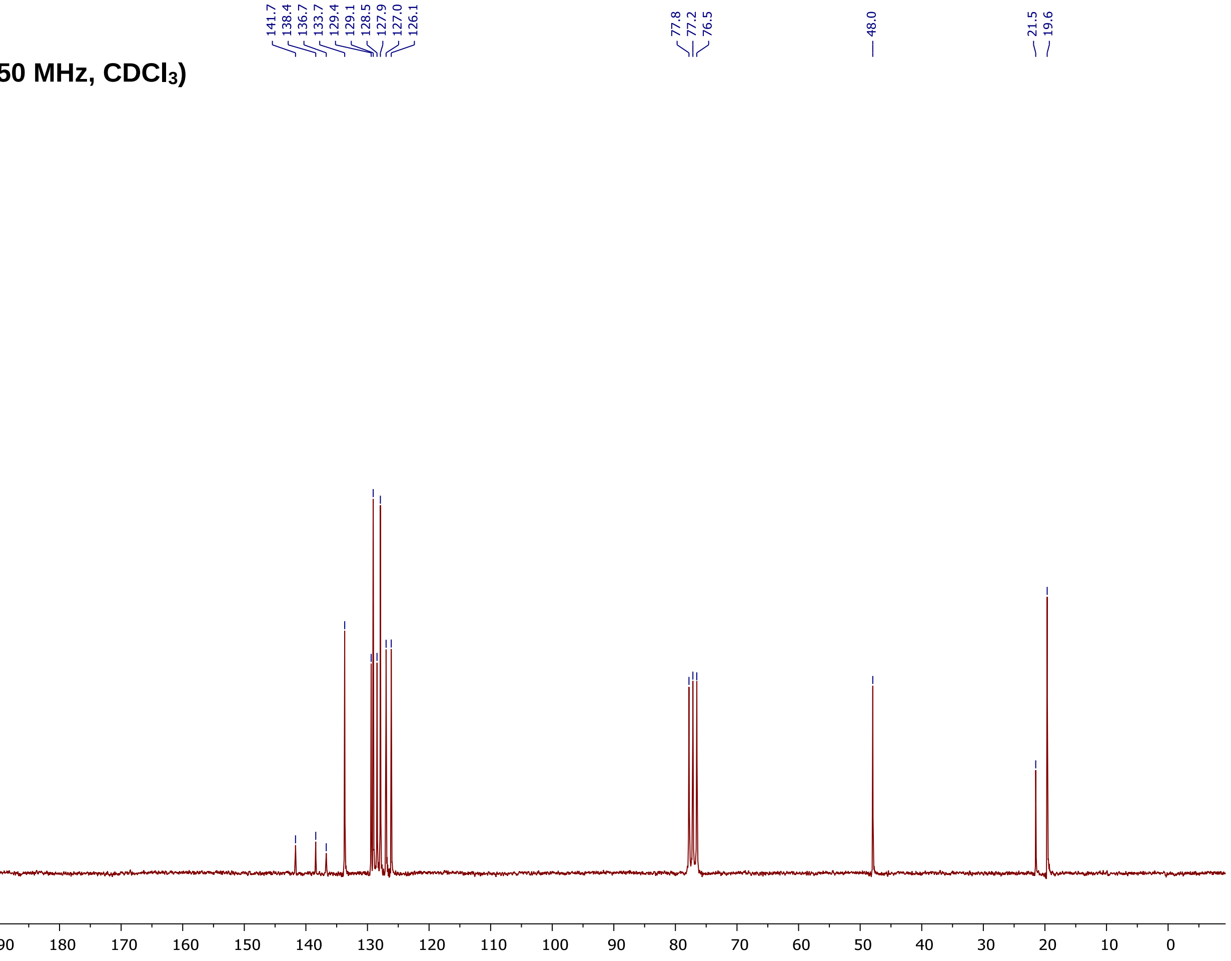

^{1}H NMR (200 MHz, $CDCl_3$)

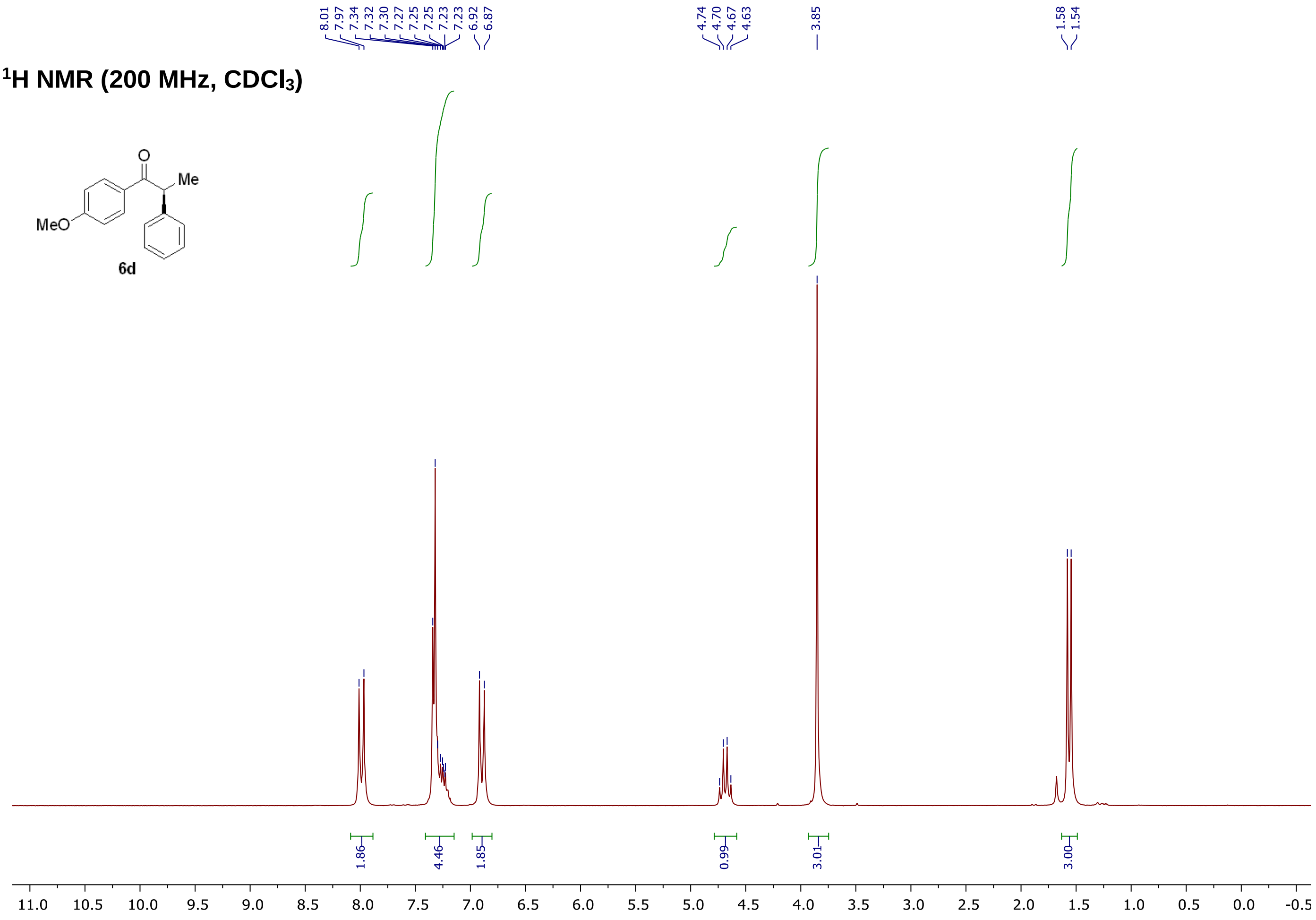

^{13}C NMR (50 MHz, $CDCl_3$)

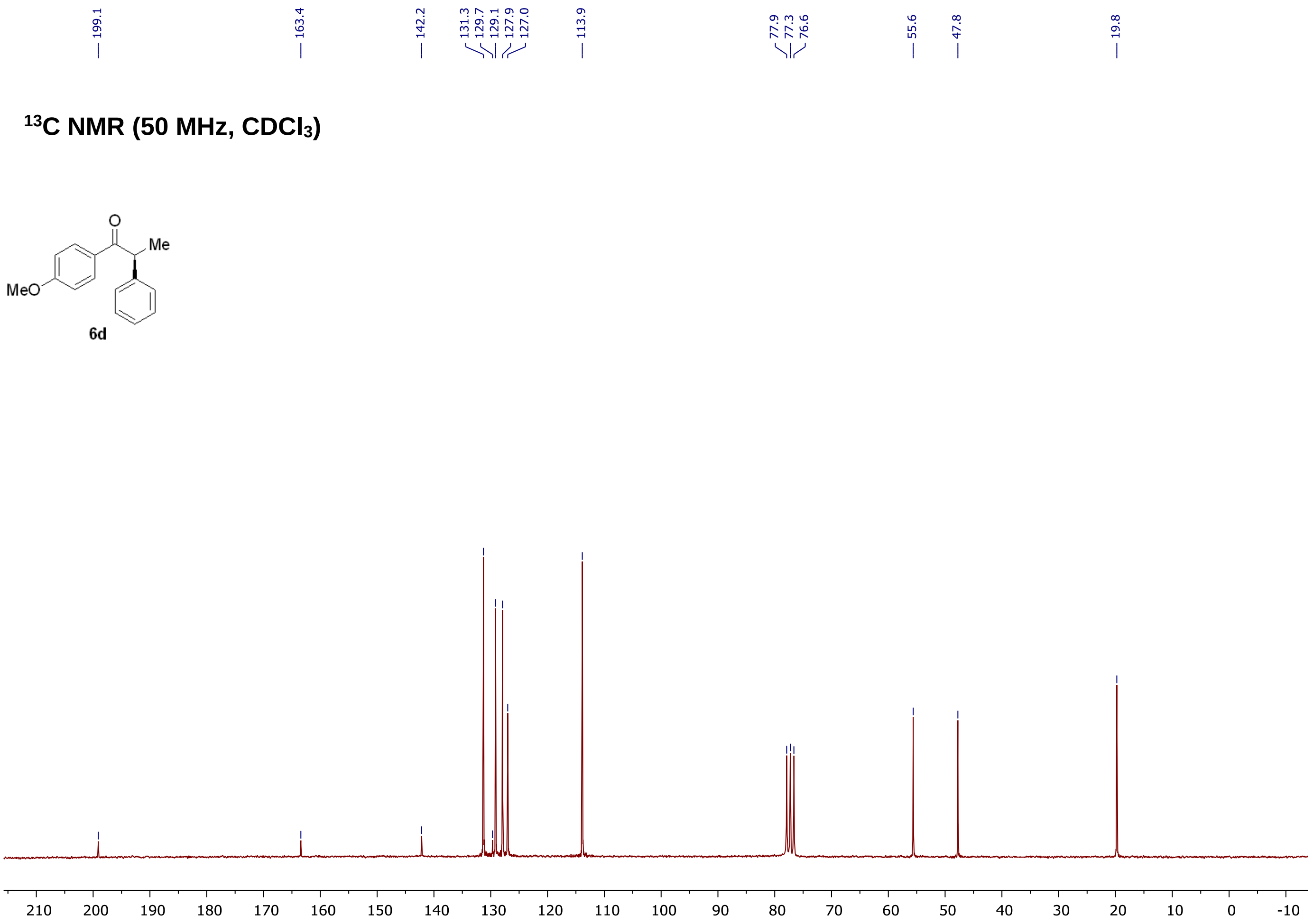

¹H NMR (300 MHz, CDCl3)

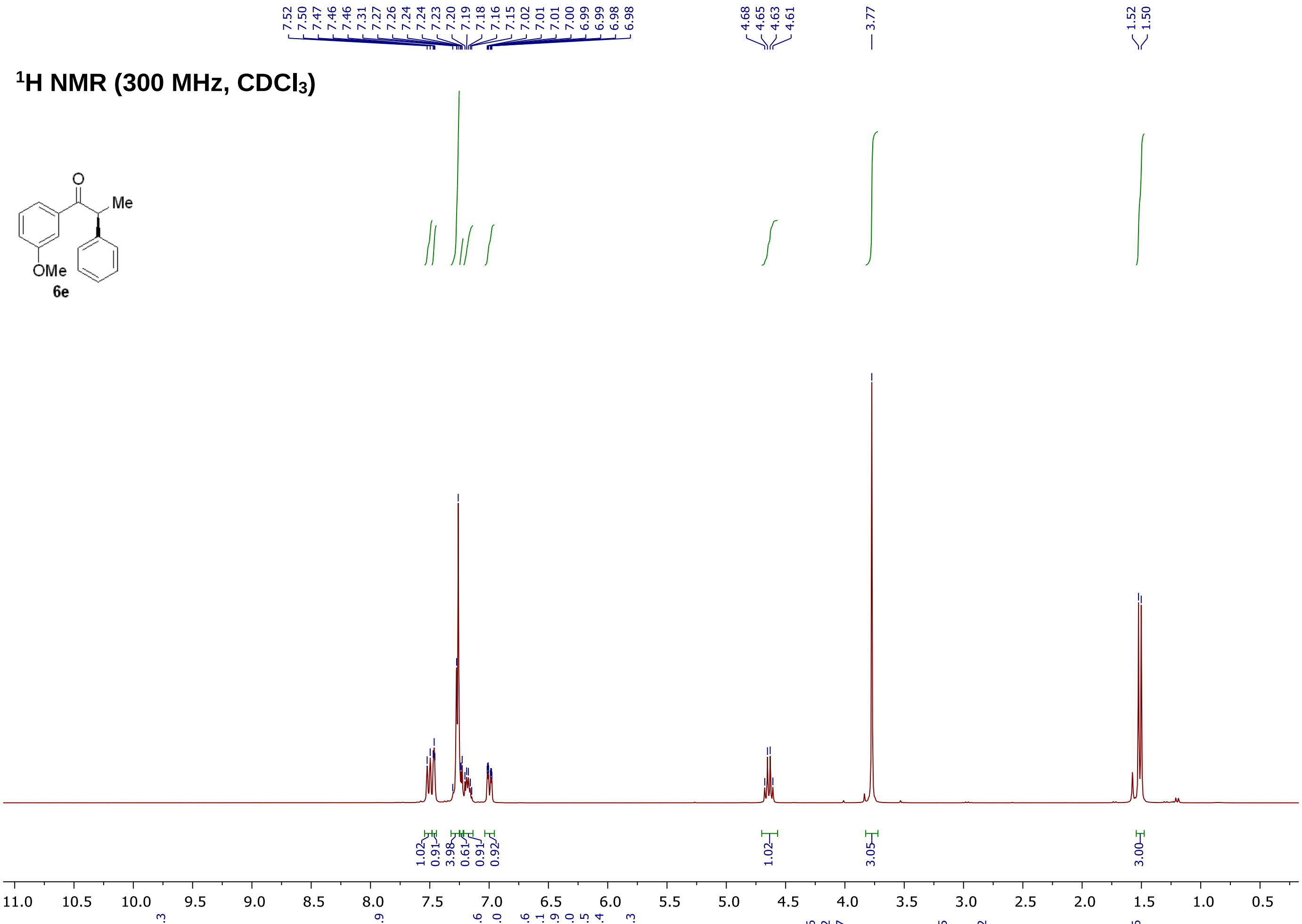

¹³C NMR (75 MHz, CDCl3)

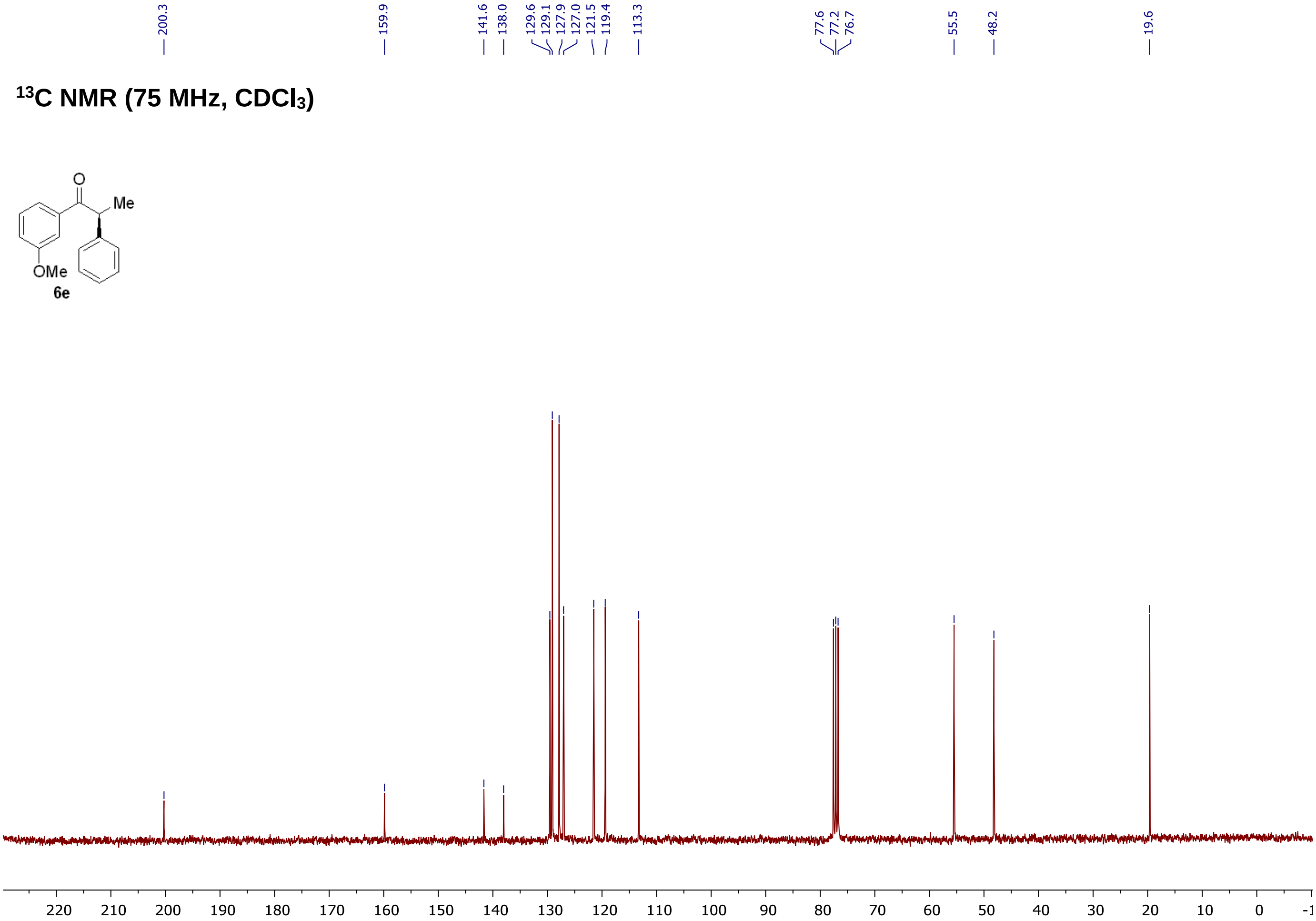

^{1}H NMR (200 MHz, $CDCl_3$)

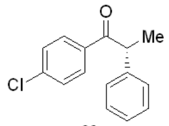

6f

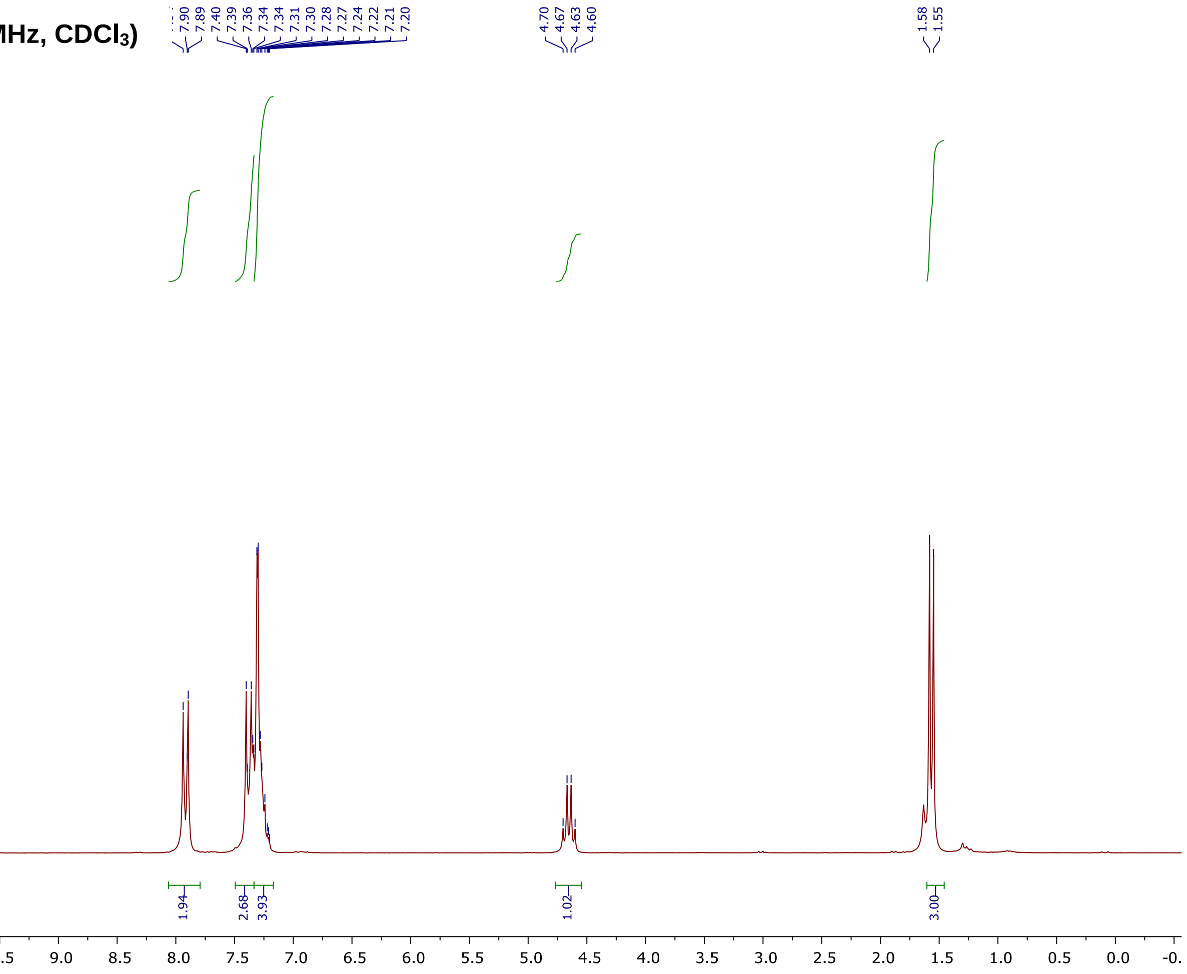

^{13}C NMR (50 MHz, $CDCl_3$

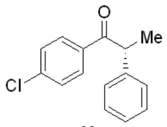

6f

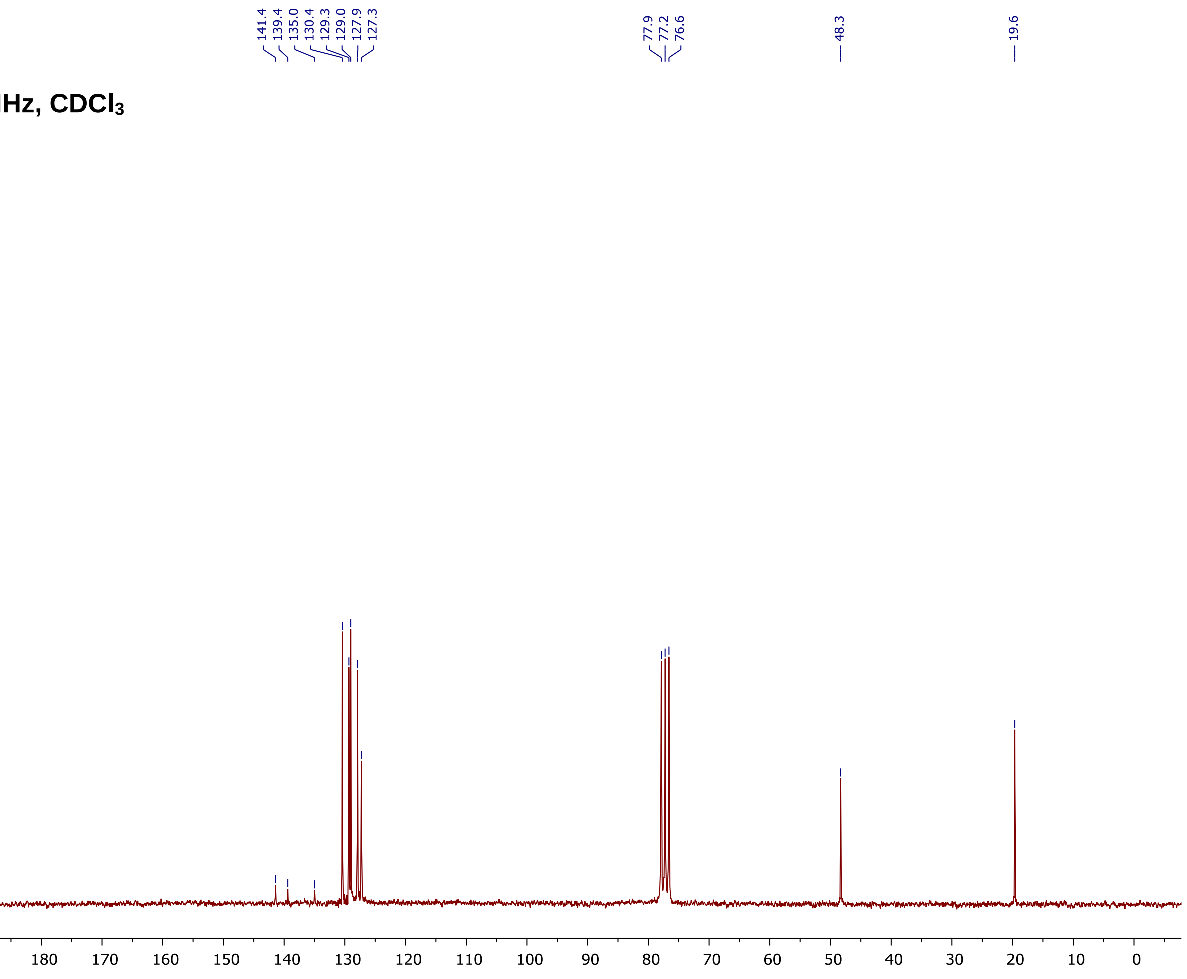

^{1}H NMR (300 MHz, CDCl$_3$)

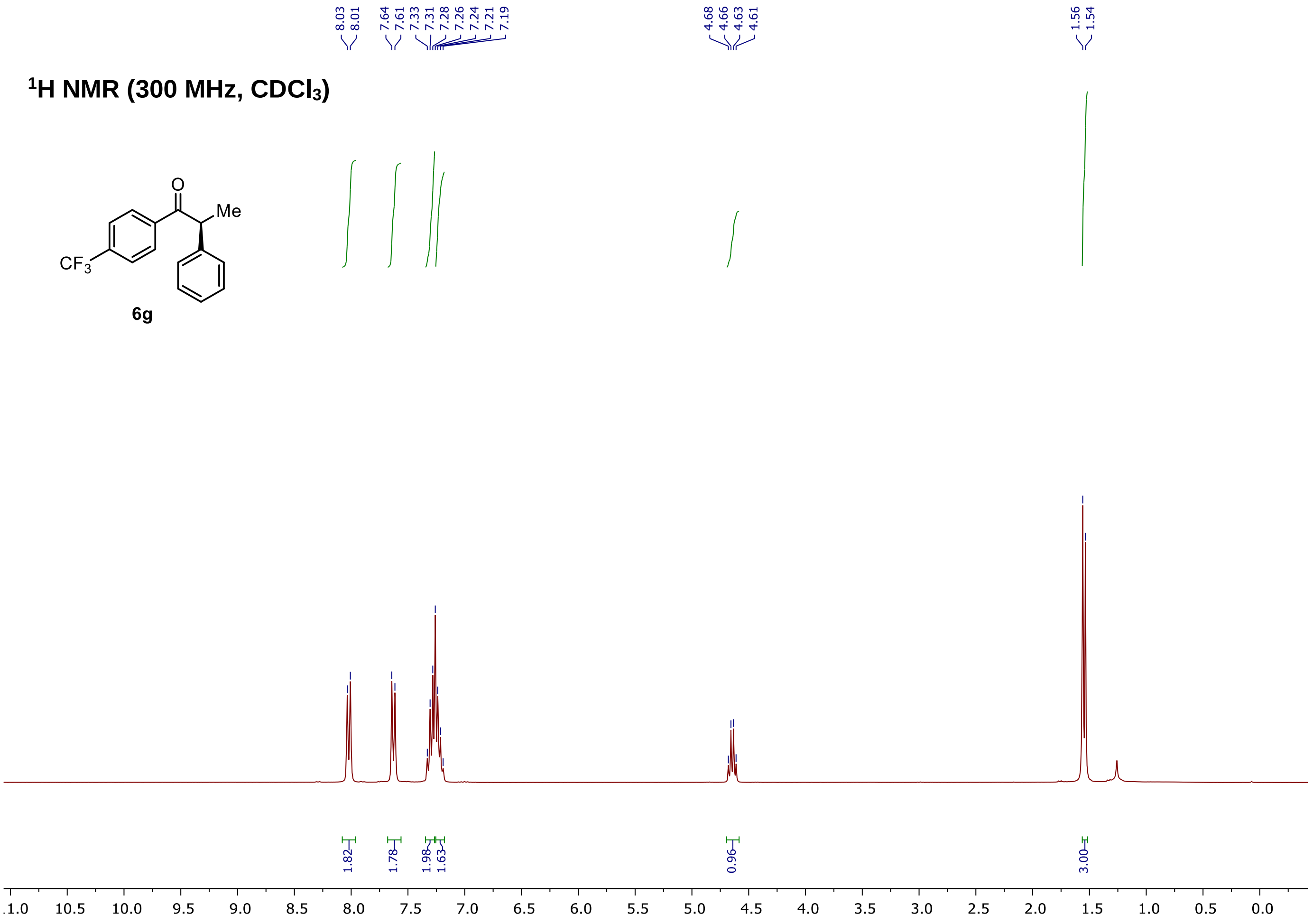

6g

^{13}C NMR (75 MHz, CDCl$_3$)

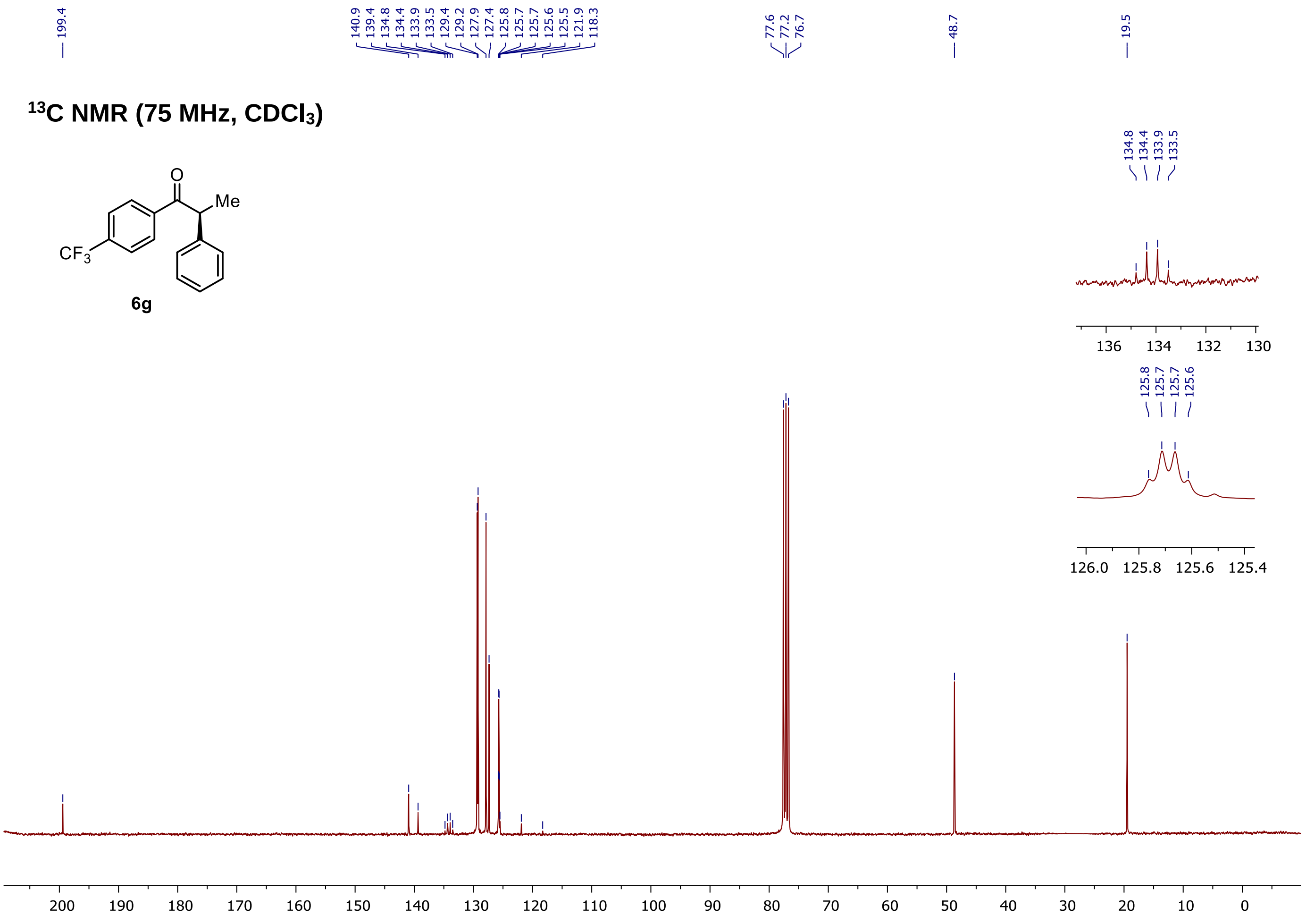

6g

^{19}F NMR (188 MHz, CDCl$_3$)

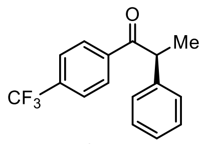

6g

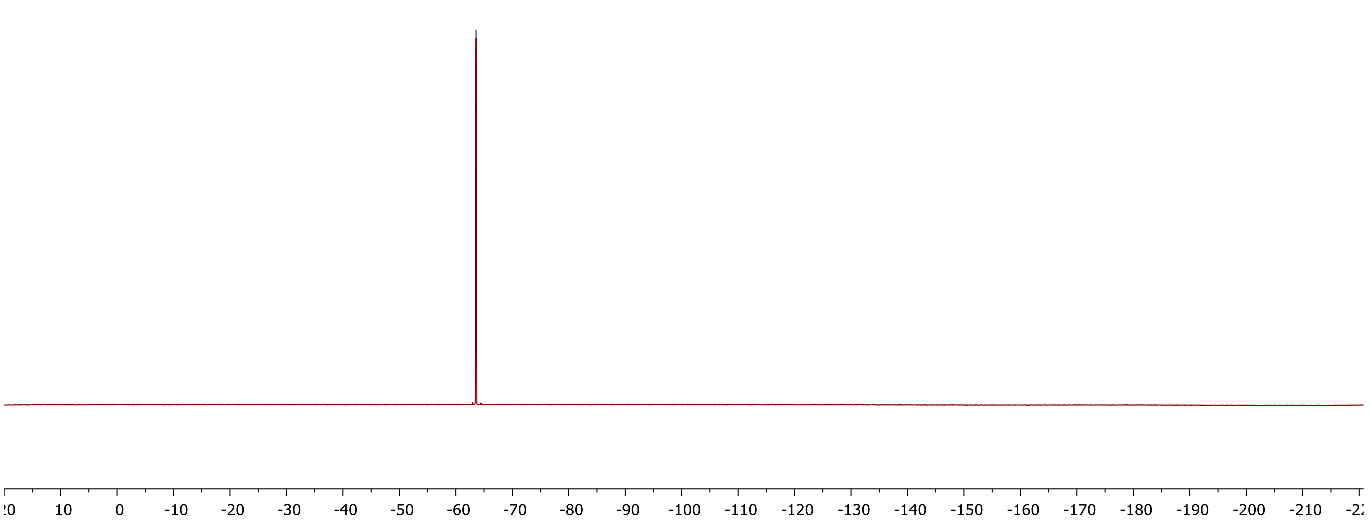

^{1}H NMR (200 MHz, CDCl$_3$)

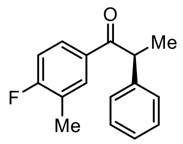

6h

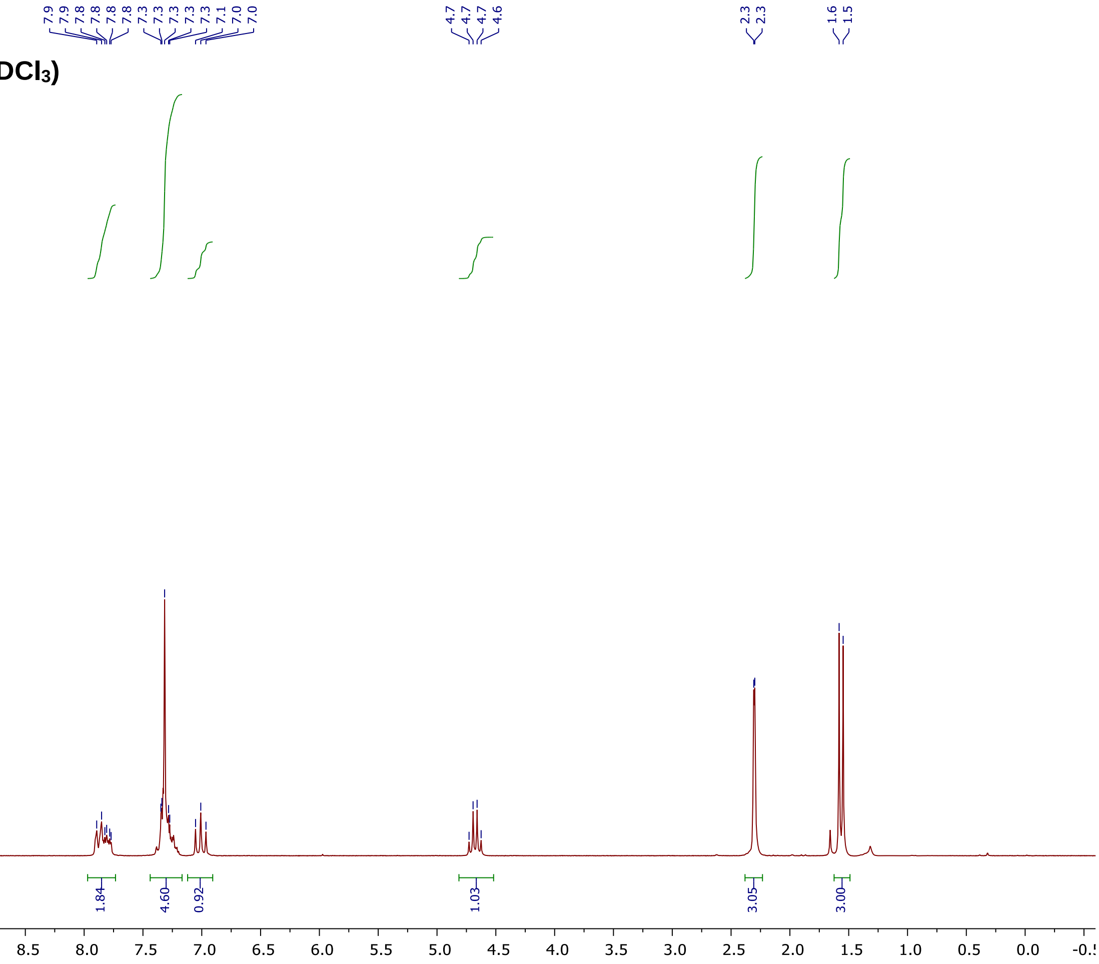

^{13}C NMR (50 MHz, CDCl$_3$)

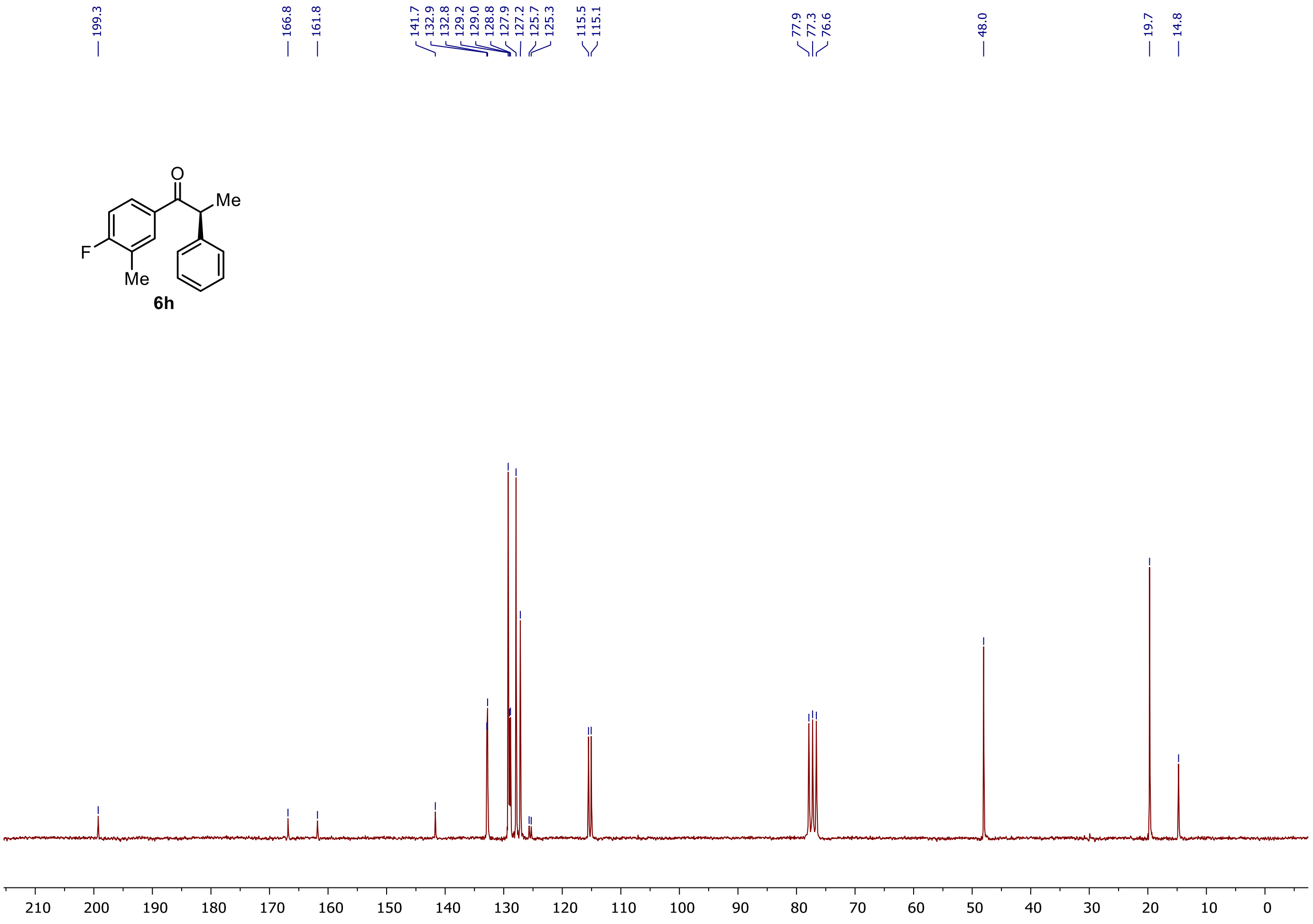

^{19}F NMR (188 MHz, CDCl$_3$)

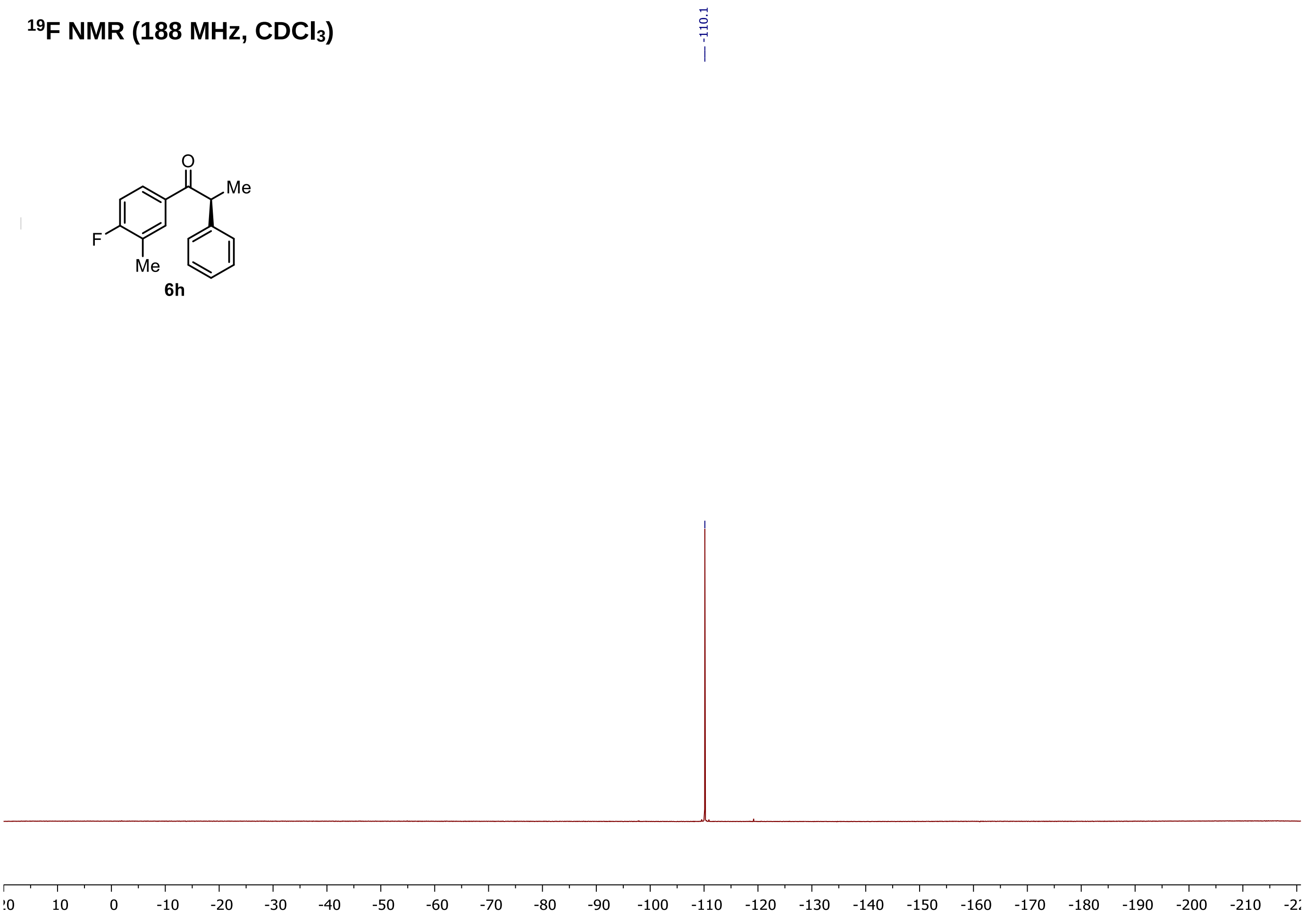

^{1}H NMR (300 MHz, $CDCl_3$)

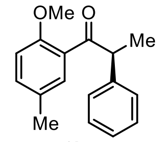

6i

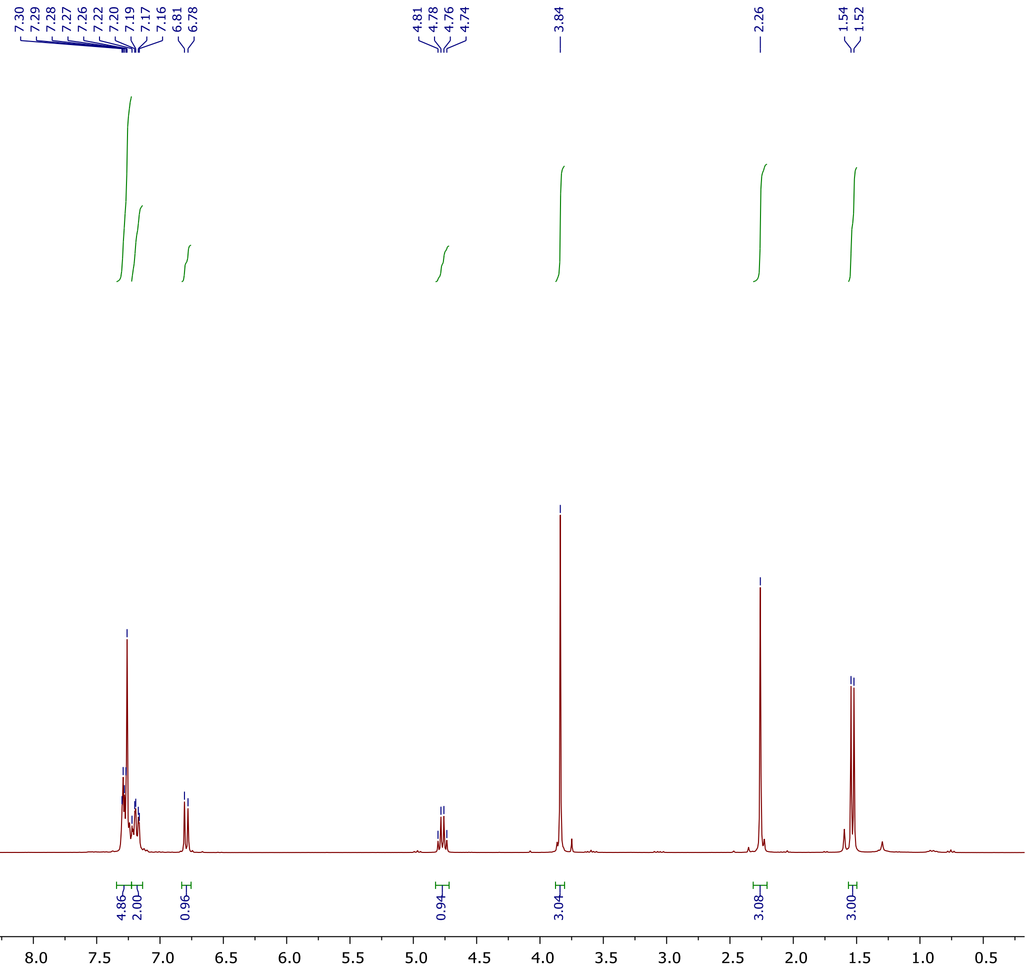

^{13}C NMR (75 MHz, $CDCl_3$)

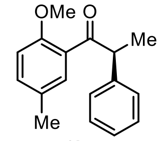

6i

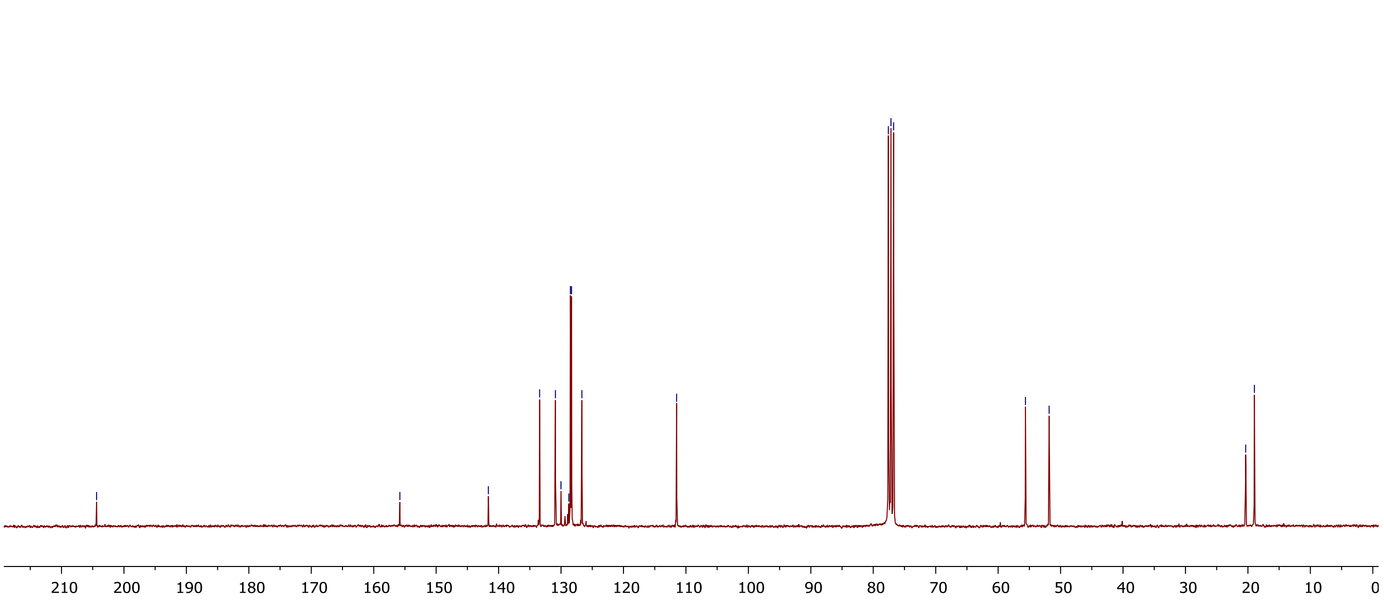

^{1}H NMR (200 MHz, $CDCl_3$)

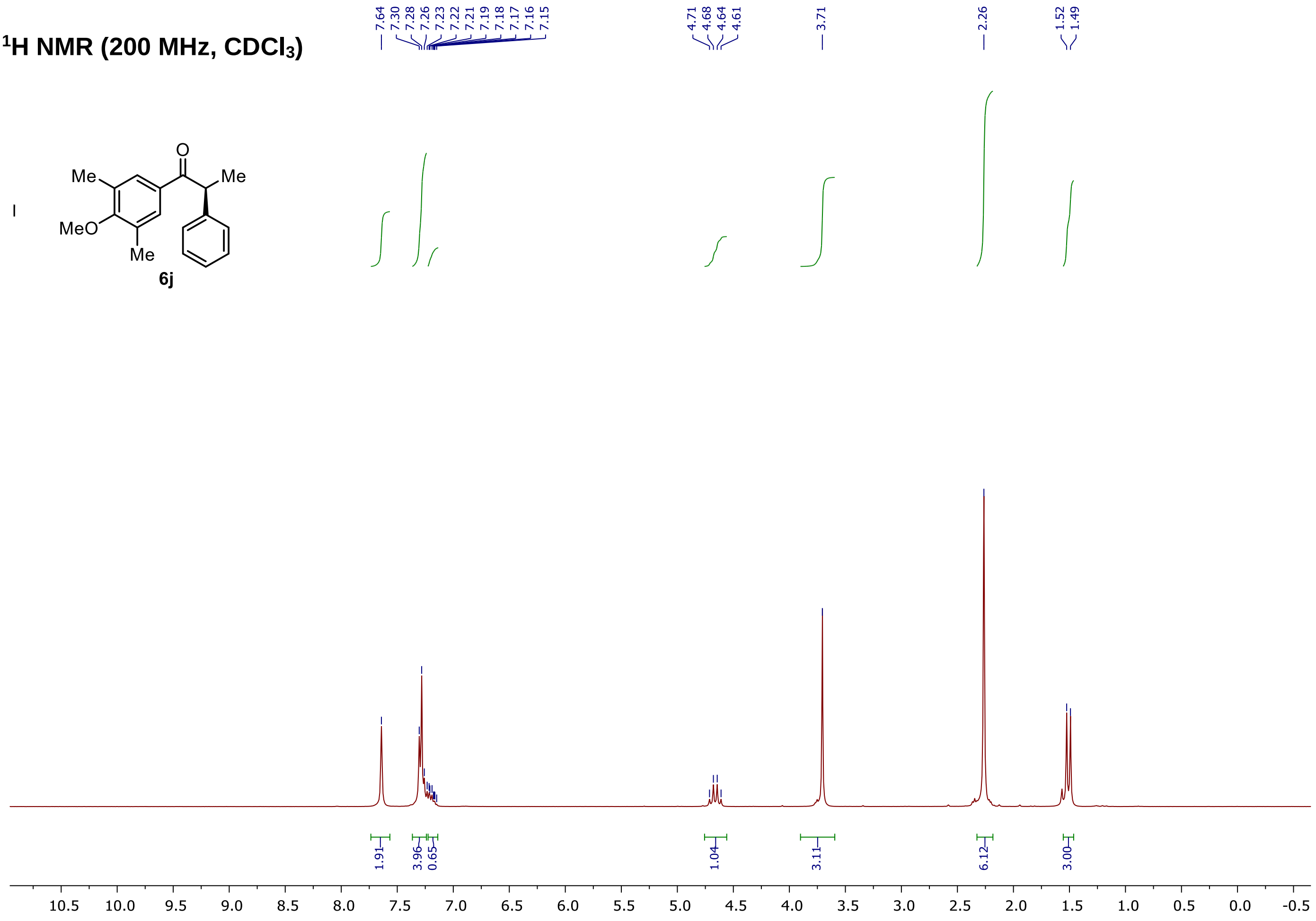

^{13}C NMR (50 MHz, $CDCl_3$)

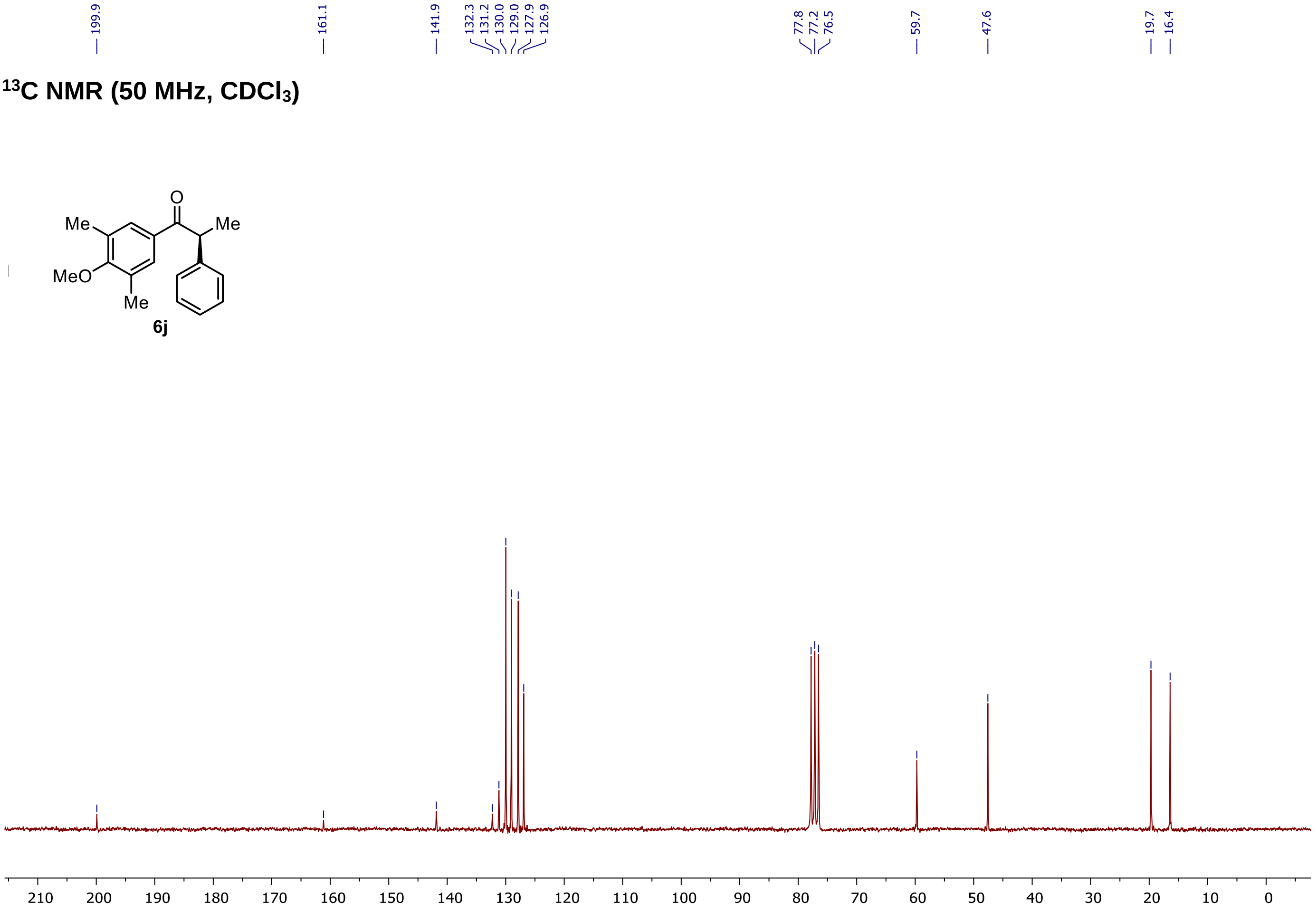

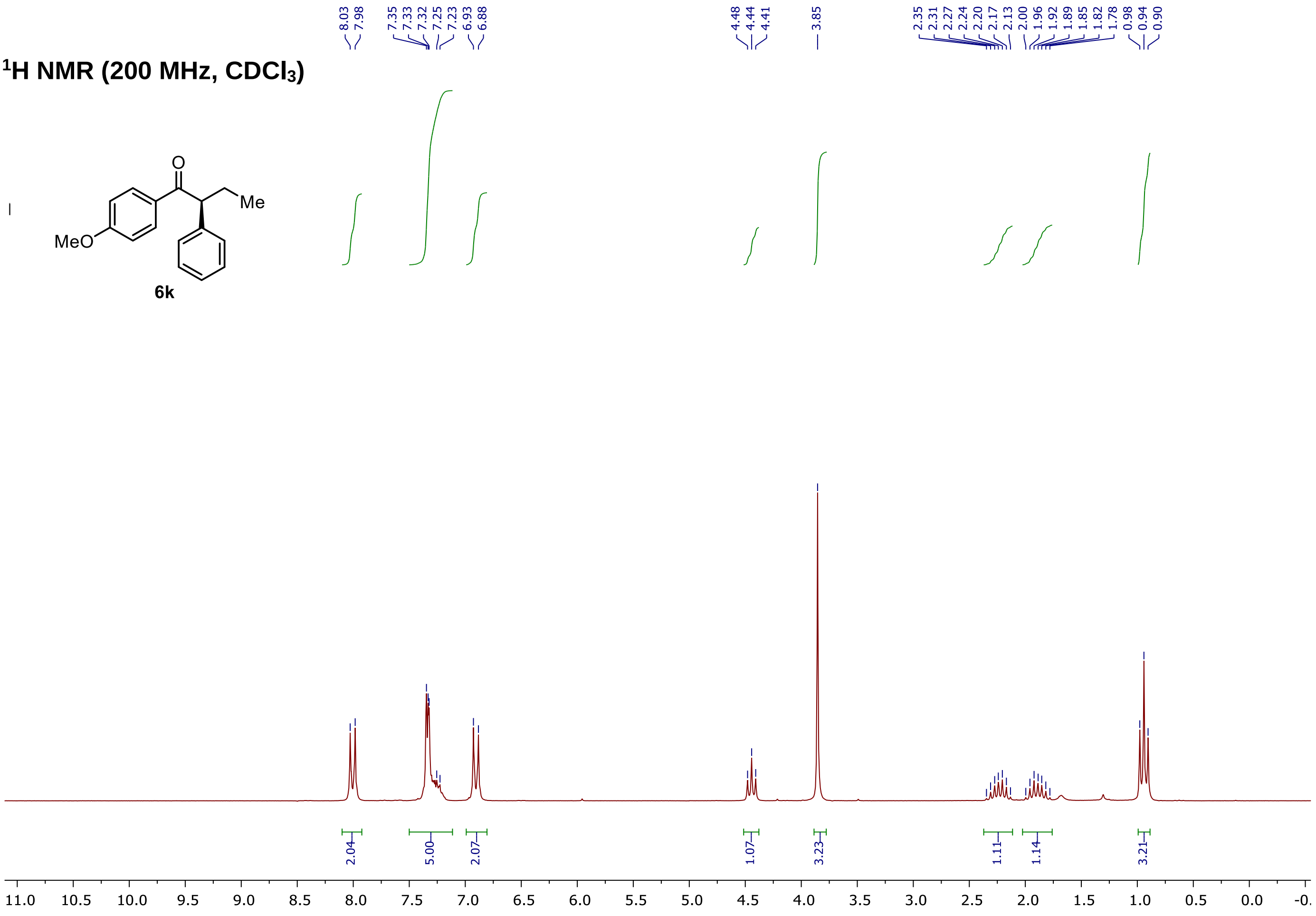

1H NMR (200 MHz, CDCl3)
O
Me
MeO
6k
8.03 7.98 7.35 7.33 7.32 7.25 7.23 6.93 6.88 4.48 4.44 4.41 3.85 2.35 2.31 2.27 2.24 2.20 2.17 2.13 2.00 1.96 1.92 1.89 1.85 1.82 1.78 0.98 0.94 0.90
2.04 5.00 2.07 1.07 3.23 1.11 1.14 3.21
11.0 10.5 10.0 9.5 9.0 8.5 8.0 7.5 7.0 6.5 6.0 5.5 5.0 4.5 4.0 3.5 3.0 2.5 2.0 1.5 1.0 0.5 0.0

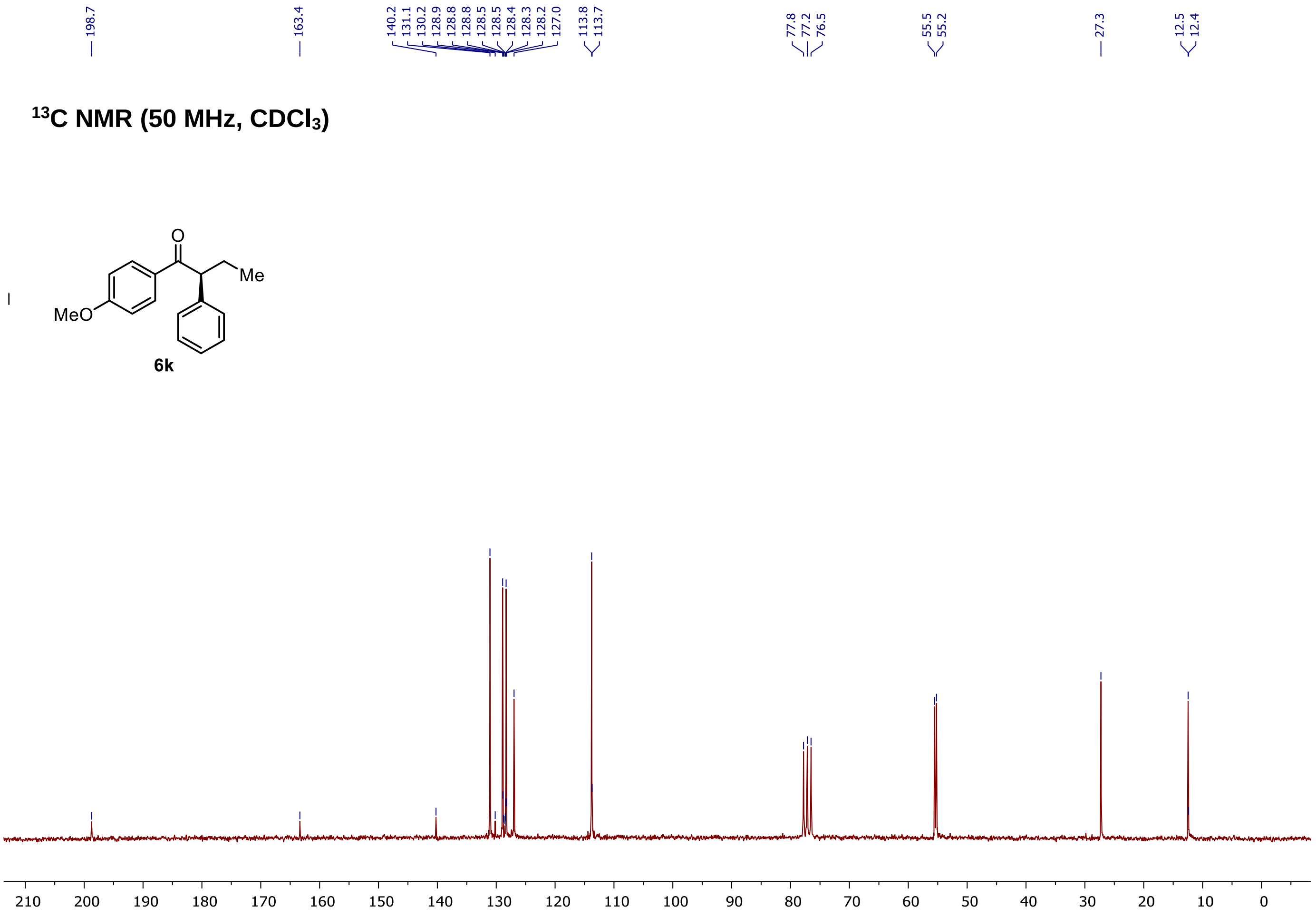

198.7 163.4 140.2 131.1 130.2 128.9 128.8 128.8 128.5 128.4 128.3 128.2 127.0 113.8 113.7 77.8 77.2 76.5 55.5 55.2 27.3 12.5 12.4
13C NMR (50 MHz, CDCl3)
O
Me
MeO
6k
210 200 190 180 170 160 150 140 130 120 110 100 90 80 70 60 50 40 30 20 10 0

^{1}H NMR (200 MHz, $CDCl_3$)

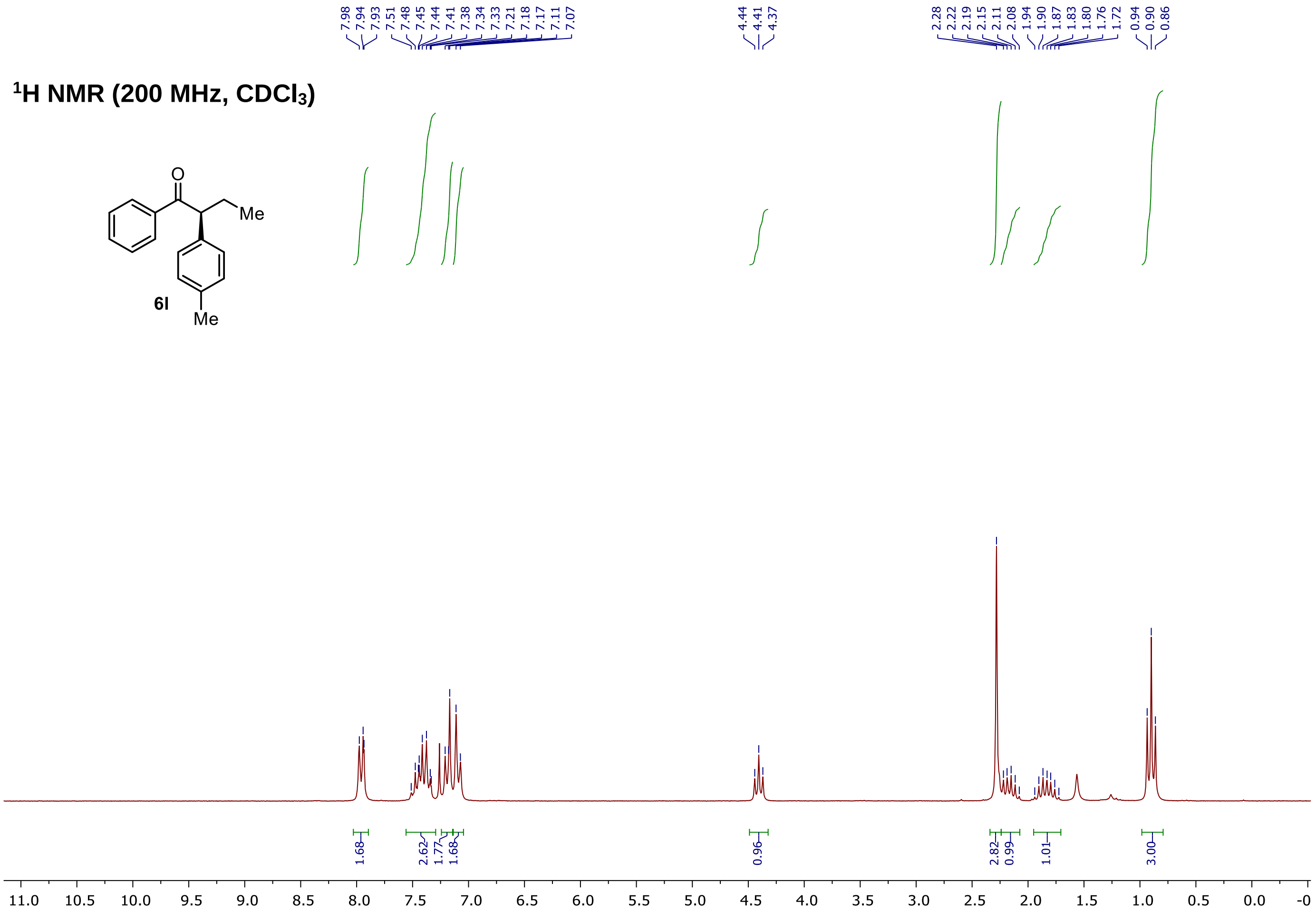

^{13}C NMR (50 MHz, $CDCl_3$)

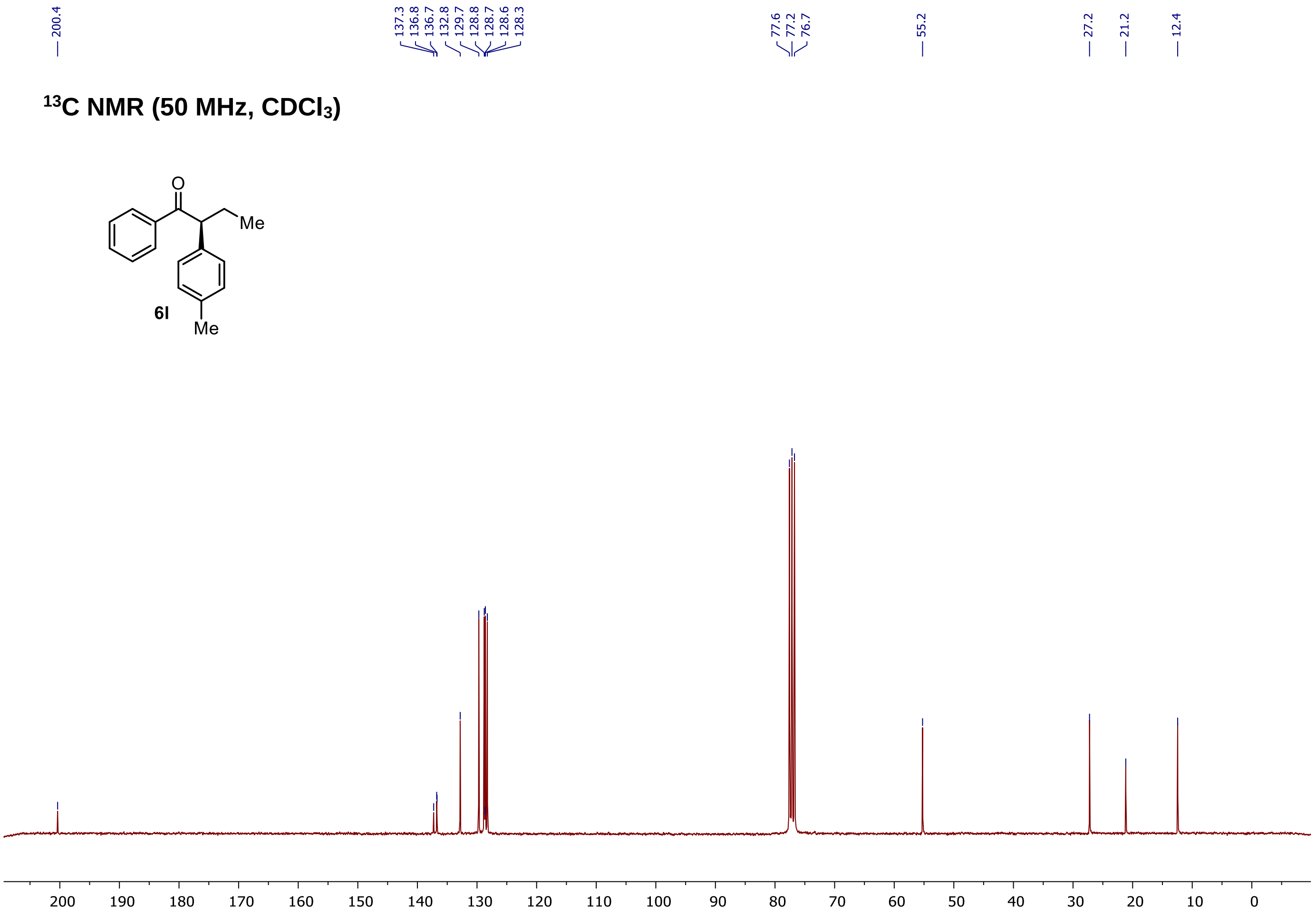

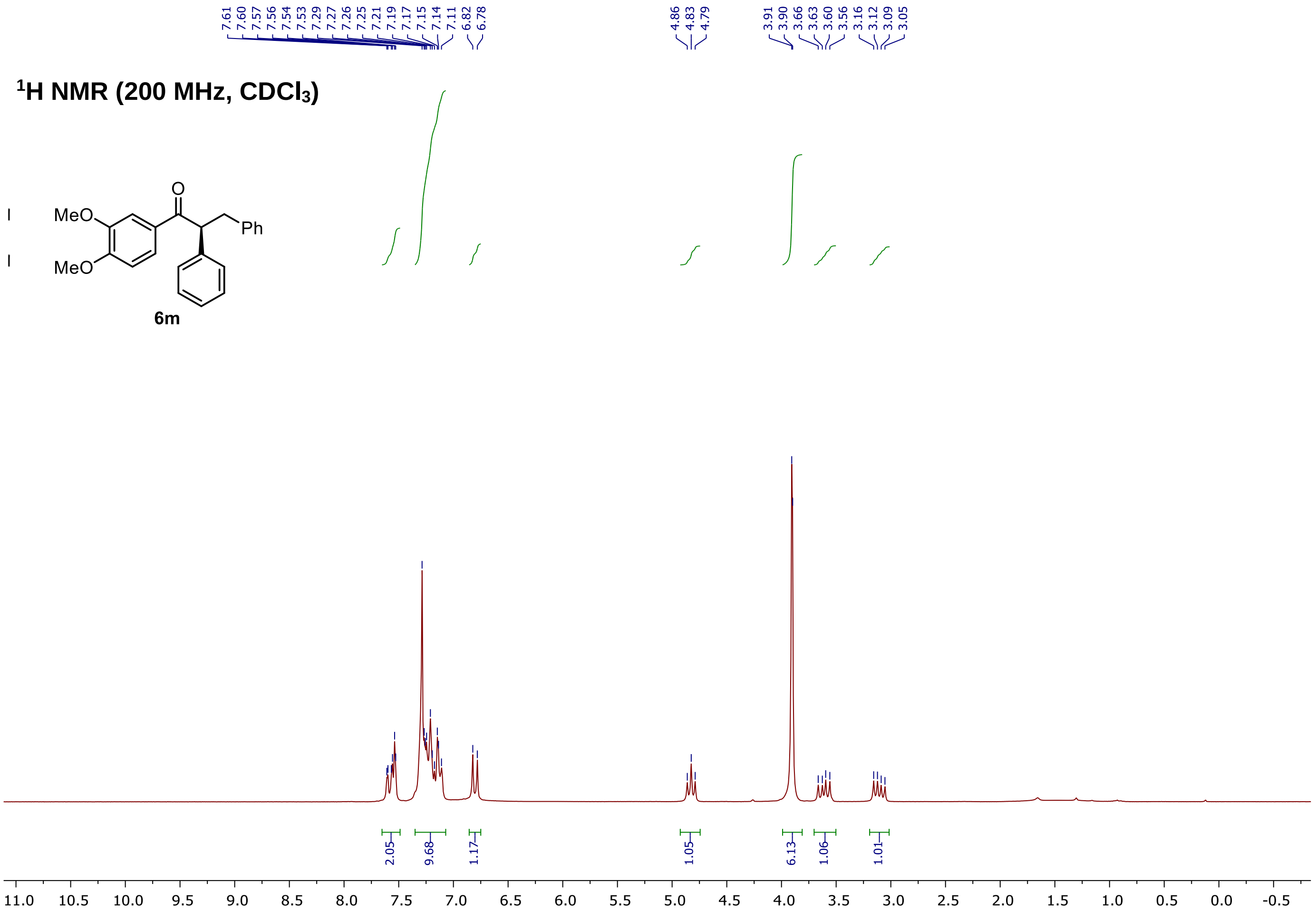

7.61 7.60 7.57 7.56 7.54 7.53 7.29 7.27 7.26 7.25 7.21 7.19 7.17 7.15 7.14 7.11 6.82 6.78
4.86 4.83 4.79
3.91 3.90 3.66 3.63 3.60 3.56 3.16 3.12 3.09 3.05
^{1}H NMR (200 MHz, CDCl$_3$)
MeO
MeO
O
Ph
6m
2.05 9.68 1.17 1.05 6.13 1.06 1.01
11.0 10.5 10.0 9.5 9.0 8.5 8.0 7.5 7.0 6.5 6.0 5.5 5.0 4.5 4.0 3.5 3.0 2.5 2.0 1.5 1.0 0.5 0.0 -0.5

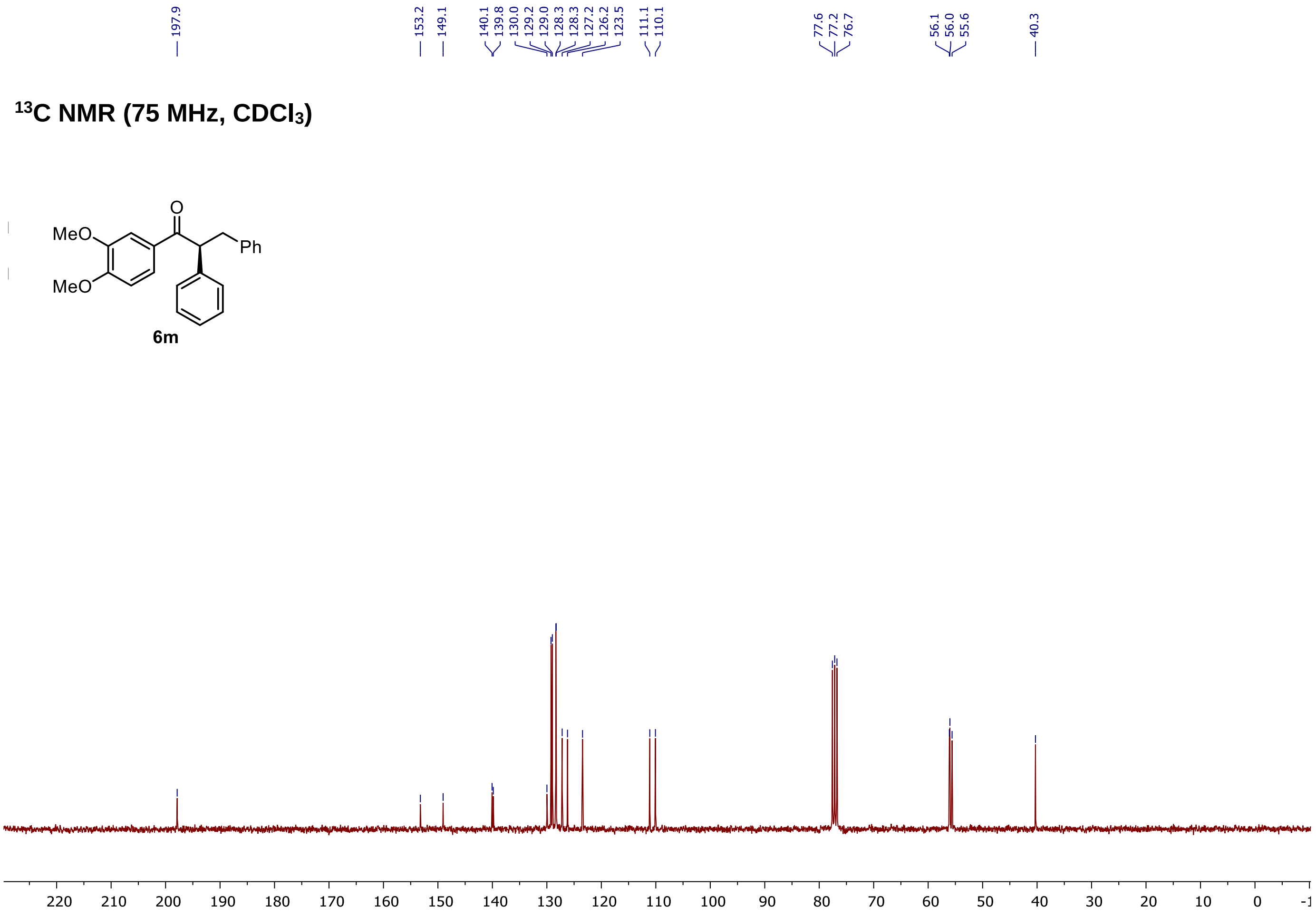

197.9
153.2 149.1
140.1 139.8 130.0 129.2 129.0 128.3 128.3 127.2 126.2 123.5
111.1 110.1
77.6 77.2 76.7
56.1 56.0 55.6
40.3
^{13}C NMR (75 MHz, CDCl$_3$)
MeO
MeO
O
Ph
6m
220 210 200 190 180 170 160 150 140 130 120 110 100 90 80 70 60 50 40 30 20 10 0

^{1}H NMR (300 MHz, $CDCl_3$)

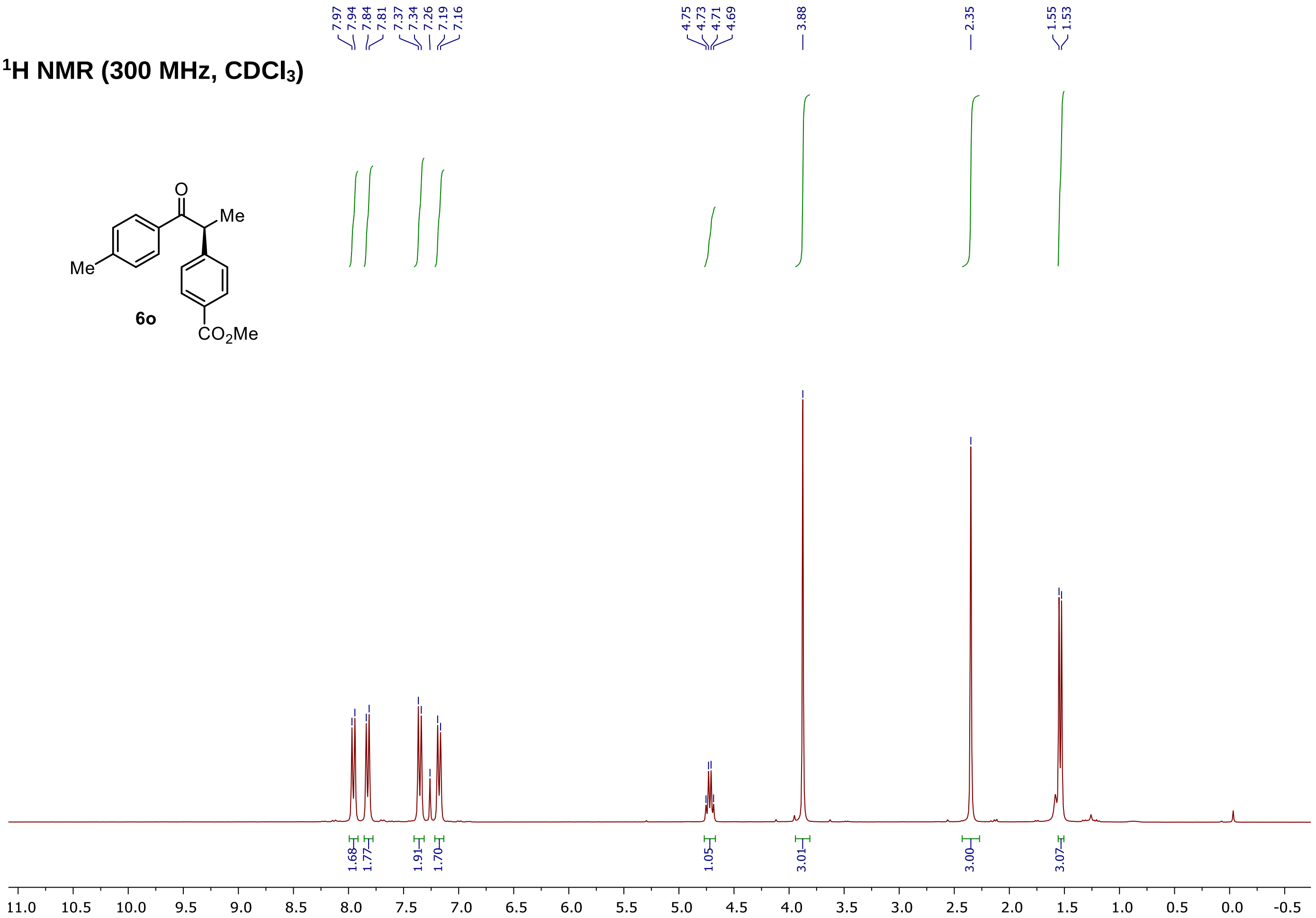

^{13}C NMR (75 MHz, $CDCl_3$)

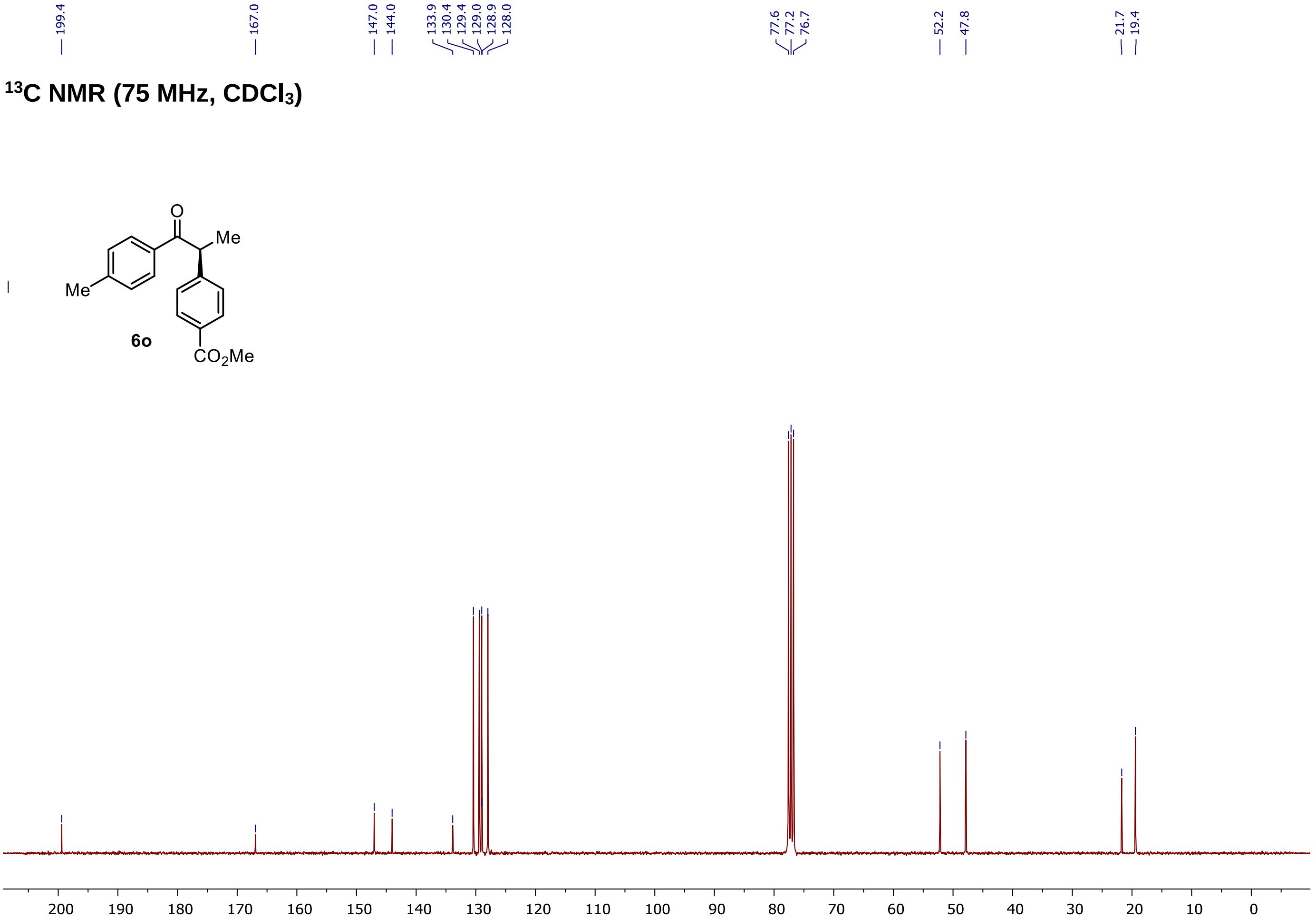

^1H NMR (300 MHz, $CDCl_3$)

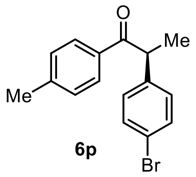

6p

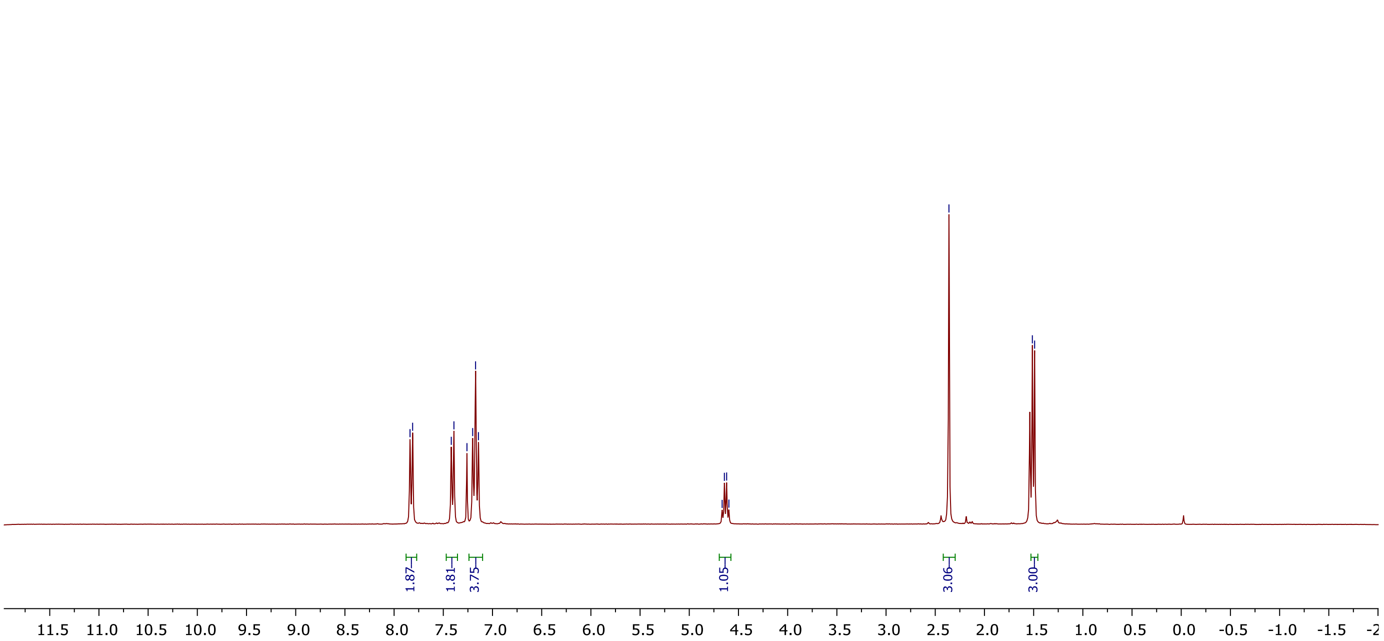

^{13}C NMR (75 MHz, $CDCl_3$)

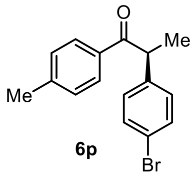

6p

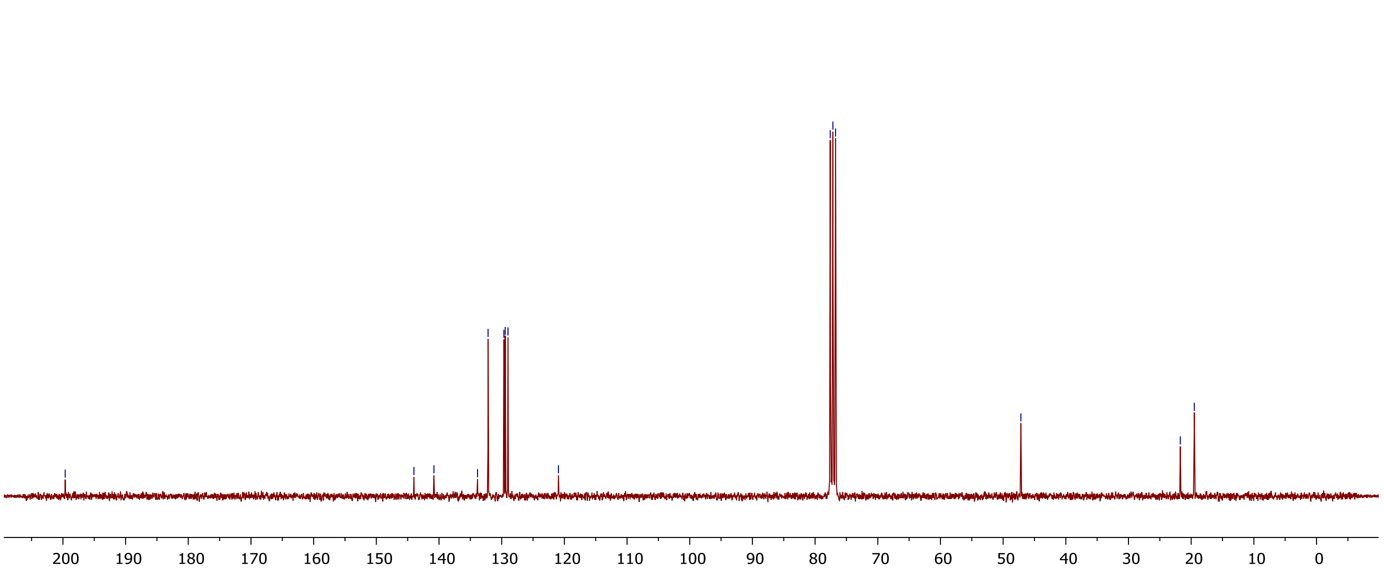

^{1}H NMR (300 MHz, $CDCl_3$)

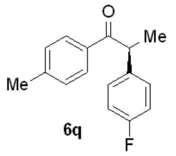

6q

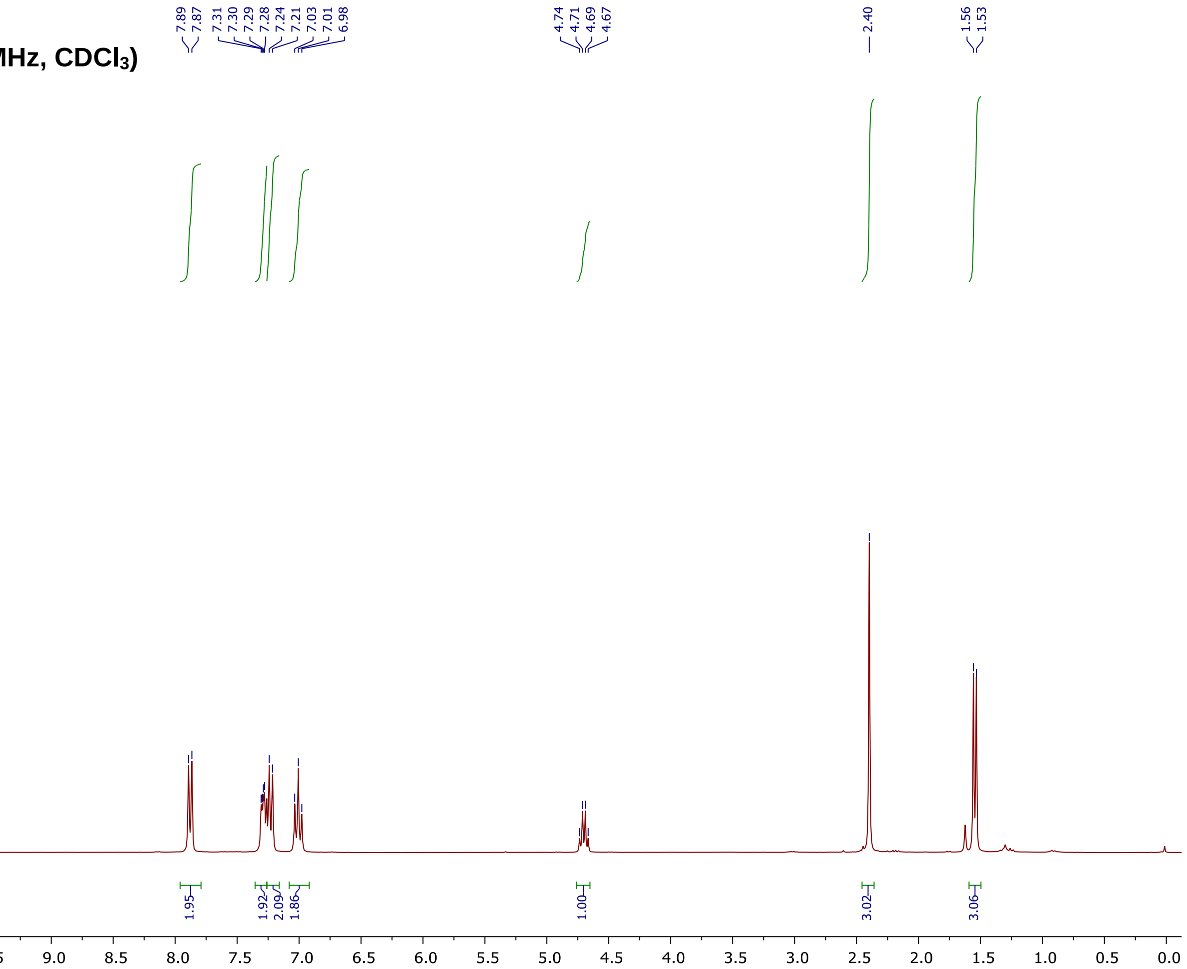

^{13}C NMR (50 MHz, $CDCl_3$)

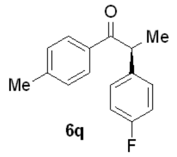

6q

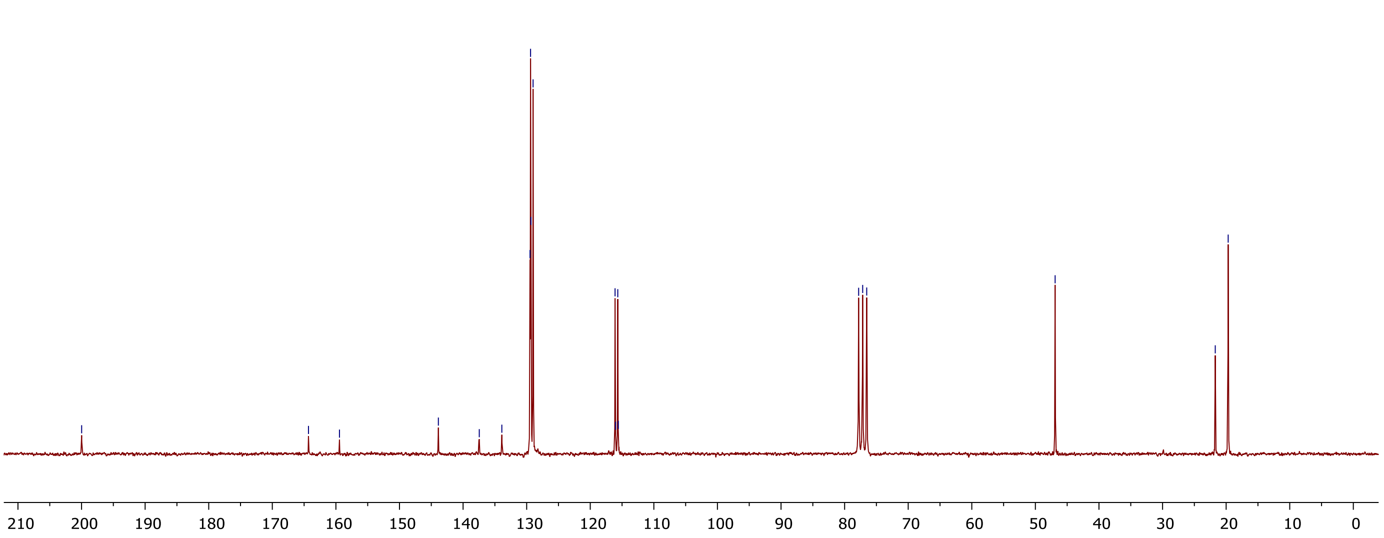

^{19}F NMR (188 MHz, $CDCl_3$)

6q

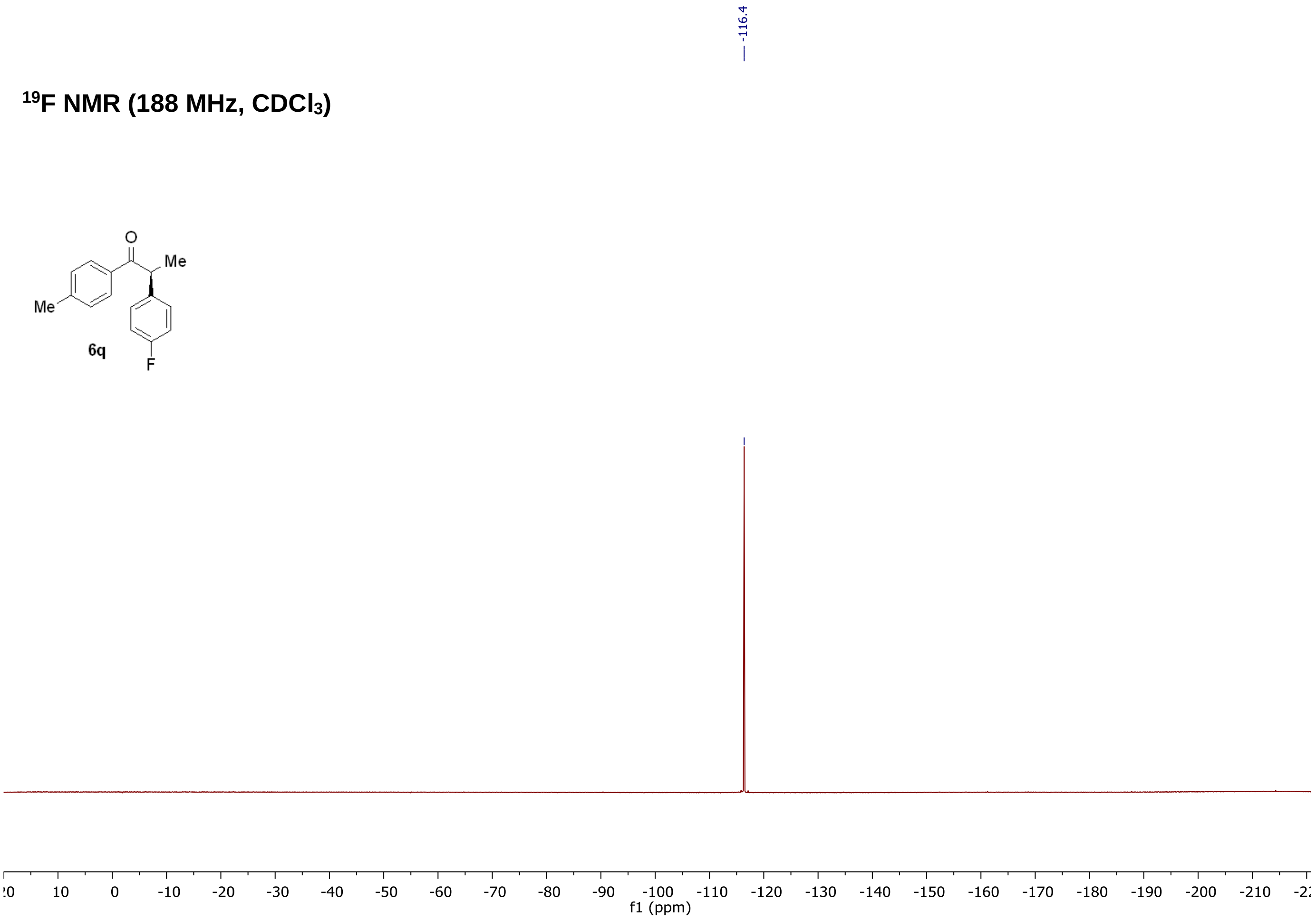

^{1}H NMR (200 MHz, $CDCl_3$)

6r

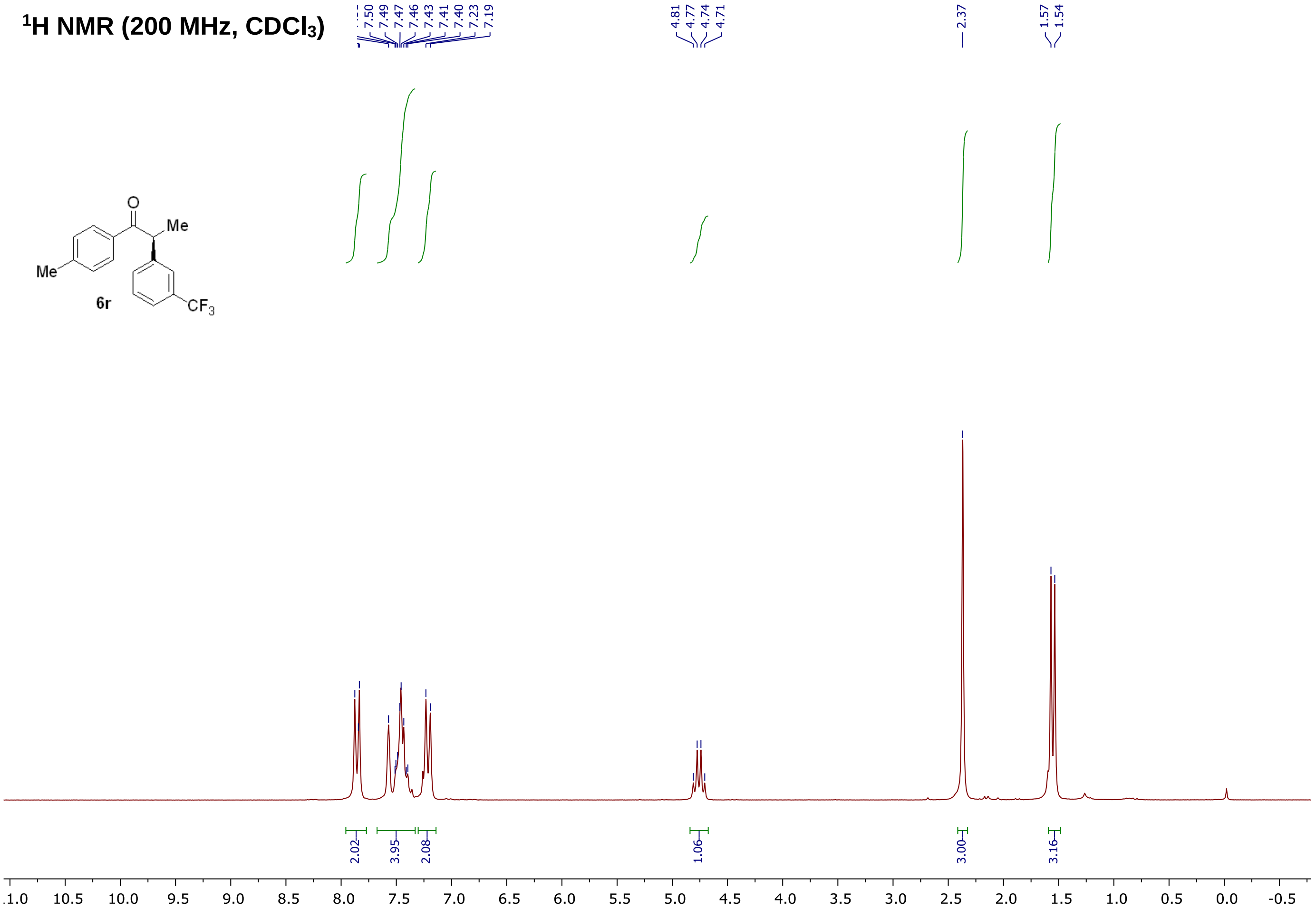

^{13}C NMR (75 MHz, $CDCl_3$)

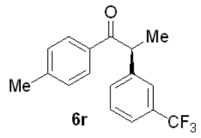

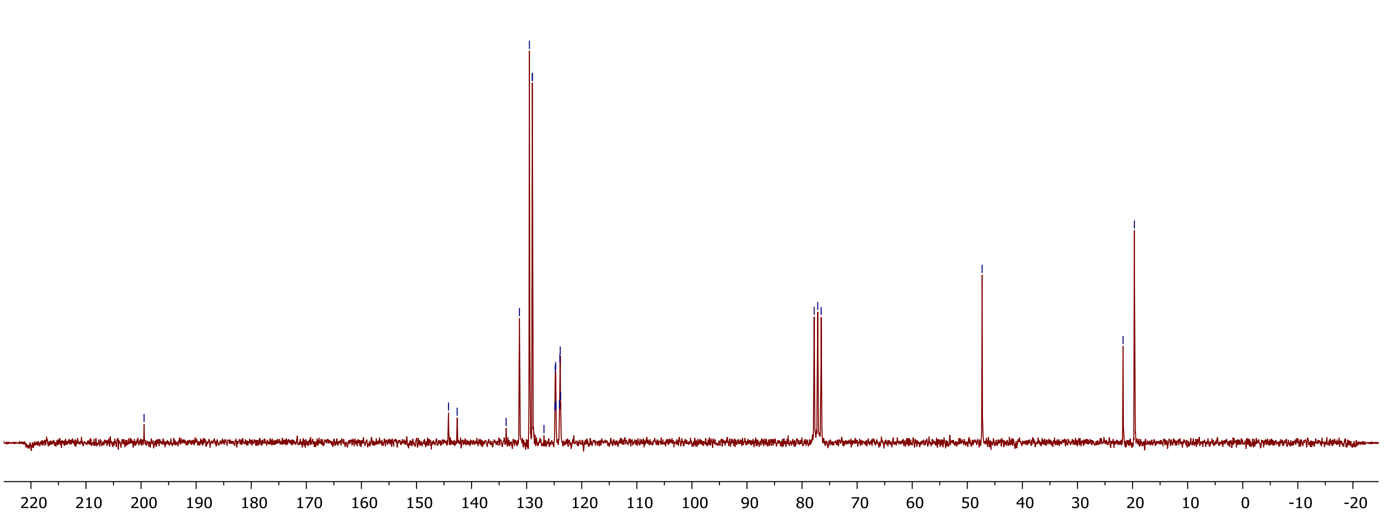

^{19}F NMR (188 MHz, $CDCl_3$)

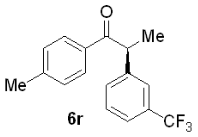

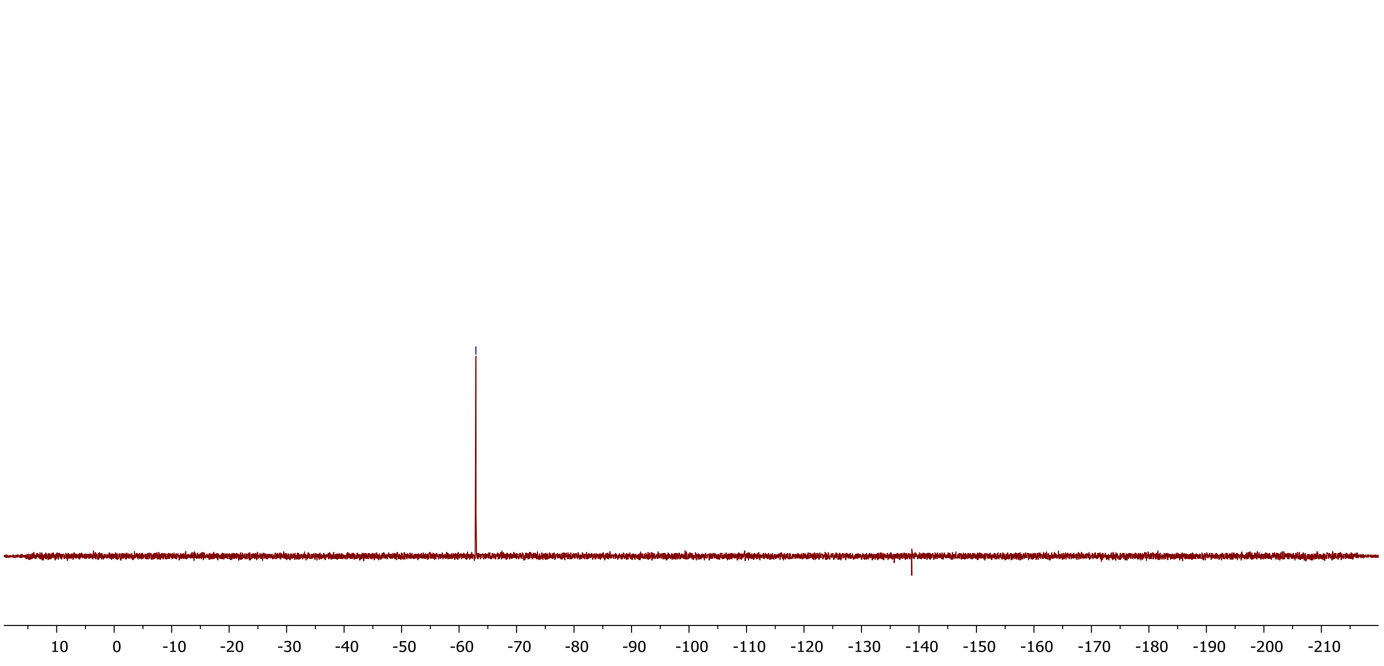

^{1}H NMR (200 MHz, $CDCl_3$)

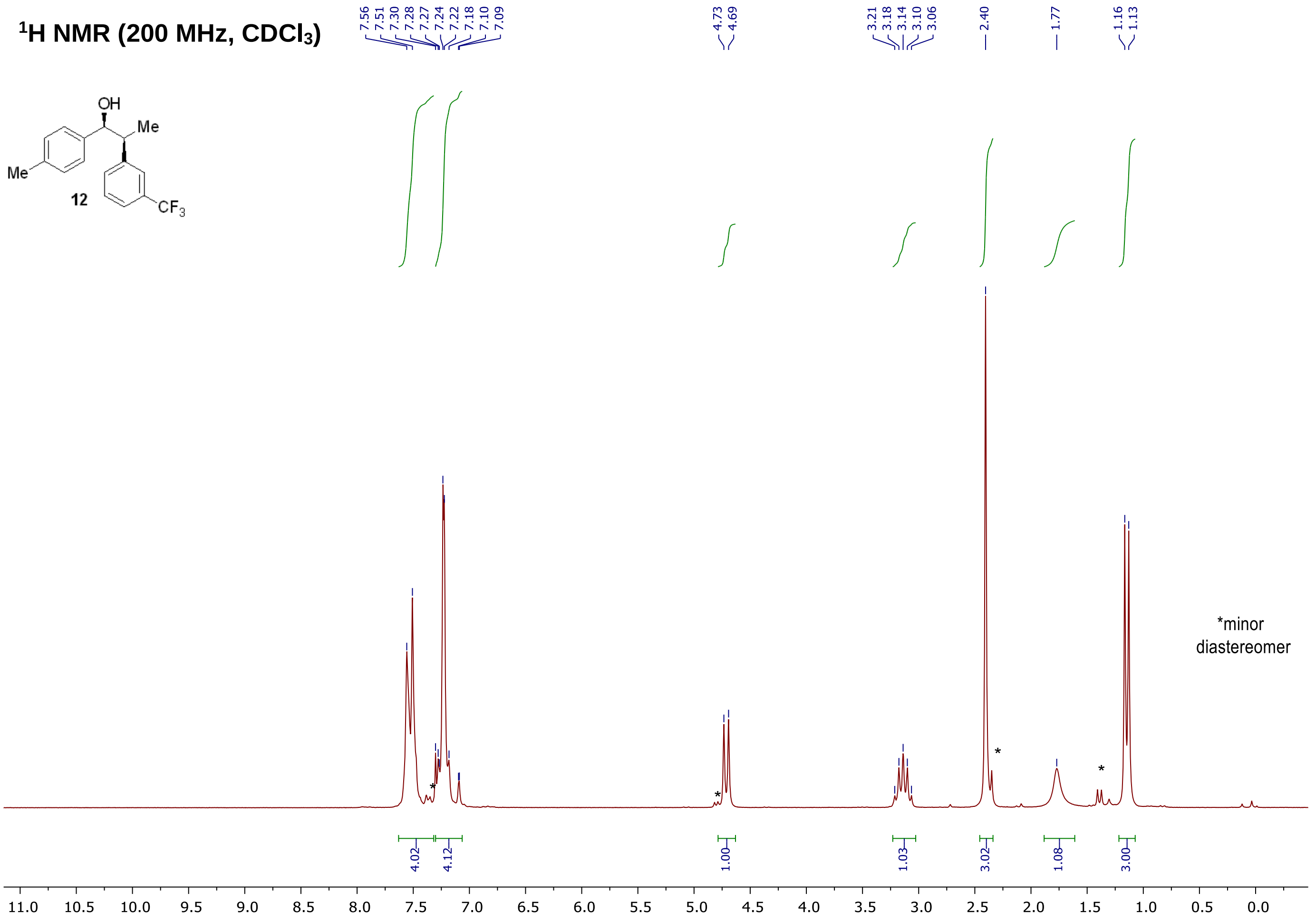

^{13}C NMR (50 MHz, $CDCl_3$)

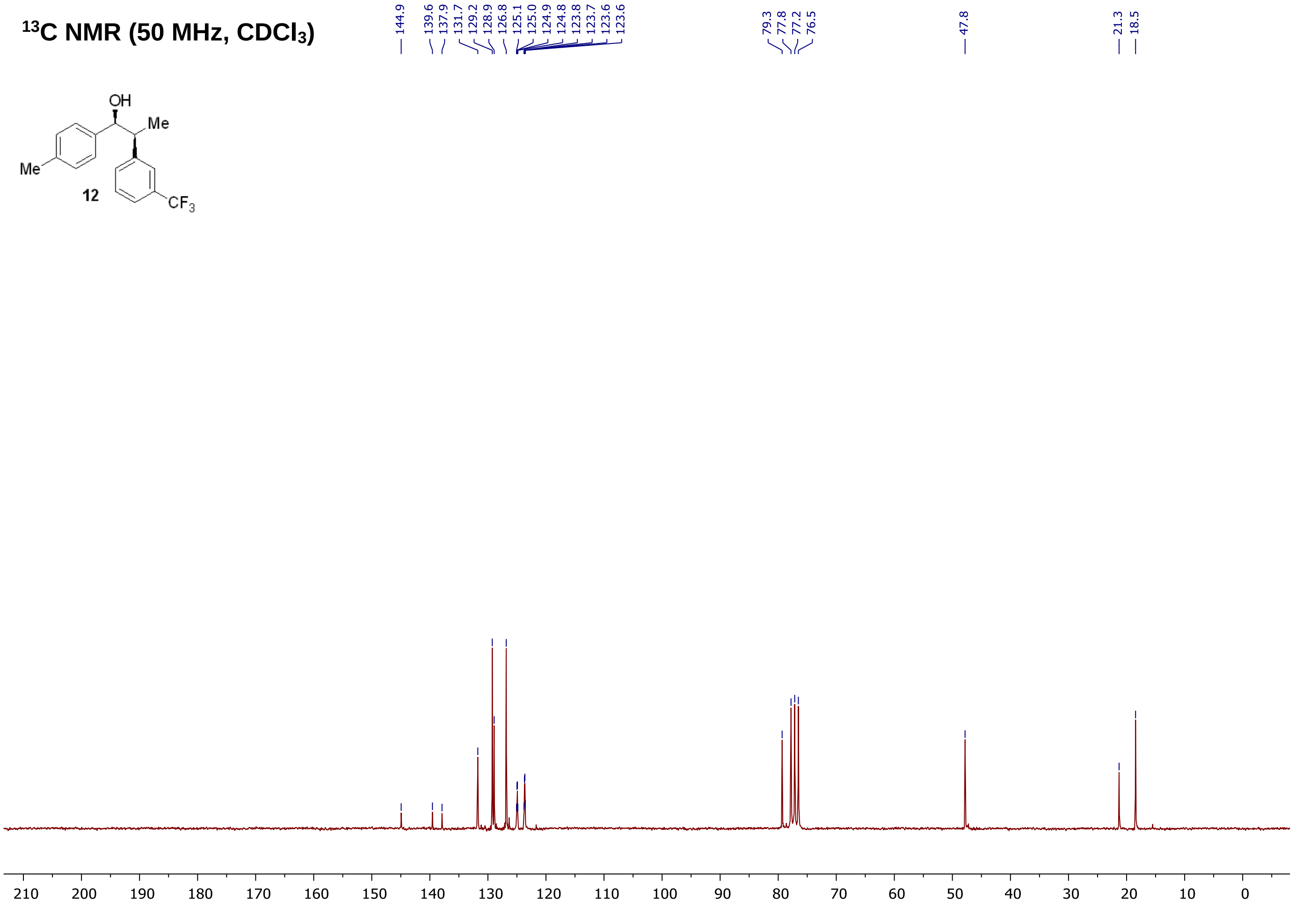

^{19}F NMR (188 MHz, $CDCl_3$)

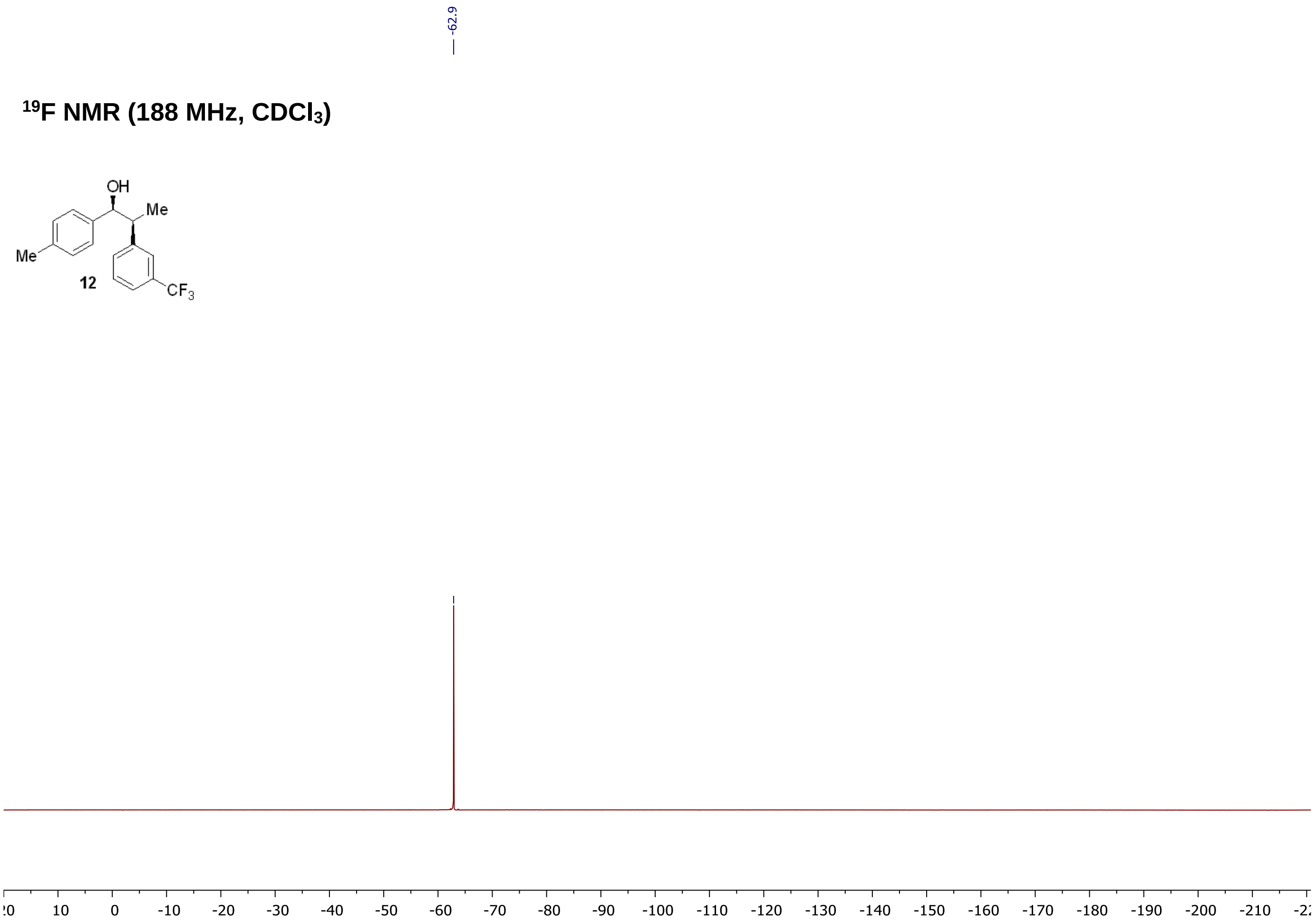

^{1}H NMR (200 MHz, $CDCl_3$)

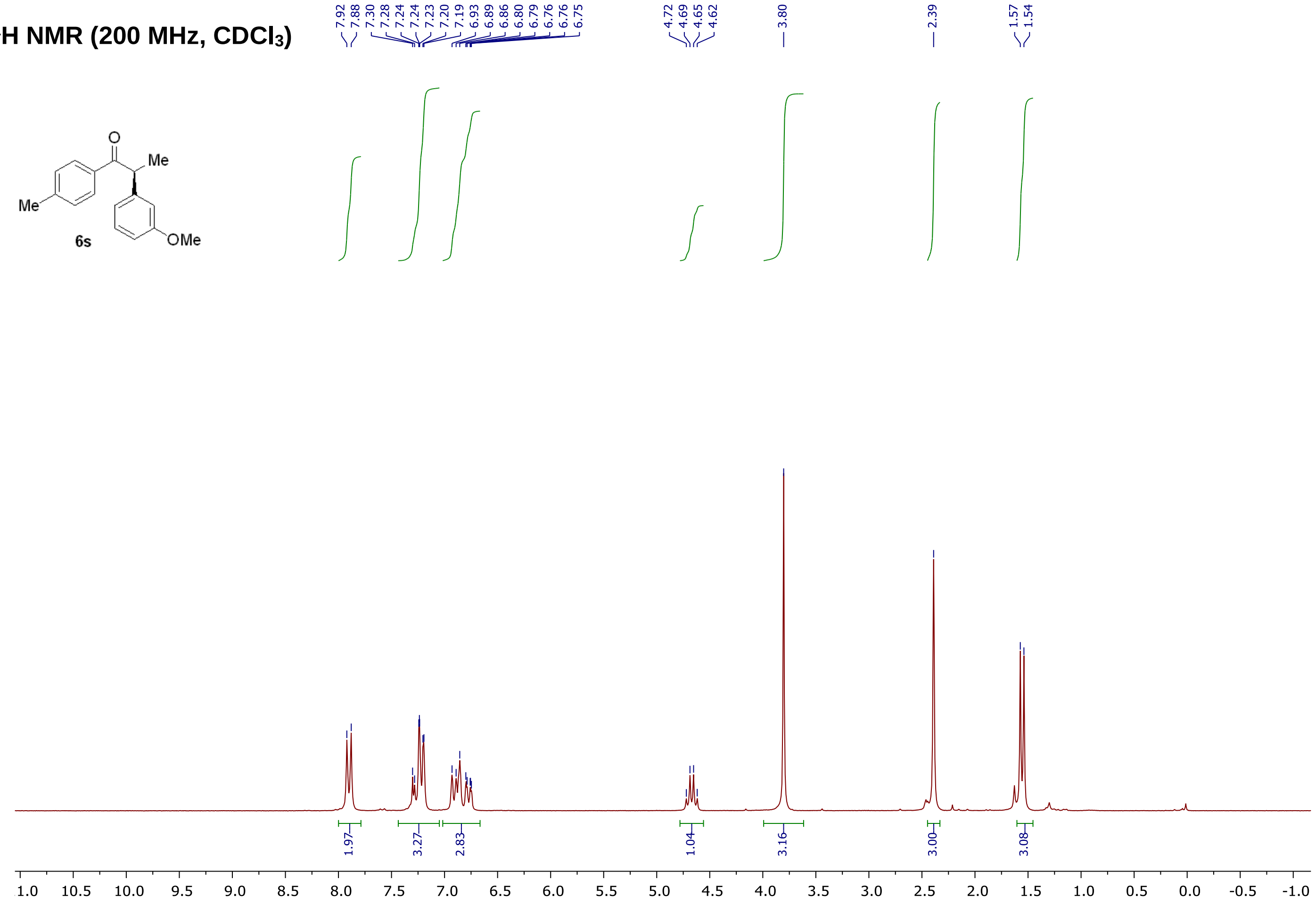

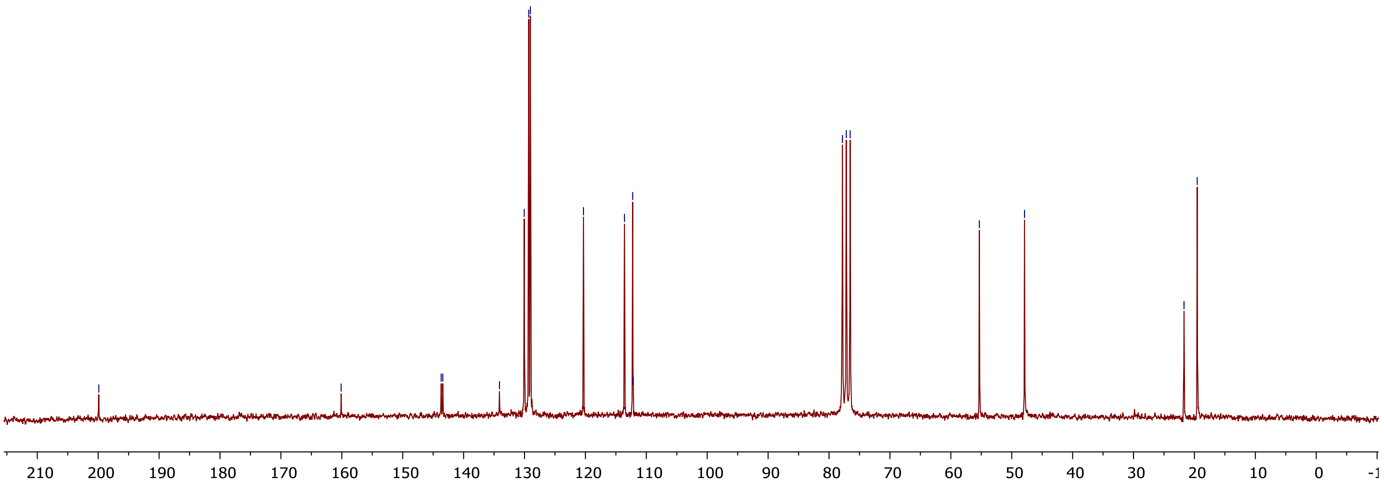

^{13}C NMR (50 MHz, $CDCl_3$)

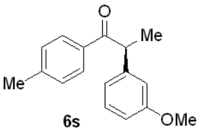

6s

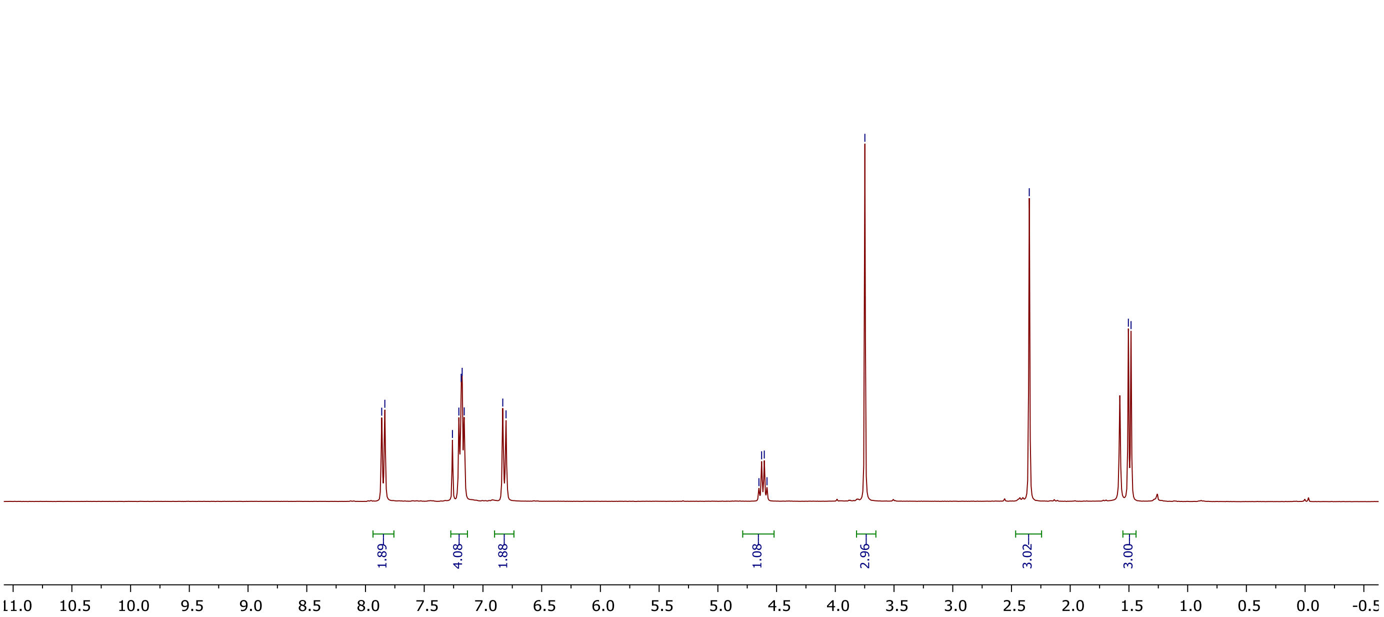

^{1}H NMR (300 MHz, $CDCl_3$)

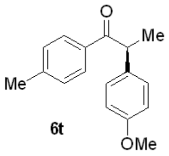

6t

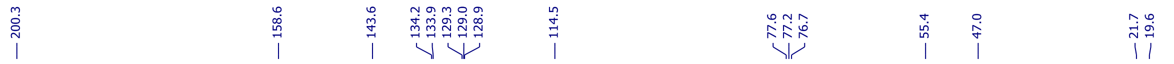

^{13}C NMR (75 MHz, $CDCl_3$)

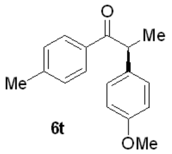

6t

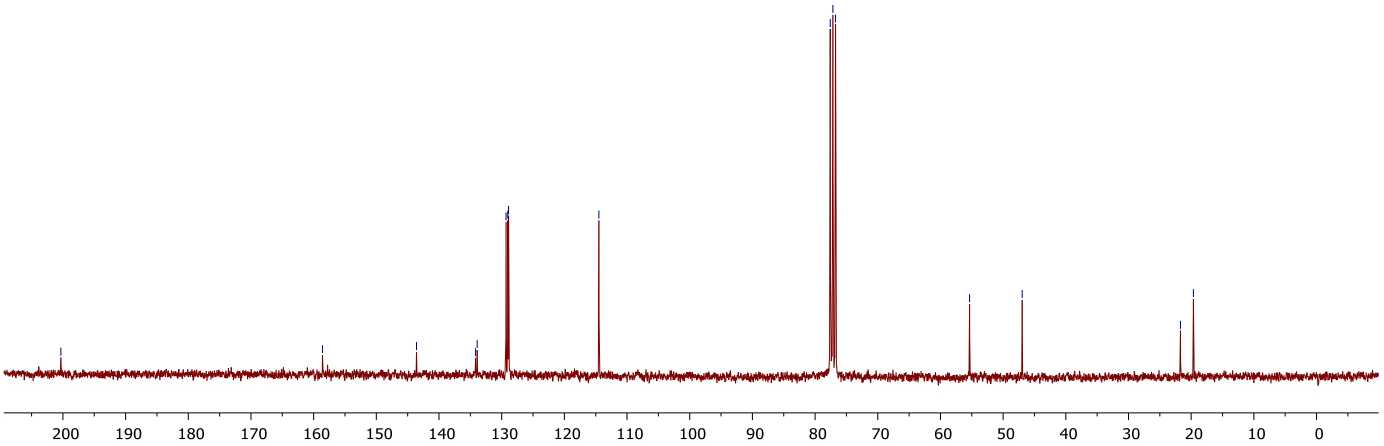

^{1}H NMR (300 MHz, $CDCl_3$)

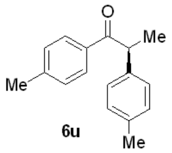

6u

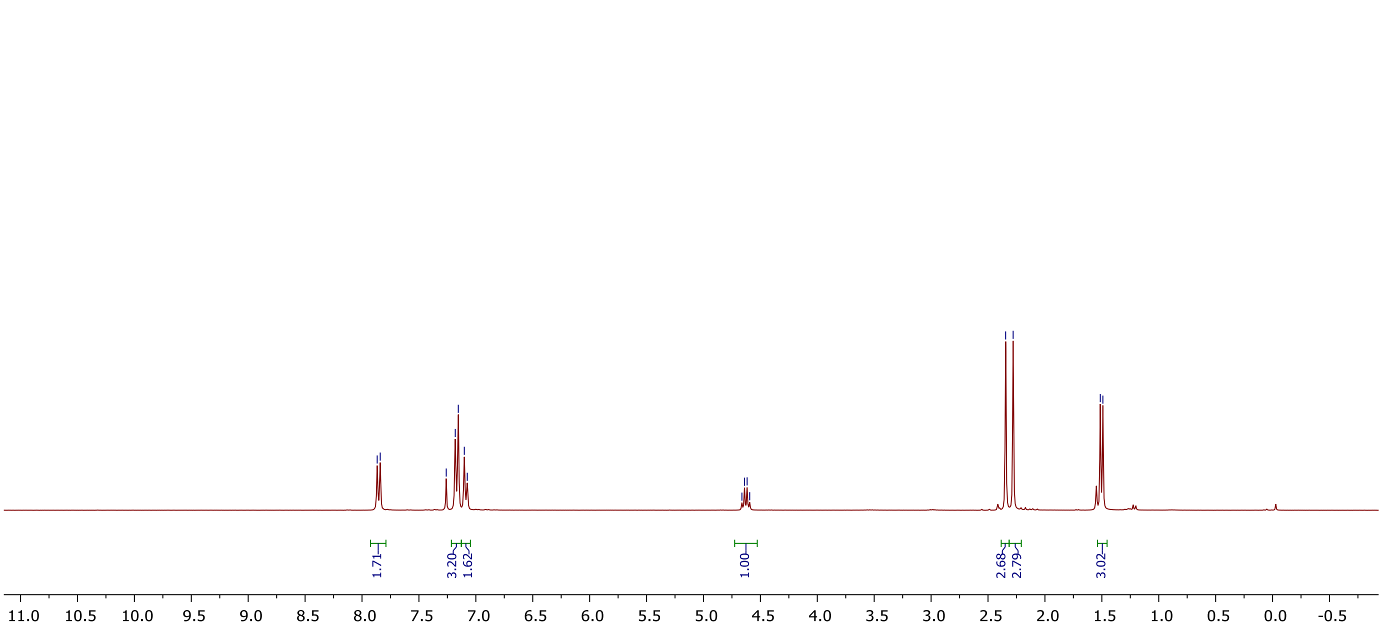

^{13}C NMR (75 MHz, $CDCl_3$)

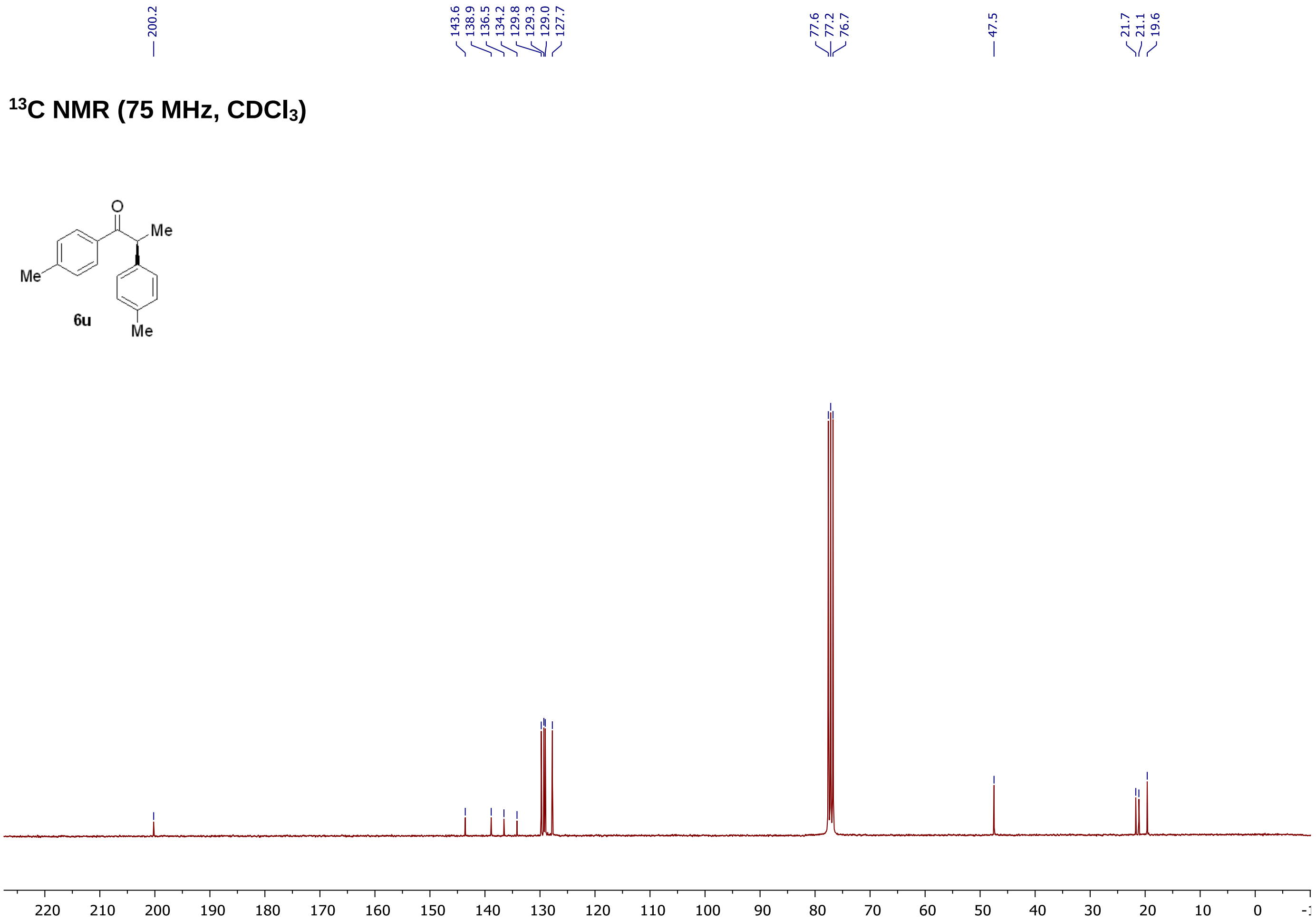

^{1}H NMR (300 MHz, $CDCl_3$)

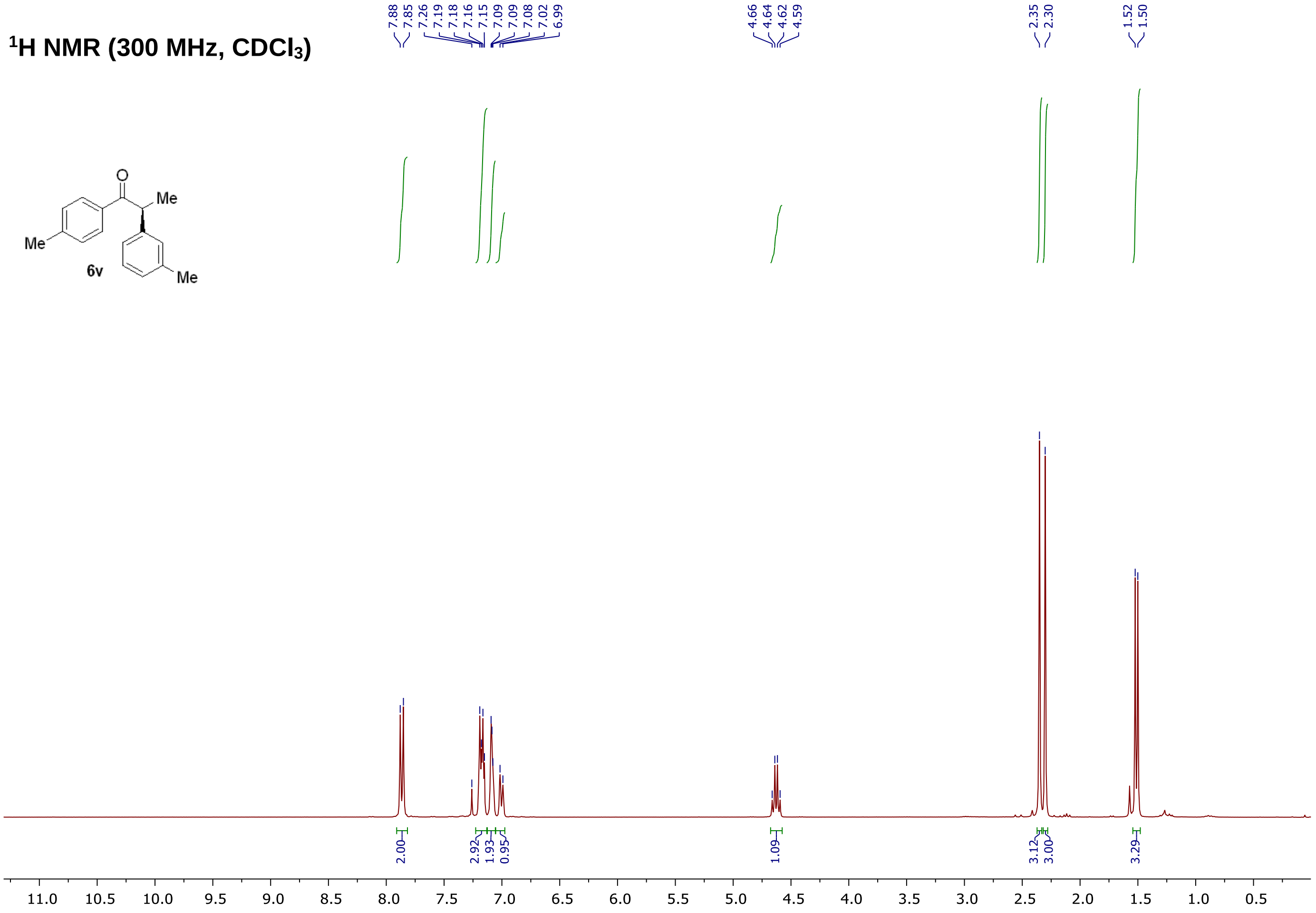

^{13}C NMR (75 MHz, CDCl$_3$)

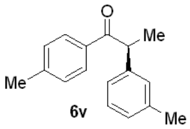

6v

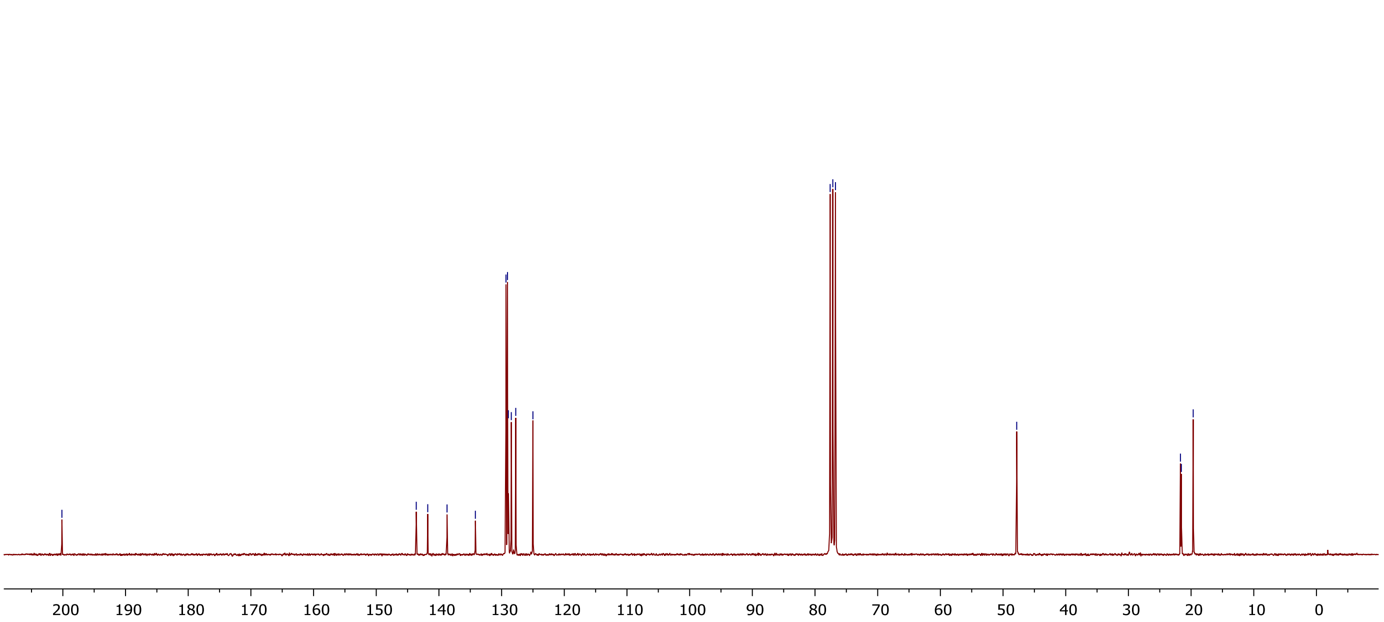

^{1}H NMR (300 MHz, CDCl$_3$)

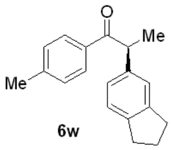

6w

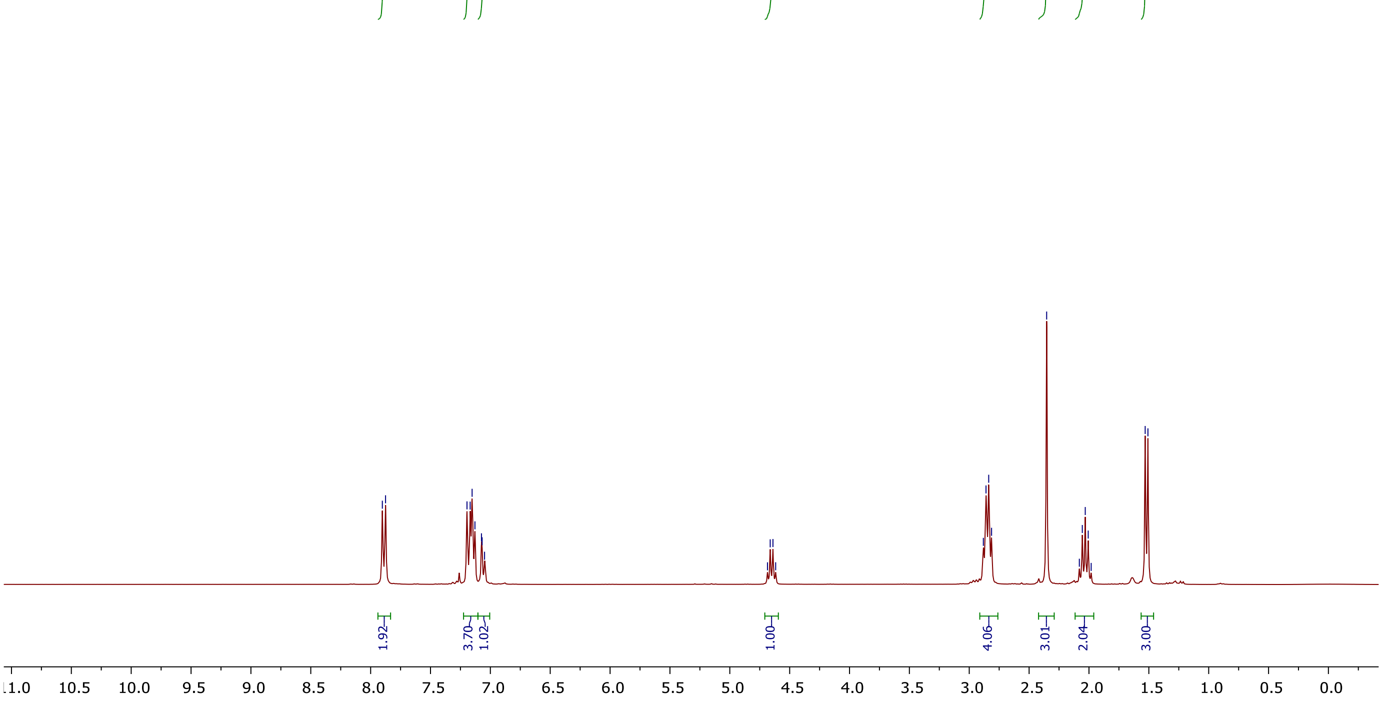

^{13}C NMR (75 MHz, $CDCl_3$)

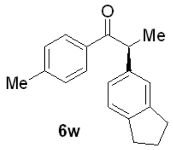

6w

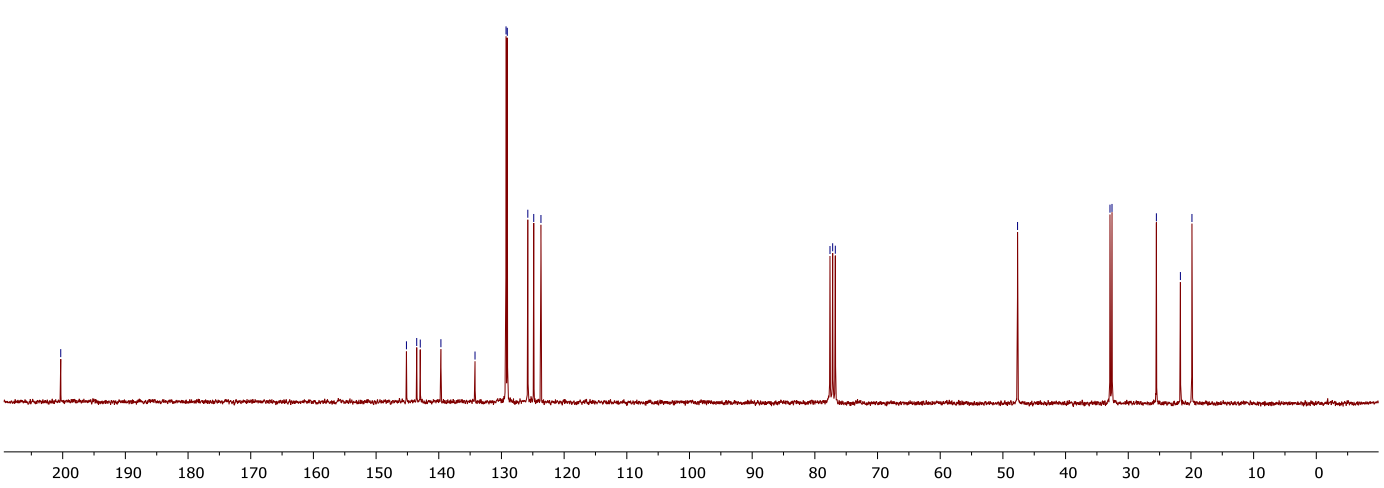

^{1}H NMR (200 MHz, $CDCl_3$)

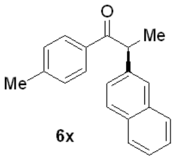

6x

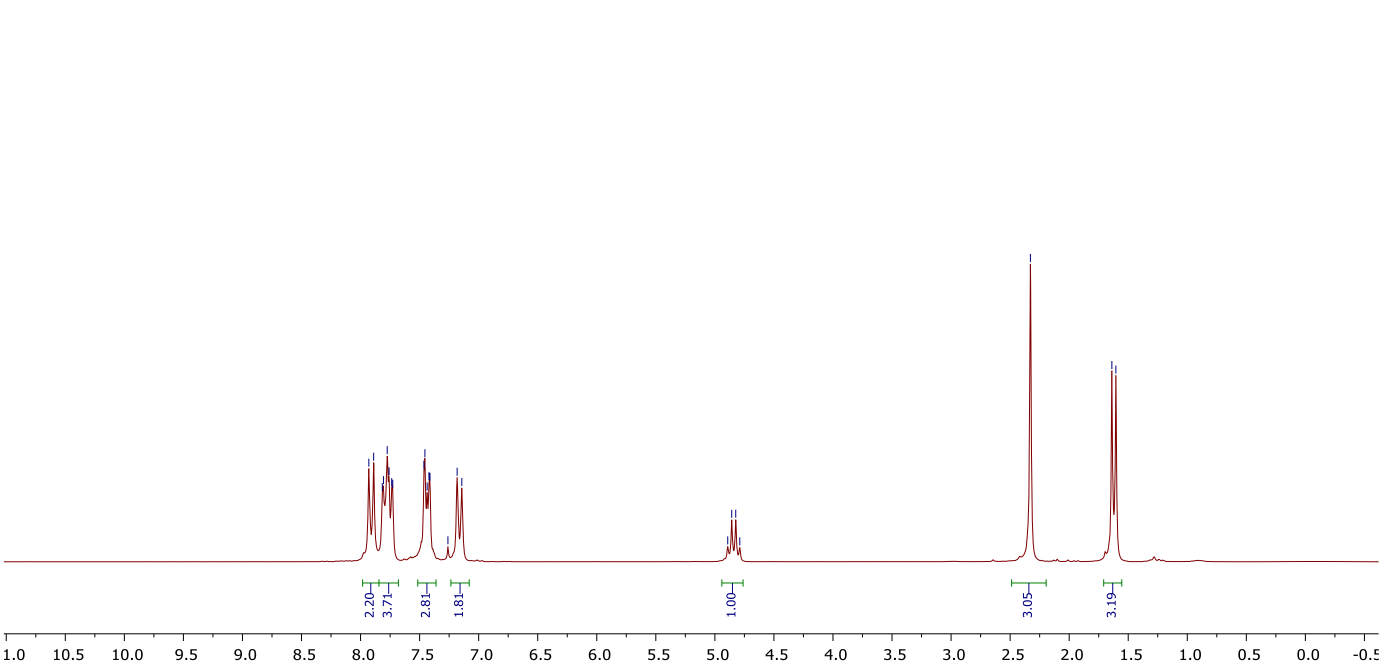

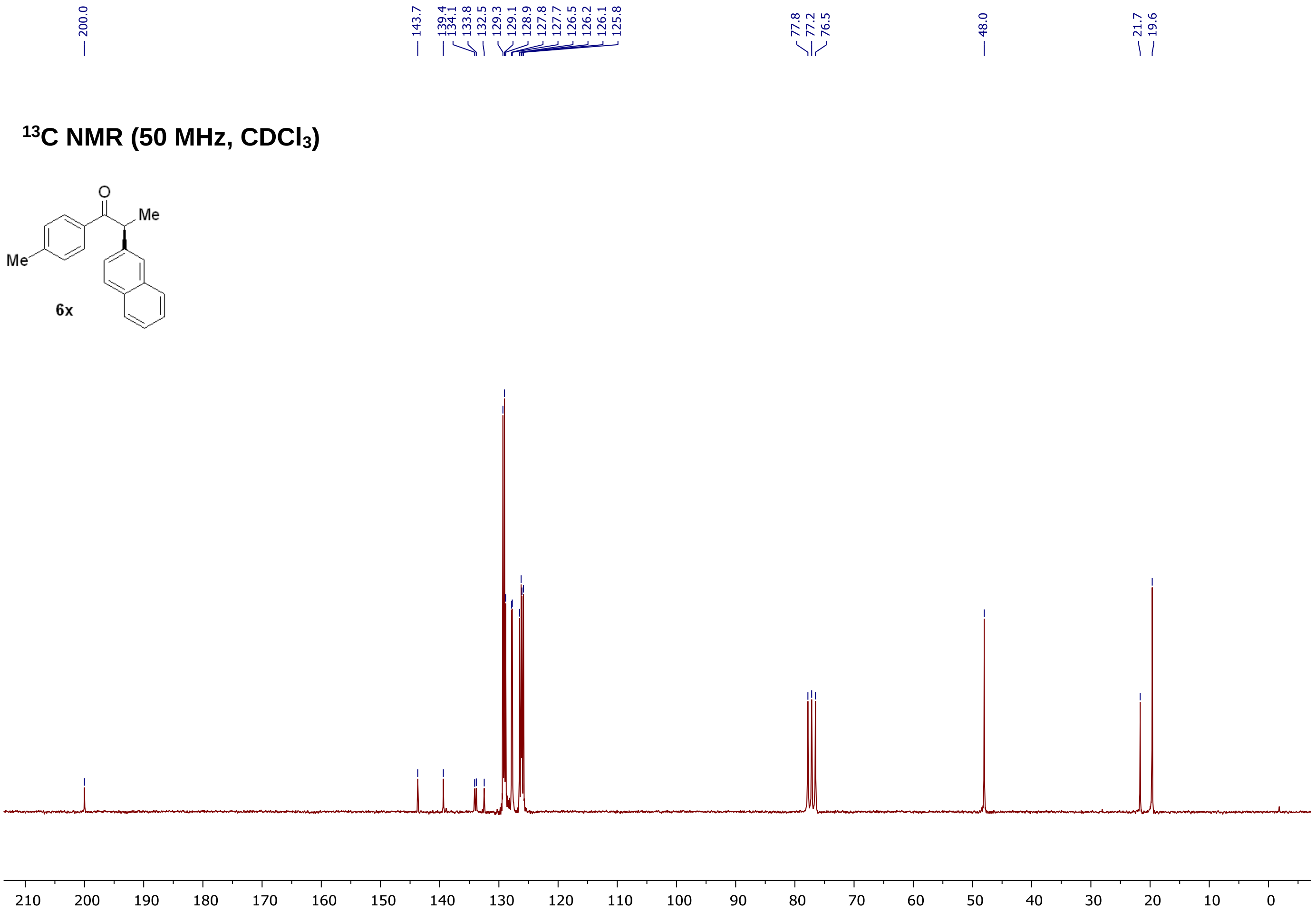
13C NMR (50 MHz, CDCl3)
Me
Me
6x
200.0
143.7
139.4
134.1
133.8
132.5
129.3
129.1
128.9
127.8
127.7
126.5
126.2
126.1
125.8
77.8
77.2
76.5
48.0
21.7
19.6

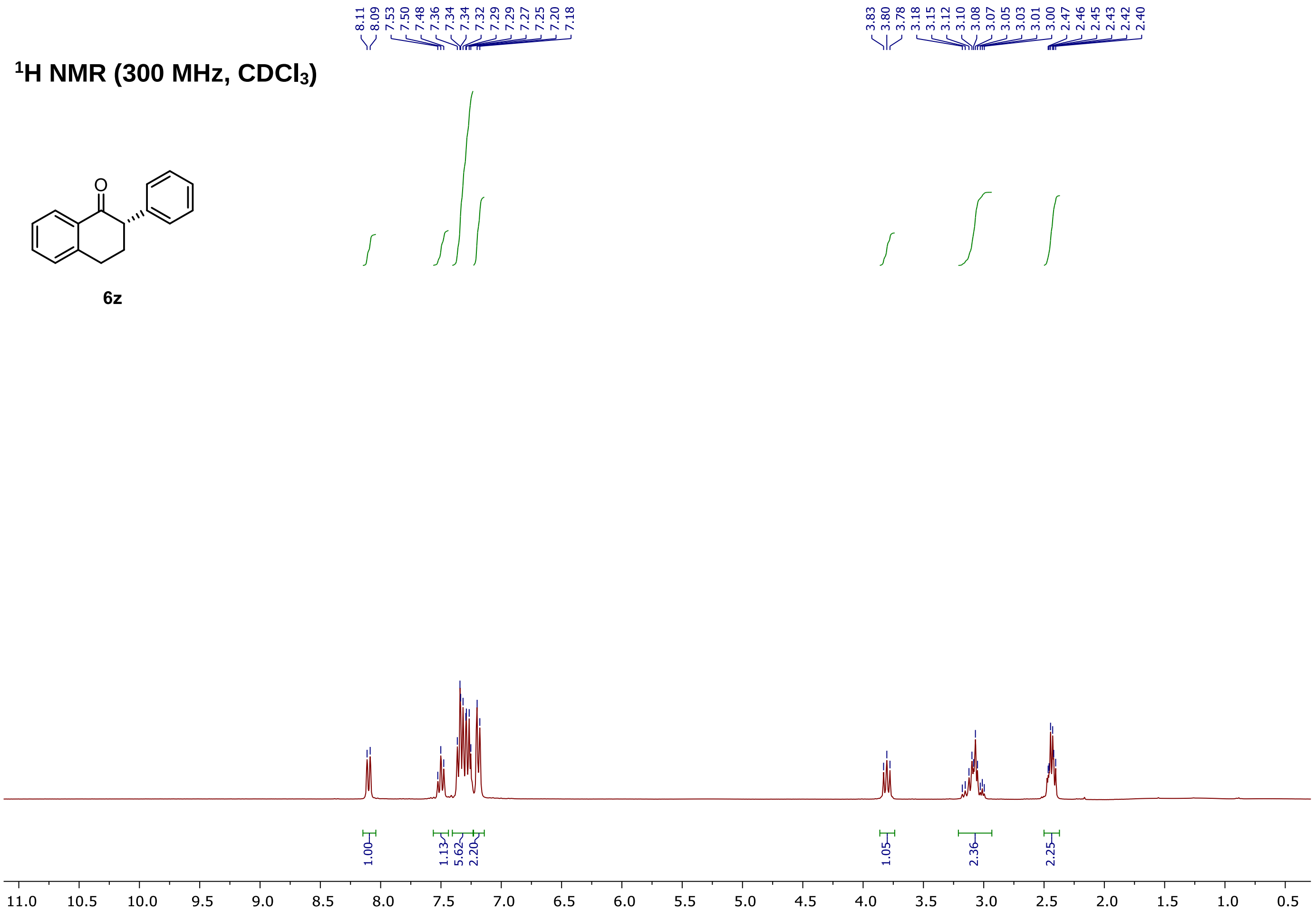
1H NMR (300 MHz, CDCl3)
6z
1.00
1.13
5.62
2.20
1.05
2.36
2.25

^{13}C NMR (75 MHz, $CDCl_3$)

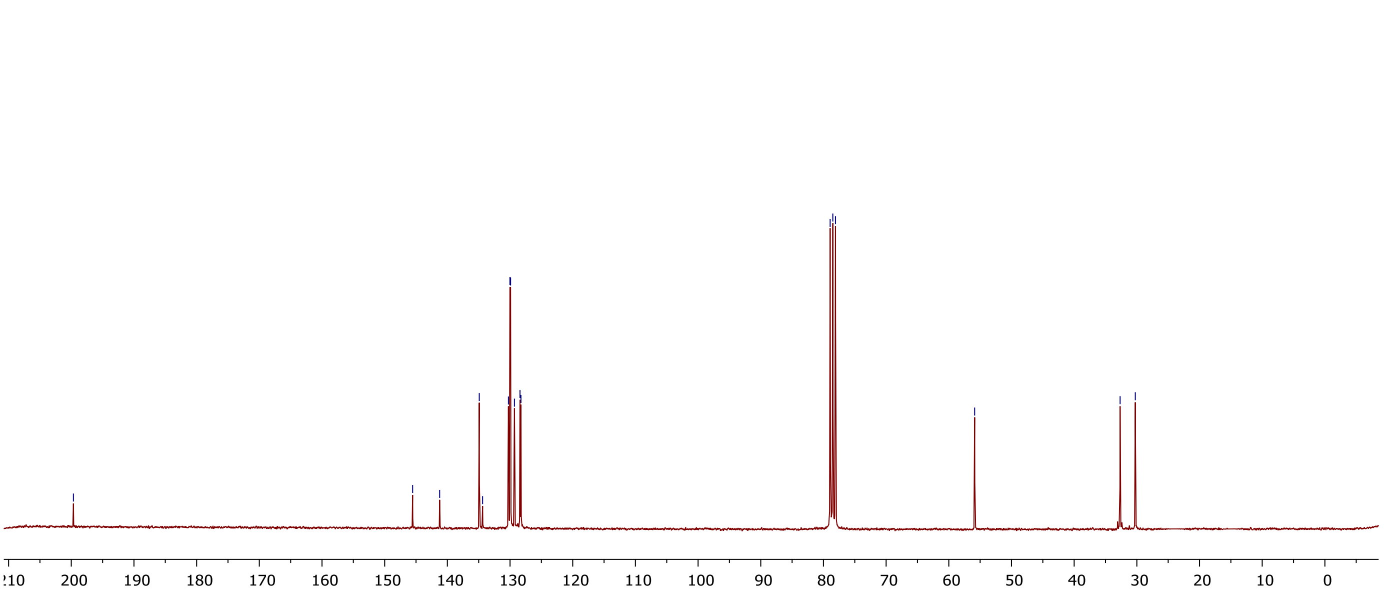

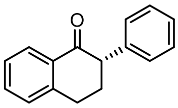

6z

^{1}H NMR (300 MHz, $CDCl_3$)

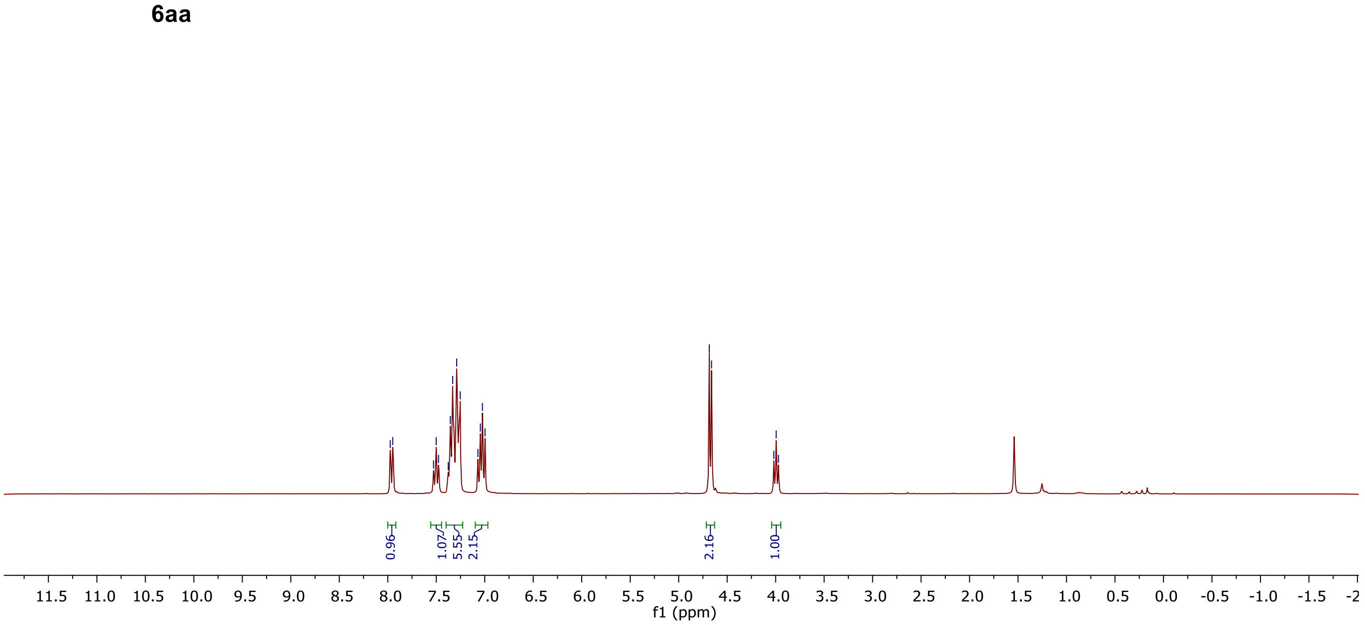

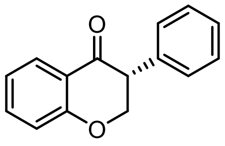

6aa

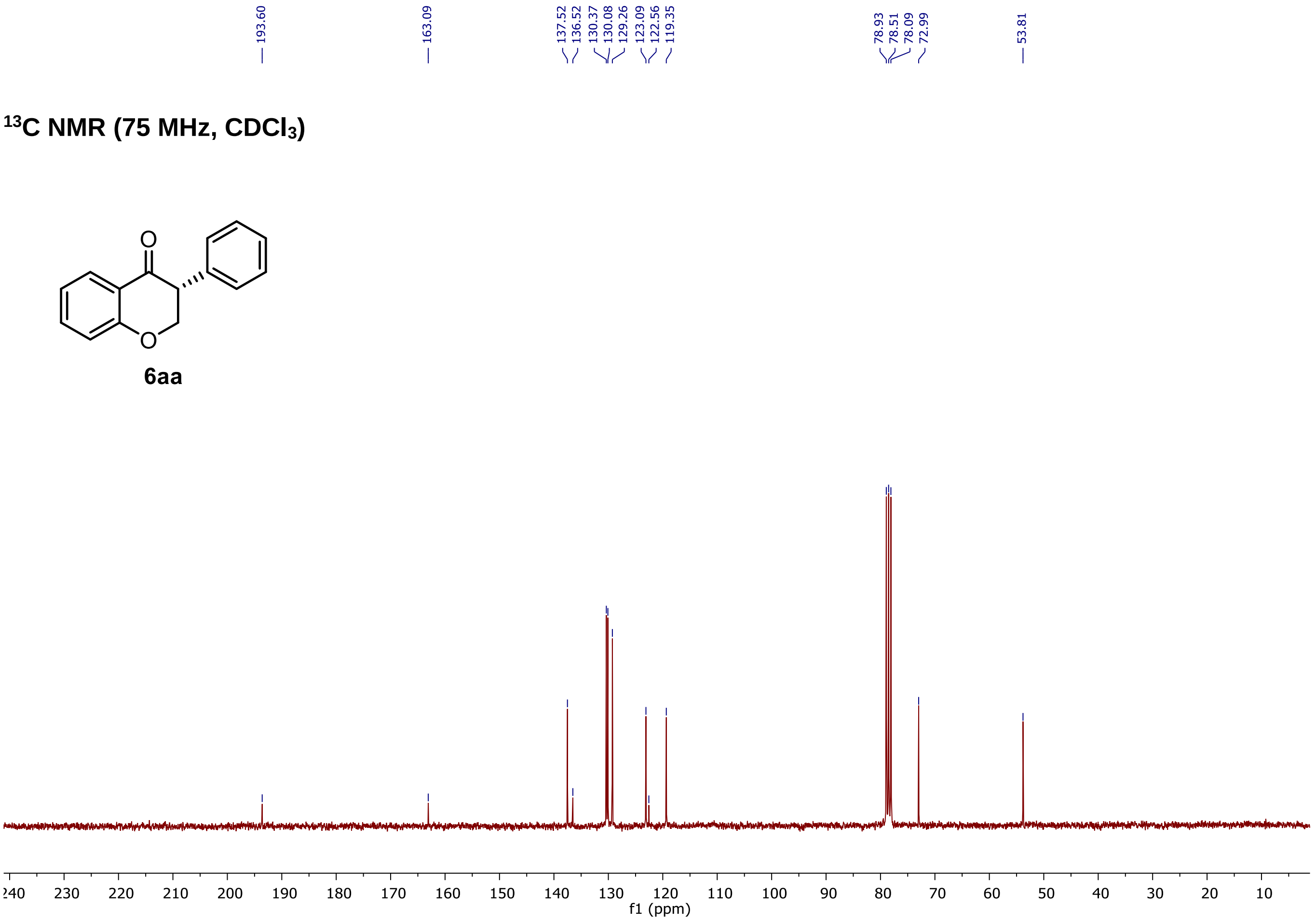
193.60
163.09
137.52
136.52
130.37
130.08
129.26
123.09
122.56
119.35
78.93
78.51
78.09
72.99
53.81
^{13}C NMR (75 MHz, $CDCl_3$)
O
O
6aa
240 230 220 210 200 190 180 170 160 150 140 130 120 110 100 90 80 70 60 50 40 30 20 10
f1 (ppm)

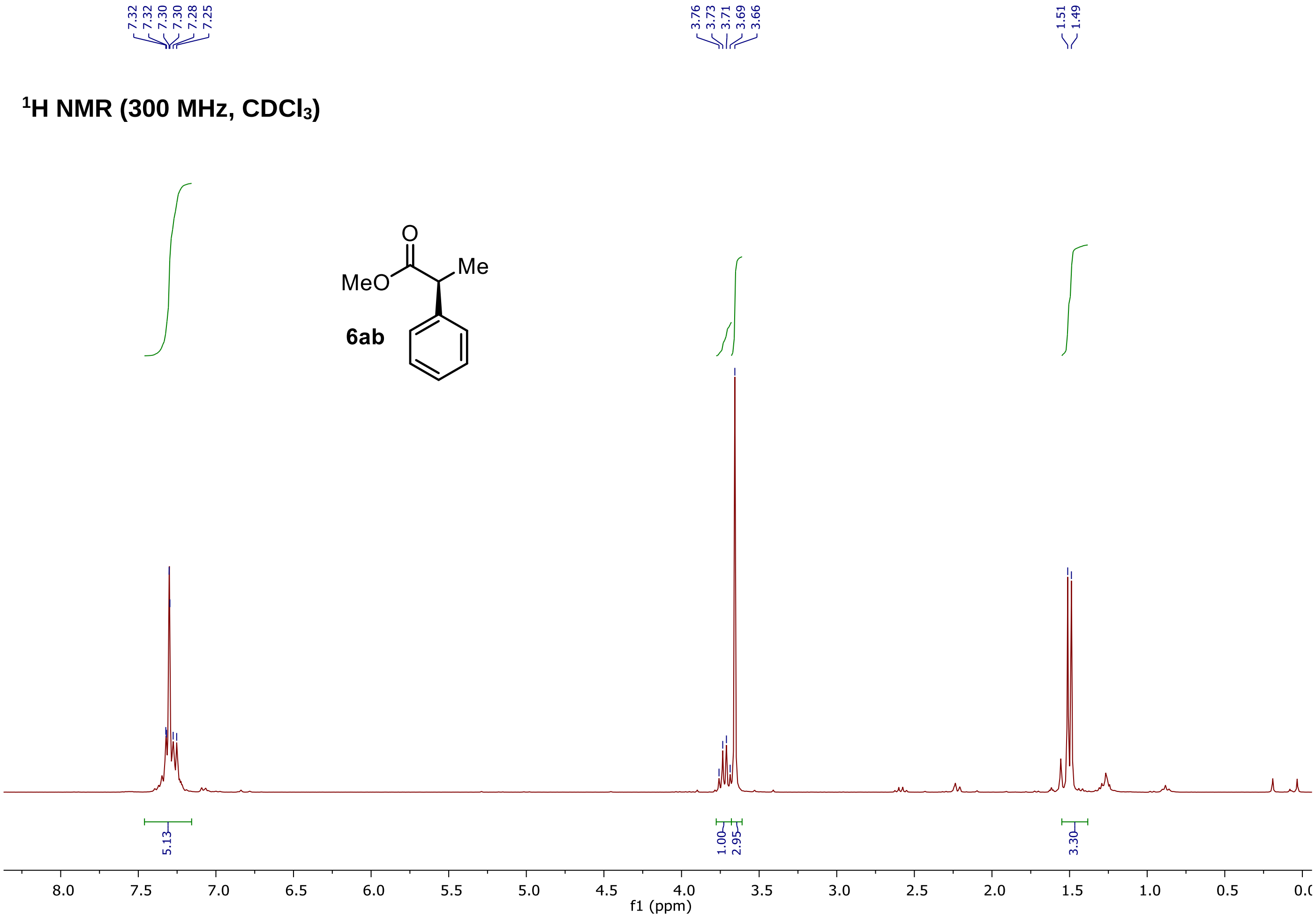
7.32
7.32
7.30
7.30
7.28
7.25
3.76
3.73
3.71
3.69
3.66
1.51
1.49
^{1}H NMR (300 MHz, $CDCl_3$)
O
MeO
Me
6ab
5.13
1.00
2.95
3.30
8.0 7.5 7.0 6.5 6.0 5.5 5.0 4.5 4.0 3.5 3.0 2.5 2.0 1.5 1.0 0.5
f1 (ppm)

^{13}C NMR (75 MHz, $CDCl_3$)

6ab

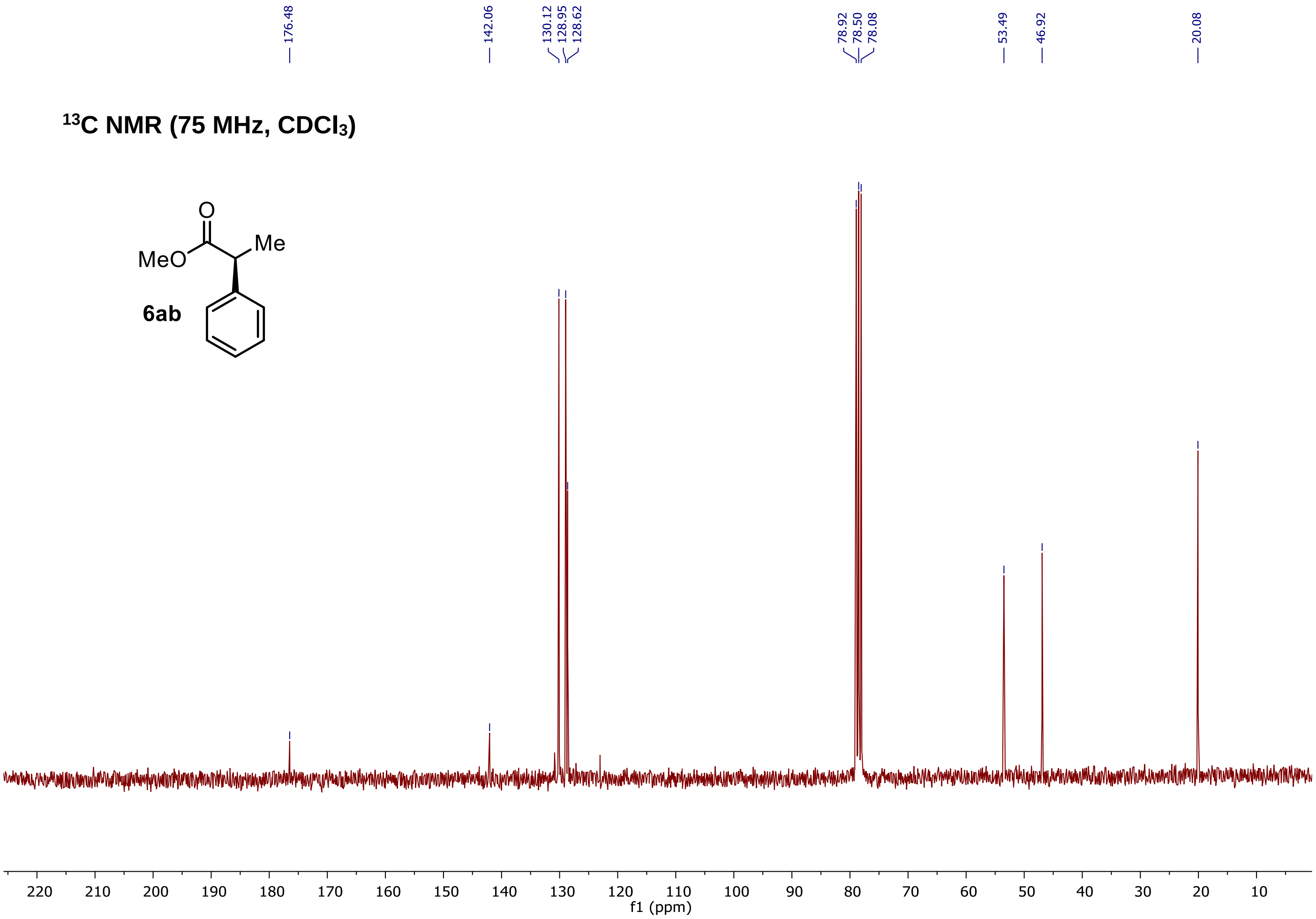

^{1}H NMR (300 MHz, $CDCl_3$)

6ac

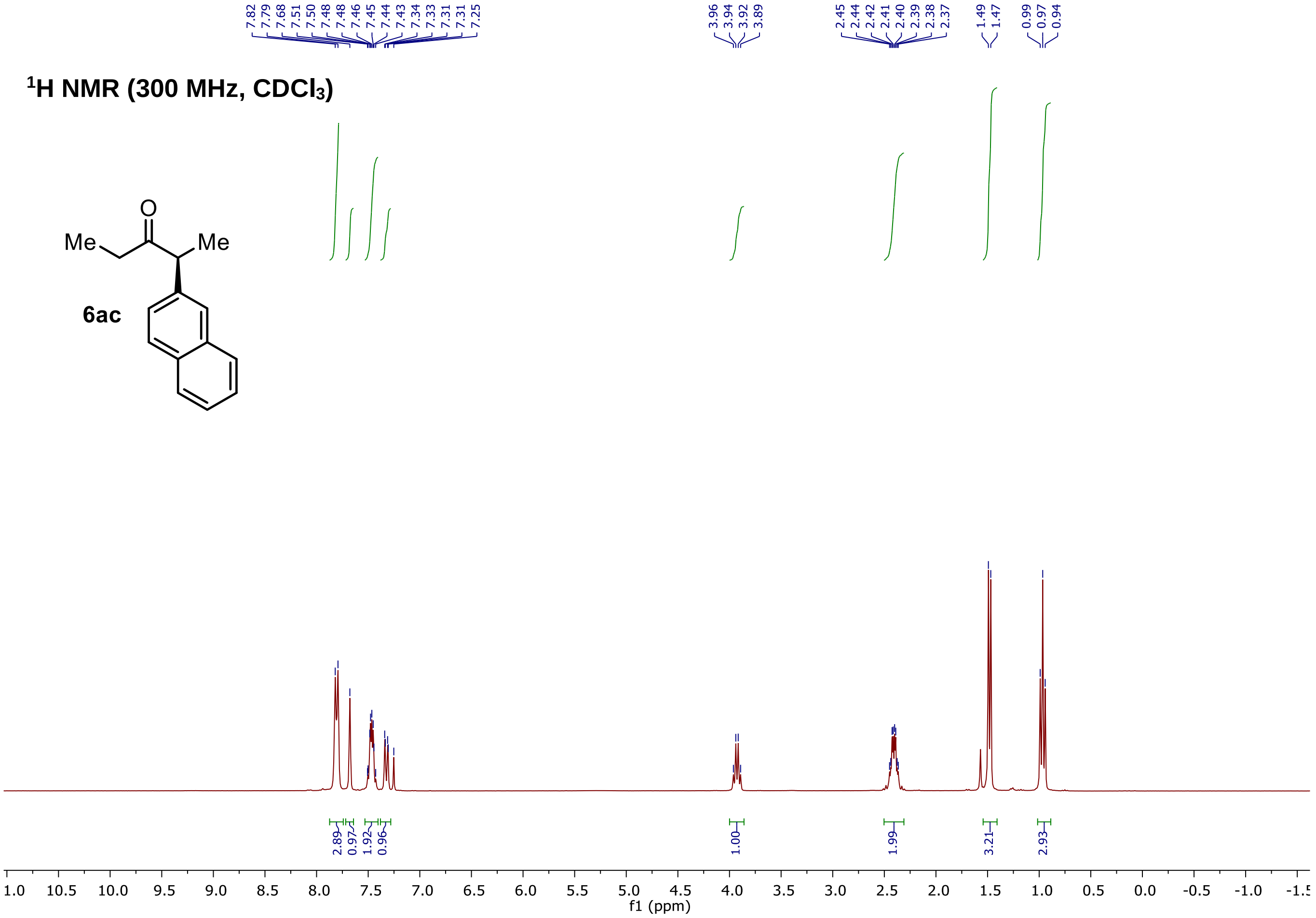

^{13}C NMR (75 MHz, $CDCl_3$)

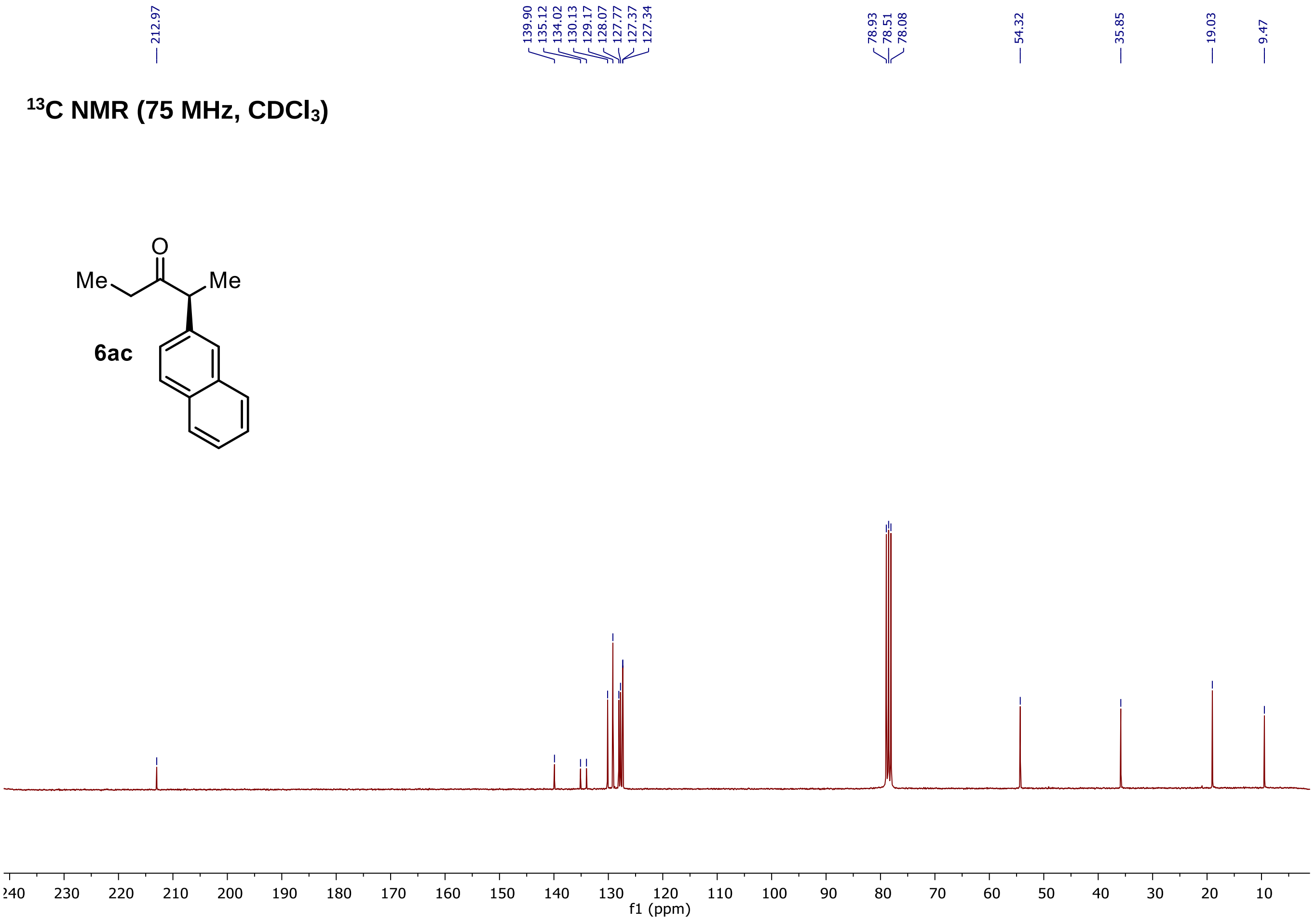

^{1}H NMR (200 MHz, $CDCl_3$)

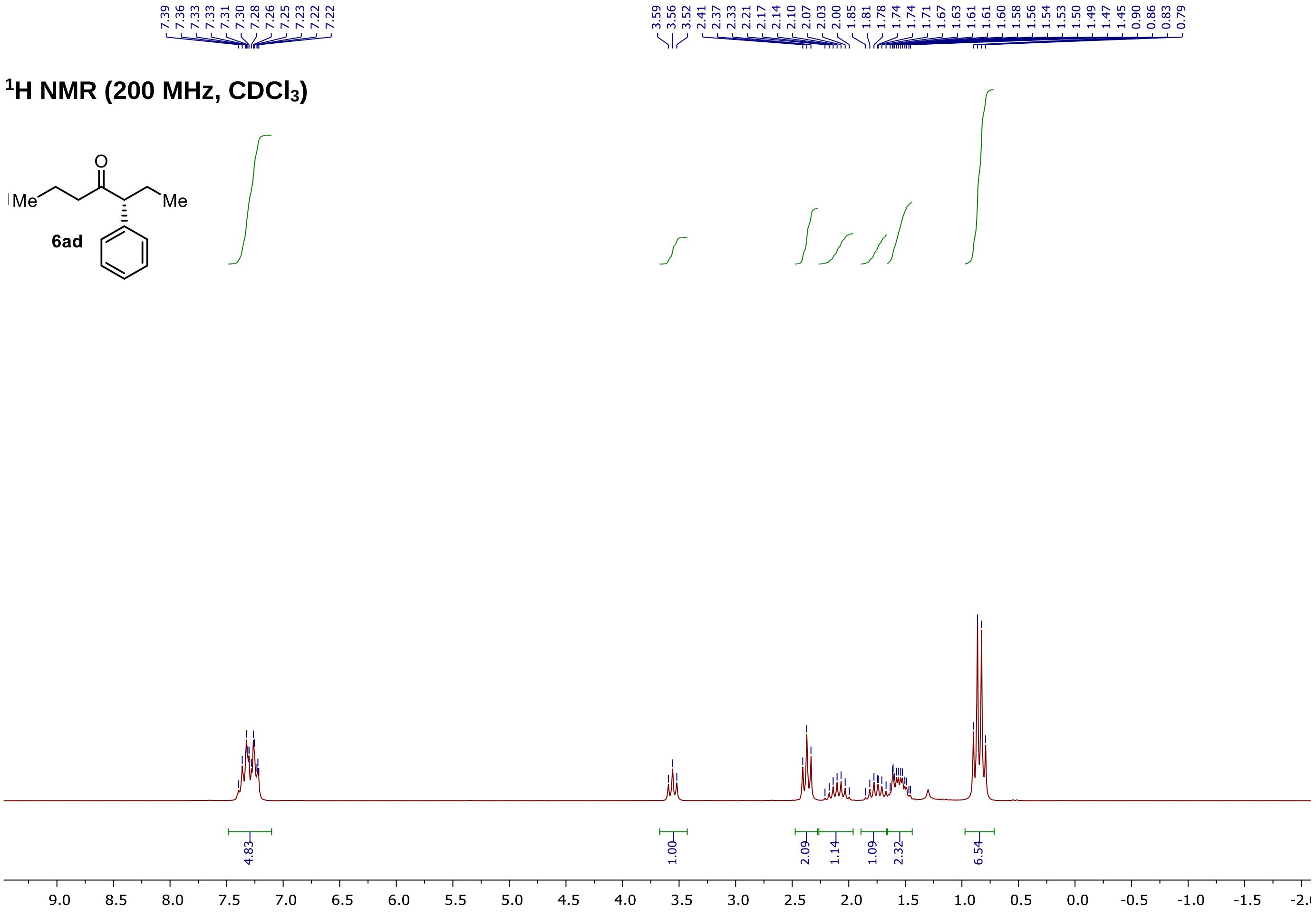

^{13}C NMR (50 MHz, $CDCl_3$)

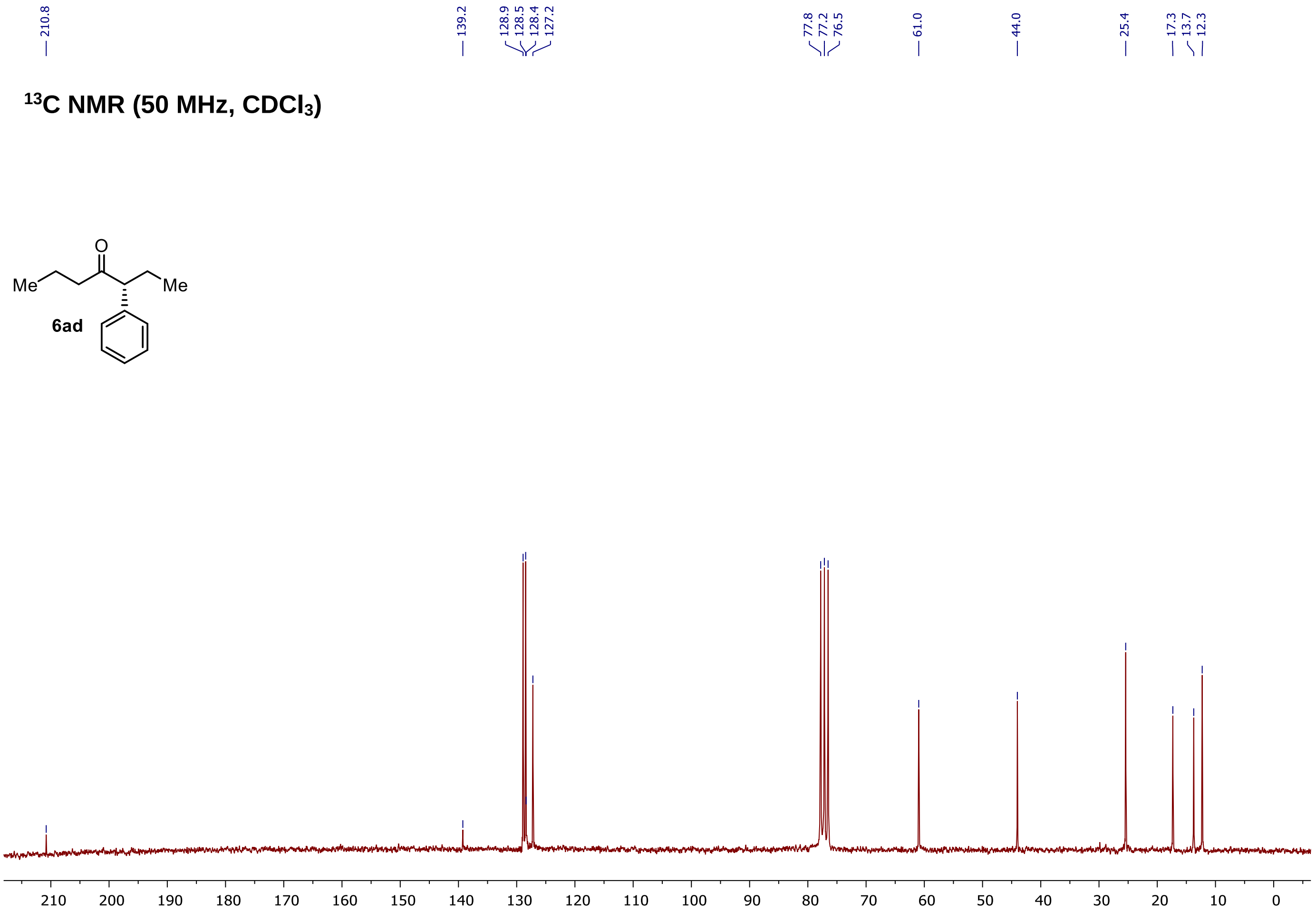

6ad

^{1}H NMR (300 MHz, $CDCl_3$)

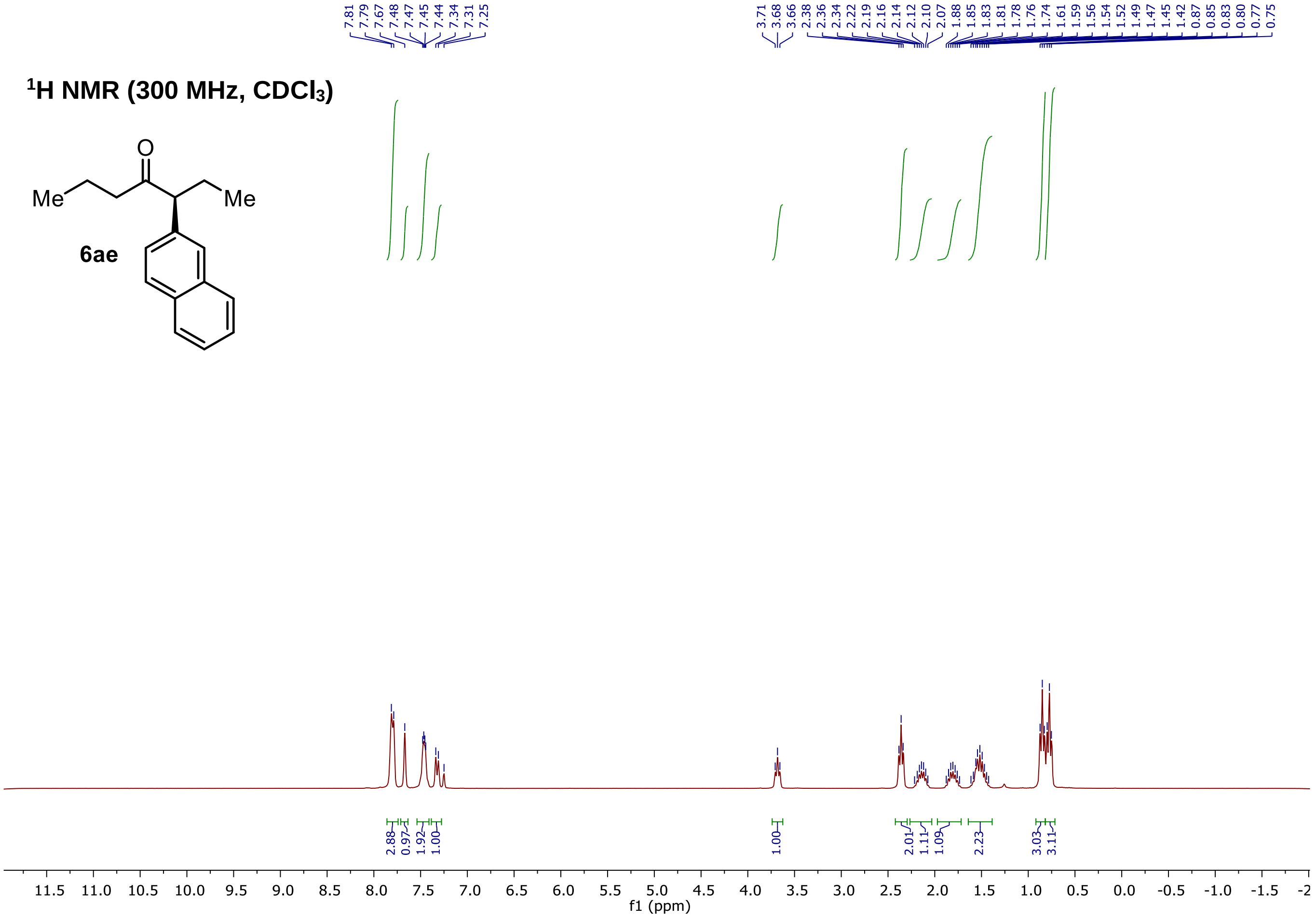

6ae

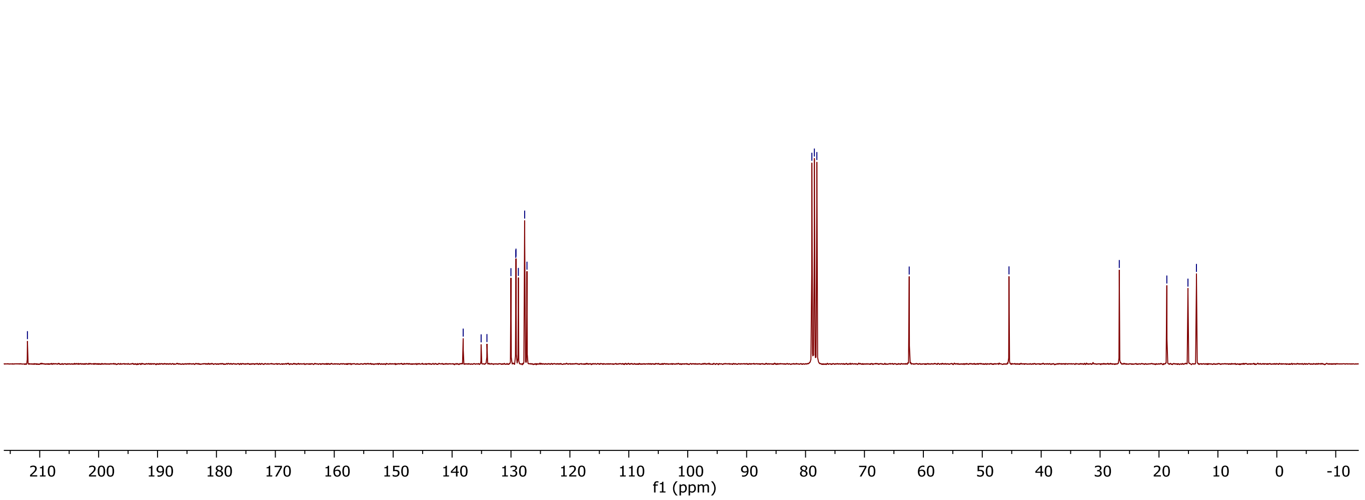

^{13}C NMR (75 MHz, $CDCl_3$)

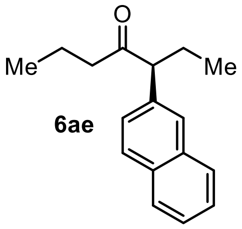

6ae

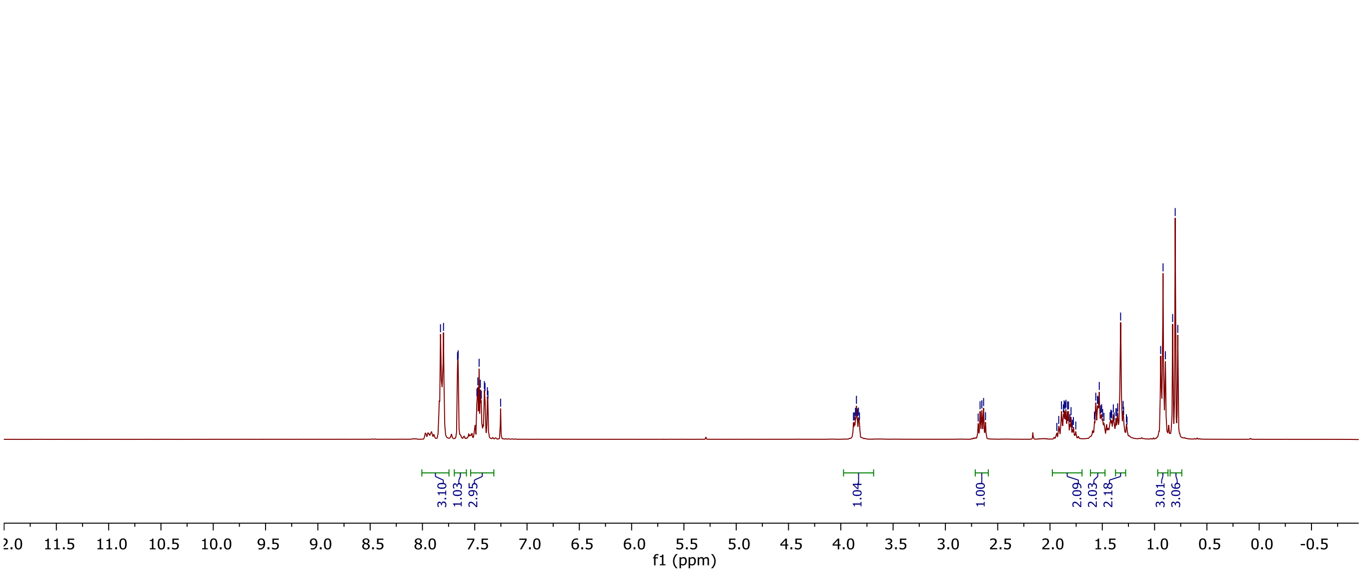

^{1}H NMR (300 MHz, $CDCl_3$)

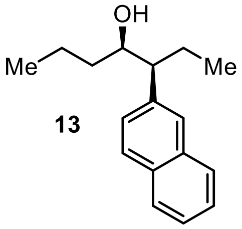

13

^{13}C NMR (75 MHz, $CDCl_3$)

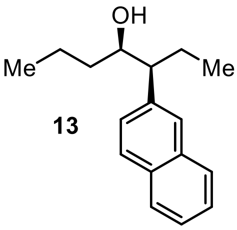

13

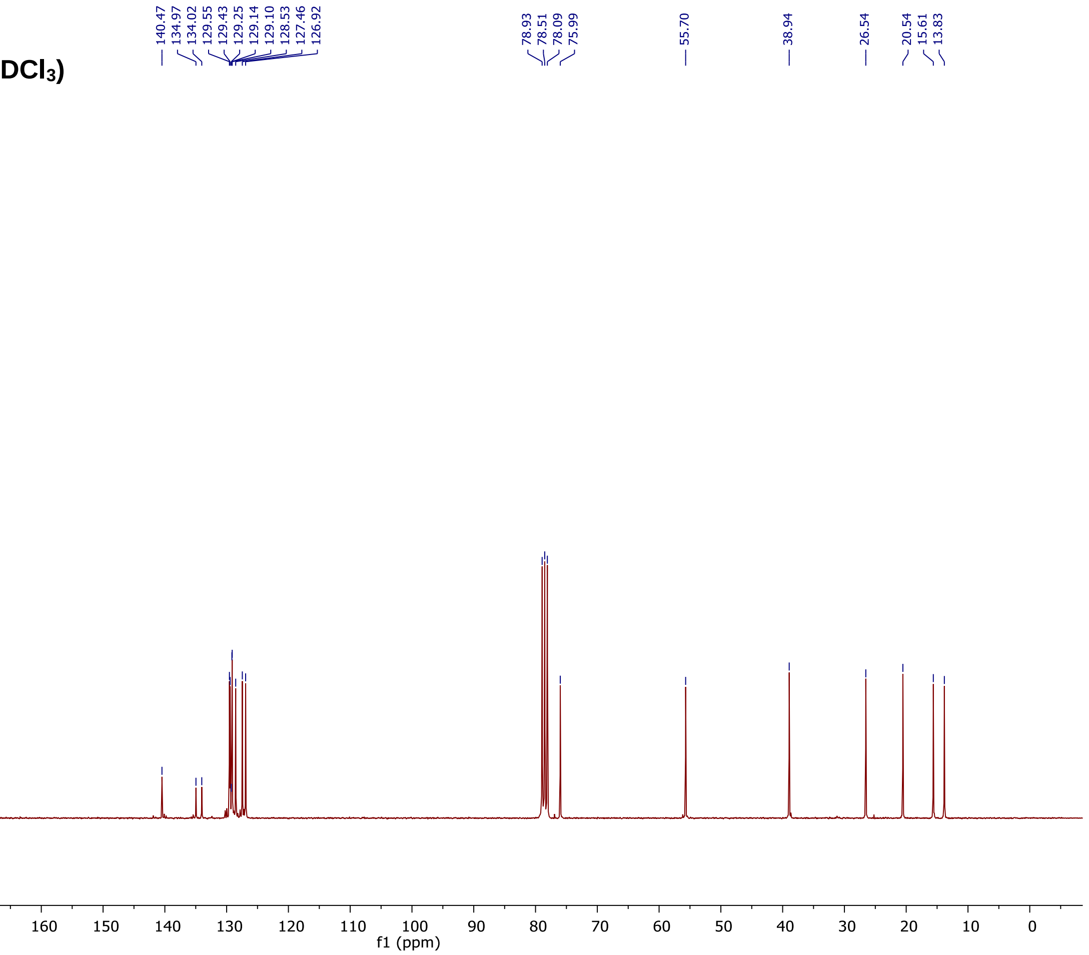

^{1}H NMR (300 MHz, $CDCl_3$)

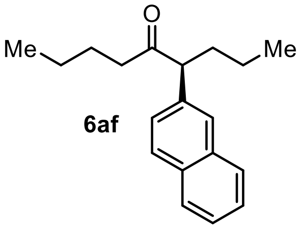

6af

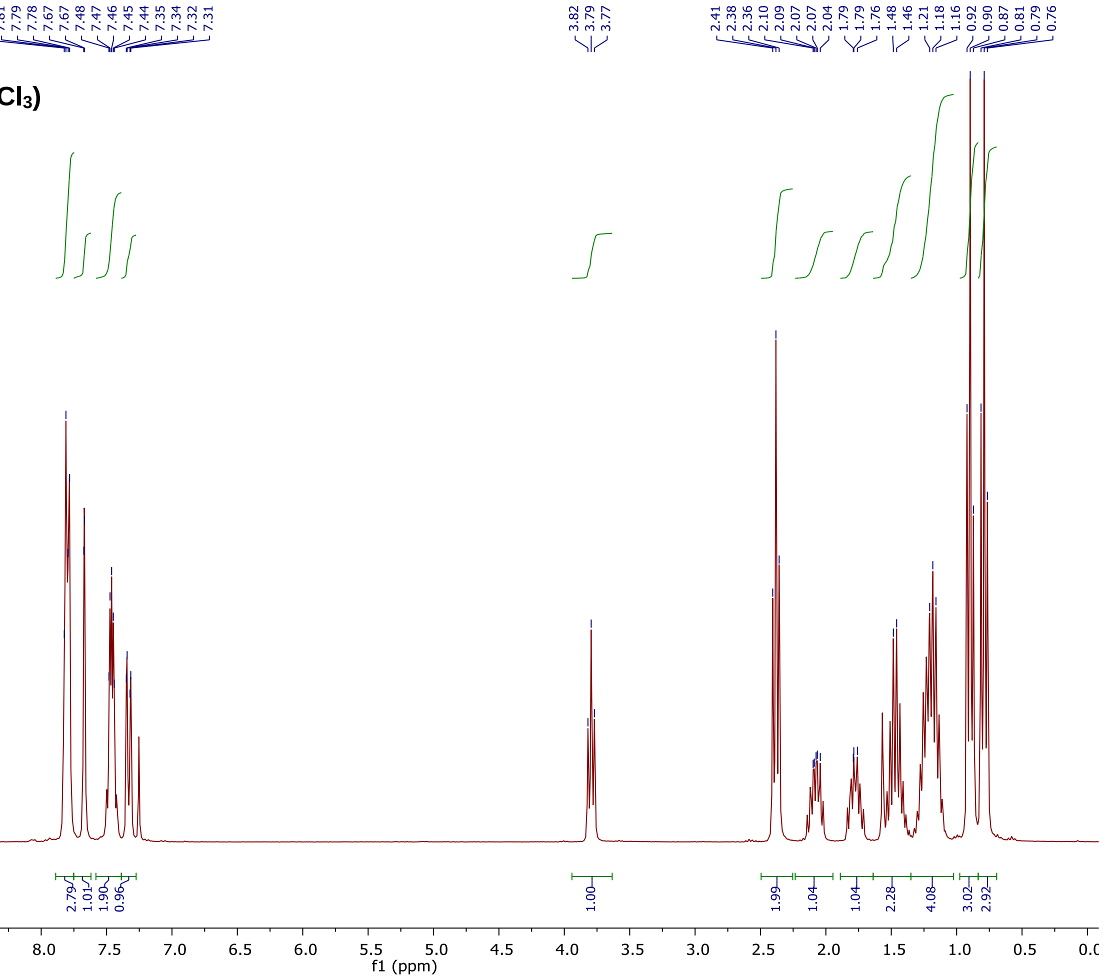

^{13}C NMR (75 MHz, $CDCl_3$)

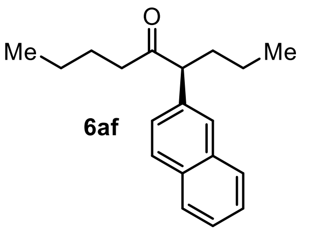

6af

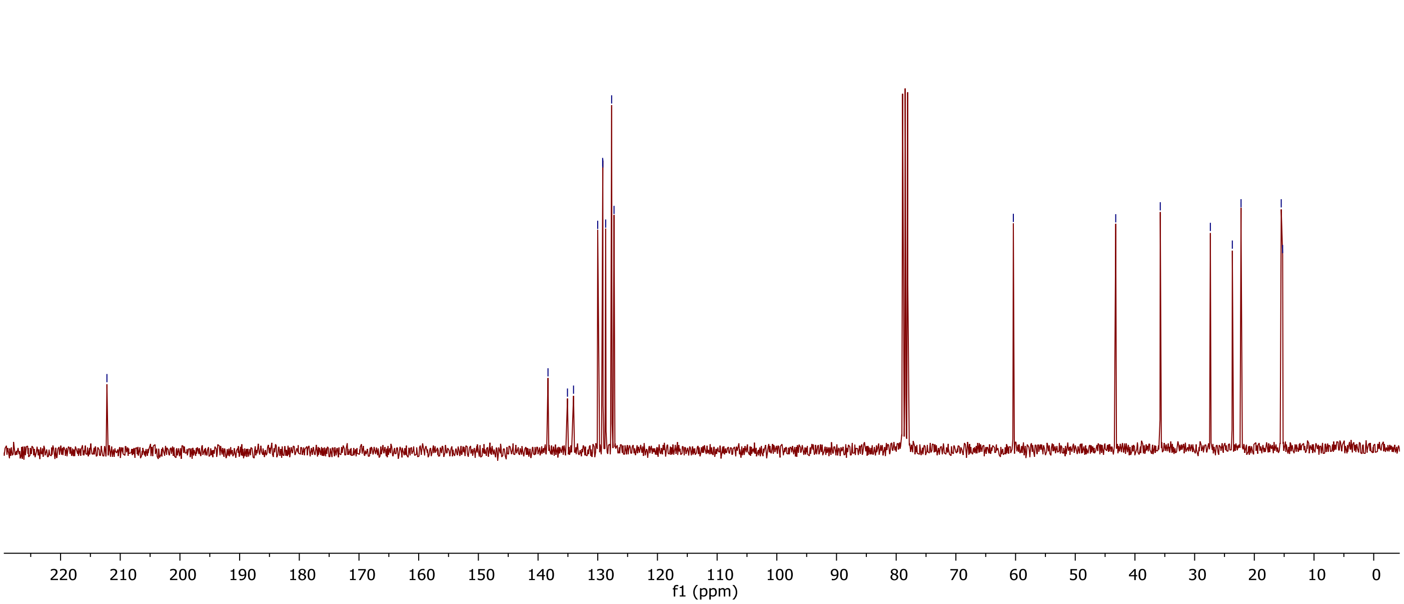

^{1}H NMR (300 MHz, $CDCl_3$)

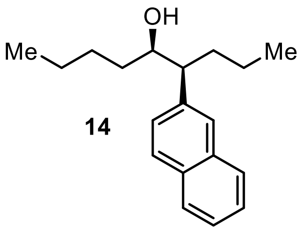

14

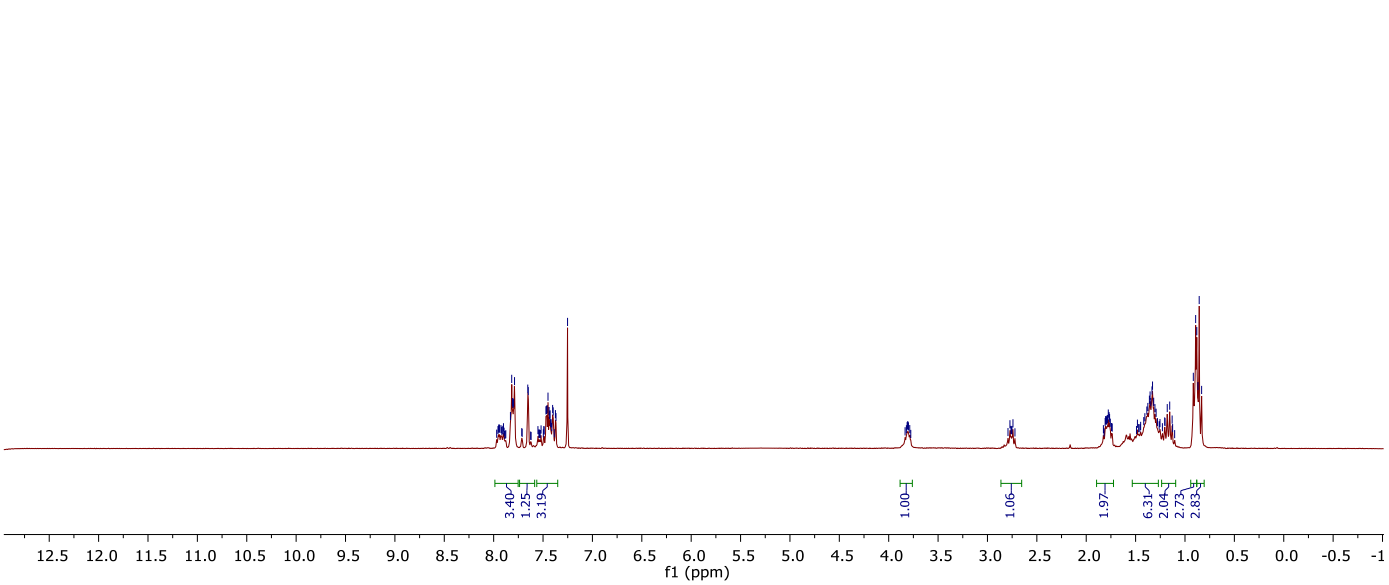

^{13}C NMR (75 MHz, $CDCl_3$)

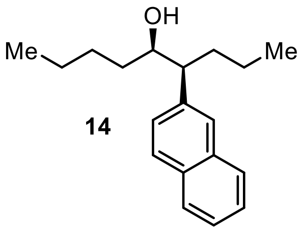

14

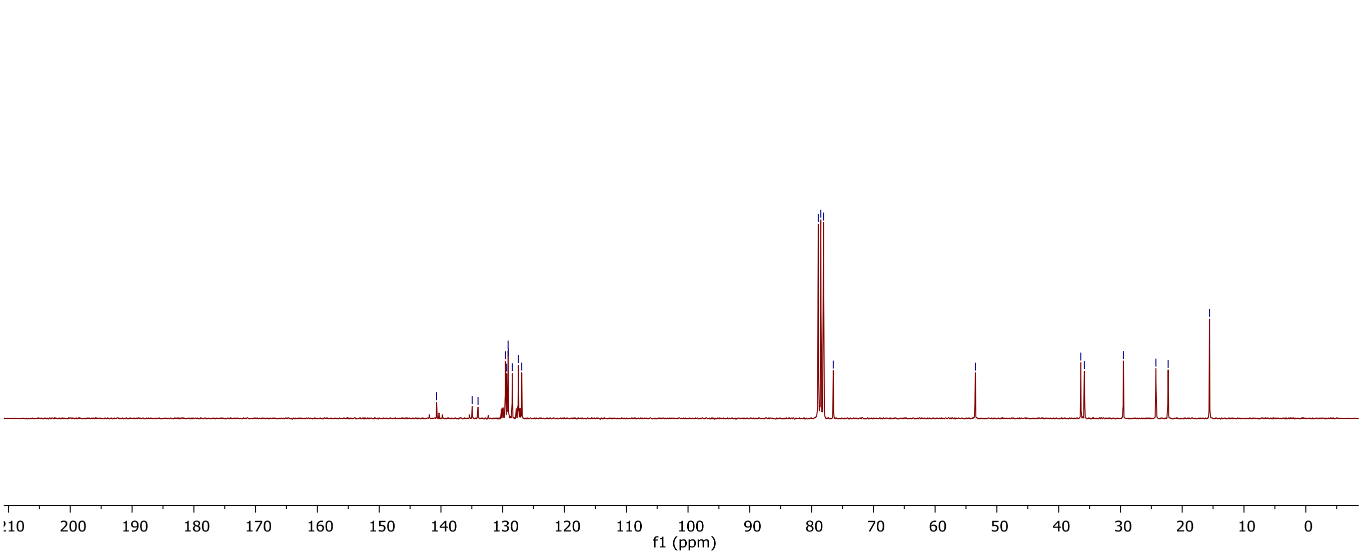

Geometries of ligands

3a

C	-2.17328300	-3.50139200	-3.31164300
C	-1.22011000	-2.66861400	-3.83548900
C	-0.42249800	-1.85426700	-2.98735500
C	-0.62697800	-1.91083400	-1.58089700
C	-1.61185300	-2.80056400	-1.06555800
C	-2.36707200	-3.57044900	-1.90966700
H	0.72488800	-0.94493400	-4.57769600
H	-2.78272100	-4.11506400	-3.96834100
H	-1.06027600	-2.61303600	-4.90932700
C	0.56323900	-0.97779300	-3.50325200
C	0.13498000	-1.05116100	-0.73477200
H	-1.77280100	-2.84363500	0.00745900
H	-3.12622800	-4.23217500	-1.50429000
C	1.06647800	-0.18194800	-1.26901100
C	1.28747500	-0.16566400	-2.67027400
H	2.01637200	0.51988300	-3.09135200
C	-0.13498000	-1.05124000	0.73476000
C	0.62698000	-1.91097600	1.58082000
C	-1.06651800	-0.18211000	1.26905500
C	1.61189700	-2.80063000	1.06542500
C	0.42245700	-1.85454900	2.98727800
C	-1.28757000	-0.16597700	2.67031100
C	2.36712000	-3.57056600	1.90948500
H	1.77287200	-2.84360600	-0.00759100
C	1.22007200	-2.66894800	3.83535700
C	-0.56333200	-0.97816400	3.50323000
H	-2.01650500	0.51949900	3.09143600
C	2.17329200	-3.50164200	3.31146200
H	3.12631200	-4.23222500	1.50406700
H	1.06020800	-2.61347200	4.90919600
H	-0.72501900	-0.94542100	4.57767200
H	2.78273700	-4.11534800	3.96812000
P	-1.97286600	0.98025600	0.16807700
P	1.97289100	0.98030500	-0.16796700
C	3.37052200	-0.02512800	0.42331700
C	4.16479400	-0.79382500	-0.43285900
C	3.56127100	-0.09495200	1.80406000
C	5.15856900	-1.61390500	0.09169100
H	3.99765300	-0.76035300	-1.50799100
C	4.55109400	-0.92342000	2.32657300
H	2.90728600	0.48493600	2.44977400
C	5.35047800	-1.67774500	1.47170300
H	5.77782600	-2.20958500	-0.57221600
H	4.68970600	-0.98926900	3.40146600
H	6.11999700	-2.32668900	1.87982900
C	2.60567400	2.24156000	-1.30660400
C	3.96374500	2.46891200	-1.52804400
C	1.64223800	3.04306700	-1.93531400
C	4.36369300	3.49183800	-2.38643500
H	4.70874500	1.85389400	-1.03049800
C	2.05021200	4.06042700	-2.79011500
H	0.58532200	2.85149900	-1.74924000
C	3.40877900	4.28350000	-3.01689200
H	5.42059100	3.67081400	-2.55908700
H	1.30772300	4.68241700	-3.28068600
H	3.72268400	5.08002200	-3.68521400
C	-3.37045300	-0.02512500	-0.42340200
C	-4.16478600	-0.79390100	0.43264600
C	-3.56110300	-0.09482300	-1.80416500
C	-5.15852000	-1.61393500	-0.09205200
H	-3.99772000	-0.76052700	1.50779300
C	-4.55088800	-0.92324300	-2.32682600
H	-2.90707400	0.48512800	-2.44977900
C	-5.35033200	-1.67764700	-1.47208300
H	-5.77782300	-2.20967800	0.57175600
H	-4.68942200	-0.98899300	-3.40173500
H	-6.11981900	-2.32655400	-1.88032400
C	-2.60572500	2.24140100	1.30679400
C	-1.64232800	3.04284600	1.93564400
C	-3.96380900	2.46872800	1.52817700
C	-2.05035400	4.06011200	2.79053200
H	-0.58540100	2.85129700	1.74961700
C	-4.36380900	3.49155800	2.38665900
H	-4.70877900	1.85376000	1.03052300
C	-3.40893500	4.28315500	3.01725700
H	-1.30789500	4.68205100	3.28121200
H	-5.42071800	3.67051100	2.55927100
H	-3.72288000	5.07960300	3.68564700
O	-1.21144000	1.56659300	-0.98216400
O	1.21154400	1.56653400	0.98238100

3b

C	0.24260000	2.97560000	-2.14130000
C	-0.24660000	1.67610000	-2.18060000
C	1.29520000	3.28650000	-1.27600000
C	0.29970000	0.67960000	-1.35350000
C	1.32870000	1.01430000	-0.48170000
C	1.84720000	2.31470000	-0.44600000
H	2.63690000	2.56450000	0.25040000
C	-0.29960000	-0.67980000	-1.35340000
C	0.24670000	-1.67650000	-2.18030000
C	-1.32860000	-1.01440000	-0.48160000
C	-0.24250000	-2.97590000	-2.14090000
C	-1.84710000	-2.31480000	-0.44570000
C	-1.29510000	-3.28670000	-1.27550000
H	-2.63690000	-2.56440000	0.25070000
P	-1.96800000	0.25990000	0.67630000
P	1.96810000	-0.25980000	0.67630000
C	3.21490000	-1.15900000	-0.29640000
C	4.04620000	-0.54120000	-1.23570000
C	3.32190000	-2.53390000	-0.08440000
C	4.98750000	-1.29030000	-1.93140000
H	3.94790000	0.52470000	-1.42840000
C	4.26800000	-3.27750000	-0.78710000
H	2.64910000	-3.00660000	0.62580000
C	5.11240000	-2.66810000	-1.71700000
H	5.63340000	-0.80400000	-2.65900000
H	4.34910000	-4.34800000	-0.61450000
C	2.84330000	0.72250000	1.92420000
C	4.22520000	0.71600000	2.09610000
C	2.04000000	1.51250000	2.76110000
C	4.80580000	1.49510000	3.09670000
H	4.85330000	0.10610000	1.45210000
C	2.63170000	2.28450000	3.75060000
H	0.96030000	1.52030000	2.61070000
C	4.02090000	2.28570000	3.93480000
H	5.88510000	1.48630000	3.22760000
H	2.00940000	2.90000000	4.39610000
C	-3.21480000	1.15900000	-0.29660000
C	-4.04620000	0.54110000	-1.23580000
C	-3.32180000	2.53400000	-0.08490000

C	-4.98750000	1.29010000	-1.93160000
H	-3.94800000	-0.52490000	-1.42830000
C	-4.26790000	3.27750000	-0.78770000
H	-2.64880000	3.00670000	0.62520000
C	-5.11230000	2.66790000	-1.71740000
H	-5.63340000	0.80370000	-2.65910000
H	-4.34880000	4.34800000	-0.61530000
C	-2.84330000	-0.72230000	1.92430000
C	-2.04000000	-1.51220000	2.76120000
C	-4.22520000	-0.71560000	2.09610000
C	-2.63180000	-2.28400000	3.75080000
H	-0.96040000	-1.52000000	2.61080000
C	-4.80590000	-1.49460000	3.09680000
H	-4.85330000	-0.10580000	1.45200000
C	-4.02110000	-2.28510000	3.93490000
H	-2.00960000	-2.89950000	4.39640000
H	-5.88520000	-1.48570000	3.22760000
O	-0.98420000	1.20940000	1.29170000
O	0.98430000	-1.20930000	1.29180000
O	-1.27050000	1.27730000	-2.97590000
C	-2.01130000	2.28140000	-3.63330000
H	-1.40500000	2.79520000	-4.38970000
H	-2.84390000	1.77160000	-4.11900000
O	1.27060000	-1.27780000	-2.97570000
C	2.01140000	-2.28200000	-3.63290000
H	1.40510000	-2.79590000	-4.38930000
H	2.84400000	-1.77230000	-4.11870000
H	2.40210000	-3.01170000	-2.91300000
H	-2.40210000	3.01120000	-2.91350000
H	0.16450000	-3.77070000	-2.75360000
H	-0.16450000	3.77030000	-2.75410000
O	-1.70610000	-4.58290000	-1.30500000
O	1.70610000	4.58270000	-1.30570000
C	-2.71420000	-4.96070000	-0.39310000
H	-2.40480000	-4.77480000	0.64250000
H	-3.65050000	-4.42360000	-0.58810000
H	-2.87190000	-6.02890000	-0.54240000
C	2.71410000	4.96070000	-0.39380000
H	2.40480000	4.77500000	0.64180000
H	3.65050000	4.42360000	-0.58870000
H	2.87180000	6.02890000	-0.54320000
C	-4.64210000	-3.11290000	5.03100000
H	-4.43150000	-2.67790000	6.01400000
H	-5.72760000	-3.17380000	4.91800000
H	-4.24050000	-4.13090000	5.03330000
C	-6.10920000	3.47580000	-2.50830000
H	-7.05270000	2.93550000	-2.62800000
H	-6.32080000	4.43190000	-2.02270000
H	-5.72600000	3.69090000	-3.51260000
C	4.64190000	3.11360000	5.03070000
H	4.43130000	2.67860000	6.01380000
H	5.72730000	3.17460000	4.91780000
H	4.24020000	4.13160000	5.03300000
C	6.10900000	-3.47630000	-2.50790000
H	7.04990000	-2.93310000	-2.63440000
H	6.32610000	-4.42910000	-2.01840000
H	5.72230000	-3.69800000	-3.50940000

3c

C	2.17657800	-3.53352700	3.31710200
C	0.92845900	-2.88323000	3.90715200
C	0.25079100	-1.92112500	2.95160100
C	0.51445800	-1.93600900	1.57283000
C	1.52552200	-2.90614000	0.98564200
C	1.86108900	-4.06482400	1.92253600
H	-0.87037800	-0.99620700	4.52898500
H	2.98235300	-2.79187300	3.24027800
H	0.20998000	-3.67027000	4.17803700
C	-0.67195500	-1.00703100	3.45941200
C	-0.14930900	-1.02146000	0.73463800
H	1.16019200	-3.28819000	0.02614300
H	2.70662600	-4.62958300	1.51626800
C	-1.06316800	-0.09700800	1.26080300
C	-1.32184900	-0.10280400	2.63287300
H	-2.01136700	0.61628400	3.06472000
C	0.14933000	-1.02134700	-0.73462500
C	-0.51440800	-1.93583600	-1.57290200
C	1.06311800	-0.09676700	-1.26071900
C	-1.52534400	-2.90614400	-0.98578200
C	-0.25081100	-1.92076300	-2.95168500
C	1.32170200	-0.10235900	-2.63280500
C	-1.86076500	-4.06478300	-1.92278300
H	-1.15996500	-3.28821800	-0.02631300
C	-0.92842800	-2.88282100	-3.90732200
C	0.67182600	-1.00652200	-3.45942500
H	2.01115500	0.61682700	-3.06459800
C	-2.17641600	-3.53336500	-3.31726800
H	-1.00882300	-4.75634200	-1.98270000
H	-1.16647200	-2.35431900	-4.83765100
H	0.87020000	-0.99555100	-4.52900600
H	-2.52887600	-4.33291800	-3.97737000
P	1.92672900	1.10470700	-0.17581200
P	-1.92673300	1.10456800	0.17598000
C	-3.37583800	0.15883700	-0.39794700
C	-4.20323800	-0.56503800	0.46681400
C	-3.58271000	0.09557800	-1.77630300
C	-5.23880600	-1.33912700	-0.04623600
H	-4.02585400	-0.53625400	1.53997200
C	-4.61943400	-0.68328000	-2.28786700
H	-2.91008000	0.64701500	-2.42796600
C	-5.44411700	-1.40055300	-1.42483400
H	-5.87951700	-1.90302400	0.62487400
H	-4.78023100	-0.73445000	-3.36090900
H	-6.24781000	-2.01180700	-1.82478900
C	-2.52091700	2.38770100	1.31496100
C	-3.87331600	2.67827900	1.49735700
C	-1.54265000	3.14697100	1.97275000
C	-4.25274100	3.71710200	2.34569000
H	-4.63206300	2.10038600	0.97713700
C	-1.93028100	4.18098800	2.81729800
H	-0.49055100	2.91237200	1.81439800
C	-3.28290900	4.46499100	3.00595400
H	-5.30553100	3.94192000	2.48732700
H	-1.17465600	4.76876500	3.32959300
H	-3.58022700	5.27441900	3.66632500
C	3.37592100	0.15895000	0.39786900
C	4.20323300	-0.56482700	-0.46706200
C	3.58296300	0.09556200	1.77619200
C	5.23887000	-1.33895300	0.04578900
H	4.02573500	-0.53592300	-1.54019700
C	4.61974900	-0.68334400	2.28755800
H	2.91039800	0.64691500	2.42799300
C	5.44433900	-1.40051900	1.42435800
H	5.87950600	-1.90278200	-0.62545100
H	4.78067300	-0.73462300	3.36057500
H	6.24807100	-2.01182300	1.82415800

C	2.52075900	2.38797300	-1.31471800
C	1.54241200	3.14728400	-1.97234300
C	3.87313600	2.67859800	-1.49721300
C	1.92994200	4.18138600	-2.81683100
H	0.49033500	2.91265200	-1.81390200
C	4.25245900	3.71751000	-2.34548100
H	4.63193900	2.10066900	-0.97711300
C	3.28254800	4.46543500	-3.00558500
H	1.17426000	4.76919600	-3.32900200
H	5.30523000	3.94237100	-2.48719600
H	3.57978700	5.27492900	-3.66591100
O	1.17550800	1.66224300	0.99588100
O	-1.17541400	1.66224400	-0.99558400
H	-0.20986500	-3.66972800	-4.17836800
H	-2.98228600	-2.79182800	-3.24029700
H	-2.70618500	-4.62973400	-1.51653900
H	-2.44932600	-2.35590000	-0.75586400
H	1.16636400	-2.35482800	4.83757200
H	2.52910200	-4.33311500	3.97712800
H	1.00926500	-4.75653900	1.98232400
H	2.44943100	-2.35576300	0.75576600

3d

C	-0.19343900	-2.13770500	-2.96667800
C	-0.51067300	-2.13853200	-1.60783300
H	1.07470700	-1.31450900	-4.48581800
C	0.82905500	-1.31431800	-3.42813300
C	0.19449300	-1.31736600	-0.71845800
C	1.19195300	-0.47011300	-1.19968100
C	1.51635700	-0.47517800	-2.55977400
H	2.27704300	0.19513600	-2.94656700
C	-0.19442900	-1.31750400	0.71841100
C	0.51083300	-2.13871400	1.60767800
C	-1.19199100	-0.47044700	1.19973100
C	0.19356600	-2.13811000	2.96651700
C	-1.51645700	-0.47576000	2.55980900
C	-0.82907000	-1.31493700	3.42806000
H	-2.27719700	0.19444400	2.94668600
H	-1.07473000	-1.31529500	4.48574300
P	-1.99812400	0.70325000	0.04350600
P	1.99818900	0.70338800	-0.04332100
C	3.38573500	-0.23383900	0.67703200
C	4.13443500	-1.16811700	-0.04449900
C	3.69422400	0.02190800	2.01475500
C	5.20510100	-1.81863400	0.56229400
H	3.87288200	-1.39293700	-1.07607500
C	4.76518700	-0.63309900	2.62062200
H	3.07984800	0.72889000	2.56566000
C	5.52314800	-1.54770700	1.89328800
H	5.78886900	-2.54102100	-0.00069500
H	5.00707800	-0.42913800	3.65950100
H	6.35972500	-2.05556600	2.36433000
C	2.70303400	1.95720300	-1.14981500
C	4.07094300	2.13666800	-1.35219800
C	1.77669900	2.79034600	-1.79223900
C	4.51848900	3.14754500	-2.20158100
H	4.78664200	1.49206500	-0.84867800
C	2.23184300	3.79563000	-2.63749600
H	0.71132000	2.62931700	-1.62705700
C	3.60046400	3.97364600	-2.84260300
H	5.58331300	3.28971800	-2.35969800
H	1.51854400	4.44263800	-3.13905300
H	3.95104300	4.76023000	-3.50450500
C	-3.38552000	-0.23388600	-0.67726200
C	-4.13415500	-1.16852500	0.04386800
C	-3.69401500	0.02239100	-2.01488500
C	-5.20476900	-1.81886600	-0.56321000
H	-3.87259900	-1.39375300	1.07535400
C	-4.76492300	-0.63243500	-2.62103500
H	-3.07969300	0.72965300	-2.56548900
C	-5.52282400	-1.54740600	-1.89409200
H	-5.78848800	-2.54153400	-0.00053100
H	-5.00681600	-0.42805900	-3.65983400
H	-6.35936500	-2.05512100	-2.36535300
C	-2.70328300	1.95680300	1.15010900
C	-1.77717800	2.79015500	1.79258800
C	-4.07123800	2.13573000	1.35269300
C	-2.23259300	3.79512400	2.63807700
H	-0.71176800	2.62946800	1.62732500
C	-4.51905400	3.14629300	2.20230400
H	-4.78676300	1.49095200	0.84915100
C	-3.60125300	3.97261200	2.84337000
H	-1.51947000	4.44229400	3.13967700
H	-5.58391000	3.28806000	2.36056500
H	-3.95204000	4.75895500	3.50544700
O	-1.14858900	1.30900900	-1.03261500
O	1.14883400	1.30891200	1.03307000
O	-1.49678200	-2.88958700	-1.05284900
C	-2.39445600	-3.53564900	-1.92834700
H	-1.89779600	-4.33486300	-2.49297600
H	-3.17203000	-3.96622800	-1.29716700
O	1.49712700	-2.88947200	1.05261700
C	2.39486600	-3.53554300	1.92804100
H	1.89829000	-4.33489300	2.49254900
H	3.17251200	-3.96593200	1.29682400
H	2.84898000	-2.81812600	2.62273600
H	-2.84867400	-2.81819400	-2.62292600
H	0.73603000	-2.76776100	3.66228300
H	-0.73583100	-2.76734600	-3.66251000

3f

C	0.80754100	-2.76739300	-2.71955000
C	0.21251300	-2.67824300	-1.46793100
H	2.07866800	-1.76740900	-4.13417300
C	1.61677200	-1.72545400	-3.15271400
C	0.37591000	-1.56003600	-0.64353000
C	1.26253400	-0.55085100	-1.05574500
C	1.86255100	-0.63976000	-2.31477700
H	2.52922100	0.14921800	-2.64928300
C	-0.37567500	-1.55987600	0.64369600
C	-0.21207500	-2.67781300	1.46842600
C	-1.26268400	-0.55085600	1.05545000
C	-0.80741700	-2.76698100	2.71987000
C	-1.86310400	-0.63983200	2.31430800
C	-1.61722900	-1.72527400	3.15252400
H	-2.53015300	0.14893800	2.64853600
H	-2.07948600	-1.76721700	4.13381300
P	-1.87673900	0.70837700	-0.12879200
P	1.87651600	0.70853700	0.12831700
C	3.26515000	-0.17076700	0.92095500
C	4.26840100	-0.83554600	0.20821700
C	3.28114700	-0.18129300	2.31621200
C	5.28819900	-1.49037200	0.88973500
H	4.25042100	-0.84577600	-0.87925500

C	4.30058400	-0.84471200	2.99641200
H	2.48224600	0.32765400	2.84746700
C	5.30385700	-1.49468000	2.28443500
H	6.06788300	-2.00395900	0.33559400
H	4.30940500	-0.85457200	4.08205700
H	6.09876300	-2.01129800	2.81410100
C	2.59917200	2.01988400	-0.90244800
C	3.94369600	2.38584200	-0.80361200
C	1.74202600	2.72507400	-1.75856000
C	4.43464000	3.44498300	-1.56421300
H	4.60881000	1.84959500	-0.13292000
C	2.24137100	3.78035000	-2.51418600
H	0.69424500	2.43803400	-1.81636500
C	3.58517700	4.13965200	-2.41957800
H	5.48017900	3.72699900	-1.48525900
H	1.57835800	4.32765200	-3.17739900
H	3.96920000	4.96504500	-3.01210900
C	-3.26590900	-0.17099600	-0.92040900
C	-4.27100600	-0.83205500	-0.20680300
C	-3.28013300	-0.18599400	-2.31563000
C	-5.29080000	-1.48772800	-0.88748500
H	-4.25455300	-0.83858500	0.88072800
C	-4.29960200	-0.85026300	-2.99498200
H	-2.48001400	0.32041300	-2.84748400
C	-5.30465800	-1.49655700	-2.28219600
H	-6.07191600	-1.99842800	-0.33269100
H	-4.30708400	-0.86355400	-4.08060100
H	-6.09959400	-2.01379200	-2.81121400
C	-2.59873200	2.02031400	0.90169700
C	-1.74221000	2.72347200	1.76008100
C	-3.94230500	2.38900000	0.79999000
C	-2.24124100	3.77925800	2.51520200
H	-0.69502400	2.43469200	1.81983100
C	-4.43294300	3.44865100	1.56007300
H	-4.60698400	1.85447200	0.12749600
C	-3.58412800	4.14118400	2.41780500
H	-1.57862800	4.32488900	3.18019400
H	-5.47774800	3.73273300	1.47885500
H	-3.96791800	4.96696000	3.00995300
O	-0.93238500	1.20233000	-1.18376000
O	0.93184800	1.20340700	1.18254500
O	-0.58342800	-3.71207900	-1.02176400
O	0.58439900	-3.71142000	1.02259500
C	0.00059900	-4.48407000	0.00063600
H	0.81846500	-5.10088800	-0.38670100
H	-0.81713600	-5.10088900	0.38824600
H	-0.62267500	-3.64674300	3.32765800
H	0.62296700	-3.64736500	-3.32708900

3g

C	0.54127900	-2.44797000	-2.85288900
C	0.02165600	-2.42710000	-1.56058100
H	1.80134900	-1.44105700	-4.27076200
C	1.39862200	-1.43840900	-3.26261200
C	0.31853500	-1.38917100	-0.67343000
C	1.22072700	-0.39103600	-1.08418700
C	1.75055900	-0.42371700	-2.37507700
H	2.43641300	0.35264200	-2.69886800
C	-0.31859200	-1.38910400	0.67369600
C	-0.02179700	-2.42698900	1.56092100
C	-1.22079100	-0.39092400	1.08432700
C	-0.54150000	-2.44777500	2.85319800
C	-1.75072900	-0.42354000	2.37517300
C	-1.39886400	-1.43818200	3.26279400
H	-2.43659000	0.35284500	2.69888500
H	-1.80166600	-1.44076200	4.27091500
P	-1.88833100	0.82674900	-0.11718700
P	1.88836100	0.82670900	0.11719100
C	3.24494600	-0.10608100	0.89968300
C	4.17130600	-0.86617400	0.17896700
C	3.31854900	-0.05711000	2.29247100
C	5.17557400	-1.55632800	0.84896600
H	4.10208300	-0.92489900	-0.90504300
C	4.32188300	-0.75525000	2.96108300
H	2.57365000	0.52109100	2.83175500
C	5.25076300	-1.49983600	2.24040500
H	5.89525400	-2.14478300	0.28801900
H	4.37629000	-0.71920800	4.04490300
H	6.03330900	-2.04308700	2.76185300
C	2.64358500	2.12128200	-0.90991500
C	4.01293200	2.39282200	-0.89742300
C	1.77683900	2.90901800	-1.68044000
C	4.51985800	3.44370100	-1.65954500
H	4.68373700	1.79031200	-0.29165300
C	2.29188400	3.95514700	-2.43784100
H	0.71138100	2.68542300	-1.67767900
C	3.66074000	4.22224300	-2.42893500
H	5.58490200	3.65454700	-1.64817900
H	1.62372400	4.56591400	-3.03734900
H	4.05734400	5.04109400	-3.02228600
C	-3.24479100	-0.10618400	-0.89972500
C	-4.17168200	-0.86560400	-0.17898000
C	-3.31765400	-0.05812600	-2.29257900
C	-5.17574500	-1.55599100	-0.84904000
H	-4.10304700	-0.92360900	0.90510600
C	-4.32077700	-0.75651500	-2.96125100
H	-2.57239900	0.51963700	-2.83183600
C	-5.25018500	-1.50042200	-2.24055900
H	-5.89586100	-2.14390500	-0.28808600
H	-4.37461900	-0.72117600	-4.04512100
H	-6.03257700	-2.04385500	-2.76205100
C	-2.64367500	2.12137400	0.90976900
C	-1.77709600	2.90883200	1.68077100
C	-4.01294400	2.39328000	0.89664100
C	-2.29223400	3.95502100	2.43802400
H	-0.71168900	2.68499000	1.67847200
C	-4.51996600	3.44422300	1.65861100
H	-4.68361600	1.79099700	0.29050100
C	-3.66101900	4.22246700	2.42849100
H	-1.62420700	4.56556700	3.03790300
H	-5.58495000	3.65534500	1.64675500
H	-4.05769700	5.04135800	3.02173700
O	-0.96681100	1.37042500	-1.16776400
O	0.96691100	1.37048000	1.16777800
O	-0.80473200	-3.45392800	-1.16276100
C	-0.10745000	-4.62555500	-0.75114800
H	0.85673900	-4.68543800	-1.27031500
O	0.80457600	-3.45386800	1.16319500
C	0.10727400	-4.62551000	0.75166400
H	-0.85691300	-4.68534200	1.27084000
H	0.71793100	-5.48728600	1.04036700
H	-0.71812000	-5.48733800	-1.03980000
H	-0.25744200	-3.26069100	3.51466400
H	0.25715900	-3.26092000	-3.51428600

3h

C	0.45735400	-2.39456500	-2.73619000
C	-0.06190500	-2.29191900	-1.44741700
H	1.77071100	-1.51361800	-4.18619800
C	1.36925400	-1.44550600	-3.17972000
C	0.30763900	-1.23921800	-0.60306400
C	1.23997400	-0.29623500	-1.05430000
C	1.76533300	-0.40455100	-2.34420500
H	2.47604900	0.33184100	-2.70629900
C	-0.31154700	-1.19760800	0.75259500
C	0.04633600	-2.19514200	1.66399400
C	-1.26605800	-0.24260300	1.12863200
C	-0.50323000	-2.23220800	2.94318600
C	-1.81871300	-0.28369800	2.41091300
C	-1.43444600	-1.27178000	3.31405800
H	-2.54582900	0.46340000	2.71416300
H	-1.85932700	-1.28667900	4.31310900
P	-1.93870200	0.92633200	-0.11365200
P	1.90416800	0.97078700	0.09395900
C	3.28248500	0.09154400	0.90032800
C	4.21030400	-0.68625400	0.20091100
C	3.37137700	0.19907600	2.28887400
C	5.23127600	-1.33525400	0.88666300
H	4.12899100	-0.79143500	-0.87870500
C	4.39130100	-0.45825000	2.97377300
H	2.62544400	0.78952700	2.81333200
C	5.32161700	-1.22038200	2.27355400
H	5.95234700	-1.93699600	0.34175200
H	4.45749700	-0.37667900	4.05453600
H	6.11681200	-1.73240600	2.80739900
C	2.62801000	2.23096000	-0.99576900
C	3.99122900	2.53132800	-1.00729200
C	1.73905600	2.95974500	-1.79777500
C	4.47010500	3.55350800	-1.82481100
H	4.67858500	1.97279500	-0.37801500
C	2.22609000	3.97772700	-2.61014600
H	0.67827900	2.71519400	-1.77393100
C	3.58897800	4.27376100	-2.62536900
H	5.53040900	3.78740300	-1.83244700
H	1.54070600	4.54436900	-3.23312800
H	3.96354900	5.07002500	-3.26212300
C	-3.27114000	-0.05378700	-0.87891800
C	-4.25012400	-0.72930100	-0.14369600
C	-3.25270500	-0.15421400	-2.27059100
C	-5.21732800	-1.48124800	-0.80101600
H	-4.25172100	-0.67258000	0.94269800
C	-4.21790200	-0.91588600	-2.92578200
H	-2.46868400	0.36252800	-2.81685900
C	-5.20090500	-1.57366700	-2.19269500
H	-5.98052800	-2.00165300	-0.23039400
H	-4.20157700	-0.99539200	-4.00858000
H	-5.95539300	-2.16447400	-2.70390100
C	-2.70697400	2.24058700	0.87497700
C	-1.84645400	3.01879900	1.66209800
C	-4.07000000	2.53589400	0.82239500
C	-2.36255800	4.08013400	2.39716800
H	-0.78406700	2.77954900	1.68512000
C	-4.57830800	3.60134400	1.56310400
H	-4.73421000	1.94012000	0.20270700
C	-3.72603400	4.37024300	2.34978900
H	-1.69999800	4.68494000	3.00876400
H	-5.63875900	3.83097300	1.52220400
H	-4.12392000	5.20045400	2.92631000
O	-1.01078600	1.44284400	-1.17211300
O	0.97709200	1.53782700	1.12639400
O	-0.98056000	-3.18260300	-0.95685400
C	-0.65698400	-4.56155300	-1.06079200
O	0.98100400	-3.11630900	1.27547800
C	0.56566300	-4.47754300	1.18593000
H	-0.43447100	-4.59655300	1.62277300
H	1.27182500	-5.06848200	1.77838700
H	-0.54831500	-4.86234000	-2.11108400
H	-0.18239500	-3.00644900	3.63415700
H	0.13718900	-3.20163500	-3.38754700
H	-1.53570300	-5.07478700	-0.65867200
C	0.58850700	-4.94496500	-0.26813300
H	0.68287800	-6.03754900	-0.30003600
H	1.48622200	-4.52813800	-0.73892700

3i

C	-0.46429300	-2.95433000	-2.03228000
C	0.12271900	-1.69633400	-2.07846900
C	-1.54379900	-3.17665200	-1.17318900
C	-0.35293900	-0.65394800	-1.26493300
C	-1.40943700	-0.90243200	-0.39694800
C	-2.02625000	-2.15849100	-0.35523400
H	-2.83601800	-2.34294200	0.33859900
C	0.35301600	0.65402200	-1.26491100
C	-0.12260700	1.69643300	-2.07843000
C	1.40947900	0.90246700	-0.39688000
C	0.46440200	2.95442900	-2.03216500
C	2.02628600	2.15852600	-0.35507700
C	1.54386500	3.17671900	-1.17301100
H	2.83602200	2.34295100	0.33879800
P	1.93313300	-0.41673200	0.76766500
P	-1.93315900	0.41667200	0.76767800
C	-3.11526700	1.42639700	-0.18137000
C	-4.00348800	0.88771100	-1.11708800
C	-3.11647900	2.79997900	0.07028600
C	-4.90846100	1.71707700	-1.77300600
H	-3.97887800	-0.17748700	-1.33605400
C	-4.02246600	3.62833400	-0.58958500
H	-2.39643200	3.20017000	0.77866900
C	-4.92168500	3.08605000	-1.50501500
H	-5.60132800	1.29812300	-2.49672600
H	-4.02584300	4.69572100	-0.38982000
H	-5.63002800	3.73071100	-2.01735500
C	-2.87989900	-0.49224100	2.02319500
C	-4.25725000	-0.36654300	2.19896400
C	-2.13628700	-1.34458600	2.85224800
C	-4.89801500	-1.09484500	3.20082300
H	-4.83154600	0.29690900	1.55772100
C	-2.78364400	-2.06979900	3.84609800
H	-1.06152500	-1.43634300	2.69426000
C	-4.16280300	-1.94551900	4.02047900
H	-5.97026500	-0.99497600	3.34042800
H	-2.21151900	-2.73233500	4.48862100
H	-4.66391300	-2.51163600	4.80033000
C	3.11537200	-1.42630100	-0.18138700
C	4.00369100	-0.88746400	-1.11692600
C	3.11657400	-2.79992100	0.07006400
C	4.90875100	-1.71672000	-1.77286200
H	3.97909100	0.17776600	-1.33573500
C	4.02263900	-3.62816700	-0.58983400

H	2.39644700	-3.20022900	0.77830000
C	4.92195600	-3.08573400	-1.50508000
H	5.60169700	-1.29764800	-2.49643800
H	4.02600200	-4.69558400	-0.39023300
H	5.63036300	-3.73031000	-2.01743900
C	2.87970800	0.49205900	2.02339700
C	2.13597400	1.34425900	2.85248900
C	4.25704700	0.36639900	2.19928900
C	2.78319800	2.06937300	3.84649700
H	1.06122500	1.43599000	2.69440300
C	4.89767800	1.09460100	3.20130600
H	4.83143700	-0.29694300	1.55801600
C	4.16234600	1.94513500	4.02099900
H	2.21097900	2.73179900	4.48905000
H	5.96991900	0.99476400	3.34100500
H	4.66335100	2.51117300	4.80097400
O	0.86813200	-1.27830300	1.37695300
O	-0.86819700	1.27813400	1.37719100
O	1.18357300	-1.38476300	-2.86395400
C	1.86071600	-2.44969100	-3.49505200
H	1.22485600	-2.94126600	-4.24192600
H	2.72427200	-2.00503000	-3.99014800
O	-1.18341800	1.38488200	-2.86398200
C	-1.86052500	2.44982000	-3.49510200
H	-1.22462900	2.94139500	-4.24194700
H	-2.72406300	2.00516800	-3.99023900
H	-2.20308200	3.18574400	-2.75686700
H	2.20324800	-3.18561900	-2.75680700
H	0.11303400	3.78445300	-2.63261300
H	-0.11289400	-3.78433100	-2.63274200
O	2.05061100	4.43830800	-1.19353800
O	-2.05054500	-4.43824000	-1.19379400
C	3.07262000	4.73978200	-0.26834100
H	2.74259400	4.56091500	0.76220900
H	3.97508400	4.14741700	-0.46338500
H	3.29887800	5.79770700	-0.40285800
C	-3.07259200	-4.73976100	-0.26865400
H	-2.74260900	-4.56094200	0.76191800
H	-3.97505000	-4.14739000	-0.46370900
H	-3.29884000	-5.79768000	-0.40323000

3j

C	0.24260000	2.97560000	-2.14130000
C	-0.24660000	1.67610000	-2.18060000
C	1.29520000	3.28650000	-1.27600000
C	0.29970000	0.67960000	-1.35350000
C	1.32870000	1.01430000	-0.48170000
C	1.84720000	2.31470000	-0.44600000
H	2.63690000	2.56450000	0.25040000
C	-0.29960000	-0.67980000	-1.35340000
C	0.24670000	-1.67650000	-2.18030000
C	-1.32860000	-1.01440000	-0.48160000
C	-0.24250000	-2.97590000	-2.14090000
C	-1.84710000	-2.31480000	-0.44570000
C	-1.29510000	-3.28670000	-1.27550000
H	-2.63690000	-2.56440000	0.25070000
P	-1.96800000	0.25990000	0.67630000
P	1.96810000	-0.25980000	0.67630000
C	3.21490000	-1.15900000	-0.29640000
C	4.04620000	-0.54120000	-1.23570000
C	3.32190000	-2.53390000	-0.08440000
C	4.98750000	-1.29030000	-1.93140000
H	3.94790000	0.52470000	-1.42840000
C	4.26800000	-3.27750000	-0.78710000
H	2.64910000	-3.00660000	0.62580000
C	5.11240000	-2.66810000	-1.71700000
H	5.63340000	-0.80400000	-2.65900000
H	4.34910000	-4.34800000	-0.61450000
C	2.84330000	0.72250000	1.92420000
C	4.22520000	0.71600000	2.09610000
C	2.04000000	1.51250000	2.76110000
C	4.80580000	1.49510000	3.09670000
H	4.85330000	0.10610000	1.45210000
C	2.63170000	2.28450000	3.75060000
H	0.96030000	1.52030000	2.61070000
C	4.02090000	2.28570000	3.93480000
H	5.88510000	1.48630000	3.22760000
H	2.00940000	2.90000000	4.39610000
C	-3.21480000	1.15900000	-0.29660000
C	-4.04620000	0.54110000	-1.23580000
C	-3.32180000	2.53400000	-0.08490000
C	-4.98750000	1.29010000	-1.93160000
H	-3.94800000	-0.52490000	-1.42830000
C	-4.26790000	3.27750000	-0.78770000
H	-2.64880000	3.00670000	0.62520000
C	-5.11230000	2.66790000	-1.71740000
H	-5.63340000	0.80370000	-2.65910000
H	-4.34880000	4.34800000	-0.61530000
C	-2.84330000	-0.72230000	1.92430000
C	-2.04000000	-1.51220000	2.76120000
C	-4.22520000	-0.71560000	2.09610000
C	-2.63180000	-2.28400000	3.75080000
H	-0.96040000	-1.52000000	2.61080000
C	-4.80590000	-1.49460000	3.09680000
H	-4.85330000	-0.10580000	1.45200000
C	-4.02110000	-2.28510000	3.93490000
H	-2.00960000	-2.89950000	4.39640000
H	-5.88520000	-1.48570000	3.22760000
O	-0.98420000	1.20940000	1.29170000
O	0.98430000	-1.20930000	1.29180000
O	-1.27050000	1.27730000	-2.97590000
C	-2.01130000	2.28140000	-3.63330000
H	-1.40500000	2.79520000	-4.38970000
H	-2.84390000	1.77160000	-4.11900000
O	1.27060000	-1.27780000	-2.97570000
C	2.01140000	-2.28200000	-3.63290000
H	1.40510000	-2.79590000	-4.38930000
H	2.84400000	-1.77230000	-4.11870000
H	2.40210000	-3.01170000	-2.91300000
H	-2.40210000	3.01120000	-2.91350000
H	0.16450000	-3.77070000	-2.75360000
H	-0.16450000	3.77030000	-2.75410000
O	-1.70610000	-4.58290000	-1.30500000
O	1.70610000	4.58270000	-1.30570000
C	-2.71420000	-4.96070000	-0.39310000
H	-2.40480000	-4.77480000	0.64250000
H	-3.65050000	-4.42360000	-0.58810000
H	-2.87190000	-6.02890000	-0.54240000
C	2.71410000	4.96070000	-0.39380000
H	2.40480000	4.77500000	0.64180000
H	3.65050000	4.42360000	-0.58870000
H	2.87180000	6.02890000	-0.54320000
C	-4.64210000	-3.11290000	5.03100000
H	-4.43150000	-2.67790000	6.01400000
H	-5.72760000	-3.17380000	4.91800000
H	-4.24050000	-4.13090000	5.03330000

C	-6.10920000	3.47580000	-2.50830000
H	-7.05270000	2.93550000	-2.62800000
H	-6.32080000	4.43190000	-2.02270000
H	-5.72600000	3.69090000	-3.51260000
C	4.64190000	3.11360000	5.03070000
H	4.43130000	2.67860000	6.01380000
H	5.72730000	3.17460000	4.91780000
H	4.24020000	4.13160000	5.03300000
C	6.10900000	-3.47630000	-2.50790000
H	7.04990000	-2.93310000	-2.63440000
H	6.32610000	-4.42910000	-2.01840000
H	5.72230000	-3.69800000	-3.50940000

3k

C	-0.18050200	-2.92801700	-2.27756800
C	0.27791300	-1.61666200	-2.28802900
C	-1.22265100	-3.28375000	-1.41730200
C	-0.28867600	-0.65326500	-1.43654500
C	-1.30847500	-1.03205200	-0.57133200
C	-1.79610600	-2.34432900	-0.56469400
H	-2.57898900	-2.62713800	0.12722300
C	0.28383500	0.71812800	-1.40155400
C	-0.28097300	1.72413800	-2.20382200
C	1.30531900	1.05065500	-0.51983400
C	0.18310400	3.03153200	-2.13101400
C	1.79610900	2.35998800	-0.44784400
C	1.22654300	3.34132600	-1.25436900
H	2.57984200	2.60560400	0.25731900
P	1.97141400	-0.24099300	0.60303000
P	-1.97557500	0.20179700	0.61340300
C	-3.22227600	1.11302600	-0.35324400
C	-4.03692200	0.49712600	-1.30564100
C	-3.33434800	2.48151100	-0.11529900
C	-4.99119700	1.23709400	-2.00280600
H	-3.91732300	-0.56467200	-1.51604100
C	-4.27789200	3.24564600	-0.80853900
H	-2.66311500	2.94284700	0.60601600
C	-5.09894800	2.60578100	-1.73686400
H	-5.83751700	3.19149900	-2.28246300
C	-2.85824300	-0.82002400	1.82711000
C	-4.24117900	-0.83253100	1.97050300
C	-2.04825900	-1.60492600	2.66044600
C	-4.84123700	-1.63413900	2.94934200
H	-4.86379500	-0.21778300	1.32351000
C	-2.62715900	-2.40717000	3.63838500
H	-0.96635800	-1.58463000	2.52189100
C	-4.02252400	-2.40995000	3.76502300
H	-4.48095300	-3.03920400	4.52694300
C	3.23190100	-1.09405300	-0.39820200
C	4.01556700	-0.43524300	-1.35002800
C	3.39068300	-2.46111400	-0.18675000
C	4.98115000	-1.13222100	-2.07268100
H	3.85755800	0.62481600	-1.54149900
C	4.35166400	-3.18313800	-0.90370700
H	2.74270600	-2.95854000	0.53205900
C	5.13666600	-2.50247300	-1.83209800
H	5.88447100	-3.05499200	-2.39946000
C	2.83994500	0.72244300	1.87329800
C	2.01677300	1.43828200	2.75472500
C	4.22272000	0.76080200	2.00879700
C	2.58255100	2.19701300	3.77346300
H	0.93499400	1.40020500	2.62012600
C	4.81010000	1.51907800	3.03005700
H	4.85566200	0.19949700	1.32394300
C	3.97879600	2.22532300	3.89350000
H	4.42723200	2.81839500	4.68982100
O	1.00501500	-1.21660200	1.20410100
O	-1.00679700	1.13929600	1.26921600
O	1.28910600	-1.17460500	-3.07676500
C	2.06095900	-2.14573600	-3.74782800
H	1.47080900	-2.66724800	-4.51206000
H	2.87741800	-1.60429500	-4.22736000
O	-1.29561900	1.32456400	-3.00995400
C	-2.04076900	2.32723500	-3.66400100
H	-1.43157300	2.85484900	-4.40899900
H	-2.86359900	1.81428100	-4.16332400
H	-2.44767900	3.04685900	-2.94266900
H	2.47551500	-2.87355200	-3.03895200
H	-0.23705400	3.83368100	-2.72524800
H	0.24280300	-3.69862000	-2.90999400
O	1.61175400	4.64596000	-1.25057700
O	-1.60228700	-4.58875600	-1.47525000
C	2.60941200	5.02018700	-0.32592000
H	2.29988800	4.80465500	0.70401800
H	3.55596000	4.50441100	-0.52959400
H	2.74879400	6.09442400	-0.44965300
C	-2.60412700	-5.00885900	-0.57507300
H	-2.30126800	-4.84108800	0.46576500
H	-3.55112500	-4.48680800	-0.75991300
H	-2.73941700	-6.07645900	-0.74971500
C	-4.36982300	4.73351400	-0.58135900
H	-3.53856200	5.25347400	-1.07118600
H	-5.30193900	5.14138300	-0.98136200
H	-4.31929100	4.97397000	0.48444800
C	-5.89110000	0.57226900	-3.01426200
H	-6.75166500	0.10144300	-2.52583700
H	-6.27832800	1.29456500	-3.73828900
H	-5.35729200	-0.20988100	-3.56190300
C	-6.34175100	-1.65385400	3.10040300
H	-6.73647900	-0.64300000	3.24391200
H	-6.82308300	-2.06686900	2.20730000
H	-6.64603600	-2.26057100	3.95719800
C	-1.77425000	-3.24251700	4.55961200
H	-0.77017000	-3.37507400	4.14967100
H	-1.67332700	-2.76209800	5.53914000
H	-2.21582500	-4.23047600	4.72323100
C	1.71993700	2.96824000	4.74025200
H	0.66948500	2.93020700	4.44288400
H	1.80067100	2.55462500	5.75134800
H	2.02505100	4.01866000	4.79239900
C	6.31051000	1.56053500	3.17753300
H	6.72027800	0.55482100	3.31609200
H	6.78359300	1.98374200	2.28497900
H	6.60792800	2.16746500	4.03657400
C	4.50972400	-4.66589500	-0.67966300
H	3.57393700	-5.19395200	-0.89090800
H	5.28911000	-5.08512600	-1.32135900
H	4.77457900	-4.88013800	0.36068400
C	5.84468600	-0.42945200	-3.09004900
H	6.80473900	-0.13178100	-2.65336500
H	6.06197900	-1.07902800	-3.94329600
H	5.35671000	0.47470900	-3.46389000

3l

C	0.04690100	-2.42942600	2.98494300
C	0.40998100	-2.46556300	1.64428100
C	-0.96830600	-1.56276100	3.39846700
C	-0.22941000	-1.63627400	0.70670400
C	-1.21591500	-0.75972700	1.14316600
C	-1.60606100	-0.72570400	2.48737100
H	-2.36502800	-0.02659700	2.81356600
C	0.22933600	-1.63616500	-0.70709100
C	-0.41011500	-2.46525000	-1.64480700
C	1.21590000	-0.75961000	-1.14340200
C	-0.04703800	-2.42890800	-2.98546300
C	1.60606900	-0.72541200	-2.48759600
C	0.96825400	-1.56227000	-3.39883400
H	2.36510400	-0.02632400	-2.81367700
P	1.97699500	0.40329000	0.05931700
P	-1.97699200	0.40337200	-0.05937600
C	-3.29235800	-0.57551200	-0.84790400
C	-4.02817600	-1.54616300	-0.16778500
C	-3.53107200	-0.34724300	-2.20421100
C	-5.02906500	-2.27147600	-0.81424200
H	-3.80400800	-1.76551400	0.87492500
C	-4.52181200	-1.05497000	-2.88322100
H	-2.91493700	0.37895200	-2.72932700
C	-5.26323900	-2.00222000	-2.16823800
C	-2.76236900	1.64086800	1.00910200
C	-4.13993500	1.77684700	1.16964200
C	-1.88643800	2.48775500	1.69547400
C	-4.66218000	2.76564700	2.00376600
H	-4.81974700	1.11031800	0.64282200
C	-2.37723500	3.48230200	2.53935600
H	-0.81039700	2.34616000	1.57989000
C	-3.76663300	3.60639600	2.67183800
C	3.29227900	-0.57575400	0.84777900
C	4.02815200	-1.54632100	0.16758900
C	3.53086600	-0.34768100	2.20413700
C	5.02894400	-2.27175000	0.81405100
H	3.80408500	-1.76551400	-0.87517600
C	4.52150400	-1.05554400	2.88316200
H	2.91469700	0.37845800	2.72929100
C	5.26296600	-2.00271400	2.16812300
C	2.76248700	1.64088600	-1.00894800
C	1.88664400	2.48789100	-1.69528800
C	4.14007100	1.77685400	-1.16933300
C	2.37754600	3.48254100	-2.53898300
H	0.81058900	2.34631700	-1.57980700
C	4.66242200	2.76574200	-2.00328600
H	4.81981700	1.11022900	-0.64255000
C	3.76696200	3.60660900	-2.67132300
O	1.08945300	1.03878300	1.08733500
O	-1.08946000	1.03897700	-1.08733200
O	1.39160200	-3.25947300	1.14870600
C	2.21360400	-3.93235500	2.07712700
H	1.65009200	-4.69482800	2.62959500
H	2.99905400	-4.41374700	1.49313700
O	-1.39179600	-3.25916200	-1.14935900
C	-2.21372700	-3.93198700	-2.07787600
H	-1.65012500	-4.69429600	-2.63048000
H	-2.99911900	-4.41356300	-1.49396000
H	-2.66836900	-3.22514100	-2.78339600
H	2.66815000	-3.22556900	2.78277000
H	-0.52599600	-3.04596100	-3.73562300
H	0.52580200	-3.04666500	3.73498500
O	1.25646600	-1.59837300	-4.72762500
O	-1.25650500	-1.59904600	4.72725800

C	2.23019500	-0.69392600	-5.20167000
H	1.94091000	0.34520000	-5.00248900
H	3.20905200	-0.88538000	-4.74502700
H	2.29496800	-0.85580100	-6.27779000
C	-2.23008000	-0.69452000	5.20147200
H	-1.94063600	0.34459100	5.00243700
H	-3.20898100	-0.88575000	4.74483300
H	-2.29484000	-0.85655100	6.27756800
C	-4.78416700	-0.85540700	-4.35259200
H	-4.68667100	-1.79933000	-4.89709300
H	-5.80048600	-0.48865800	-4.53298900
H	-4.08149300	-0.13273900	-4.77311100
C	-5.80712500	-3.34336600	-0.09551500
H	-6.70032700	-2.93512200	0.39207100
H	-6.13447900	-4.11804100	-0.79379600
H	-5.19347100	-3.80536100	0.68262100
C	-6.14411600	2.93553500	2.21061100
H	-6.49455000	3.90204700	1.83233300
H	-6.69780000	2.14957100	1.69111300
H	-6.39683800	2.90091200	3.27411300
C	-1.44271400	4.38420200	3.30284500
H	-0.51248700	3.85844800	3.53211000
H	-1.17730300	5.27173500	2.71669000
H	-1.90154600	4.72250700	4.23540800
C	1.44313400	4.38457900	-3.30243700
H	0.51284500	3.85894700	-3.53172700
H	1.17782400	5.27212300	-2.71625100
H	1.90201300	4.72287900	-4.23497900
C	6.14438000	2.93555900	-2.21003800
H	6.49485400	3.90206000	-1.83176900
H	6.69799000	2.14957700	-1.69049000
H	6.39716200	2.90089400	-3.27352400
C	4.78366400	-0.85623200	4.35260200
H	4.68626500	-1.80028500	4.89689500
H	5.79990000	-0.48934300	4.53319300
H	4.08082700	-0.13376200	4.77318800
C	5.80706300	-3.34358000	0.09530400
H	6.70102300	-2.93555600	-0.39107100
H	6.13326600	-4.11895900	0.79334800
H	5.19391200	-3.80463400	-0.68378000
O	-6.20893700	-2.73263300	-2.85593000
O	-4.29216900	4.55964400	3.51930300
O	4.29257700	4.55995700	-3.51862700
O	6.20851400	-2.73333800	2.85580100
C	7.52815800	-2.24337800	2.68583800
H	8.17754500	-2.86625200	3.30278500
H	7.84631400	-2.31111800	1.63830800
H	7.60418600	-1.19844500	3.01157100
C	4.35663100	5.84957900	-2.93321300
H	4.97186400	5.83908800	-2.02493200
H	4.81049800	6.51012500	-3.67383700
H	3.35722200	6.22159300	-2.67781100
C	-4.35612000	5.84937200	2.93410800
H	-4.97114400	5.83901900	2.02568700
H	-4.81017900	6.50976700	3.67475100
H	-3.35666200	6.22146500	2.67900800
C	-7.52854700	-2.24277800	-2.68539500
H	-8.17811600	-2.86556200	-3.30224000
H	-7.84632300	-2.31075100	-1.63776300
H	-7.60473600	-1.19777900	-3.01088000

3n

C	-0.39160700	-2.13483000	-2.83950800
C	-0.58724600	-2.09246400	-1.46109000
H	0.73589800	-1.42018400	-4.52393800
C	0.58103900	-1.38145400	-3.45226000
C	0.14880900	-1.27881500	-0.63708100
C	1.12690100	-0.46190200	-1.24992300
C	1.33675900	-0.53176400	-2.62507900
H	2.07642700	0.11336200	-3.08835200
C	-0.15042100	-1.19192500	0.81118100
C	0.60351600	-1.87907500	1.72854300
C	-1.14046800	-0.32021000	1.32056300
C	0.41513400	-1.74661700	3.10260400
C	-1.34708800	-0.22055500	2.69449300
C	-0.57343600	-0.94334500	3.61937800
H	-2.09513400	0.46656300	3.07651900
H	-0.72336500	-0.84568500	4.68806400
P	-2.01044700	0.77614600	0.14038000
P	1.99895500	0.77026900	-0.21348500
C	3.42542100	-0.11444000	0.49848700
C	3.99525600	-1.25186900	-0.07795700
C	3.95322000	0.40174800	1.68655900
C	5.09733900	-1.85800100	0.52076500
H	3.56583600	-1.67101900	-0.98432800
C	5.06135000	-0.19951800	2.27792000
H	3.47612400	1.26572300	2.14101100
C	5.63471200	-1.32764000	1.69291900
H	5.53620400	-2.74469000	0.07348300
H	5.47258100	0.20618500	3.19726000
H	6.49725700	-1.79916000	2.15479000
C	2.65087500	1.94930800	-1.42775200
C	4.00165400	2.05831200	-1.75587400
C	1.70400500	2.78689100	-2.03289900
C	4.41142200	3.00519700	-2.69334600
H	4.73243800	1.40738400	-1.28270800
C	2.12124200	3.72749400	-2.96752900
H	0.65209700	2.67787000	-1.76920400
C	3.47271400	3.83650300	-3.29702300
H	5.46279100	3.09317100	-2.94993600
H	1.39232500	4.37827300	-3.44089600
H	3.79470500	4.57319500	-4.02736500
C	-3.40652800	-0.19840100	-0.51008800
C	-4.04706700	-1.21237600	0.20681900
C	-3.83894000	0.12190400	-1.80050400
C	-5.12810300	-1.88543100	-0.35733700
H	-3.69113800	-1.48330400	1.19805900
C	-4.92512000	-0.54670000	-2.35932600
H	-3.30601800	0.89001400	-2.35411600
C	-5.57198800	-1.54716700	-1.63547500
H	-5.62437600	-2.67495800	0.19881000
H	-5.26293700	-0.29175000	-3.35925200
H	-6.41903400	-2.06978200	-2.07046100
C	-2.69827000	2.07308500	1.20415700
C	-1.76898400	2.98000900	1.73310600
C	-4.05687900	2.20985400	1.48538000
C	-2.21228800	4.01660500	2.54605800
H	-0.71052400	2.84987400	1.50699200
C	-4.49282100	3.25317500	2.30077900
H	-4.77351800	1.50661300	1.06925600
C	-3.57187800	4.15287700	2.82943600
H	-1.49770000	4.72115700	2.96045800
H	-5.55048100	3.36360200	2.52000500
H	-3.91297600	4.96520800	3.46456800
O	-1.20026600	1.33911900	-0.98798200
O	1.20715500	1.44266000	0.86708500
O	-1.58182900	-2.96476800	-1.11461600
O	-1.27368800	-3.01969100	-3.38811600
C	-2.21027900	-3.29701300	-2.34593800
H	-2.45240700	-4.35964500	-2.35399200
H	-3.10169600	-2.66983600	-2.47885100
O	1.32669200	-2.52804300	3.75288100
O	1.62174000	-2.76139500	1.49185600
C	2.27627200	-2.88469400	2.74742000
H	2.58530300	-3.91907800	2.89593400
H	3.12588100	-2.18891600	2.78241800

3o

C	-0.15413400	-2.02713500	-2.99712800
C	-0.42787500	-2.10119900	-1.63368000
H	0.99051500	-1.10630700	-4.56433800
C	0.78694400	-1.16324300	-3.50145400
C	0.19953700	-1.29507400	-0.71507400
C	1.16123100	-0.38265700	-1.21443900
C	1.44872700	-0.33797300	-2.57455400
H	2.18009900	0.37155700	-2.94703300
C	-0.19957000	-1.29503100	0.71516300
C	0.42784800	-2.10109900	1.63381400
C	-1.16125900	-0.38258200	1.21447900
C	0.15411200	-2.02695800	2.99725800
C	-1.44874700	-0.33781500	2.57459300
C	-0.78696400	-1.16303600	3.50153700
H	-2.18011200	0.37174100	2.94703800
H	-0.99052900	-1.10603500	4.56441900
P	-1.98161000	0.74235800	0.01713100
P	1.98160900	0.74233200	-0.01714700
C	3.21245800	-0.34594900	0.76661200
C	4.04874400	-1.19776900	0.03804500
C	3.20704200	-0.38944900	2.15735700
C	4.89886600	-2.08122600	0.69556600
H	4.02110500	-1.18542900	-1.05071600
C	4.04192700	-1.27986700	2.84130800
H	2.52188900	0.26192500	2.69699400
C	4.88019600	-2.10654600	2.09569200
H	5.53885600	-2.80083000	2.61738300
C	2.81967800	1.95459500	-1.06909400
C	4.19909800	2.04634900	-1.21151900
C	1.97192700	2.85914800	-1.72439300
C	4.75777600	3.04501000	-2.01925000
H	4.85140600	1.34763600	-0.69105600
C	2.50908200	3.85747000	-2.52955000
H	0.89279100	2.76269600	-1.59732800
C	3.90192200	3.93124300	-2.66573200
H	4.32806100	4.70963200	-3.29751600
C	-3.21240900	-0.34596800	-0.76664900
C	-4.04865600	-1.19783800	-0.03809600
C	-3.20698100	-0.38945500	-2.15739600
C	-4.89873100	-2.08133000	-0.69563100
H	-4.02102000	-1.18551300	1.05066600
C	-4.04180700	-1.27991800	-2.84136000
H	-2.52186300	0.26195800	-2.69702700
C	-4.88004500	-2.10664200	-2.09575600
H	-5.53866700	-2.80095500	-2.61745700
C	-2.81973600	1.95464200	1.06900600
C	-1.97202700	2.85930500	1.72420700
C	-4.19915300	2.04625600	1.21154400
C	-2.50921900	3.85759400	2.52938000
H	-0.89289000	2.76294700	1.59707800

C	-4.75786800	3.04488700	2.01928600
H	-4.85143200	1.34744400	0.69117800
C	-3.90205600	3.93122100	2.66568200
H	-4.32822400	4.70956800	3.29749800
O	-1.11783900	1.39175100	-1.02163600
O	1.11787600	1.39173000	1.02164900
O	-1.43134600	-3.00306100	-1.41591200
O	-0.98702800	-2.88006500	-3.66859000
C	-1.62921500	-3.65868800	-2.66073400
H	-1.16832500	-4.65475400	-2.62769400
H	-2.69854200	-3.71218600	-2.87292500
O	0.98699700	-2.87985400	3.66876800
O	1.43131400	-3.00297600	1.41608900
C	1.62927700	-3.65847500	2.66096600
H	1.16851700	-4.65460300	2.62801200
H	2.69861500	-3.71181900	2.87315600
C	1.62144800	4.85169400	-3.23406300
H	0.57048900	4.56474300	-3.15328900
H	1.73255500	5.85057300	-2.79838400
H	1.87718100	4.92784300	-4.29585700
C	6.25448300	3.14651600	-2.17361900
H	6.53111300	3.98593800	-2.81658000
H	6.73982800	3.28996900	-1.20278200
H	6.66905500	2.23304800	-2.61281200
C	5.81622200	-3.00186500	-0.06904800
H	6.86603700	-2.79831000	0.16713100
H	5.62228000	-4.04983300	0.18192400
H	5.68418300	-2.88461400	-1.14759200
C	4.00468300	-1.35636900	4.34696300
H	3.03719200	-1.74158800	4.68804400
H	4.78986100	-2.01365600	4.73104700
H	4.13969800	-0.36701200	4.79463500
C	-4.00453500	-1.35643000	-4.34701300
H	-3.03710300	-1.74182800	-4.68806200
H	-4.78981800	-2.01358000	-4.73111700
H	-4.13936000	-0.36705100	-4.79469100
C	-5.81605900	-3.00200800	0.06897000
H	-6.86588400	-2.79843200	-0.16715400
H	-5.62213600	-4.04996300	-0.18207000
H	-5.68397600	-2.88481800	1.14751400
C	-6.25457900	3.14628800	2.17367600
H	-6.66927300	2.23215700	2.61136000
H	-6.53120500	3.98471500	2.81793300
H	-6.73981600	3.29133600	1.20301600
C	-1.62162700	4.85191600	3.23380700
H	-0.57065100	4.56503700	3.15300200
H	-1.73282200	5.85076700	2.79808600
H	-1.87731900	4.92809800	4.29560800

3p

C	-0.12141400	-2.64249300	-2.89358600
C	-0.43445600	-2.61462200	-1.53685200
H	1.05597900	-1.82207500	-4.49166800
C	0.81558700	-1.79614700	-3.43524500
C	0.16581200	-1.73940700	-0.66550300
C	1.12064200	-0.84218400	-1.20120300
C	1.43448800	-0.88650500	-2.55709300
H	2.15867900	-0.18978500	-2.96595500
C	-0.21404400	-1.67999800	0.76423200
C	0.41627400	-2.47480000	1.68905300
C	-1.13552900	-0.72374300	1.25200200
C	0.18339700	-2.35653600	3.05588800
C	-1.38549900	-0.63287800	2.61846800
C	-0.72334400	-1.45147600	3.55346500
H	-2.08711900	0.10646300	2.98993900
H	-0.90756100	-1.36354100	4.61779600
P	-1.95055300	0.38512700	0.03152900
P	1.91977400	0.36000400	-0.05698300
C	3.17152000	-0.63880500	0.80207100
C	3.86930900	-1.67321500	0.19614000
C	3.33584000	-0.40217700	2.16766800
C	4.79642800	-2.45144500	0.90110100
H	3.66337900	-1.90091700	-0.84665900
C	4.27766100	-1.10528900	2.91696600
H	2.69743000	0.34721300	2.62137200
C	5.05968300	-2.06586100	2.23092800
C	2.73842400	1.49677200	-1.19452900
C	4.09759700	1.50635600	-1.45281100
C	1.87870900	2.33876800	-1.90678700
C	4.66058600	2.38629000	-2.39039000
H	4.73716300	0.81026000	-0.91788900
C	2.37605600	3.24549600	-2.83530400
H	0.81165100	2.24640800	-1.72060400
C	3.78413600	3.30475600	-2.99563000
C	-3.21660100	-0.68018400	-0.71839000
C	-3.86101100	-1.70453200	-0.02501100
C	-3.43595400	-0.52753000	-2.07969800
C	-4.79318500	-2.52579400	-0.65344200
H	-3.60191500	-1.86767000	1.01509000
C	-4.34647300	-1.33050300	-2.77974700
H	-2.85166600	0.22736800	-2.59691600
C	-5.08108600	-2.25684100	-2.01508100
C	-2.75242000	1.60999500	1.08575400
C	-1.89208200	2.52683500	1.69758000
C	-4.10216400	1.60821500	1.39023300
C	-2.38329000	3.49606300	2.56454400
H	-0.82935500	2.44097300	1.48653600
C	-4.65711800	2.54730200	2.27293600
H	-4.74016400	0.85477200	0.93748600
C	-3.78647900	3.53424100	2.76856800
O	-1.10500700	1.00865500	-1.04010200
O	1.06126500	1.07394700	0.94527700
O	-1.37280500	-3.56991700	-1.26290100
O	-0.85803000	-3.61847500	-3.50413500
C	-1.81762100	-4.03490800	-2.53237400
H	-1.86770700	-5.12475500	-2.52260200
H	-2.79257300	-3.58520600	-2.76210300
O	0.96547900	-3.25454200	3.72522900
O	1.34027400	-3.45242900	1.45962200
C	1.81420600	-3.85009600	2.74129500
H	1.75262800	-4.93728500	2.82878800
H	2.84130800	-3.49145500	2.87580900
C	1.41433600	4.05456000	-3.72336300
C	6.16477400	2.21456700	-2.69516700
C	5.36736700	-3.68737100	0.17325300
C	4.39080800	-0.89767000	4.43818900
C	-4.40269800	-1.15411800	-4.31331600
C	-5.37279000	-3.75300100	0.07202700
C	-6.14355400	2.35943500	2.64748800
C	-1.41612700	4.39884700	3.35053200
C	4.10587500	-2.22970700	5.15717500
H	3.07155100	-2.54610000	4.98248300
H	4.78675000	-3.01681200	4.82380300
H	4.23609300	-2.09938500	6.23795000
C	3.35075800	0.11850100	4.93202300
H	3.42158500	0.19616800	6.02157700

H	3.51993200	1.11626400	4.51338400
H	2.33047000	-0.19301000	4.68409100
C	5.77832100	-0.38069000	4.85755400
H	6.07745000	0.48405600	4.25469600
H	5.74502400	-0.06272900	5.90564000
H	6.53959900	-1.15726200	4.77232000
C	6.26659000	-3.24469500	-0.99340900
H	7.12926200	-2.66837600	-0.64224900
H	6.64285800	-4.12202700	-1.53153200
H	5.71601300	-2.62286000	-1.70700700
C	6.13907000	-4.68024400	1.05893300
H	6.31691900	-5.58920900	0.47388600
H	7.11146500	-4.30954600	1.38507900
H	5.56501500	-4.95308200	1.94851300
C	4.17466300	-4.48598800	-0.40019100
H	3.51160000	-4.82045100	0.40416400
H	3.56882600	-3.90901200	-1.10298200
H	4.54857200	-5.36949600	-0.92869300
O	6.10353500	-2.64852700	2.92030700
O	-4.29196000	4.59306700	3.49621200
O	-6.11096200	-2.96872600	-2.59619400
O	4.28770600	4.31287000	-3.79333300
C	7.36063500	-2.10382800	2.54721800
H	8.11595900	-2.62437200	3.13960000
H	7.56047900	-2.25869500	1.48136700
H	7.40010600	-1.02957200	2.75906300
C	4.79104500	5.39863200	-3.02992200
H	5.63303400	5.08723100	-2.40230200
H	5.12829300	6.15372800	-3.74330900
H	4.01186000	5.82034500	-2.38583100
C	7.00129500	2.66106900	-1.48402400
H	6.72261400	2.10387200	-0.58361300
H	8.06577300	2.48569400	-1.67790900
H	6.86783000	3.72637500	-1.27122500
C	6.66954500	2.93657900	-3.95639900
H	6.04096400	2.71562900	-4.82274400
H	6.72119100	4.01975500	-3.84459500
H	7.68452900	2.58259500	-4.16836400
C	6.43529200	0.71267100	-2.94158100
H	6.22533300	0.09387200	-2.06530200
H	5.82740800	0.34251700	-3.77386100
H	7.49034600	0.56887300	-3.19762200
C	1.67306700	3.67748600	-5.19385000
H	0.98614000	4.23069700	-5.84490700
H	2.69733600	3.91511400	-5.48967000
H	1.50013700	2.60713000	-5.34984500
C	-0.04796500	3.70891200	-3.40178200
H	-0.25708800	2.64084900	-3.51896800
H	-0.32291500	3.99004200	-2.37982200
H	-0.69908600	4.25862300	-4.08929600
C	1.57504700	5.57541800	-3.55480500
H	2.50486000	5.93579500	-3.99685700
H	0.74791700	6.08450700	-4.06222700
H	1.54409600	5.86101200	-2.49743800
C	0.04413800	4.06639000	3.00682600
H	0.28872400	3.01725400	3.20071800
H	0.27632600	4.27304700	1.95678700
H	0.70123600	4.68669000	3.62509300
C	-1.61640200	4.13529000	4.85450200
H	-0.92539800	4.75723000	5.43542600
H	-2.63723700	4.36898400	5.16517800
H	-1.40744800	3.08601400	5.08949400
C	-1.62553300	5.89655000	3.06673000
H	-2.55075000	6.26743200	3.50977200
H	-0.79742800	6.46604000	3.50344300
H	-1.63628800	6.09671000	1.98949200
C	-4.84949400	5.59660200	2.66121300
H	-5.69624600	5.20878900	2.08441200
H	-5.19333500	6.39719300	3.31972000
H	-4.10086600	5.98744400	1.96357200
C	-6.35296300	0.87969100	3.04347700
H	-7.39516400	0.72600000	3.34280500
H	-6.14173000	0.18531200	2.22577300
H	-5.71096800	0.61349000	3.88965800
C	-6.63117600	3.18077300	3.85341400
H	-5.96935600	3.05982200	4.71462100
H	-6.72101500	4.24684200	3.64371500
H	-7.62732700	2.81895600	4.13131200
C	-7.03464600	2.66549000	1.43173900
H	-6.94169000	3.70962200	1.11640400
H	-6.77031900	2.03426300	0.57706200
H	-8.08580900	2.47954900	1.68049300
C	-2.95612200	-1.16883300	-4.85791100
H	-2.32886600	-0.38252200	-4.43177600
H	-2.46971200	-2.13047100	-4.66106400
H	-2.97995500	-1.01951600	-5.94285200
C	-5.03319600	0.20697700	-4.65451300
H	-5.03697200	0.35605000	-5.74019900
H	-6.06720900	0.27463300	-4.30076000
H	-4.46940500	1.02805500	-4.20042000
C	-5.14152000	-2.26444700	-5.07997800
H	-4.78638700	-3.25737000	-4.79014700
H	-6.22360700	-2.24103800	-4.94683000
H	-4.94200900	-2.12978800	-6.14864800
C	-4.92657800	-5.01554300	-0.68885400
H	-5.33036700	-5.90994800	-0.20023300
H	-5.27734600	-4.99906400	-1.72373700
H	-3.83305400	-5.09033300	-0.68030000
C	-4.82298100	-3.86089200	1.50208400
H	-3.73006400	-3.92472000	1.51466000
H	-5.12976100	-3.01160200	2.12294900
H	-5.21603400	-4.77101600	1.96580600
C	-6.90774300	-3.72859400	0.17326100
H	-7.38026600	-3.90321500	-0.79366500
H	-7.23895700	-4.52172200	0.85282600
H	-7.26089800	-2.77340700	0.57862700
C	-7.30518200	-2.20602000	-2.68684000
H	-7.16119400	-1.31491800	-3.30688200
H	-8.05607000	-2.85247000	-3.14595200
H	-7.64557300	-1.89167300	-1.69393100

3q

C	0.35388900	-1.65901200	2.94156200
C	0.53553900	-1.65292400	1.56704400
H	-0.64737900	-0.80379700	4.64160200
C	-0.51207200	-0.79790200	3.56687400
C	-0.13129400	-0.79113100	0.73119000
C	-1.02112100	0.11571000	1.35025300
C	-1.20058000	0.09808000	2.73216900
H	-1.87800300	0.81129200	3.19066800
C	0.13122700	-0.79116800	-0.73126000
C	-0.53576300	-1.65292200	-1.56704000
C	1.02109000	0.11555500	-1.35043000
C	-0.35420100	-1.65909800	-2.94156800
C	1.20046900	0.09784200	-2.73235700
C	0.51180000	-0.79809500	-3.56697600

H	1.87797300	0.81093100	-3.19091800
H	0.64703400	-0.80404000	-4.64171300
P	1.95660600	1.28243300	-0.28269900
P	-1.95647400	1.28272700	0.28249900
C	-3.37422100	0.27688000	-0.25031200
C	-4.16338900	-0.45921500	0.63796300
C	-3.60383500	0.19223100	-1.62518200
C	-5.18972800	-1.26172000	0.15175200
H	-3.96750400	-0.41423700	1.70735600
C	-4.62524700	-0.62133000	-2.10863200
H	-2.96386300	0.75957700	-2.29592000
C	-5.41757900	-1.34411700	-1.22122600
H	-5.80318300	-1.83466500	0.84018000
H	-4.79559300	-0.70047100	-3.17789300
H	-6.20876500	-1.98447700	-1.59940600
C	-2.53072200	2.55533600	1.43545200
C	-3.87847100	2.80091300	1.69918700
C	-1.53656500	3.34827700	2.02627300
C	-4.23664300	3.83410300	2.56330800
H	-4.64677000	2.19267900	1.22964300
C	-1.90368000	4.37611000	2.88712300
H	-0.48915500	3.14463300	1.80385200
C	-3.25128600	4.61740400	3.15652400
H	-5.28482400	4.02803000	2.76911200
H	-1.13823200	4.99270800	3.34820700
H	-3.53304100	5.42266700	3.82856400
C	3.37414000	0.27637800	0.25025300
C	4.16315100	-0.45997000	-0.63795000
C	3.60373300	0.19184300	1.62513500
C	5.18932500	-1.26263700	-0.15164900
H	3.96725800	-0.41507500	-1.70734700
C	4.62497900	-0.62186800	2.10867200
H	2.96384900	0.75937700	2.29579800
C	5.41716000	-1.34492000	1.22133900
H	5.80266700	-1.83579300	-0.84000200
H	4.79533200	-0.70090200	3.17793900
H	6.20821200	-1.98540200	1.59959200
C	2.53119500	2.55495100	-1.43557900
C	1.53726600	3.34828300	-2.02626300
C	3.87901500	2.80014100	-1.69930300
C	1.90468400	4.37612200	-2.88697000
H	0.48980200	3.14493800	-1.80382300
C	4.23749300	3.83334200	-2.56328900
H	4.64711700	2.19154900	-1.22989700
C	3.25236600	4.61703800	-3.15635500
H	1.13943400	4.99303600	-3.34795700
H	5.28573000	4.02696600	-2.76908800
H	3.53434800	5.42233300	-3.82826100
O	1.22711900	1.83578300	0.90392600
O	-1.22697400	1.83593000	-0.90418500
O	1.46899200	-2.60769600	1.24120400
O	1.16794800	-2.62465400	3.48210600
C	1.80273300	-3.25113600	2.42038900
O	-1.16833700	-2.62472300	-3.48200900
O	-1.46921100	-2.60765700	-1.24107700
C	-1.80317200	-3.25105700	-2.42022800
F	-1.43385300	-4.53208200	-2.36047600
F	-3.12075800	-3.23621600	-2.60290500
F	1.43310600	-4.53209100	2.36062400
F	3.12030100	-3.23661300	2.60316300

3r

C	2.61544400	-2.92786500	3.04424100
C	0.59202000	-1.80971500	2.91974000
C	0.69394300	-1.86499300	1.52230900
C	2.25430900	-3.59905400	1.73471400
H	-0.42977300	-0.97883100	4.59184800
H	3.24036100	-2.04699200	2.84546200
C	-0.36563700	-0.99440100	3.50900800
C	-0.10780800	-1.04408700	0.73541300
H	3.14803600	-3.92136100	1.19526900
C	-1.04211200	-0.18916300	1.32936100
C	-1.18031700	-0.19041200	2.71987400
H	-1.89524300	0.46965900	3.20011200
C	0.10788300	-1.04364600	-0.73577500
C	-0.69387300	-1.86414500	-1.52309400
C	1.04205700	-0.18828000	-1.32930600
C	-0.59214600	-1.80797800	-2.92050500
C	1.18011100	-0.18868900	-2.71983300
C	-2.25397800	-3.59832500	-1.73639000
C	0.36539700	-0.99224500	-3.50937500
H	1.89495600	0.47170300	-3.19975500
C	-2.61546400	-2.92632500	-3.04540400
H	-1.60560100	-4.46316400	-1.92860000
H	0.42941700	-0.97599500	-4.59221100
H	-3.14240900	-3.60944200	-3.71389200
P	1.99220500	0.96527700	-0.27619400
P	-1.99213300	0.96495500	0.27677100
C	-3.44256000	0.01493200	-0.28761900
C	-3.93549300	-1.10511200	0.38604100
C	-4.05675300	0.44087600	-1.46946900
C	-5.04452700	-1.78536100	-0.11220300
H	-3.43876300	-1.45543100	1.28700000
C	-5.17026700	-0.23574900	-1.96196100
H	-3.63863700	1.29142500	-2.00084500
C	-5.66358700	-1.34924500	-1.28307100
H	-5.42619000	-2.65585600	0.41342200
H	-5.64732200	0.09919000	-2.87809000
H	-6.53028500	-1.87908800	-1.66759600
C	-2.58456500	2.20878500	1.45612100
C	-3.92082400	2.35502900	1.82666600
C	-1.60672400	3.06845000	1.97587400
C	-4.28544800	3.36165600	2.71995400
H	-4.67605800	1.68784300	1.41944500
C	-1.97877900	4.06867400	2.86665100
H	-0.56615700	2.93123200	1.68183300
C	-3.31610500	4.21537300	3.23769200
H	-5.32552900	3.47849500	3.00872800
H	-1.22562900	4.73591200	3.27430500
H	-3.60188300	4.99874900	3.93350100
C	3.44271600	0.01490300	0.28740800
C	3.93571400	-1.10455900	-0.38717200
C	4.05684300	0.43988000	1.46963800
C	5.04473800	-1.78519800	0.11054200
H	3.43903000	-1.45412500	-1.28845100
C	5.17036400	-0.23712800	1.96160000
H	3.63866000	1.28996200	2.00170700
C	5.66373700	-1.35004300	1.28180300
H	5.42643900	-2.65525200	-0.41578500
H	5.64738300	0.09706200	2.87802200
H	6.53043400	-1.88018700	1.66591500
C	2.58441900	2.20973600	-1.45498700
C	1.60647800	3.06945900	-1.97445000
C	3.92069800	2.35641700	-1.82529800
C	1.97844900	4.07017800	-2.86470500
H	0.56590500	2.93192200	-1.68057500

C	4.28523500	3.36353600	-2.71806300
H	4.67601300	1.68918400	-1.41830600
C	3.31579000	4.21731000	-3.23551700
H	1.22521900	4.73747000	-3.27212300
H	5.32532900	3.48071200	-3.00665500
H	3.60150000	5.00106600	-3.93092500
O	1.28549200	1.58528100	0.89130300
O	-1.28520500	1.58563500	-0.89023600
H	-3.24048700	-2.04568300	-2.84591600
H	-3.14754200	-3.92119000	-1.19700800
H	3.14236800	-3.61135100	3.71236900
H	1.60607800	-4.46415600	1.92622900
O	1.42664200	-2.53672600	3.71829700
O	1.58633500	-2.68046400	0.88661000
O	-1.58604800	-2.68015900	-0.88778900
O	-1.42683600	-2.53454400	-3.71941100

3s

C	-0.40590200	-2.97732200	-1.65204900
C	0.26618600	-1.76809600	-1.90908700
C	-0.27888300	-0.67908900	-1.13736600
C	-1.30120200	-1.06086200	-0.32621300
S	-1.66179000	-2.76370300	-0.46100000
C	0.27913800	0.68773900	-1.13267200
C	1.30112900	1.06386700	-0.31847400
C	-0.26472900	1.78184200	-1.89797000
S	1.66230600	2.76751700	-0.44175500
C	0.40770000	2.98908900	-1.63262200
C	1.35289200	-1.75164100	-2.79729300
H	1.88918400	-0.82246300	-2.97282000
C	-0.02563900	-4.16615000	-2.28209800
H	-0.54816500	-5.09569900	-2.07936900
C	-1.35034100	1.77150900	-2.78759600
H	-1.88697600	0.84381200	-2.96975400
C	0.02870700	4.18204300	-2.25559600
H	0.55154100	5.10998600	-2.04642800
C	-1.04038000	4.14935700	-3.13609200
H	-1.35418900	5.06587100	-3.62636800
C	-1.73005400	2.95213200	-3.39804200
H	-2.57517500	2.95858500	-4.07901400
C	1.73405000	-2.92825600	-3.41452800
H	2.58017400	-2.93009100	-4.09428200
C	1.04457300	-4.12740400	-3.16101200
H	1.35931500	-5.04064800	-3.65675000
P	-2.12331700	0.05492200	0.85569700
P	2.12223100	-0.06006700	0.85627700
O	-1.26140200	1.17747100	1.34493400
O	1.25921000	-1.18457900	1.33908000
C	-3.55320400	0.67320600	-0.07864600
C	-4.48894200	-0.17590300	-0.67910400
C	-3.63421100	2.05260500	-0.27184300
C	-5.50987000	0.35839200	-1.45705000
H	-4.41553600	-1.25317100	-0.54310400
C	-4.65477200	2.58316500	-1.05825000
H	-2.88056700	2.69152200	0.18113000
C	-5.59077900	1.73845500	-1.64719100
H	-6.23843400	-0.29970200	-1.92046800
H	-4.71138600	3.65572900	-1.21699500
H	-6.38505700	2.15276300	-2.26122600
C	-2.71013400	-1.06909700	2.14600000
C	-4.04487600	-1.12816200	2.55042100
C	-1.73660500	-1.83811700	2.79938800
C	-4.41331900	-1.96802300	3.59851600
H	-4.79418200	-0.52004300	2.05089900
C	-2.11418600	-2.67131900	3.84645800
H	-0.69477200	-1.77350900	2.48297300
C	-3.44947400	-2.73864200	4.24269500
H	-5.45125400	-2.01717500	3.91303500
H	-1.36443800	-3.26932200	4.35495800
H	-3.73888900	-3.39178800	5.06072100
C	3.55093500	-0.67488200	-0.08213700
C	4.48593100	0.17619000	-0.68098800
C	3.63135600	-2.05360800	-0.28035500
C	5.50544300	-0.35559300	-1.46251100
H	4.41290900	1.25298000	-0.54106000
C	4.65051100	-2.58160600	-1.07025900
H	2.87816500	-2.69394000	0.17134500
C	5.58570800	-1.73498600	-1.65776900
H	6.23337500	0.30399700	-1.92479000
H	4.70650300	-3.65358700	-1.23306700
H	6.37874700	-2.14719400	-2.27480300
C	2.71032500	1.05595000	2.15277700
C	1.73755700	1.82173000	2.81110900
C	4.04549500	1.11275200	2.55608800
C	2.11634600	2.64957600	3.86198400
H	0.69541500	1.75914600	2.49528800
C	4.41513500	1.94718400	3.60813000
H	4.79425800	0.50741600	2.05237800
C	3.45205600	2.71466200	4.25719800
H	1.36722000	3.24513800	4.37424300
H	5.45341900	1.99465800	3.92175700
H	3.74233600	3.36362900	5.07822200

3u

C	0.33521300	-1.70664600	2.94795000
C	0.56416400	-1.76392600	1.56754400
H	-0.77273100	-0.82222300	4.54859300
C	-0.61555400	-0.84554900	3.47550100
C	-0.15492300	-0.90814300	0.72953500
C	-1.08401700	-0.00428500	1.25839900
C	-1.32337600	0.00463600	2.63124000
H	-2.03726500	0.69964400	3.06125500
C	0.15489400	-0.90823700	-0.72947400
C	-0.56431900	-1.76401700	-1.56740300
C	1.08410400	-0.00455500	-1.25840800
C	-0.33535200	-1.70687400	-2.94781000
C	1.32349000	0.00420700	-2.63124800
C	0.61555700	-0.84596200	-3.47542200
H	2.03746500	0.69909200	-3.06131700
H	0.77272200	-0.82275000	-4.54851800
P	1.97126400	1.13666700	-0.11759200
P	-1.97111400	1.13690000	0.11749700
C	-3.27074100	0.05991200	-0.55385800
C	-4.06268600	-0.76387500	0.25146800
C	-3.35552200	-0.03042000	-1.94378100
C	-4.94762300	-1.66202800	-0.33382600
H	-3.97135600	-0.71663400	1.33491000
C	-4.23250900	-0.94167200	-2.52706300
H	-2.70587600	0.59686500	-2.54865200
C	-5.02760000	-1.75389900	-1.72304700
H	-5.56506600	-2.30039400	0.29089300
H	-4.28791300	-1.02326400	-3.60823000
H	-5.71019400	-2.46592400	-2.17779800
C	-2.70205900	2.36010500	1.23524200

C	-4.07333500	2.49679400	1.44800000
C	-1.79609100	3.22255600	1.86819900
C	-4.54408000	3.49328800	2.30165100
H	-4.77199100	1.83219100	0.94654400
C	-2.27447200	4.21322600	2.71785500
H	-0.72794300	3.09801000	1.68961900
C	-3.64627100	4.34707300	2.93531200
H	-5.61129900	3.60348200	2.46820900
H	-1.57762400	4.88359500	3.21162300
H	-4.01547400	5.12199500	3.60067400
C	3.27083800	0.05971000	0.55389800
C	4.06279300	-0.76416600	-0.25133000
C	3.35562400	-0.03044300	1.94382900
C	4.94774900	-1.66222500	0.33407700
H	3.97144400	-0.71708300	-1.33477700
C	4.23262200	-0.94160900	2.52722600
H	2.70596600	0.59690800	2.54862000
C	5.02773000	-1.75391700	1.72331200
H	5.56519800	-2.30066400	-0.29056000
H	4.28801500	-1.02307500	3.60840400
H	5.71033900	-2.46587000	2.17815200
C	2.70227500	2.35976100	-1.23542300
C	1.79632500	3.22223700	-1.86837300
C	4.07354800	2.49636400	-1.44826200
C	2.27471400	4.21284400	-2.71809800
H	0.72817900	3.09775200	-1.68973900
C	4.54430100	3.49279700	-2.30198100
H	4.77220000	1.83176500	-0.94679900
C	3.64650900	4.34660700	-2.93563200
H	1.57787600	4.88322900	-3.21185900
H	5.61151800	3.60292500	-2.46859500
H	4.01572000	5.12148000	-3.60104600
O	1.16634500	1.76195300	0.98129500
O	-1.16620000	1.76204900	-0.98147200
O	1.52492400	-2.54669200	1.00949400
C	1.41676600	-3.95649000	1.16813200
H	0.45181900	-4.23603300	1.60298400
H	1.51205300	-4.40544500	0.17536500
O	-1.52519900	-2.54652000	-1.00921600
C	-1.41774500	-3.95634200	-1.16811000
H	-0.45292300	-4.23630700	-1.60296800
H	-1.51328200	-4.40538700	-0.17541000
H	-2.22210000	-4.31882300	-1.81276800
H	2.22094500	-4.31947100	1.81273100
Cl	1.30118600	-2.67469200	4.02686200
Cl	-1.30149700	-2.67474600	-4.02673700

3v

C	-0.11222400	2.98279900	-2.28955200
C	-0.44544700	1.63469800	-2.32595400
C	0.89728000	3.41701900	-1.42615200
C	0.21609200	0.71064700	-1.49862400
C	1.19737300	1.16596900	-0.62653100
C	1.55870800	2.51870500	-0.59360100
H	2.31302000	2.86017900	0.10310800
C	-0.21612700	-0.71075900	-1.49854900
C	0.44537900	-1.63487700	-2.32583100
C	-1.19737100	-1.16601600	-0.62637500
C	0.11215600	-2.98297500	-2.28931300
C	-1.55870500	-2.51875000	-0.59333000
C	-0.89731200	-3.41712800	-1.42583900
H	-2.31299200	-2.86016700	0.10343400
P	-1.98474300	0.01987700	0.53550300
P	1.98477300	-0.01985700	0.53539500
C	3.33330400	-0.75960300	-0.42871700
C	4.08203000	-0.05531700	-1.38243200
C	3.61839600	-2.10360900	-0.20117900
C	5.11061900	-0.67905600	-2.06674000
H	3.84638700	0.98475000	-1.59573900
C	4.65124500	-2.74606300	-0.88547700
H	3.00975400	-2.65062300	0.51362500
C	5.40295400	-2.02773700	-1.81729000
H	5.70325300	-0.15373500	-2.80850700
H	4.85291100	-3.79241100	-0.68850400
C	2.72622300	1.06253800	1.78260300
C	4.10207200	1.24030200	1.95551900
C	1.83429600	1.74250500	2.61826700
C	4.58060400	2.08554700	2.94544600
H	4.80442600	0.71520300	1.31349000
C	2.30591500	2.59376000	3.61318700
H	0.76212700	1.61043800	2.47083700
C	3.68464700	2.76472800	3.77747700
H	5.64356400	2.23717300	3.10048300
H	1.59774600	3.11197900	4.24893800
C	-3.33325300	0.75963200	-0.42862800
C	-4.08198900	0.05534200	-1.38233200
C	-3.61832400	2.10364600	-0.20111300
C	-5.11056800	0.67908600	-2.06665100
H	-3.84635900	-0.98473100	-1.59562400
C	-4.65116300	2.74610400	-0.88542100
H	-3.00967500	2.65066300	0.51368400
C	-5.40288500	2.02777400	-1.81722100
H	-5.70321000	0.15376200	-2.80840900
H	-4.85281400	3.79245800	-0.68846400
C	-2.72621900	-1.06246700	1.78273900
C	-1.83430400	-1.74241900	2.61842700
C	-4.10207100	-1.24020700	1.95565500
C	-2.30593700	-2.59363600	3.61337200
H	-0.76213200	-1.61037000	2.47099800
C	-4.58061700	-2.08541200	2.94560800
H	-4.80441500	-0.71511800	1.31360700
C	-3.68467200	-2.76457900	3.77766400
H	-1.59777700	-3.11184300	4.24914300
H	-5.64357900	-2.23701900	3.10064700
O	-1.12115200	1.08221700	1.14854700
O	1.12120700	-1.08220800	1.14845400
O	-1.41712900	1.11802200	-3.11880600
C	-2.27245000	2.02770800	-3.77423500
H	-1.73245000	2.60994000	-4.53139600
H	-3.04093000	1.42476100	-4.25815600
O	1.41702600	-1.11826400	-3.11876600
C	2.27231900	-2.02800100	-3.77416100
H	1.73228600	-2.61029100	-4.53125400
H	3.04077800	-1.42509300	-4.25816200
H	2.74372400	-2.70707400	-3.05279300
H	-2.74382500	2.70683600	-3.05289900
H	0.60956100	-3.72284200	-2.90403500
H	-0.60965400	3.72261900	-2.90431000
O	-1.15421000	-4.75271700	-1.45921900
O	1.15417400	4.75260500	-1.45963900
C	-2.11974000	-5.24602200	-0.55672800
H	-1.84053800	-5.03262700	0.48223500
H	-3.10893600	-4.81591800	-0.75576500
H	-2.15606200	-6.32436500	-0.71217000
C	2.11974200	5.24597100	-0.55722300
H	1.84058500	5.03264500	0.48176600

H	3.10893000	4.81585400	-0.75627300
H	2.15605700	6.32430400	-0.71273900
O	-4.24809000	-3.57027700	4.71422200
O	-6.42632800	2.54764500	-2.54077100
O	4.24805100	3.57046500	4.71401100
O	6.42640200	-2.54760600	-2.54083600
C	6.75327500	-3.90366300	-2.32851100
H	7.59023500	-4.12047000	-2.99185300
H	7.05465400	-4.08280000	-1.28963700
H	5.91062300	-4.56005400	-2.57741600
C	3.38243800	4.26090000	5.58804500
H	2.76979000	3.56560700	6.17405300
H	4.02343700	4.83342700	6.25824300
H	2.72434100	4.94582700	5.03992000
C	-6.75319000	3.90370700	-2.32845900
H	-7.59015500	4.12051200	-2.99179500
H	-7.05455700	4.08285800	-1.28958400
H	-5.91053600	4.56009000	-2.57737900
C	-3.38248800	-4.26070100	5.58827500
H	-2.76983000	-3.56540200	6.17426400
H	-4.02349600	-4.83320100	6.25848800
H	-2.72440000	-4.94565200	5.04016700

3z

C	0.59067400	2.65879700	-2.52093200
C	-0.00956200	1.41031800	-2.41232300
C	1.54868100	3.04448400	-1.57832800
C	0.34447700	0.53016600	-1.37038200
C	1.29947000	0.93690900	-0.44173400
C	1.91610000	2.19014600	-0.54189900
H	2.65467500	2.49289700	0.18828400
C	-0.34151600	-0.78360600	-1.24422100
C	0.01123200	-1.84388600	-2.10251100
C	-1.29321800	-1.00884800	-0.25270200
C	-0.58350400	-3.09256100	-1.96874600
C	-1.90510000	-2.26021000	-0.10999700
C	-1.53603500	-3.29534800	-0.96540500
H	-2.64038600	-2.42042700	0.66720200
P	-1.85737700	0.41210500	0.77587800
P	1.86179800	-0.26052700	0.84160300
O	-0.84580900	1.33933600	1.37838800
O	0.84872500	-1.05318100	1.61073500
O	-0.97810000	0.96947100	-3.24983500
O	0.97285700	-1.56522400	-3.01415800
C	2.88270300	-1.35337900	-0.19021000
C	2.47793100	-2.66384500	-0.24797400
C	3.89080300	-0.87550400	-1.07250800
C	3.03842100	-3.55331200	-1.20066400
H	1.67543100	-3.00075500	0.40533700
C	4.47109100	-1.72176000	-1.97806100
H	4.17851000	0.17287900	-1.04053900
C	2.57313800	-4.88779300	-1.33507000
C	4.05056300	-3.07693400	-2.07929600
H	5.24030800	-1.36167100	-2.65639500
C	3.09179400	-5.71410500	-2.30056700
H	1.78654600	-5.23264500	-0.66872300
C	4.57552100	-3.95819300	-3.05981000
C	4.10617700	-5.24334200	-3.17017800
H	2.72690700	-6.73132300	-2.40289900
H	5.34981700	-3.59365200	-3.73009400
H	4.51043900	-5.90627800	-3.92903000
C	-2.87783100	1.28444200	-0.44854000
C	-2.48139300	2.56361300	-0.74993400
C	-3.87845600	0.64180500	-1.22877600
C	-3.04382600	3.25426900	-1.85427100
H	-1.68361000	3.02259500	-0.16926000
C	-4.46030800	1.29858500	-2.27897800
H	-4.15898600	-0.38412200	-1.00146300
C	-2.58796000	4.54377600	-2.23467000
C	-4.04889800	2.61395700	-2.63110700
H	-5.22367500	0.81157700	-2.88032000
C	-3.10823100	5.17060700	-3.33929700
H	-1.80727800	5.01371700	-1.64197900
C	-4.57561100	3.29178000	-3.76101500
C	-4.11506900	4.53719500	-4.10864400
H	-2.75054700	6.15371100	-3.62895200
H	-5.34416200	2.80193800	-4.35355900
H	-4.52066300	5.04310100	-4.97947200
C	2.91564000	0.73377300	1.92678700
C	4.26804900	0.54106600	2.05771600
C	2.24475500	1.72450400	2.70414500
C	5.02792700	1.33417500	2.96047100
H	4.77953600	-0.22378000	1.47652800
C	2.95932100	2.50183000	3.57441500
H	1.16859500	1.84306100	2.58330700
C	6.42767700	1.16015200	3.11554600
C	4.36421700	2.33224900	3.72638300
H	2.45906900	3.26068500	4.17069200
C	7.13766600	1.94320700	3.98949200
H	6.92578100	0.39271800	2.52867900
C	5.12546100	3.12663600	4.62224300
C	6.47816900	2.93801700	4.75098400
H	8.20810800	1.80270100	4.10219800
H	4.61477900	3.88706300	5.20727800
H	7.04993500	3.55112200	5.44073700
C	-2.91474900	-0.35339400	2.03012000
C	-4.26714600	-0.13643100	2.11525100
C	-2.24966400	-1.17495300	2.98860200
C	-5.03262700	-0.73661000	3.15190200
H	-4.77431400	0.50148700	1.39406700
C	-2.96983600	-1.76648700	3.99051900
H	-1.17333300	-1.31650300	2.89812800
C	-6.43231500	-0.53208700	3.26400200
C	-4.37485600	-1.56768700	4.10063500
H	-2.47399800	-2.39561000	4.72536800
C	-7.14806500	-1.12764200	4.27090300
H	-6.92553700	0.10746000	2.53656700
C	-5.14205100	-2.17007100	5.13099600
C	-6.49460600	-1.95642600	5.21466200
H	-8.21840900	-0.96507700	4.34963400
H	-4.63620800	-2.80305400	5.85538700
H	-7.07088200	-2.42171100	6.00838500
C	-1.26252400	1.74735800	-4.39439300
H	-1.63590400	2.74270600	-4.12709600
H	-0.37237100	1.84768400	-5.02776400
H	-2.04156500	1.20845600	-4.93432900
H	-0.32037100	-3.93220300	-2.60056300
C	1.26034200	-2.54716900	-3.98841100
H	0.37020200	-2.77184500	-4.58906800
H	2.03614700	-2.11841900	-4.62329400
H	1.63883800	-3.47066200	-3.53528200
H	0.32917500	3.36342900	-3.30095000
O	-2.04656300	-4.55375300	-0.89826800
O	2.06572300	4.28950700	-1.75490200
C	-2.98829200	-4.81609200	0.12001900
H	-3.88944000	-4.20331200	-0.00299900

H	-3.25059400	-5.86972500	0.02478300
H	-2.56339200	-4.63081700	1.11382000
C	3.01128200	4.73745800	-0.80739500
H	3.90925300	4.10788700	-0.81218900
H	3.27827200	5.75193700	-1.10331100
H	2.58814700	4.74824300	0.20420800

3x

C	-0.81091300	-2.84107100	-2.32047800
C	-0.11208800	-1.63957200	-2.34317900
C	-1.77300200	-3.05646000	-1.32903100
C	-0.39201700	-0.63144700	-1.39776500
C	-1.35834700	-0.86647800	-0.42384100
C	-2.06119500	-2.07766800	-0.38313900
H	-2.79884800	-2.25238100	0.38814700
C	0.39202600	0.63138700	-1.39786800
C	0.11184200	1.63944900	-2.34326400
C	1.35846100	0.86658800	-0.42408900
C	0.81060100	2.84099200	-2.32080100
C	2.06120500	2.07784000	-0.38357500
C	1.77283200	3.05652600	-1.32953300
H	2.79890100	2.25269400	0.38763800
P	1.82171900	-0.49648800	0.73618100
P	-1.82148300	0.49669900	0.73633400
C	-2.75062300	1.55856600	-0.40863600
C	-2.27289900	2.85960000	-0.53361800
C	-3.69910000	1.03649800	-1.29026000
C	-2.73248300	3.64866600	-1.58896700
H	-1.48936200	3.19128600	0.13934900
C	-4.17138700	1.83621600	-2.32993400
H	-4.00837700	0.00058200	-1.19880800
C	-3.67213600	3.13635300	-2.49076000
C	-2.92331900	-0.33256700	1.90505400
C	-4.31015300	-0.21157300	1.89451500
C	-2.25966700	-1.18595800	2.78873200
C	-5.05779700	-0.97641000	2.79706400
H	-4.79098700	0.46168800	1.19392500
C	-3.01183900	-1.94872200	3.68054200
H	-1.17621300	-1.26038600	2.73367400
C	-4.41087000	-1.83482600	3.69526100
C	2.75050500	-1.55859000	-0.40886600
C	3.69888200	-1.03668700	-1.29069800
C	2.27256800	-2.85955900	-0.53371000
C	4.17085500	-1.83649900	-2.33044400
H	4.00828800	-0.00080000	-1.19936600
C	2.73184000	-3.64871900	-1.58912600
H	1.48909800	-3.19111300	0.13940100
C	3.67142000	-3.13658600	-2.49110100
C	2.92382900	0.33278500	1.90464100
C	4.31065500	0.21173100	1.89394600
C	2.26030600	1.18614700	2.78844700
C	5.05842800	0.97647600	2.79647300
H	4.79138800	-0.46152300	1.19328200
C	3.01260400	1.94881600	3.68023400
H	1.17684500	1.26060600	2.73353900
C	4.41162800	1.83483600	3.69481300
O	0.74854200	-1.27706300	1.43794300
O	-0.74825200	1.27750900	1.43773700
O	0.88583700	-1.37164500	-3.21509900
O	-0.88624800	1.37155100	-3.21499800
O	-4.03167100	3.86080300	-3.59056100
O	-5.14784800	-2.54759000	4.59505000
O	5.14871500	2.54747300	4.59461300
O	4.03060800	-3.86113000	-3.59095500
C	-5.02397700	4.84129700	-3.32125000
H	-5.94641800	4.36664100	-2.96752200
H	-4.66628900	5.56125600	-2.57768100
H	-5.21695400	5.35422100	-4.26444200
C	-5.40711700	-3.87668600	4.17061900
H	-5.99362000	-4.34692100	4.96109900
H	-5.98405600	-3.87813800	3.23780600
H	-4.47111500	-4.42909000	4.03101800
C	5.40803100	3.87658800	4.17027400
H	4.47204000	4.42905200	4.03082500
H	5.99463900	4.34672000	4.96073700
H	5.98487100	3.87809100	3.23740000
C	5.02288300	-4.84171300	-3.32186500
H	5.21560500	-5.35465000	-4.26510100
H	5.94544600	-4.36714800	-2.96833300
H	4.66528600	-5.56164600	-2.57822400
O	2.28504400	-4.90043800	-1.86962900
O	5.07568400	-1.44181700	-3.26105000
O	-2.48621000	-2.83668400	4.55768900
O	-6.41287000	-0.96936600	2.87428200
O	6.41350600	0.96935900	2.87356200
O	2.48712600	2.83675500	4.55749300
O	-5.07639900	1.44140900	-3.26030700
O	-2.28595000	4.90046000	-1.86956100
C	-1.34268600	5.46294100	-0.97554900
H	-1.15042100	6.47102700	-1.34262900
H	-0.40798400	4.89100000	-0.96957200
H	-1.74719100	5.51263600	0.04205800
C	-5.50381800	0.09815900	-3.20191100
H	-4.65818500	-0.59292100	-3.30158700
H	-6.18854000	-0.03734400	-4.03885600
H	-6.03107800	-0.11261300	-2.26254700
C	7.09884700	0.08533400	2.01520200
H	6.80646400	-0.95536400	2.19845400
H	8.15847100	0.21014900	2.23724700
H	6.91622300	0.32758400	0.96023000
C	1.07520400	2.95584500	4.56525600
H	0.69432600	3.27085800	3.58687300
H	0.84389900	3.71077000	5.31619100
H	0.60102800	2.00638100	4.83694200
C	-7.09832700	-0.08522600	2.01613200
H	-8.15792200	-0.21006100	2.23831200
H	-6.91585800	-0.32733500	0.96109900
H	-6.80590600	0.95544500	2.19947800
C	-1.07427400	-2.95561300	4.56536000
H	-0.69341100	-3.27051200	3.58693400
H	-0.84283800	-3.71056300	5.31622900
H	-0.60019500	-2.00610900	4.83708200
C	5.50323500	-0.09860000	-3.20282800
H	4.65763900	0.59254600	-3.30235800
H	6.18778600	0.03679100	-4.03993100
H	6.03072000	0.11218200	-2.26359400
C	1.34220100	-5.46293200	-0.97518200
H	1.74717600	-5.51259000	0.04224100
H	1.14980000	-6.47103000	-1.34215900
H	0.40748200	-4.89102700	-0.96879000
H	0.61933600	3.63746200	-3.02991200
H	-0.61979500	-3.63766000	-3.02948600
C	1.13809300	-2.31411300	-4.24135200
H	2.00495200	-1.93903400	-4.78628000
H	1.38262600	-3.30259200	-3.83704000
H	0.27475400	-2.39259000	-4.91349400

C	-1.13860900	2.31428000	-4.24100100
H	-0.27538300	2.39281000	-4.91328200
H	-2.00563200	1.93945100	-4.78583700
H	-1.38290500	3.30271500	-3.83643200
O	2.36597300	4.28270000	-1.35633800
O	-2.36621500	-4.28260900	-1.35563600
C	3.31402700	4.56322700	-0.34878400
H	4.17056400	3.88032600	-0.40504300
H	3.65144800	5.58421900	-0.52700700
H	2.86794300	4.49123700	0.65086400
C	-3.31406100	-4.56300900	-0.34785200
H	-4.17055600	-3.88004100	-0.40393900
H	-3.65161100	-5.58397900	-0.52595700
H	-2.86773400	-4.49100600	0.65168900

References

[1] Watson, A.J.A.; Atkinson, B.N.; Maxwell, A.C.; Williams, J.M.J. *Adv. Synth. Catal.* **2013**, 355: 734-740.

[2] Kazuo, A.; Naoki, T. Bull. *Chem. Soc. Jpn.* **1982**, 55, 1655 – 1656.

[3] Suzuki, Y.; Hasegawa, Y.; Sato, M.; Saito, M. Yamamoto, N.; Miyasaka, K.; Mikami, T.; Miyazawa, K. Teikoku Hormone Mfg. Co., Ltd. Patent *US4695581*, **1987**, A.

[4] Peng, J.-B.; Chen, B.; Qi, X.; Ying, J.; Wu, X.-F. *Adv. Synth. Catal.* **2018**, *360*, 4153.

[5] N. Sundar ;Bhat, S.V. *Synthetic Communications*, **1998**, 28:12, 2311-2316.

[6] a) Allen, A. E.; MacMillan, D. W. C. *J. Am. Chem. Soc*. **2011**, *133*, 4260. b) Laudadio, G.; Gemoets, H. P. L.; Hessel, V.; Noël, T. *J. Org. Chem*, **2017**, *82* (22), 11735-11741. c) Kitamura, T.; Matsuyuki, J.; Taniguchi, H. *Synthesis*, **1994**, 2, 147-148.

[7] Schumacher, R.; Reißig, H.-U. *Liebigs Annalen* **1997**, *3*, 521-526.

[8] Tu, G.; Huang, H.; Kuang, B.; Li, S.; Xiong, F. *J. Enzyme Inhib. Med. Chem.* **2011**, 26, 222-230.

[9] Kuroda, H.; Hanaki, E.; Izawa, H.; Kano, M.; Itahashi, H. Tetrahedron **2004**, 60, 1913-1920.

[10] Hu, Y; Sun, W.; Zhang, T.; Xu, N.; Xu, J.; Lan, Y.; Liu, C. *Angew. Chem. Int. Ed.* **2019**, *58*, 15813.

[11] Dratch, S.; Charnikhova, T.; Sarabèr, F.C.E.; Jansen, B. J.M.; De Groot, A. *Tetrahedron*, **2003**, 59, 24, 4287 – 4295.

[12] Hellberg, L.; Juarez, A. *Tetrahedron Lett.,* **1974**, 3553-3554.

[13] Vita, M.A.; Waser, J. *Org. Lett.* **2013**, *15*, 3246-3249;

[14] Heathcock, C. H.; Davidsen, S. K.; Hug, K. T.; Flippin, L. A. *J. Org. Chem.* **1986** *51*, 3027-3037.

[15] Smietana, M.; Mioskowski, C. *Org. Lett.* **2001**, 3, 1037-1039.

[16] Huang, Z.; Liu, Z.; Zhou, J. *J. Am. Chem. Soc*. **2011**, 133, 15882-15885.

[17] Adbur-Rashid, K.; JIA, W.; Guo, R.; Chen, X.; Amoroso, Dino. Kanata Chemical Technologies Inc. Patent *WO2012/31358*, **2012**, A1

[18] Adbur-Rashid, K.; JIA, W.; Guo, R.; Chen, X.; Amoroso, Dino. Kanata Chemical Technologies Inc. Patent *WO2012/31358*, **2012**, A1

[19] Lundin, P. M.; Esquivias, J.; Fu, G. C. *Angew. Chem., Int. Ed.* **2009**, 48, 154–156.

[20] Cheon, C. H.; Kanno, O.; Toste, D. *J. Am. Chem. Soc*. **2011**, *133,* 13248.

[21] Lou, S.; Fu, G. *J. Am. Chem. Soc.* **2010**, *132*, 4, 1264–1266.

[22] Peng, C.; Zhang, W.; Yan, G.; Wang, J. *Org. Lett.* **2009,** *11*, 1667-1670.

[23] Cherney, A. H.; Reisman, S. E. *Tetrahedron* **2014**, 70, 3259.

[21] Gaussian 16, Revision C.01, Frisch, M. J.; Trucks, G. W.; Schlegel, H. B.; Scuseria, G. E.; Robb, M. A.; Cheeseman, J. R.; Scalmani, G.; Barone, V.; Petersson, G. A.; Nakatsuji, H.; Li, X.; Caricato, M.; Marenich, A. V.; Bloino, J.; Janesko, B. G.; Gomperts, R.; Mennucci, B.; Hratchian, H. P.; Ortiz, J. V.; Izmaylov, A. F.; Sonnenberg, J. L.; Williams-Young, D.; Ding, F.; Lipparini, F.; Egidi, F.; Goings, J.; Peng, B.; Petrone, A.; Henderson, T.; Ranasinghe, D.; Zakrzewski, V. G.; Gao, J.; Rega, N.; Zheng, G.; Liang, W.; Hada, M.; Ehara, M.; Toyota, K.; Fukuda, R.; Hasegawa, J.; Ishida, M.; Nakajima, T.; Honda, Y.; Kitao, O.; Nakai, H.; Vreven, T.; Throssell, K.; Montgomery, J. A., Jr.; Peralta, J. E.; Ogliaro, F.; Bearpark, M. J.; Heyd, J. J.; Brothers, E. N.; Kudin, K. N.; Staroverov, V. N.; Keith, T. A.; Kobayashi, R.; Normand, J.; Raghavachari, K.; Rendell, A. P.; Burant, J. C.; Iyengar, S. S.; Tomasi, J.; Cossi, M.; Millam, J. M.; Klene, M.; Adamo, C.; Cammi, R.; Ochterski, J. W.; Martin, R. L.; Morokuma, K.; Farkas, O.; Foresman, J. B.; Fox, D. J. Gaussian, Inc., Wallingford CT, 2016.

[22] Molecular Modeling Pro Version 6.3.6; Quinn, J. A.; ChemSW, Inc.: Fairfield , CA.

[23] Falivene, L.; Cao, Z.; Petta, A.; Serra, L.; Poater, A.; Oliva, R.; Scarano, V.; Cavallo, L., Towards the online computer-aided design of catalytic pockets. Nat. Chem. 2019, 11, 872-879.

[24] MATLAB and Statistics Toolbox Release R2020a, The MathWorks, Inc., Natick, Massachusetts, United States.

[24] a) Chung, J. Y.; Mancheno, D.; Dormer, P. G.; Variankaval, N.; Ball, R. G.; Tsou, N. N. *Org. Lett.* **2008**, 10, 3037–3040. b) Bandar, J. S.; Ascic, E.; Buchwald, S.L. *J. Am. Chem. Soc.* **2016**,*138*, 8, 5821-5824.

[25] Huang, Z.; Lim, L. H.; Chen, Z.; Li, Y.; Zhou, F.; Su, H.; Zhou, J., *Ang. Chem. Int. Ed*. **2013**, *52*, 4906.

[26] a) Piccolo, O.; Azzena, U.; Melloni, G.; Delogu, G.; Valoti, E. J. Org. Chem. 1991, 56, 183. b) Mao, J.; Liu, F.; Wang, M.; Wu, L.; Zheng, B.; Liu, S.; Zhong, J.; Bian, Q.; Walsh, P. *J. Am. Chem. Soc*. **2014,** 136, 17662-17668.
[27] Gandolfo, E.; Tang, X.; Raha Roy, S.; Melchiorre, P. *Angew. Chem. Int. Ed*. **2019**, 58, 16854.
